# Supplementary material for: Appraisal of clinical practice guidelines and consensus statements on obstetric anaesthesia: a systematic review using the AGREE II instrument
Source: BMJ Open. 2024 May 28;14(5):e084759. doi: 10.1136/bmjopen-2024-084759 (PMC11138284; doi:10.1136/bmjopen-2024-084759)
Supplement: Supplementary data [file bmjopen-2024-084759supp001.pdf]

Supplementary Materials

Contents

Supplementary Table 1. Search strategy in PubMed.
Supplementary Table 2. Search strategy in EMBASE using the OVID interface.
Supplementary Table 3. Searches in Chinese databases.
Supplementary Table 4. Searches in guideline databases.
Supplementary Table 5. Excluded studies and reasons for exclusion.
Supplementary methods. How to calculate the scores.
Supplementary Table 6. Scores for each individual AGREE II domain items by each reviewer.
Supplementary Table 7. Domain score for each included guidance document.
Supplementary Table 8. Mean scores across reviewers for the individual AGREE II domain items.
Supplementary Table 9. The intraclass correlation coefficient between reviewers for each individual AGREE II domain item.
Supplementary Table 10. Summary of recommendations for the peri-anesthetic evaluation and preparation by included guidance document.
Supplementary Table 11. Summary of recommendations for the anesthetic care for labor and vaginal delivery by included guidance document.
Supplementary Table 12. Summary of recommendations for the anesthetic care for cesarean delivery by included guidance document.
Supplementary Table 13. Summary of recommendations for the monitoring and management of obstetric and anesthetic or intra- or postoperative pain management by included guidance document.
Supplementary Table 14. Summary of recommendations for the management of adverse events by included guidance document.
Supplementary Table 15. Summary of recommendations for the management of obstetric and anesthetic emergencies by included guidance document.

Huang L, *et al. BMJ Open* 2024; 14:e084759. doi: 10.1136/bmjopen-2024-084759

Supplementary Table 1. Search strategy in PubMed.

1	((((((cesarean[Title/Abstract]) OR (cesarean delivery[Title/Abstract])) OR (cesarean section[Title/Abstract])) OR (obstetric[Title/Abstract])) OR (obstetrical delivery[Title/Abstract])) OR (obstetric anesthesia[Title/Abstract])) OR (delivery[Title/Abstract])	633,790
2	((expert consensus[Title/Abstract]) OR (guideline[Title/Abstract])) OR (recommendation statement[Title/Abstract])	88,342
3	("2000/01/01"[Date - Publication]: "3000"[Date - Publication])	22,218,196
4	1 AND 2 AND 3	3,181

Date of literature search: June 6, 2023

Supplementary Table 2. Search strategy in EMBASE using the OVID interface.

1	cesarean.m_titl.	26,523
2	cesarean delivery.m_titl.	8,147
3	cesarean section.m_titl.	12,708
4	obstetric.m_titl.	23,566
5	obstetrical delivery.m_titl.	16
6	obstetric anesthesia.m_titl.	961
7	delivery.m_titl.	185,141
8	1 or 2 or 3 or 4 or 5 or 6 or 7	224,835
9	guideline.m_titl.	29,968
10	expert consensus.m_titl.	3,906
11	recommendation statement.m_titl.	538
26	9 or 10 or 11	34,373
21	8 and 12	183

Date of literature search: Embase <1974 to 2023 June 06>

Huang L, *et al. BMJ Open* 2024; 14:e084759. doi: 10.1136/bmjopen-2024-084759

Supplementary Table 3. Searches in Chinese databases.

**Chinese Biomedical Literature Database**			
[Original search term in Chinese] (cesarean OR obstetric OR obstetric anesthesia) AND (guideline OR consensus OR statement OR recommendation)	Results found	Full text screened	Included documents
	357	2	1
Date of literature search: June 8, 2023			
**Wanfang Data**			
[Original search term in Chinese] (cesarean OR obstetric OR obstetric anesthesia) AND (guideline OR consensus OR statement OR recommendation)	Results found	Full text screened	Included documents
	32	2	2
Date of literature search: June 8, 2023			
**Chinese Medical Association Database**			
[Original search term in Chinese] (cesarean OR obstetric OR obstetric anesthesia) AND (guideline OR consensus OR statement OR recommendation)	Results found	Full text screened	Included documents
	249	4	2
Date of literature search: June 8, 2023			

Supplementary Table 4. Searches in guideline databases.

Databases	Date of search	Search strategy	Results found	Full text screened	Included documents	URL
National Guideline Clearinghouse	2023/6/7	cesarean OR cesarean section OR cesarean delivery OR obstetric OR obstetrical delivery OR obstetric anesthesia OR delivery	75	1	0	www.guideline.gov
Guidelines International Network	2023/6/7	cesarean OR cesarean section OR cesarean delivery OR obstetric OR obstetrical delivery OR obstetric anesthesia OR delivery Search mode: Guidelines	5	1	1	www.g-i-n.net
National Institute for Health and Care Excellence	2023/6/7	cesarean OR obstetric filter type: guidance	77	3	1	www.nice.org.uk
Scottish Intercollegiate Guidelines Network	2023/6/7	Not applicable	1	0	0	www.sign.ac.uk/our-guidelines.html
Turning Research Into Practice Database	2023/6/7	cesarean OR cesarean section OR obstetric OR obstetric anesthesia filter: Guidelines	2,905	11	6	www.tripdatabase.com
Epistemonikos database	2023/6/7	cesarean OR obstetric OR obstetric anesthesia filter: Broad syntheses OR Structured summaries	5	1	1	www.epistemonikos.org

Huang L, *et al. BMJ Open* 2024; 14:e084759. doi: 10.1136/bmjopen-2024-084759

Supplementary Table 5. Excluded studies and reasons for exclusion.

Study	Reason for exclusion
Caughey AB, Sundaram V, Kaimal AJ, et al. Maternal and neonatal outcomes of elective induction of labor. Evidence Report/Technology Assessment No. 176. (Prepared by the Stanford University-UCSF Evidenced-based Practice Centre under contract No. 290-02-0017.) AHRQ Publication No. 09-E005. Rockville, MD.: Agency for Healthcare Research and Quality. March 2009.	Not providing specific recommendations for obstetric anesthesia
Association of Ontario Midwives. Vaginal birth after previous low-segment cesarean section. 2021; (Clinical Practice Guideline No. 14).	Not providing specific recommendations for obstetric anesthesia
Obstetric Care Consensus: Safe prevention of the primary cesarean delivery. March 2014	Not providing specific recommendations for obstetric anesthesia
Guidelines for neuraxial analgesia or anesthesia in obstetrics. American Society of Anesthesiologists, October 13, 2021.	Conference abstract
Optimal goals for anesthesia care in obstetrics. American Society of Anesthesiologists, October 13, 2021.	Conference abstract
NICE guideline: Preterm labor and birth. 10 June 2022	Not providing specific recommendations for obstetric anesthesia
Y Gu, YB Xu, CH, Wang, et al. The reading of the United States Society for Obstetric Anesthesia and Perinatology: consensus statement on the management of cardiac arrest in pregnancy. Journal of Chinese Physician 2017; 19(9): 1286-1293.	Secondary interpretation of foreign guidelines, not original guidelines
Interpretation of the American College of Obstetricians and Gynecologists: 2017 Obstetric Analgesia and Anesthesia Practice Guide. Journal of practical obstetrics and gynecology 2017; 33 (8):586-589.	Secondary interpretation of foreign guidelines, not original guidelines
Expert Consensus on Obstetric Anesthesia in China (2008) and (2017)	Replaced by updated versions from the same organization
Hawkins JL. et al. Practice guidelines for obstetric anesthesia: an update report by the American Society of Anesthesiologists Task Force on Obstetric Anesthesia. Anesthesiology 2007; 106: 843–63.	Replaced by updated versions from the same organization

Opderbecke HW. et al. Vereinbarung über die Zusammenarbeit in der operativen Gynäkologie und in der Geburtshilfe. Anästh Intensivmed 1996; 37: 414–8.	Not English
Opderbecke HW. et al. Airway management. Anästh Intensivmed 2004; 45: 302–6.	Not English
Gogarten W. et al. Regionalanästhesieverfahren in der Geburtshilfe. Anästh Intensivmed 2004; 45: 151–3.	Not English
T. Piepho1. et al. S1 guidelines on airway management. Guideline of the German Society of Anesthesiology and Intensive Care Medicine. Anesthetist. 2015 Dec;64 Supple 1:27-40.	Not providing specific recommendations for obstetric anesthesia
The National Institute of Clinical Excellence (NICE) guidelines for cesarean sections: implications for the Anesthetist (2004), (2011) and (2014)	Replaced by updated versions from the same organization
Intrapartum Care: Care of Healthy Women and Their Babies During Childbirth (2014)	Replaced by updated versions from the same organization
Osborne, L. et al. Evidence-based anesthesia: fever of unknown origin in parturient and neuraxial anesthesia.	Replaced by updated versions from the same organization
Kearsley, A. et al. The development of a local guideline for epidural insertion in obstetric patients with signs of maternal sepsis. Anesthesia 2015. 70: 56.	Abstract
Korneliussen, J. et al. Implementation of new guideline recommendations, using ultra-low dose intrathecal morphine, for post-cesarean analgesia and its impact on pain, nausea and pruritus. Acta anesthesiologic Scandinavica 2023. 67: 537-538.	Abstract
Nasser, L. et al. IN SITU SIMULATION TO FACILITATE INTRODUCTION OF A NEW OBSTETRIC RAPID SEQUENCE OF ANESTHESIA GUIDELINE. BMJ Simulation and Technology Enhanced Learning 2017. 3: A3.	Abstract
Cohen, J. et al. Implementation of the Association of Anesthetists safety guideline: neurological monitoring associated with obstetric neuraxial block (2020): improving patient safety in a district general hospital labor ward requires a multidisciplinary and multimodal approach. Anesthesia 2022. 77: 12.	Conference abstract
McGuire, R. et al. Changing of the weather? Introduction of an evidence-based guideline for obstetric general anesthesia after regional surveys and the use of simulation to facilitate guideline implementation at University Hospital South Manchester. Anesthesia 2017. 72: 87.	Conference abstract

Han, W. Y. et al. Anesthesia and perioperative management for cesarean section in patient with factor XI deficiency: a case report. Beijing da xue xue bao. Yi xue ban. Health sciences 2014. 46: 329-332.	Case report
McDonnell, N. J. et al. Acute magnesium toxicity in an obstetric patient undergoing general anesthesia for cesarean delivery. International journal of obstetric anesthesia 2010. 19: 226-231.	Case report
Oji-Zurmeyer, J. et al. National survey of obstetric anesthesia clinical practices in the republic of Austria. International journal of obstetric anesthesia 2019. 39: 95-98.	Primary study
Terblanche, N. C. et al. A survey of Anesthetists on uterotonic usage practices for elective cesarean section in Australia and New Zealand. Anesthesia and intensive care 2021. 49: 440-447.	Primary study
Terfasa, E. A. et al. Obstetric analgesia utilization in labor pain management and associated factors among obstetric care providers in the West Shewa Zone, Central Ethiopia. SAGE open medicine 2022. 10: 20503121221088705.	Primary study
Thuillier, C. et al. Impact of recommended changes in labor management for prevention of the primary cesarean delivery. American journal of obstetrics and gynecology 2018. 218: 341.e341- e349.	Primary study
Cobb, B. et al. Do obstetric Anesthesiologists provide more guideline-concordant care for patients undergoing cesarean delivery? Anesthesia and analgesia 2018. 126: 404-408.	Primary study
Fiedler, A. et al. Adverse effect of delayed pushing on postpartum blood loss in nulliparous women with epidural analgesia. International journal of gynecology and obstetrics: the official organ of the International Federation of Gynecology and Obstetrics 2020. 150: 92-97.	Primary study
Gunka, V. et al. Determination of ChloraPrep® drying time before neuraxial anesthesia in elective cesarean delivery. A prospective observational study. International journal of obstetric anesthesia 2019. 38: 19-24.	Primary study
Kinsella, S. M. et al. Category-1 cesarean section: a survey of anesthetic and peri-operative management in the UK. Anesthesia 2010. 65: 362-368.	Primary study
Lee, E. K. et al. Investigating a Needle-Based Epidural Procedure in Obstetric Anesthesia. AMIA ... Annual Symposium proceedings. AMIA Symposium 2018. 2018: 720-729.	Primary study
Nasrallah, F. K. et al. The 30-minute decision-to-incision interval for emergency cesarean delivery: fact or fiction? American journal of perinatology 2004. 21: 63-68.	Primary study
Ward, C. et al. Does the presence of epidural analgesia reduce the risk of neonatal sepsis in the setting of an intrapartum fever? The journal of maternal-fetal & neonatal medicine: the official journal of the European	Primary study

Association of Perinatal Medicine, the Federation of Asia and Oceania Perinatal Societies, the International Society of Perinatal Obstet 2022. 35: 2110-2115.	
Jejaw, M. et al. Comprehensive emergency management of obstetric and newborn care program implementation at University of Gondar Comprehensive Specialized Hospital, Northwest Ethiopia, 2021: an evaluation study. Reproductive health 2023. 20: 76.	Primary study
Ji, H. et al. Factors contributing to the rapid rise of cesarean section: a prospective study of primiparous Chinese women in Shanghai. BMJ open 2015. 5: e008994.	Primary study
Johri, M. et al. A cluster-randomized trial to reduce cesarean delivery rates in Quebec: cost-effectiveness analysis. BMC medicine 2017. 15: 96.	Primary study
McDougall, A. R. A. et al. Expert consensus on novel medicines to prevent preterm birth and manage preterm labor: Target product profiles. BJOG: an international journal of obstetrics and gynecology 2022.	Not providing specific recommendations for obstetric anesthesia
Sultan, P. et al. Expert Consensus Regarding Core Outcomes for Enhanced Recovery after Cesarean Delivery Studies: A Delphi Study. Anesthesiology 2022. 137: 201-211.	Not providing specific recommendations for obstetric anesthesia
Bremerich, D. et al. S1 Guideline: Obstetric analgesia and anesthesia. Anesthesiology und Intensivmedizin 2020. 61: S300-S340.	Not English
Lavery, J. P. et al. Is the obstetric guideline of 30 minutes from decision to incision for Cesarean delivery clinically significant? Journal of healthcare risk management: the journal of the American Society for Healthcare Risk Management 1999. 19: 11-20.	Pre 2000
Santerre, R. E. The effect of the ACOG guideline on vaginal births after cesarean. Medical care research and review : MCRR 1996. 53: 315-329.	Pre 2000
Morris, J. M. et al. Protocol for the immediate delivery versus expectant care of women with preterm prelabor rupture of the membranes close to term (PPROMT) Trial [ISRCTN44485060]. BMC pregnancy and childbirth 2006. 6: 9.	Protocol
Gochnour, G. et al. The UTAH VBAC Study. Maternal and child health journal 2005. 9: 181-188.	Review
King, V. J. et al. Clinical practice guideline executive summary: Labor after cesarean/planned vaginal birth after cesarean. Annals of family medicine 2015. 13: 80-81.	Review

Huang L, *et al. BMJ Open* 2024; 14:e084759. doi: 10.1136/bmjopen-2024-084759

Keag, O. E. et al. Long-term risks and benefits associated with cesarean delivery for mother, baby, and subsequent pregnancies: Systematic review and meta-analysis. PLoS medicine 2018. 15: e1002494.	Review
Gulbransen, K. et al. Scoping Review of Best Practice Guidelines for Care in the Labor and Birth Setting of Pregnant Women Who Use Methamphetamines. Journal of obstetric, gynecologic, and neonatal nursing: JOGNN 2022. 51: 141-152.	Review
Hocaloski, S. et al. Perinatal Care for Women with Spinal Cord Injuries: A Collaborative Workshop for Consensus on Care in Canada. Topics in spinal cord injury rehabilitation 2017. 23: 386-396.	Review
Hong, J. et al. Comparison of Maternal Labor-Related Complications and Neonatal Outcomes Following Elective Induction of Labor at 39 Weeks of Gestation vs Expectant Management: A Systematic Review and Meta-analysis. JAMA network open 2023. 6: e2313162.	Review
Indermuhle, P. et al. Use of Scheduled Nonopioid Analgesia to Decrease Inpatient Opioid Consumption After Scheduled Cesarean Birth. Nursing for women's health 2022. 26: 344-352.	Review
James, B. et al. Obstetric general anesthetic safety checklist: Guideline development through team simulation. International journal of obstetric anesthesia 2015. 24: S24.	Review
Kuehn, B. M. Obstetrics group relaxes guideline for trial of labor after cesarean delivery. Jama 2010. 304: 951-952.	Review
Lagro-Janssen, T. Pain management during labor--an informed choice. Nederlands tijdschrift voor geneeskunde 2009. 153: A742.	Review
Mash, R. et al. Alternative mechanisms for delivery of medication in South Africa: A scoping review. South African family practice : official journal of the South African Academy of Family Practice/Primary Care 2021. 63: e1-e8.	Review
Melman, S. et al. Identification of barriers and facilitators for optimal cesarean section care: perspective of professionals. BMC pregnancy and childbirth 2017. 17: 230.	Review
Morris, S. et al. Breech presentation management: A critical review of leading clinical practice guidelines. Women and birth : journal of the Australian College of Midwives 2022. 35: e233-e242.	Review
Mutlu, S. et al. Evaluation of pregnancy, delivery, and postpartum effectiveness of maternity school trainings organized based on the guideline of ministry of health in Turkey: A comparative study. International journal of preventive medicine 2021. 12: 173.	Review

Heslehurst, N. et al. GestationaL Obesity Weight management: Implementation of National Guidelines (GLOWING): a pilot cluster randomized controlled trial of a guideline implementation intervention for the management of maternal obesity by midwives. Pilot and feasibility studies 2018. 4: 47.	Review
Lyerly, A. D. et al. Toward an ethically responsible approach to vaginal birth after cesarean. Seminars in perinatology 2010. 34: 337-344.	Review
Nasser, L. et al. Farewell thiopentone? Northwest deanery surveys of obstetric general anesthesia highlight increasing popularity of induction with propofol. Would a region-wide guideline improve training and safety? Anesthesia 2017. 72: 94.	Review
Neuhaus, S. et al. "Why mothers die". Learning from the analysis of anesthesia-related maternal deaths (1985-2013). Der Anesthetist 2016. 65: 281-294.	Review
Panda, S. et al. Review of cesarean section on maternal request in a tertiary care institute; scenario in developing country. Kathmandu University medical journal (KUMJ) 2013. 11: 349-354.	Review
Reale, S. C. et al. The Most Influential Publications in Obstetric Anesthesiology, 1998-2017: Utilizing the Delphi Method for Expert Consensus. Anesthesia and analgesia 2020. 131: 239-244.	Review
Roderick, E. et al. A national survey of neurological monitoring practice after obstetric regional anesthesia in the UK. Anesthesia 2017. 72: 755-759.	Review
Rungreungvanich, M. et al. An analysis of intraoperative recall of awareness in Thai Anesthesia Incidents Study (THAI Study). Journal of the Medical Association of Thailand 2005. 88 Supple 7: S95-101.	Review
Sakoda, A. et al. Review of 197 cases of urgent cesarean section performed in 2010 using NICE classification. Masui. The Japanese journal of Anesthesiology 2014. 63: 1339-1343.	Review
Sommerness, S. A. et al. The Perinatal Birth Environment: Communication Strategies and Processes for Adherence to a Standardized Guideline in Women Undergoing Second-Stage Labor With Epidural Anesthesia. The Journal of perinatal & neonatal nursing 2017. 31: 41-50.	Review
Terui, K. Obstetric anesthesia: from basics to recent advances in neonatal resuscitation: from Apgar score to NCPR program. Masui. The Japanese journal of Anesthesiology 2010. 59: 293-302.	Review
Van Gorp, S. F. J. et al. Do we need a national guideline for obstetric analgesia in the Netherlands? Evaluation of a questionnaire among dutch Anesthetists. Nederlands Tijdschrift voor Anesthesiology 2005. 18: 100-103.	Review
Williamson, R. M. et al. Availability of lipid emulsion in obstetric anesthesia in the UK: a national questionnaire	Review

survey. Anesthesia 2008. 63: 385-388.	
Winther, L. P. et al. Failed intubation and subsequent airway management during Cesarean section in general anesthesia. Ugeskrift for laeger 2010. 172: 1914-1916.	Review
Friedman, C. L. et al. Resident Attitudes Towards Cesarean Delivery in Canadian Obstetrics and Gynecology Residency Programs. Journal of obstetrics and gynecology. JOGC 2020. 42: 16-24.	Review
Bagou, G. et al. Guidelines for the management of urgent obstetric situations in emergency medicine, 2022. Anesthesia, critical care & pain medicine 2022. 41: 101127.	Not providing specific recommendations for obstetric anesthesia
Crawford, C. New guideline covers labor, vaginal birth after cesarean. Annals of family medicine 2015. 13: 186-187.	Not providing specific recommendations for obstetric anesthesia
Elammary, M. N. et al. Middle eastern college of obstetricians and gynecologists (MCOG) practice guidelines: Role of prediction models in management of trial of labor after cesarean section. Practice guideline no. 05-O-22. Journal of gynecology obstetrics and human reproduction 2023. 52: 102598.	Not providing specific recommendations for obstetric anesthesia
Fischer, T. et al. From the OEGGG - S3 Guideline "Vaginal delivery at term": The position of the Austrian Society of Gynecology and Obstetrics (OEGGG). Geburtshilfe und Frauenheilkunde 2021. 81: 857-861.	Not providing specific recommendations for obstetric anesthesia
Gagnon, R. et al. SOGC CLINICAL PRACTICE GUIDELINE: guidelines for the management of vasa previa. International journal of gynecology and obstetrics: the official organ of the International Federation of Gynecology and Obstetrics 2010. 108: 85-89.	Not providing specific recommendations for obstetric anesthesia
Hey, J. Clinical practice guideline on vaginal delivery of breech presentation. Journal of obstetrics and gynecology Canada. 2009. 31: 1123.	Not providing specific recommendations for obstetric anesthesia
Jain, V. et al. Guideline No. 402: Diagnosis and Management of Placenta Previa. Journal of obstetrics and gynecology Canada. 2020. 42: 906-917.e901.	Not providing specific recommendations for obstetric anesthesia
Jain, V. et al. Guidelines for the Management of a Pregnant Trauma Patient. Journal of obstetrics and gynecology Canada. 2015. 37: 553-574.	Not providing specific recommendations for obstetric

	anesthesia
Jain, V. et al. Guideline No. 439: Diagnosis and Management of Vasa Previa. Journal of obstetrics and gynecology Canada. 2023.	Not providing specific recommendations for obstetric anesthesia
Ladhani, N. N. N. et al. No. 347-Obstetric Management at Borderline Viability. Journal of obstetrics and gynecology Canada. 2017. 39: 781-791.	Not providing specific recommendations for obstetric anesthesia
Lalonde, A. B. Vaginal breech delivery guideline: the time has come. Journal of obstetrics and gynecology Canada. 2009. 31: 483-486.	Not providing specific recommendations for obstetric anesthesia
Marte, K. et al. Reduction in the Cesarean Delivery Rate after Obstetric Care Consensus Guideline Implementation. Obstetrics and gynecology 2016. 128: 1445.	Not providing specific recommendations for obstetric anesthesia
Maxwell, C. et al. SOGC Clinical Practice Guideline. Management guidelines for obstetric patients and neonates born to mothers with suspected or probable severe acute respiratory syndrome (SARS). No. 225, April 2009. International journal of gynecology and obstetrics: the official organ of the International Federation of Gynecology and Obstetrics 2009. 107: 82-86.	Not providing specific recommendations for obstetric anesthesia
Nelson, G. et al. Guideline for perioperative obstetrical care highlights evidence gap related to timing of urinary catheter removal after elective cesarean delivery. American journal of obstetrics and gynecology 2020. 222: 635.	Not providing specific recommendations for obstetric anesthesia
Oliver, E. et al. Comparison of international guidelines on the management of twin pregnancy. European journal of obstetrics, gynecology, and reproductive biology 2023. 285: 97-104.	Not providing specific recommendations for obstetric anesthesia
Podrasky, A. E. et al. ACR appropriateness Criteria® second and third trimester bleeding. Ultrasound quarterly 2013. 29: 293-301.	Not providing specific recommendations for obstetric anesthesia
Robinson, D. et al. Guideline No. 431: Postpartum Hemorrhage and Hemorrhagic Shock. Journal of obstetrics and gynecology Canada. 2022. 44: 1293-1310.e1291.	Not providing specific recommendations for obstetric

	anesthesia
Scott-Warren, V. et al. Development of a fibrinogen concentrate guideline for use in major obstetric Hemorrhage. Transfusion Medicine 2016. 26: 30.	Not providing specific recommendations for obstetric anesthesia
Stafford, I. A. et al. Association between Maternal Obesity Class, Adherence to Labor Guidelines, and Perinatal Outcomes. AJP reports 2021. 11: e105-e112.	Not providing specific recommendations for obstetric anesthesia
Triebwasser, J. et al. Effect of Single-Unit Transfusion Guideline on Red Blood Cell Transfusion for Obstetric Patients. Obstetrics and gynecology 2023. 141: 59S.	Not providing specific recommendations for obstetric anesthesia
Zhu, L. et al. The Chinese guideline for prevention of pelvic and abdominal adhesions after obstetric and gynecologic surgery (2023 edition). Zhonghua fu chan ke za zhi 2023. 58: 161-169.	Not providing specific recommendations for obstetric anesthesia
Bonnet, M. P. et al. Reprint of: Severe pre-eclampsia: guidelines for clinical practice from the French Society of anesthesiology and intensive care (SFAR) and the French College of gynaecologists and obstetricians (CNGOF). Gynecol Obstet Fertil Senol. 2022 Jan;50(1):2-25.	Not English

Reason for exclusion: Review; Primary study; Editorial; Conference abstract; Not providing specific recommendations for obstetric anesthesia; Multimedia section; Replaced by updated versions from the same organization; Conference abstract; Comment letter; Technology appraisal; Book; Not released by a professional association.

Supplementary methods. How to calculate the scores.

The reviewers individually assessed each item using a Likert scale, and the scores were combined to calculate the domain scores. The total score for each domain was calculated by summing the scores of all the items within that domain for each reviewer. This total score was then standardized as a percentage of the maximum possible score for that specific domain. The calculation method is as follows:

$$\frac{Obtained\ score - Minimum\ possible\ score}{Maximum\ possible\ score - Minimum\ possible\ score} * 100\%$$

The minimum possible score is calculated as: (number of questions) × (number of reviewers) × 1. The maximum possible score is calculated as: (number of questions) × (number of reviewers) × 7.

Five guidance documents were selected for the pilot scoring phase, and discussions were held to clarify scoring criteria. After completing scoring for all included documents, a meeting was held to discuss items with scores differing by more than one point. Reviewers were then given the opportunity to revise their scores or maintain their original scores, with all original and revised scores, along with reasons for modifying scores, meticulously recorded for quality control purposes.

Huang L, *et al. BMJ Open* 2024; 14:e084759. doi: 10.1136/bmjopen-2024-084759

Supplementary Table 6. Scores for each individual AGREE II domain items by each reviewer.

Document	Domain 1			Domain 2			Domain 3								Domain 4			Domain 5				Domain 6	
**Item**	**1**	**2**	**3**	**4**	**5**	**6**	**7**	**8**	**9**	**10**	**11**	**12**	**13**	**14**	**15**	**16**	**17**	**18**	**19**	**20**	**21**	**22**	**23**
OG-OG-CMA_2014																							
Rev-1	4	4	7	2	1	3	1	1	1	1	3	1	1	1	5	1	1	1	1	1	1	1	1
Rev-2	4	3	7	2	3	2	1	1	1	1	3	1	1	1	5	1	1	1	1	1	1	1	1
Rev-3	4	3	7	3	3	2	1	1	1	1	4	1	1	1	5	1	1	1	1	1	1	1	1
Rev-4	3	4	7	2	1	3	1	1	1	1	3	1	1	1	4	1	1	1	1	1	1	1	1
OG-A-CMA_2016																							
Rev-1	5	6	5	6	6	3	1	1	1	1	6	1	1	1	5	6	3	1	5	1	1	1	1
Rev-2	6	4	4	6	6	4	1	1	1	1	6	1	1	1	6	6	3	1	4	1	1	1	1
Rev-3	5	6	5	6	6	3	1	1	1	1	5	1	1	1	5	6	3	1	4	1	1	1	1
Rev-4	5	4	5	6	6	3	1	1	1	1	6	1	1	1	5	5	3	1	4	1	1	1	1
ASA_2016																							
Rev-1	7	7	7	7	6	7	7	7	6	7	7	6	6	6	6	6	6	6	6	1	1	6	6
Rev-2	7	7	7	7	7	7	7	6	6	7	7	6	7	6	6	6	6	6	6	1	1	6	6
Rev-3	7	7	7	6	6	7	7	7	6	7	6	6	6	6	6	6	5	6	6	1	1	5	6
Rev-4	7	7	7	7	7	6	7	6	6	7	7	6	7	6	6	5	6	6	6	1	1	5	6
AAGBI_2017																							
Rev-1	5	5	5	6	1	1	2	2	2	4	2	1	1	1	6	5	5	2	1	1	1	2	3
Rev-2	5	5	5	6	1	1	2	2	2	4	2	1	1	1	6	5	5	2	1	1	1	2	3
Rev-3	4	5	5	5	1	1	3	2	2	5	2	1	1	1	5	6	5	2	1	1	1	3	2
Rev-4	5	5	6	6	1	1	2	3	2	4	3	1	1	1	6	5	6	2	1	1	1	2	3
ERAS_2018 (Part 1)																							
Rev-1	6	6	6	5	4	4	6	6	6	6	6	6	4	3	5	5	5	2	5	1	2	2	6
Rev-2	5	6	5	5	4	3	6	6	5	6	6	5	3	3	5	5	4	2	5	1	2	3	5
Rev-3	5	6	6	5	4	3	6	5	6	5	6	6	4	3	5	3	5	2	5	1	2	2	6
Rev-4	6	5	6	5	3	4	6	6	6	6	5	6	3	3	6	3	5	2	5	1	2	2	5

Huang L, *et al. BMJ Open* 2024; 14:e084759. doi: 10.1136/bmjopen-2024-084759

ERAS_2018 (Part 2)																							
Rev-1	6	6	6	5	4	4	6	6	6	6	6	6	4	3	5	5	5	2	5	1	2	2	6
Rev-2	6	6	4	5	3	4	5	6	4	6	5	6	4	3	5	6	5	2	4	1	2	3	6
Rev-3	5	5	6	5	4	3	5	6	5	6	6	5	4	3	5	5	6	2	5	1	2	2	5
Rev-4	6	6	5	5	4	4	6	6	5	6	6	6	4	3	4	6	5	2	6	1	3	3	6
ERAS_2018 (Part 3)																							
Rev-1	6	6	6	5	4	4	6	6	6	6	6	6	4	3	5	5	5	3	5	1	2	1	6
Rev-2	6	6	5	3	4	5	6	6	5	6	5	6	3	3	5	4	5	4	5	1	3	1	5
Rev-3	5	6	6	5	4	4	5	6	6	5	6	5	4	3	6	5	6	3	6	1	2	1	6
Rev-4	6	5	6	6	4	4	6	5	6	6	6	6	3	3	5	6	5	4	5	1	3	1	5
ACOG_2019																							
Rev-1	7	6	5	5	4	5	6	6	5	5	5	4	3	5	6	6	5	4	4	1	1	6	6
Rev-2	7	5	5	4	4	4	6	5	5	4	4	3	3	4	6	6	4	5	5	1	1	5	6
Rev-3	7	6	6	5	5	5	5	6	4	5	5	4	4	5	5	6	5	4	4	1	1	6	5
Rev-4	7	6	5	5	4	5	6	5	5	5	4	3	3	5	6	5	4	4	4	1	1	5	5
SOGC_2019 (Delivery and Postpartum Care)																							
Rev-1	6	6	7	6	3	7	7	6	6	6	6	6	3	6	6	6	6	4	3	1	1	2	6
Rev-2	5	5	7	5	3	7	7	5	5	5	6	5	4	5	4	5	5	3	3	1	1	2	6
Rev-3	6	5	6	5	4	7	7	5	4	5	5	6	3	5	5	6	5	4	4	1	1	2	5
Rev-4	5	5	6	6	3	7	7	5	6	6	5	5	4	6	6	5	6	3	3	1	1	2	6
SOGC_2022 (Twin Pregnancies)																							
Rev-1	6	6	6	6	3	7	7	6	6	6	6	6	5	6	6	6	6	4	3	1	1	2	6
Rev-2	5	5	6	6	3	7	7	5	5	6	6	5	4	5	6	5	5	3	3	1	1	2	6
Rev-3	5	5	5	5	4	6	7	5	4	6	5	6	4	5	5	6	5	4	4	1	1	2	5
Rev-4	6	6	6	5	3	7	7	5	6	6	5	5	5	6	6	5	6	4	3	1	1	2	6
SOAP_2019																							
Rev-1	6	6	5	6	6	5	4	4	4	4	5	5	6	3	5	5	5	4	3	1	1	2	7

Huang L, *et al. BMJ Open* 2024; 14:e084759. doi: 10.1136/bmjopen-2024-084759

Rev-2	6	5	5	6	6	5	3	4	4	4	5	5	6	3	5	6	5	3	3	1	1	2	6
Rev-3	5	5	5	5	5	5	4	4	3	4	5	6	5	3	5	5	5	4	3	1	1	2	7
Rev-4	6	5	5	6	6	5	4	4	4	4	5	5	6	3	5	6	5	4	3	1	1	2	6
AA and OAA_2020																							
Rev-1	5	5	5	4	1	1	2	1	1	1	1	1	1	1	4	4	5	2	1	1	1	6	6
Rev-2	5	5	4	6	1	1	2	1	1	1	1	1	1	1	4	3	4	2	2	1	1	5	6
Rev-3	6	5	4	4	1	1	2	1	1	1	1	1	1	1	4	4	5	2	1	1	1	5	5
Rev-4	5	5	4	5	1	1	2	1	1	1	1	1	1	1	4	3	5	2	2	1	1	6	6
PROSPECT_2020																							
Rev-1	5	6	5	2	1	2	7	7	6	7	7	7	1	1	7	6	7	6	1	1	1	6	6
Rev-2	5	5	5	2	1	2	7	7	7	7	7	7	3	1	6	6	6	6	3	1	1	6	6
Rev-3	5	6	5	2	1	2	7	7	6	6	7	7	1	1	6	6	6	5	1	1	1	5	6
Rev-4	5	5	5	2	1	2	7	7	7	7	6	7	3	1	6	6	6	6	3	1	1	6	5
CHBSA_2020																							
Rev-1	5	5	5	4	3	2	1	1	1	1	4	1	1	1	5	5	5	2	1	1	1	1	1
Rev-2	5	5	5	4	2	2	1	1	1	1	4	1	1	1	5	5	4	2	1	1	1	1	1
Rev-3	5	6	5	4	3	2	1	1	1	1	3	1	1	1	4	5	5	2	1	1	1	1	1
Rev-4	5	5	5	4	2	2	1	1	1	1	3	1	1	1	5	4	4	2	1	1	1	1	1
CAA_2020 (spinal)																							
Rev-1	5	6	6	5	1	7	1	1	1	1	3	1	1	1	6	6	6	1	5	1	1	1	1
Rev-2	6	6	6	5	1	6	1	1	1	1	2	1	1	1	5	5	5	1	4	1	1	1	1
Rev-3	5	6	5	5	1	6	1	1	1	1	2	1	1	1	5	6	5	1	4	1	1	1	1
Rev-4	5	6	6	4	1	6	1	1	1	1	2	1	1	1	5	5	5	1	4	1	1	1	1
CAA_2020 (delivery analgesia)																							
Rev-1	6	6	5	5	2	4	1	1	1	1	3	1	1	1	7	6	6	1	3	1	1	1	1
Rev-2	6	6	6	6	2	5	1	1	1	1	4	1	1	1	7	6	6	1	2	1	1	1	1
Rev-3	5	6	5	5	2	4	1	1	1	1	3	1	1	1	7	6	6	1	2	1	1	1	1

Rev-4	6	6	5	6	2	4	1	1	1	1	4	1	1	1	7	6	6	1	2	1	1	1	1
SOAP_2020																							
Rev-1	5	5	5	6	3	2	6	5	7	6	2	3	1	1	6	6	7	5	3	1	1	1	6
Rev-2	6	6	5	7	4	2	6	6	7	6	4	4	1	1	6	6	6	6	3	1	1	1	6
Rev-3	5	5	5	6	3	2	6	5	6	5	3	4	1	1	5	6	5	6	2	1	1	1	5
Rev-4	5	6	6	7	3	2	6	6	7	6	4	4	1	1	6	5	6	6	3	1	1	1	6
NICE_2021																							
Rev-1	5	7	7	6	6	5	1	1	1	1	6	1	1	7	3	5	3	3	4	1	1	1	1
Rev-2	6	6	7	7	6	6	1	1	1	1	7	1	1	7	5	6	6	5	6	1	1	1	1
Rev-3	6	7	7	6	5	5	1	1	1	1	7	1	1	6	3	4	4	3	4	1	1	1	1
Rev-4	6	6	7	7	6	6	1	1	1	1	7	1	1	7	5	6	6	5	6	1	1	1	1
OAA_2022																							
Rev-1	5	5	4	6	1	4	5	4	1	4	4	1	1	1	3	3	5	5	3	1	1	2	4
Rev-2	5	5	6	6	1	3	6	3	2	3	4	1	1	1	5	5	5	4	2	1	1	4	5
Rev-3	5	5	5	5	1	4	6	4	1	5	3	1	1	1	4	5	5	4	3	1	1	4	4
Rev-4	6	5	6	6	1	3	6	2	2	3	4	1	1	1	5	5	5	4	2	1	1	4	5
ANNA_2023																							
Rev-1	6	6	5	6	3	5	1	1	1	2	1	1	1	3	6	6	5	5	2	1	1	6	6
Rev-2	6	6	6	6	2	6	1	1	1	2	1	1	1	2	7	7	6	5	3	1	1	6	6
Rev-3	5	5	5	5	3	6	1	1	1	2	1	1	1	4	6	6	6	5	2	1	1	6	6
Rev-4	6	5	6	6	2	6	1	1	1	2	1	1	1	2	7	7	6	5	3	1	1	5	6
RCoA_2023																							
Rev-1	7	6	6	7	3	7	6	7	7	7	7	5	5	7	6	6	7	6	6	6	5	7	7
Rev-2	6	6	7	7	4	6	5	7	7	7	7	6	6	6	7	7	7	6	6	6	4	6	6
Rev-3	7	5	6	7	4	6	6	7	7	7	7	6	5	7	7	6	7	6	6	5	5	6	6
Rev-4	6	6	7	7	4	6	5	7	7	7	7	6	6	6	7	7	7	6	5	6	5	6	6
French_2021																							

Huang L, *et al. BMJ Open* 2024; 14:e084759. doi: 10.1136/bmjopen-2024-084759

Rev-1	6	4	6	6	3	4	1	1	1	4	1	1	4	1	5	3	5	1	3	1	1	1	4
Rev-2	6	5	7	7	2	5	1	1	1	5	2	1	5	1	6	4	6	2	2	1	1	1	5
Rev-3	5	5	6	7	3	5	1	1	1	6	1	1	5	1	6	3	6	3	3	1	1	1	5
Rev-4	7	4	7	7	4	6	1	1	1	5	1	1	4	1	7	2	7	1	2	1	1	1	4

Huang L, *et al. BMJ Open* 2024; 14:e084759. doi: 10.1136/bmjopen-2024-084759

Supplementary Table 7. Domain score for each included guidance document.

Document	Domain 1, %	Domain 2, %	Domain 3, %	Domain 4, %	Domain 5, %	Domain 6, %
OG-OG-CMA_2014	62.5	20.8	6.6	20.8	0.0	0.0
OG-A-CMA_2016	66.7	68.1	11.7	61.1	13.5	0.0
ASA_2016	100.0	94.4	91.3	80.6	41.7	79.2
AAGBI_2017	66.7	26.4	18.4	73.6	4.2	25.0
ERAS_2018 (Part 1)	77.8	51.4	69.9	61.1	25.0	47.9
ERAS_2018 (Part 2)	76.4	52.8	69.4	69.4	26.0	52.1
ERAS_2018 (Part 3)	79.2	55.6	69.4	69.4	34.4	37.5
ACOG_2019	83.3	59.7	60.7	72.2	27.1	75.0
SOGC_2019 (Delivery and Postpartum Care)	79.2	70.8	73.5	73.6	19.8	47.9
SOGC_2022 (Twin Pregnancies)	76.4	69.4	76.5	76.4	20.8	47.9
SOAP_2019	72.2	75.0	56.1	69.4	19.8	54.2
AA and OAA_2020	63.9	20.8	4.1	51.4	6.3	77.1
PROSPECT_2020	69.4	11.1	75.5	86.1	24.0	79.2
CHBSA_2020	68.1	30.6	7.1	61.1	4.2	0.0
CAA_2020 (spinal)	77.8	50.0	4.6	72.2	13.5	0.0
CAA_2020 (delivery analgesia)	77.8	48.6	7.1	88.9	5.2	0.0
SOAP_2020	72.2	48.6	53.1	80.6	27.1	39.6
NICE_2021	90.3	81.9	25.5	61.1	29.2	0.0
OAA_2022	69.4	40.3	28.6	59.7	19.8	50.0
ANNA_2023	76.4	61.1	7.7	87.5	22.9	81.3
RCoA_2023	87.5	77.8	90.3	95.8	76.0	87.5
French_2021	82.35	79.66	55.56	80.0	36.0	63.64

Supplementary Table 8. Mean scores across reviewers for the individual AGREE II domain items.

Document	The intraclass correlation coefficient, 95% confidence interval																						
	Domain 1			Domain 2			Domain 3								Domain 4			Domain 5				Domain 6	
	1	2	3	4	5	6	7	8	9	10	11	12	13	14	15	16	17	18	19	20	21	22	23
OG-OG-CMA_2014	3.8	4.7	7.0	2.3	2.0	2.5	1.0	1.0	1.0	1.0	3.3	1.0	1.0	1.0	4.8	1.0	1.0	1.0	1.0	1.0	1.0	1.0	1.0
OG-A-CMA_2016	5.3	5.0	6.0	6.0	6.0	3.3	1.0	1.0	1.0	1.0	5.8	1.0	1.0	1.0	5.3	5.8	3.0	1.0	4.3	1.0	1.0	1.0	1.0
ASA_2016	7.0	7.0	7.0	6.8	6.5	6.8	7.0	6.5	6.0	7.0	6.8	6.0	6.5	6.0	6.0	5.8	5.8	6.0	6.0	1.0	1.0	5.5	6.0
AAGBI_2017	4.8	5.0	5.3	5.8	1.0	1.0	2.3	2.3	2.0	4.3	2.3	41.0	1.0	1.0	5.8	5.3	5.3	1.0	1.0	1.0	2.3	2.3	2.8
ERAS_2018 (Part 1)	5.5	5.8	5.8	5.0	3.8	3.5	6.0	5.8	5.8	5.8	5.8	5.8	3.5	3.0	5.3	4.0	4.8	2.0	5.0	1.0	2.0	2.3	5.5
ERAS_2018 (Part 2)	5.8	5.8	5.3	5.0	3.8	3.8	5.5	6.0	5.0	6.0	5.8	5.8	4.0	3.0	4.8	5.5	5.3	2.0	5.0	1.0	2.3	2.5	5.8
ERAS_2018 (Part 3)	5.8	5.8	5.8	4.8	4.0	4.3	5.8	5.8	5.8	5.8	5.8	5.8	3.5	3.0	5.3	5.0	5.3	3.5	5.3	1.0	2.5	1.0	5.5
ACOG_2019	7.0	5.8	5.3	4.8	4.3	4.8	5.8	5.5	4.8	4.8	4.5	3.5	3.3	4.8	5.8	5.8	4.5	4.3	4.3	1.0	1.0	5.5	5.5
SOGC_2019 (Delivery and Postpartum Care)	5.5	5.3	6.5	5.5	3.3	7.0	7.0	5.3	5.3	5.5	5.5	5.5	3.5	5.5	5.3	5.5	5.5	3.5	3.3	1.0	1.0	2.0	5.8
SOGC_2022 (Twin Pregnancies)	5.5	5.5	5.8	5.5	3.3	6.8	7.0	5.3	5.3	6.0	5.5	5.5	4.5	5.5	5.8	5.5	5.5	3.8	3.3	1.0	1.0	2.0	5.8
SOAP_2019	5.8	5.3	5.0	5.8	5.8	5.0	3.8	4.0	3.8	4.0	5.0	5.3	5.8	3.0	5.0	5.5	5.0	3.8	3.0	1.0	1.0	2.0	6.5
AA and OAA_2020	5.3	5.0	4.3	4.8	1.0	1.0	2.0	1.0	1.0	1.0	1.0	1.0	1.0	1.0	4.0	3.5	4.8	2.0	1.5	1.0	1.0	5.5	5.8
PROSPECT_2020	5.0	5.5	5.0	2.0	1.0	2.0	7.0	7.0	6.5	6.8	6.8	7.0	2.0	1.0	6.3	6.0	6.3	5.8	2.0	1.0	1.0	5.8	5.8
CHBSA_2020	5.0	5.3	5.0	4.0	2.5	2.0	1.0	1.0	1.0	1.0	3.5	1.0	1.0	1.0	4.8	4.8	4.5	2.0	1.0	1.0	1.0	1.0	1.0
CAA_2020 (spinal)	5.3	6.0	5.8	4.8	1.0	6.3	1.0	1.0	1.0	1.0	2.3	1.0	1.0	1.0	5.3	5.5	5.3	1.0	4.3	1.0	1.0	1.0	1.0
CAA_2020 (delivery analgesia)	5.8	6.0	5.3	5.5	2.0	4.3	1.0	1.0	1.0	1.0	3.5	1.0	1.0	1.0	7.0	6.0	6.0	1.0	2.3	1.0	1.0	1.0	1.0
SOAP_2020	5.3	5.5	5.3	6.5	3.3	2.0	6.0	5.5	6.8	5.8	3.3	3.8	1.0	1.0	5.8	5.8	6.0	5.8	2.8	1.0	1.0	1.0	5.8
NICE_2021	5.8	6.5	7.0	6.5	5.8	5.5	1.0	1.0	1.0	1.0	6.8	1.0	1.0	6.8	4.0	5.3	4.8	4.0	5.0	1.0	1.0	1.0	1.0
OAA_2022	5.3	5.0	5.3	5.8	1.0	3.5	5.8	3.3	1.5	3.8	3.8	1.0	1.0	1.0	4.3	4.5	5.0	4.3	2.5	1.0	1.0	3.5	4.5
ANNA_2023	5.8	5.5	5.5	5.8	2.5	5.8	1.0	1.0	1.0	2.0	1.0	1.0	1.0	2.8	6.5	6.5	5.8	5.0	2.5	1.0	1.0	5.8	6.0
RCoA_2023	6.5	5.8	6.5	7.0	3.8	6.3	5.5	7.0	7.0	7.0	7.0	5.8	5.5	6.5	6.8	6.5	7.0	6.0	5.8	5.8	4.8	6.3	6.3

Huang L, *et al. BMJ Open* 2024; 14:e084759. doi: 10.1136/bmjopen-2024-084759

French_2021	6.0	4.5	6.5	6.75	3.0	5.0	1.0	1.0	1.0	5.0	1.25	1.0	4.5	1.0	6.0	3.0	6.0	1.75	2.5	1.0	1.0	1.0	4.5

Supplementary Table 9. The intraclass correlation coefficient between reviewers for each individual AGREE II domain item.

Document	The intraclass correlation coefficient (95% confidence interval)	*P* valve
OG-OG-CMA_2014	0.987 (0.975, 0.994)	<0.001
OG-A-CMA_2016	0.993 (0.986, 0.997)	<0.001
ASA_2016	0.989 (0.980, 0.995)	<0.001
AAGBI_2017	0.990 (0.982, 0.995)	<0.001
ERAS_2018 (Part 1)	0.975 (0.952, 0.988)	<0.001
ERAS_2018 (Part 2)	0.971 (0.945, 0.986)	<0.001
ERAS_2018 (Part 3)	0.967 (0.938, 0.985)	<0.001
ACOG_2019	0.970 (0.942, 0.986)	<0.001
SOGC_2019 (Delivery and Postpartum Care)	0.974 (0.950, 0.988)	<0.001
SOGC_2022 (Twin Pregnancies)	0.976 (0.953, 0.989)	<0.001
SOAP_2019	0.986 (0.974, 0.994)	<0.001
AA and OAA_2020	0.991 (0.983, 0.996)	<0.001
PROSPECT_2020	0.990 (0.981, 0.995)	<0.001
CHBSA_2020	0.994 (0.988, 0.997)	<0.001
CAA_2020 (spinal)	0.995 (0.989, 0.998)	<0.001
CAA_2020 (delivery analgesia)	0.996 (0.993, 0.998)	<0.001
SOAP_2020	0.987 (0.972, 0.994)	<0.001
NICE_2021	0.984 (0.966, 0.993)	<0.001
OAA_2022	0.970 (0.944, 0.986)	<0.001
ANNA_2023	0.991 (0.984, 0.996)	<0.001
RCoA_2023	0.925 (0.859, 0.965)	<0.001
French_2021	0.941 (0.905, 0.986)	<0.001

Huang L, *et al. BMJ Open* 2024; 14:e084759. doi: 10.1136/bmjopen-2024-084759

Supplementary Table 10. Summary of recommendations for the peri-anesthetic evaluation and preparation by included guidance document.

Recommendations	Reference contents	OG-OG-CMA_2014	OG-A-CMA_2016	ASA_2016	AAGBI_2017	ERAS_2018 (Part 1)	ERAS_2018 (Part 2)	ERAS_2018 (Part 3)	ACOG_2019	SOGC_2019	SOGC_2022	SOAP_2019	AA and OAA_2020	PROSPECT_2020	CHBSA_2020	CAA_2020	CAA_2020	SOAP_2020	NICE_2021	OAA_2022	ANNA_2023	RCoA_2023	French_2021
**Peri-anesthetic Evaluation and Preparation**																							
**1. History and physical examination before providing anesthetic care**	Yes	+	+	+	NA	NG	NA	NA	+	NA	NA	NA	NA	NA	+	+	+	NG	+/-	NA	+	NG	+
1.1 Maternal health and anesthetic history	Agree	+	+	+	NA	NG	NA	NA	+	NA	NA	NA	NA	NA	+	+	+	NG	NG	NA	+/-	NG	+
1.2 Relevant obstetric history	Agree	+	+	+	NA	NG	NA	NA	NG	NA	NA	NA	NA	NA	NG	+	+	NG	NG	NA	NG	NG	NG
1.3 Baseline blood pressure measurement	Agree	+	+	+	NA	NG	NA	NA	NG	NA	NA	NA	NA	NA	+	-	+	NG	NG	NA	+/-	NG	NG
1.4 Airway examination	Agree	NG	+	+	NA	NG	NA	NA	+	NA	NA	NA	NA	NA	NG	+	+	NG	NG	NA	+	NG	NG
1.5 Heart and lung examination	Agree	NG	NG	+	NA	NG	NA	NA	+	NA	NA	NA	NA	NA	+	+	+	NG	NG	NA	+	NG	NG

1.6 When a neuraxial anesthetic is planned, examine the patient's back	Agree	NG	+	+	NA	NG	NA	NA	+	NA	NA	NA	NA	NA	NG	+	+	NG	NG	NA	+	NG	NG
1.7 Consultation between the obstetrician and the anesthesiologist	Agree	+	NG	+	NA	+	NA	NA	+	NA	NA	NA	NA	NA	NG	+	+	NG	NG	NA	+	NG	+
**2. Preoperative communication**	Encouraged	+	NG	+	NA	+	NA	NA	NG	NA	NA	NA	NA	NA	+	+	-	+/-	+	NA	+	+/-	+
**3. Intrapartum platelet count**	Yes	+	+	+	NA	NA	NA	NA	+	NA	NA	NA	NA	NA	NG	+	NG	NG	+	NA	NG	NG	NA
-Routine	Agree	+	+	-	NA	NA	NA	NA	+	NA	NA	NA	NA	NA	NG	NG	NG	NG	-	NA	NG	NG	NA
-Individualized	Agree	-	-	+	NA	NA	NA	NA	+	NA	NA	NA	NA	NA	NG	NG	NG	NG	+	NA	NG	NG	NA
**4. Blood type and screen**	Yes	+	+	+	NA	NA	NA	NA	NG	NA	NA	NA	NA	NA	NG	+	NG	NG	+	NA	NG	NG	NA
-Routine	Agree	+	+	-	NA	NA	NA	NA	NG	NA	NA	NA	NA	NA	NG	+	NG	NG	+	NA	NG	NG	NA
-Individualized	Agree	-	-	+	NA	NA	NA	NA	NG	NA	NA	NA	NA	NA	NG	NG	NG	NG	-	NA	NG	NG	NA
**5. Blood cross-match**	Yes	+	NG	+	NA	NA	NA	NA	NG	NA	NA	NA	NA	NA	NG	NG	NG	NG	+	NA	NG	NG	NA
-Routine	Agree	+	NG	-	NA	NA	NA	NA	NG	NA	NA	NA	NA	NA	NG	NG	NG	NG	-	NA	NG	NG	NA
-Individualized	Agree	-	NG	+	NA	NA	NA	NA	NG	NA	NA	NA	NA	NA	NG	NG	NG	NG	+	NA	NG	NG	NA
**6. Peri-anesthetic recording of fetal heart rate**	Yes	+	+	+	NA	NA	NA	NA	+	+	+	NA	NA	NA	NG	+	+	NG	NG	NA	+	NG	+
**7. Aspiration prevention**	Provided	+	NG	+	NA	+	NA	NG	NA	+	NA	NA	NA	NA	+	+/-	NG	+/-	+/-	NA	+/-	+/-	NA
7.1 Clear liquids	Up to 2 h	NG	NG	Up to 2 h	NA	Up to 2 h	NA	NG	NA	NG	NA	NA	NA	NA	Up to 2 h	+	NG	+	NG	NA	NG	+/-	NA
7.2 Solids	6-8 h	+	NG	6-8 h	NA	Light meal	NA	NG	NA	NG	NA	NA	NA	NA	6 h	+	NG	+	NG	NA	NG	NG	NA

						to 6 h																	
7.3 Antacids (surgical procedures-cesarean delivery or postpartum tubal ligation)	Agree	NG	NG	+	NA	+	NA	+	NA	NG	NA	NA	NA	NA	+	+	NG	NG	+	NA	NG	+	NA
7.4 H_2-receptor antagonists (surgical procedures)	Agree	NG	NG	+	NA	+	NA	+	NA	NG	NA	NA	NA	NA	+	+	NG	NG	+	NA	NG	NG	NA
7.5 Metoclopramide (surgical procedures)	Agree	NG	NG	+	NA	NG	NA	20 mg	NA	NG	NA	NA	NA	NA	NG	+	NG	NG	NG	NA	NG	NG	NA
**8. Preoperative heparin or low-molecular-weight heparin**	Yes	NA	+	NG	NA	NA	NA	NA	+	NA	NA	NA	NA	NA	NA	NG	+/-	NG	NG	NA	+/-	+/-	NA
8.1 Prophylactic subcutaneous unfractionated heparin (<5,000 units twice daily)	Provided	NA	NG	NG	NA	NA	NA	NA	+	NA	NA	NA	NA	NA	NA	NG	NG	NG	NG	NA	NG	NG	NA
-Contraindication to neuraxial techniques	No	NA	NG	NG	NA	NA	NA	NA	+	NA	NA	NA	NA	NA	NA	NG	NG	NG	NG	NA	NG	NG	NA
-Catheter placement or removal since last dose	4-6 hours	NA	NG	NG	NA	NA	NA	NA	4-6 h	NA	NA	NA	NA	NA	NA	NG	NG	NG	NG	NA	NG	NG	NA
8.2 Intermediate unfractionated heparin (75,000-100,000 units)	Provided	NA	NG	NG	NA	NA	NA	NA	+	NA	NA	NA	NA	NA	NA	NG	NG	NG	NG	NA	NG	NG	NA
-Catheter placement or removal since last dose	> 12 hours	NA	NG	NG	NA	NA	NA	NA	> 12 hours	NA	NA	NA	NA	NA	NA	NG	NG	NG	NG	NA	NG	NG	NA

Huang L, *et al. BMJ Open* 2024; 14:e084759. doi: 10.1136/bmjopen-2024-084759

8.3 High-dose unfractionated heparin (total daily dose greater than 20,000 units)	Provided	NA	NG	NG	NA	NA	NA	NA	+	NA	NA	NA	NA	NA	NA	NG	NG	NG	NG	NA	NG	NG	NA
-Catheter placement or removal since last dose	> 24 hours	NA	NG	NG	NA	NA	NA	NA	> 24 hours	NA	NA	NA	NA	NA	NA	NG	NG	NG	NG	NA	NG	NG	NA
-With the activated partial thromboplastin time being within normal range	Yes	NA	NG	NG	NA	NA	NA	NA	+	NA	NA	NA	NA	NA	NA	NG	NG	NG	NG	NA	NG	NG	NA

+: consistent the reference with content;
+/-: partial1y consistent the reference with content;
-: inconsistent the reference with content;
NA: not applicable;
NG: not given.

Supplementary Table 11. Summary of recommendations for the anesthetic care for labor and vaginal delivery by included guidance document.

Huang L, *et al. BMJ Open* 2024; 14:e084759. doi: 10.1136/bmjopen-2024-084759

		OG-OG-CMA_2014	OG-A-CMA_2016	ASA_2016	AAGBI_2017	ERAS_2018 (Part 1)	ERAS_2018 (Part 2)	ERAS_2018 (Part 3)	ACOG_2019	SOGC_2019	SOGC_2022	SOAP_2019	AA and OAA_2020	PROSPECT_2020	CHBSA_2020	CAA_2020	CAA_2020	SOAP_2020	NICE_2021	OAA_2022	ANNA_2023	RCoA_2023	French_2021
**Recommendations**	**Reference contents**																						
**Anesthetic Care for Labor and Vaginal Delivery**																							
**1. Equipment, facilities, and support personnel**	Agree	NA	+	+	NA	NA	NA	NA	+	NA	NA	NA	NA	NA	+	NA	+	NA	NA	NA	+	+	NA
**2. Timing of neuraxial analgesia and outcome of labor**	Provided	NA	+	+	NA	NA	NA	NA	NG	NA	NA	NA	NA	NA	NG	NA	+	NA	NA	NA	NG	NG	NA
2.1 Less 5 cm dilation	Agree	NA	+/-	+	NA	NA	NA	NA	NG	NA	NA	NA	NA	NA	NG	NA	+/-	NA	NA	NA	NG	NG	NA
2.2 Offer neuraxial analgesia on an individualized basis	Agree	NA	+	+	NA	NA	NA	NA	NG	NA	NA	NA	NA	NA	NG	NA	+	NA	NA	NA	NG	NG	NA
**3. Neuraxial analgesia and trial of labor after prior cesarean delivery**	Agree	NA	NG	+	NA	NA	NA	NA	NG	NA	NA	NA	NA	NA	NG	NA	NG	NA	NA	NA	NG	NG	NA
**4. Continuous infusion epidural analgesia**	Provided	NA	+	+	NA	NA	NA	NA	+	NA	+/-	NA	NA	NA	+	NA	+	NA	NA	NA	+	NG	NA
4.1 Using local anesthetic	Agree	NA	+	+	NA	NA	NA	NA	+	NA	NG	NA	NA	NA	+	NA	+	NA	NA	NA	+	NG	NA
4.2 Add opioid	Agree	NA	+	+	NA	NA	NA	NA	+	NA	NG	NA	NA	NA	NG	NA	+	NA	NA	NA	+/-	NG	NA
4.3 Platelet concentration corresponding to	Less than $70*10^9$/L	NA	NG	NG	NA	NA	NA	NA	+	NA	NG	NA	NA	NA	NG	NA	NG	NA	NA	NA	+	NG	NA

contraindications to puncture																							
4.4 Puncture location	L_{2-3} or L_{3-4}	NA	+	NG	NA	NA	NA	NA	NG	NA	NG	NA	NA	NA	NG	NA	+	NA	NA	NA	NG	NG	NA
4.5 Analgesic concentrations	Provided	NA	+	NG	NA	NA	NA	NA	NG	NA	NG	NA	NA	NA	NG	NA	+	NA	NA	NA	+	NG	NA
-Bupivacaine	Provided	NA	+	NG	NA	NA	NA	NA	NG	NA	NG	NA	NA	NA	NG	NA	+	NA	NA	NA	+	NG	NA
--Loading dose	0.04-0.125%	NA	+	NG	NA	NA	NA	NA	NG	NA	NG	NA	NA	NA	NG	NA	+	NA	NA	NA	0.0625-0.125%	NG	NA
--Maintenance dose	0.05-0.125%	NA	+	NG	NA	NA	NA	NA	NG	NA	NG	NA	NA	NA	NG	NA	+	NA	NA	NA	0.0625-0.125%	NG	NA
-Ropivacaine	Provided	NA	+	NG	NA	NA	NA	NA	NG	NA	NG	NA	NA	NA	NG	NA	+	NA	NA	NA	+	NG	NA
--Loading dose	0.0625-0.15%	NA	+	NG	NA	NA	NA	NA	NG	NA	NG	NA	NA	NA	NG	NA	+	NA	NA	NA	0.08-0.2%	NG	NA
--Maintenance dose	0.0625-0.125%	NA	+	NG	NA	NA	NA	NA	NG	NA	NG	NA	NA	NA	NG	NA	+	NA	NA	NA	0.08-0.2%	NG	NA
-Levobupivacaine	Provided	NA	NG	NG	NA	NA	NA	NA	NG	NA	NG	NA	NA	NA	NG	NA	+	NA	NA	NA	NG	NG	NA
--Loading dose	0.04-0.125%	NA	NG	NG	NA	NA	NA	NA	NG	NA	NG	NA	NA	NA	NG	NA	+	NA	NA	NA	NG	NG	NA
--Maintenance dose	0.05-0.125%	NA	NG	NG	NA	NA	NA	NA	NG	NA	NG	NA	NA	NA	NG	NA	+	NA	NA	NA	NG	NG	NA
4.6 Opioids concentrations	Provided	NA	+	NG	NA	NA	NA	NA	NG	NA	NG	NA	NA	NA	NG	NA	+	NA	NA	NA	+/-	NG	NA
-Fentanyl	Provided	NA	+	NG	NA	NA	NA	NA	NG	NA	NG	NA	NA	NA	NG	NA	+	NA	NA	NA	NG	NG	NA
--Loading dose	0.5-2 $\mu g.ml^{-1}$	NA	1-2	NG	NA	NA	NA	NA	NG	NA	NG	NA	NA	NA	NG	NA	+	NA	NA	NA	NG	NG	NA
--Maintenance dose	1-2 $\mu g.ml^{-1}$	NA	+	NG	NA	NA	NA	NA	NG	NA	NG	NA	NA	NA	NG	NA	+	NA	NA	NA	NG	NG	NA
-Sufentanyl	Provided	NA	+	NG	NA	NA	NA	NA	NG	NA	NG	NA	NA	NA	NG	NA	+	NA	NA	NA	NG	NG	NA

Huang L, *et al. BMJ Open* 2024; 14:e084759. doi: 10.1136/bmjopen-2024-084759

--Loading dose	0.2-0.6 μg.ml^{-1}	NA	0.4-0.6	NG	NA	NA	NA	NA	NG	NA	NG	NA	NA	NA	NG	NA	+	NA	NA	NA	NG	NG	NA
--Maintenance dose	0.3-0.6 μg.ml^{-1}	NA	0.4-0.6	NG	NA	NA	NA	NA	NG	NA	NG	NA	NA	NA	NG	NA	+	NA	NA	NA	NG	NG	NA
**5. Combined spinal–epidural analgesia**	Provided	NA	+	+	NA	NA	NA	NA	+	NA	NA	NA	NA	NA	NG	NA	+	NA	NA	NA	+/-	NG	NA
5.1 Using local anesthetic	Agree	NA	+	NG	NA	NA	NA	NA	+	NA	NA	NA	NA	NA	NG	NA	+	NA	NA	NA	+	NG	NA
5.2 Add opioid	Agree	NA	+	NG	NA	NA	NA	NA	+	NA	NA	NA	NA	NA	NG	NA	+	NA	NA	NA	+	NG	NA
5.3 Platelet concentration corresponding to contraindications to puncture	Less than 70*10^9/L	NA	NG	NG	NA	NA	NA	NA	+	NA	NA	NA	NA	NA	NG	NA	+	NA	NA	NA	+	NG	NA
5.4 Puncture location	L_{2-3} or L_{3-4}	NA	+	NG	NA	NA	NA	NA	NG	NA	NA	NA	NA	NA	NG	NA	+/-	NA	NA	NA	NG	NG	NA
5.5 Epidural anesthetics (consistent with 4.5 and 4.6)	Provided	NA	+	NG	NA	NA	NA	NA	NG	NA	NA	NA	NA	NA	NG	NA	+	NA	NA	NA	+/-	NG	NA
5.6 Spinal anesthetics	Provided	NA	+	NG	NA	NA	NA	NA	+	NA	NA	NA	NA	NA	NG	NA	+	NA	NA	NA	NG	NG	NA
-Protocol 1	Provided	NA	+	NG	NA	NA	NA	NA	+	NA	NA	NA	NA	NA	NG	NA	+	NA	NA	NA	NG	NG	NA
--Single sufentanyl	2.5-7 μg	NA	+	NG	NA	NA	NA	NA	+/-	NA	NA	NA	NA	NA	NG	NA	+	NA	NA	NA	NG	NG	NA
--Single ropivacaine	2.3-3 mg	NA	2.5-3	NG	NA	NA	NA	NA	+/-	NA	NA	NA	NA	NA	NG	NA	+	NA	NA	NA	NG	NG	NA
--Combined sufentanyl and ropivacaine	2.5 μg / 2.5 mg	NA	+	NG	NA	NA	NA	NA	+/-	NA	NA	NA	NA	NA	NG	NA	+	NA	NA	NA	Sufentanyl 5-10 μg	NG	NA
-Protocol 2	Provided	NA	+	NG	NA	NA	NA	NA	+/-	NA	NA	NA	NA	NA	NG	NA	+	NA	NA	NA	NG	NG	NA
--Single fentanyl	15-25 μg	NA	+	NG	NA	NA	NA	NA	+/-	NA	NA	NA	NA	NA	NG	NA	+	NA	NA	NA	NG	NG	NA
--Single bupivacaine	2.0-2.5 mg	NA	+	NG	NA	NA	NA	NA	+/-	NA	NA	NA	NA	NA	NG	NA	+	NA	NA	NA	NG	NG	NA
--Combined fentanyl and bupivacaine	2.5 μg / 2.0 mg	NA	+	NG	NA	NA	NA	NA	+/-	NA	NA	NA	NA	NA	NG	NA	+	NA	NA	NA	Fentanyl 50-100 μg	NG	NA

BMJ Publishing Group Limited (BMJ) disclaims all liability and responsibility arising from any reliance placed on this supplemental material which has been supplied by the author(s)

**6. Single-injection spinal**	Provided	NA	NG	+	NA	NA	NA	NA	+	NA	NA	NA	NA	NA	NG	NA	+	NA	NA	NA	+	NG	NA
6.1 Using local anesthetic	Agree	NA	NG	+	NA	NA	NA	NA	+	NA	NA	NA	NA	NA	NG	NA	+	NA	NA	NA	NG	NG	NA
6.2 Add opioid	Agree	NA	NG	+	NA	NA	NA	NA	+	NA	NA	NA	NA	NA	NG	NA	+	NA	NA	NA	NG	NG	NA
6.3 Platelet concentration corresponding to contraindications to puncture	Less than $70*10^9$/L	NA	NG	NG	NA	NA	NA	NA	+	NA	NA	NA	NA	NA	NG	NA	NG	NA	NA	NA	+	NG	NA
6.4 Puncture location	L_{3-4}	NA	NG	NG	NA	NA	NA	NA	NG	NA	NA	NA	NA	NA	NG	NA	NG	NA	NA	NA	NG	NG	NA
6.6 Analgesic concentrations (consistent with 5.6)	Agree	NA	NG	NG	NA	NA	NA	NA	NG	NA	NA	NA	NA	NA	NG	NA	+	NA	NA	NA	NG	NG	NA
6.7 Spinal needles	Provided	NA	NG	+	NA	NA	NA	NA	NG	NA	NA	NA	NA	NA	NG	NA	NG	NA	NA	NA	+	NG	NA
-Cutting-bevel	Disagree	NA	NG	+	NA	NA	NA	NA	NG	NA	NA	NA	NA	NA	NG	NA	NG	NA	NA	NA	+	NG	NA
-Pencil-point	Agree	NA	NG	+	NA	NA	NA	NA	NG	NA	NA	NA	NA	NA	NG	NA	NG	NA	NA	NA	+	NG	NA
**7. Patient-controlled epidural analgesia (for labor analgesia)**	Agree	NA	+	+	NA	NA	NA	NA	NG	NA	NA	NA	NA	NA	+	NA	+	NA	NA	NA	+	NG	NA

+: consistent the reference with content;

+/-: partial1y consistent the reference with content;

-: inconsistent the reference with content;

NA: not applicable;

NG: not given.

Supplementary Table 12. Summary of recommendations for the anesthetic care for cesarean delivery by included guidance document.

Huang L, *et al. BMJ Open* 2024; 14:e084759. doi: 10.1136/bmjopen-2024-084759

		OG-OG-CMA_2014	OG-A-CMA_2016	ASA_2016	AAGBI_2017	ERAS_2018 (Part 1)	ERAS_2018 (Part 2)	ERAS_2018 (Part 3)	ACOG_2019	SOGC_2019	SOGC_2022	SOAP_2019	AA and OAA_2020	PROSPECT_2020	CHBSA_2020	CAA_2020	CAA_2020	SOAP_2020	NICE_2021	OAA_2022	ANNA_2023	RCoA_2023	French_2021
**Recommendations**	**Reference contents**																						
**Anesthetic Care for Cesarean Delivery**																							
**1. Equipment, facilities, and support personnel**	Agree	+	NA	+	NA	NA	NG	NA	+	+	NA	NA	NA	NA	+	NG	NA	NA	NA	NA	+	+	NA
**2. Continuous infusion epidural analgesia**	Provided	+	NA	+	NA	NA	+/-	NA	+	+/-	NA	NA	NA	NA	+	+	NA	NA	NA	NA	+	NG	NA
2.1 Using local anesthetic	Agree	NG	NA	NG	NA	NA	NG	NA	+	NG	NA	NA	NA	NA	+	+	NA	NA	NA	NA	+	NG	NA
2.2 Add opioid	Agree	NG	NA	NG	NA	NA	NG	NA	+	NG	NA	NA	NA	NA	NG	+	NA	NA	NA	NA	+	NG	NA
2.3 Platelet concentration corresponding to contraindications to puncture	Less than $70*10^9$/L	NG	NA	NG	NA	NA	NG	NA	+	NG	NA	NA	NA	NA	NG	+	NA	NA	NA	NA	+	NG	NA
2.4 Puncture location	L_{1-2} or L_{2-3}	NG	NA	NG	NA	NA	NG	NA	NG	NG	NA	NA	NA	NA	NG	+	NA	NA	NA	NA	NG	NG	NA
2.5 Recommend level of block	T_{6-4}	NG	NA	NG	NA	NA	NG	NA	T4	NG	NA	NA	NA	NA	NG	+	NA	NA	NA	NA	NG	NG	NA
2.6 Analgesic concentrations	Provided	NG	NA	NG	NA	NA	NG	NA	NG	NG	NA	NA	NA	NA	NG	+	NA	NA	NA	NA	+/-	NG	NA
-Lidocaine	1.5%-2%	NG	NA	NG	NA	NA	NG	NA	NG	NG	NA	NA	NA	NA	NG	+	NA	NA	NA	NA	NG	NG	NA
-Bupivacaine	0.50%	NG	NA	NG	NA	NA	NG	NA	NG	NG	NA	NA	NA	NA	NG	+	NA	NA	NA	NA	0.0625-0.125%	NG	NA

Huang L, *et al. BMJ Open* 2024; 14:e084759. doi: 10.1136/bmjopen-2024-084759

-Ropivacaine	0.5%-0.75%	NG	NA	NG	NA	NA	NG	NA	NG	NG	NA	NA	NA	NA	NG	+	NA	NA	NA	NA	0.08-0.2%	NG	NA
-Levobupivacaine	0.5%-0.75%	NG	NA	NG	NA	NA	NG	NA	NG	NG	NA	NA	NA	NA	NG	+	NA	NA	NA	NA	NG	NG	NA
-Chloroprocaine	3%	NG	NA	NG	NA	NA	NG	NA	NG	NG	NA	NA	NA	NA	NG	+	NA	NA	NA	NA	NG	NG	NA
2.7 Prevention of local anesthetic poisoning	Provided	NG	NA	NG	NA	NA	NG	NA	+	NG	NA	NA	NA	NA	NG	+	NA	NA	NA	NA	NA	NG	NA
-Withdrawal before injection	Agree	NG	NA	NG	NA	NA	NG	NA	NG	NG	NA	NA	NA	NA	NG	+	NA	NA	NA	NA	NA	NG	NA
-Administration of test dose	Agree	NG	NA	NG	NA	NA	NG	NA	NG	NG	NA	NA	NA	NA	NG	+	NA	NA	NA	NA	NA	NG	NA
-Addition of epinephrine	Agree	NG	NA	NG	NA	NA	NG	NA	+	NG	NA	NA	NA	NA	NG	+	NA	NA	NA	NA	NA	NG	NA
**3. Single-injection spinal**	Provided	NA	NA	+	+/-	NA	+/-	NA	+	+/-	NA	NA	NA	NA	+	+	NA	NA	NA	NA	+	NG	+
3.1 Using local anesthetic	Agree	NA	NA	NG	NG	NA	NG	NA	+	NG	NA	NA	NA	NA	+	+	NA	NA	NA	NA	+	NG	+
3.2 Add opioid	Agree	NA	NA	NG	NG	NA	NG	NA	+	NG	NA	NA	NA	NA	NG	+	NA	NA	NA	NA	+	NG	+
3.3 Platelet concentration corresponding to contraindications to puncture	Less than $70*10^9$/L	NA	NA	NG	NG	NA	NG	NA	+	NG	NA	NA	NA	NA	NG	+	NA	NA	NA	NA	+	NG	NG
3.4 Puncture location	L_{3-4}	NA	NA	NG	NG	NA	NG	NA	NG	NG	NA	NA	NA	NA	NG	+	NA	NA	NA	NA	NG	NG	NG
3.5 Recommend level of block	T_{6-4}	NA	NA	NG	NG	NA	NG	NA	NG	NG	NA	NA	NA	NA	NG	+	NA	NA	NA	NA	NG	NG	NG
3.6 Analgesic dose	Provided	NA	NA	NG	NG	NA	NG	NA	NG	NG	NA	NA	NA	NA	NG	+	NA	NA	NA	NA	NG	NG	+
-Bupivacaine	5-15 mg	NA	NA	NG	NG	NA	NG	NA	NG	NG	NA	NA	NA	NA	NG	+	NA	NA	NA	NA	NG	NG	+
-Ropivacaine	10-20 mg	NA	NA	NG	NG	NA	NG	NA	NG	NG	NA	NA	NA	NA	NG	+	NA	NA	NA	NA	NG	NG	NG
3.7 Spinal needles	Provided	NA	NA	+	NG	NA	NG	NA	NG	NG	NA	NA	NA	NA	NG	+	NA	NA	NA	NA	+	NG	NG
-Cutting-bevel	Disagree	NA	NA	+	NG	NA	NG	NA	NG	NG	NA	NA	NA	NA	NG	+	NA	NA	NA	NA	+	NG	NG
-Pencil-point	Agree	NA	NA	+	NG	NA	NG	NA	NG	NG	NA	NA	NA	NA	NG	+	NA	NA	NA	NA	+	NG	NG

Huang L, *et al. BMJ Open* 2024; 14:e084759. doi: 10.1136/bmjopen-2024-084759

**4. Combined spinal–epidural analgesia**	Provided	+	NA	+	+	NA	+/-	NA	+	+	NA	NA	NA	NA	+	+	NA	NA	NA	NA	+/-	NG	+
4.1 Puncture location	Provided	NG	NA	NG	NG	NA	NG	NA	NG	NG	NA	NA	NA	NA	NG	+	NA	NA	NA	NA	+/-	NG	NG
-Single space technique	L_{3-4}	NG	NA	NG	NG	NA	NG	NA	NG	NG	NA	NA	NA	NA	NG	+	NA	NA	NA	NA	+/-	NG	NG
-Double space technique	T_{12}-L_2/L_{3-5}	NG	NA	NG	NG	NA	NG	NA	NG	NG	NA	NA	NA	NA	NG	+	NA	NA	NA	NA	+/-	NG	NG
4.2 Anesthetics (consistent with spinal or epidural)	Agree	NG	NA	NG	NG	NA	NG	NA	+	NG	NA	NA	NA	NA	NG	+	NA	NA	NA	NA	+/-	NG	+/-
**5. General anesthesia**	Provided	+	NA	+	NA	NA	NA	NA	+	NA	NA	NA	NA	NA	+	+	NA	NA	+	NA	+	+/-	NA
5.1 General anesthesia selected condition	Provided	NG	NA	+	NA	NA	NA	NA	+	NA	NA	NA	NA	NA	NG	NG	NA	NA	NG	NA	+	NG	NA
-Profound fetal bradycardia	Agree	NG	NA	+	NA	NA	NA	NA	NG	NA	NA	NA	NA	NA	NG	NG	NA	NA	NG	NA	NG	NG	NA
-Ruptured uterus	Agree	NG	NA	+	NA	NA	NA	NA	NG	NA	NA	NA	NA	NA	NG	NG	NA	NA	NG	NA	NG	NG	NA
-Severe Hemorrhage	Agree	NG	NA	+	NA	NA	NA	NA	NG	NA	NA	NA	NA	NA	NG	NG	NA	NA	NG	NA	NG	NG	NA
-Severe placental abruption	Agree	NG	NA	+	NA	NA	NA	NA	NG	NA	NA	NA	NA	NA	NG	NG	NA	NA	NG	NA	NG	NG	NA
-Umbilical cord prolapse	Agree	NG	NA	+	NA	NA	NA	NA	NG	NA	NA	NA	NA	NA	NG	NG	NA	NA	NG	NA	NG	NG	NA
-Preterm footling breech	Agree	NG	NA	+	NA	NA	NA	NA	NG	NA	NA	NA	NA	NA	NG	NG	NA	NA	NG	NA	NG	NG	NA
-Inability to place neuraxial anesthesia	Agree	NG	NA	NG	NA	NA	NA	NA	+	NA	NA	NA	NA	NA	NG	NG	NA	NA	NG	NA	+	NG	NA
5.2 Intravenous anesthetics recommendation	Provided	NG	NA	NG	NA	NA	NA	NA	+	NA	NA	NA	NA	NA	NG	+	NA	NA	NG	NA	NG	NG	NA

-Thiopental sodium	4-5 mg.kg^{-1}	NG	NA	NG	NA	NA	NA	NA	NG	NA	NA	NA	NA	NA	NG	+	NA	NA	NG	NA	NG	NG	NA
-Propofol	1.5-2.5 mg.kg^{-1}	NG	NA	NG	NA	NA	NA	NA	+	NA	NA	NA	NA	NA	NG	+	NA	NA	NG	NA	NG	NG	NA
-Etomidate	0.2-0.3 mg.kg^{-1}	NG	NA	NG	NA	NA	NA	NA	NG	NA	NA	NA	NA	NA	NG	+	NA	NA	NG	NA	NG	NG	NA
-Ketamine	0.5~1 mg.kg^{-1}	NG	NA	NG	NA	NA	NA	NA	+	NA	NA	NA	NA	NA	NG	+	NA	NA	NG	NA	NG	NG	NA
5.3 Opioids analgesic recommendation	Provided	NG	NA	NG	NA	NA	NA	NA	NG	NA	NA	NA	NA	NA	NG	+	NA	NA	NG	NA	NG	NG	NA
-Fentanyl	2-5 μg.kg^{-1}	NG	NA	NG	NA	NA	NA	NA	NG	NA	NA	NA	NA	NA	NG	+	NA	NA	NG	NA	NG	NG	NA
-Sufentanyl	0.2~0.5 μg.kg^{-1}	NG	NA	NG	NA	NA	NA	NA	NG	NA	NA	NA	NA	NA	NG	+	NA	NA	NG	NA	NG	NG	NA
-Remifentanyl	0.5-1 μg.kg^{-1} for IV	NG	NA	NG	NA	NA	NA	NA	NG	NA	NA	NA	NA	NA	NG	+	NA	NG	NG	NA	NG	NG	NA
-Remifentanyl	4 ng.kg^{-1} for target	NG	NA	NG	NA	NA	NA	NA	NG	NA	NA	NA	NA	NA	NG	+	NA	NG	NG	NA	NG	NG	NA
5.4 Muscle relaxants recommendation	Provided	NG	NA	NG	NA	NA	NA	NA	+	NA	NA	NA	NA	NA	NG	+	NA	NG	NG	NA	NG	NG	NA
-Succinylcholine	1-1.5 mg.kg^{-1}	NG	NA	NG	NA	NA	NA	NA	NG	NA	NA	NA	NA	NA	NG	+	NA	NG	NG	NA	NG	NG	NA
-Rocuronium	0.6-1.2 mg.kg^{-1}	NG	NA	NG	NA	NA	NA	NA	NG	NA	NA	NA	NA	NA	NG	+	NA	NG	NG	NA	NG	NG	NA
5.5 Induction-delivery interval	within 10 min	NG	NA	NG	NA	NA	NA	NA	NG	NA	NA	NA	NA	NA	NG	+	NA	NG	NG	NA	NG	NG	NA

+: consistent the reference with content;

+/-: partial1y consistent the reference with content;

-: inconsistent the reference with content;

NA: not applicable;

NG: not given.

Supplementary Table 13. Summary of recommendations for the monitoring and management of obstetric and anesthetic or intra- or postoperative pain management by included guidance document.

Recommendations	Reference contents	OG-OG-CMA_2014	OG-A-CMA_2016	ASA_2016	AAGBI_2017	ERAS_2018 (Part 1)	ERAS_2018 (Part 2)	ERAS_2018 (Part 3)	ACOG_2019	SOGC_2019	SOGC_2022	SOAP_2019	AA and OAA_2020	PROSPECT_2020	CHBSA_2020	CAA_2020	CAA_2020	SOAP_2020	NICE_2021	OAA_2022	ANNA_2023	RCoA_2023	French_2021
**Neurological Monitoring Associated with Obstetric Neuraxial Block**																							
**1. Screening method (During the recovery phase) - Straight-leg raising**	Agree	NA	NA	NA	NA	NA	NA	NA	NA	NA	NA	NA	+	NA	NA	NA	NA	NA	NA	NA	NA	NA	NA
**2. Alerts incidences-unable to straight-leg raise at 4 h from the last dose**	Agree	NA	NA	NA	NA	NA	NA	NA	NA	NA	NA	NA	+	NA	NA	NA	NA	NA	NA	NA	NA	NA	NA

**Respiratory Depression Monitoring with Neuraxial Block With Opioids**																							
**1. Frequency and modality of respiratory monitoring should be based on:**	Provided	+	NA	NA	NA	NA	NA	NA	NA	NA	NA	+	NA	NA	NA	NA	NA	NA	+/-	NA	+/-	NA	NA
1.1 Low risk	Provided	NG	NA	NA	NA	NA	NA	NA	NA	NA	NA	+	NA	NA	NA	NA	NA	NA	+/-	NA	NG	NA	NA
-Neuraxial morphine dose (intrathecal ≤ 0.15 mg or epidural ≤ 3 mg)	Agree	NG	NA	NA	NA	NA	NA	NA	NA	NA	NA	+	NA	NA	NA	NA	NA	NA	NG	NA	NG	NA	NA
1.2 High risk	Provided	NG	NA	NA	NA	NA	NA	NA	NA	NA	NA	+	NA	NA	NA	NA	NA	NA	+/-	NA	NG	NA	NA
Cardiopulmonary/ neurological comorbidity	Agree	NG	NA	NA	NA	NA	NA	NA	NA	NA	NA	+	NA	NA	NA	NA	NA	NA	NG	NA	NG	NA	NA
-Obesity (body mass index ≥ 40 kg/m^2)	Agree	NG	NA	NA	NA	NA	NA	NA	NA	NA	NA	+	NA	NA	NA	NA	NA	NA	NG	NA	NG	NA	NA
-Known or suspected obstructive sleep apnea	Agree	NG	NA	NA	NA	NA	NA	NA	NA	NA	NA	+	NA	NA	NA	NA	NA	NA	NG	NA	NG	NA	NA

-Chronic opioid use or abuse	Agree	NG	NA	NA	NA	NA	NA	NA	NA	NA	NA	+	NA	NA	NA	NA	NA	NA	NG	NA	NG	NA	NA
-Hypertension	Agree	NG	NA	NA	NA	NA	NA	NA	NA	NA	NA	+	NA	NA	NA	NA	NA	NA	NG	NA	NG	NA	NA
-Magnesium administration	Agree	NG	NA	NA	NA	NA	NA	NA	NA	NA	NA	+	NA	NA	NA	NA	NA	NA	NG	NA	NG	NA	NA
-Neuraxial morphine dose (intrathecal > 0.15 mg or epidural > 3 mg)	Agree	NG	NA	NA	NA	NA	NA	NA	NA	NA	NA	+	NA	NA	NA	NA	NA	NA	NG	NA	NG	NA	NA
1.3 Respiratory monitoring frequency and duration	Provided	NG	NA	NA	NA	NA	NA	NA	NA	NA	NA	+	NA	NA	NA	NA	NA	NA	+/-	NA	NG	NA	NA
-Low risk	Provided	NG	NA	NA	NA	NA	NA	NA	NA	NA	NA	+	NA	NA	NA	NA	NA	NA	+/-	NA	NG	NA	NA
--Routine postoperative vital sign monitoring	Agree	NG	NA	NA	NA	NA	NA	NA	NA	NA	NA	+	NA	NA	NA	NA	NA	NA	+	NA	NG	NA	NA
-- q2h clinical assessment for 12 h	Agree	+/-	NA	NA	NA	NA	NA	NA	NA	NA	NA	+	NA	NA	NA	NA	NA	NA	+	NA	NG	NA	NA
-High risk	Provided	NG	NA	NA	NA	NA	NA	NA	NA		NA	+		NA	NA	NA	NA	NA	+	NA	NG	NA	NA
-- q1h clinical assessment for first 12 h	Agree	NG	NA	NA	NA	NA	NA	NA	NA	NA	NA	+	NA	NA	NA	NA	NA	NA	half hour for 2 hours	NA	NG	NA	NA
-- q2h clinical assessment for first 12-24 h	Agree	+/-	NA	NA	NA	NA	NA	NA	NA	NA	NA	+	NA	NA	NA	NA	NA	NA	NG	NA	NG	NA	NA

Huang L, *et al. BMJ Open* 2024; 14:e084759. doi: 10.1136/bmjopen-2024-084759

--Consider additional monitoring modalities (e.g., pulse oximetry, capnography)	Agree	NG	NA	NA	NA	NA	NA	NA	NA	NA	NA	+	NA	NA	NA	NA	NA	NA	+/-	NA	NG	NA	NA
**Management of Intra- or Postoperative Pain**																							
**1. Implement strategies to minimize systemic opioid utilization**	Provided	NA	NA	NA	NA	NA	NA	+	+	NA	NA	+	NA	+	+	NA	NA	+	+	+	+/-	NA	NA
**2. Neuraxial adjuvant drugs**	Provided	NA	NA	NA	NA	NA	NA	+	+	NA	NA	+	NA	+	+	NA	NA	+	+	NG	+	NA	NA
-Spinal anesthesia	Provided	NA	NA	NA	NA	NA	NA	NG	+	NA	NA	+	NA	+	+	NA	NA	+	+	NG	+	NA	NA
--Morphine	50-150 μg	NA	NA	NA	NA	NA	NA	NG	+	NA	NA	50-150 μg	NA	+	+	NA	NA	50-150 μg	NG	NG	150-500μg	NA	NA
-Hydromorphone	75-150 μg	NA	NA	NA	NA	NA	NA	NG	NG	NA	NA	NG	NA	NG	NG	NA	NA	NG	NG	NG	+	NA	NA
-Epidural or combined spinal-epidural technique.	Provided	NA	NA	NA	NA	NA	NA	NG	+	NA	NA	+	NA	+	NG	NA	NA	+	+	NG	+	NA	NA
--Morphine	1-3 mg	NA	NA	NA	NA	NA	NA	NG	+	NA	NA	1-3 mg	NA	+	NG	NA	NA	1~3 mg	NG	NG	1-3mg	NA	NA

--Diamorphine	2-3 mg	NA	NA	NA	NA	NA	NA	NG	NG	NA	NA	NG	NA	+	NG	NA	NA	NG	2.5-5 mg	NG	NG	NA	NA
**3. Others analgesic (after delivery)**	Provided	NA	NA	NA	NA	NA	NA	+	+	NA	NA	+	NA	+	+	NA	NA	+	+	NG	+	NA	NA
-Non-steroidal anti-inflammatory drug (e.g., paracetamol)	Agree	NA	NA	NA	NA	NA	NA	+	+	NA	NA	+	NA	+	+	NA	NA	+	+	NG	+	NA	NA
-A single dose of intravenous dexamethasone	Agree	NA	NA	NA	NA	NA	NA	NG	NG	NA	NA	NG	NA	+	NG	NA	NA	NG	NG	NG	+	NA	NA
**4. Local anesthetic infiltration**	Agree	NA	NA	NA	NA	NA	NA	+	+	NA	NA	NG	NA	+	+	NA	NA	+	NG	NG	+	NA	NA
**5. Continuous wound local anesthetic infusion**	Agree	NA	NA	NA	NA	NA	NA	NG	+	NA	NA	NG	NA	+	+	NA	NA	NG	NG	NG	NG	NA	NA
**6. Fascial plane blocks (e.g., transversus abdominis plane, erector spinae plane)**	Agree	NA	NA	NA	NA	NA	NA	+	+	NA	NA	NG	NA	+	+	NA	NA	+	NG	NG	NG	NA	NA
**7. Patient-controlled intravenous analgesia**	Agree	NA	NA	NA	NA	NA	NA	NG	+	NA	NA	NG	NA	+	+	NA	NA	NG	+	NG	NG	NA	NA

+: consistent the reference with content;

+/-: partial1y consistent the reference with content;

-: inconsistent the reference with content;
NA: not applicable;
NG: not given.

Supplementary Table 14. Summary of recommendations for the adverse events by included guidance document.

Recommendations	Reference contents	OG-OG-CMA_2014	OG-A-CMA_2016	ASA_2016	AAGBI_2017	ERAS_2018 (Part 1)	ERAS_2018 (Part 2)	ERAS_2018 (Part 3)	ACOG_2019	SOGC_2019	SOGC_2022	SOAP_2019	AA and OAA_2020	PROSPECT_2020	CHBSA_2020	CAA_2020	CAA_2020	SOAP_2020	NICE_2021	OAA_2022	ANNA_2023	RCoA_2023	French_2021
**1. Hypotension prevention strategies**	Provided	NA	NA	+	+	NA	+/-	+	+	+	NA	NA	NA	NA	+	+	+	+	+	NA	+/-	NA	NA
1.1 Blood pressure target of systolic arterial pressure	≥90% of baseline and avoid decrease to < 80% baseline	NA	NA	NG	+	NA	NG	NG	NG	NG	NA	NA	NA	NA	NG	NG	NG	NG	+	NA	NG	NA	NA
1.2 Position (uterine left-leaning)	Yes	NA	NA	+	+	NA	NG	NG	NG	NG	NA	NA	NA	NA	+	+	+	NG	+	NA	+	NA	NA
1.3 Volume expansion (crystals or colloids)	500-1000 ml	NA	NA	+	+	NA	+	+	+	+	NA	NA	NA	NA	+	+	+	NG	+	NA	+	NA	NA
1.4 Vasoactive drugs	Yes	NA	NA	+	+	NA	NG	+	+	NG	NA	NA	NA	NA	+	+	+	+	+	NA	+	NA	NA
-Pump infusion is recommended	Provided	NA	NA	+	+	NA	NG	NG	NG	NG	NA	NA	NA	NA	NG	NG	NG	NG	NG	NA	NG	NA	NA
-Type of drugs	Provided	NA	NA	+	+	NA	NG	+	+	NG	NA	NA	NA	NA	+	+	+	+	NG	NA	+/-	NA	NA
--Phenylephrine	20-50 μg Or	NA	NA	+	20~50 μg or	NA	NG	+	+	NG	NA	NA	NA	NA	+	+	+	+	NG	NA	+	NA	NA

	0.5 μg . kg^{-1}. min^{-1}				0.25. kg^{-1}. min^{-1}																		
--Metaraminol	1-2 mg or 4 $μg.kg^{-1}$. min^{-1}	NA	NA	NG	0.5$mg.ml^{-1}$	NA	NG	NG	NG	NG	NA	NA	NA	NA	NG	NG	NG	NG	NG	NA	NG	NA	NA
--Noradrenalin	4-6 μg or 0.08 μg kg^{-1}. min^{-1}	NA	NA	NG	NG	NA	NG	NG	NG	NG	NA	NA	NA	NA	NG	+	NG	NG	NG	NA	NG	NA	NA
--Ephedrine	2-15 mg for IV	NA	NA	NG	2-4mg	NA	NG	+	+	NG	NA	NA	NA	NA	+	+	+	NG	NG	NA	NG	NA	NA
1.5 Prophylactic vasopressor infusion should not be recommended in pre-eclampsia	Agree	NA	NA	NG	+	NA	NG	NG	NG	NG	NA	NA	NA	NA	NG	+	NG	NG	NG	NA	NG	NA	NA
**2. Inadequate neuraxial anesthesia**	Provided	NA	NA	NA	NA	NA	NA	NA	+	NA	NA	NA	NA	NA	NA	NA	+	NA	NA	+	+	+	+
2.1 Block assessment	Provided	NA	NA	NA	NA	NA	NA	NA	NG	NA	NA	NA	NA	NA	NA	NA	NG	NA	NA	+	+	NG	+
-Testing sensory block after the evidence of motor block	Agree	NA	NA	NA	NA	NA	NA	NA	NG	NA	NA	NA	NA	NA	NA	NA	NG	NA	NA	+	+	NG	NG
-Check the block at relatively fixed time intervals	Agree	NA	NA	NA	NA	NA	NA	NA	NG	NA	NA	NA	NA	NA	NA	NA	NG	NA	NA	+	NG	NG	NG
-Perform the first check early enough to allow positional changes	Agree	NA	NA	NA	NA	NA	NA	NA	NG	NA	NA	NA	NA	NA	NA	NA	NG	NA	NA	+	NG	NG	NG

2.2 Rescue for inadequate neuraxial block (change anesthesia method)	Provided	NA	NA	NA	NA	NA	NA	NA	+	NA	NA	NA	NA	NA	NA	NA	NG	NA	NA	+	+	NG	+
-A second neuraxial technique (in the case of spinal anesthesia)	Agree	NA	NA	NA	NA	NA	NA	NA	+	NA	NA	NA	NA	NA	NA	NA	+	NA	NA	+	+	NG	+
-Extending the neuraxial technique (in the case of combined spinal–epidural)	Agree	NA	NA	NA	NA	NA	NA	NA	+	NA	NA	NA	NA	NA	NA	NA	NG	NA	NA	+	+	NG	NG
-Above options are not possible, general anesthesia should be recommended	Agree	NA	NA	NA	NA	NA	NA	NA	+	NA	NA	NA	NA	NA	NA	NA	NG	NA	NA	+	+	NG	+
2.3 Rescue for inadequate neuraxial block (change anesthetic)	Provided	NA	NA	NA	NA	NA	NA	NA	+	NA	NA	NA	NA	NA	NA	NA	NG	NA	NA	+	+	NG	+
-Nitrous oxide and oxygen alone	Disagree	NA	NA	NA	NA	NA	NA	NA	NG	NA	NA	NA	NA	NA	NA	NA	NG	NA	NA	+	NG	NG	NG
-Continue with neuraxial block, consider repeated boluses of fast acting opioids	Agree	NA	NA	NA	NA	NA	NA	NA	NG	NA	NA	NA	NA	NA	NA	NA	NG	NA	NA	+	NG	NG	NG
--Fentanyl	25-50 μg	NA	NA	NA	NA	NA	NA	NA	NG	NA	NA	NA	NA	NA	NA	NA	NG	NA	NA	+	NG	NG	NG
--Alfentanil	250-500 μg	NA	NA	NA	NA	NA	NA	NA	NG	NA	NA	NA	NA	NA	NA	NA	NG	NA	NA	+	NG	NG	NG
--Ketamine	10 mg boluses	NA	NA	NA	NA	NA	NA	NA	NG	NA	NA	NA	NA	NA	NA	NA	NG	NA	NA	+	10 mg or	NG	

Huang L, *et al. BMJ Open* 2024; 14:e084759. doi: 10.1136/bmjopen-2024-084759

																					0.15 mg .kg^{-1}		+/-
-Do not treat pain with anxiolytics	Agree	NA	NA	NA	NA	NA	NA	NA	NG	NA	NA	NA	NA	NA	NA	NA	NG	NA	NA	+	NG	NG	NG
-Above options are not possible, general anesthesia should be recommended	Agree	NA	NA	NA	NA	NA	NA	NA	+	NA	NA	NA	NA	NA	NA	NA	NG	NA	NA	+	+	NG	+
**3. Post dural puncture headache (PDPH)**	Provided	NA	NA	NA	NA	NA	NA	NA	+	NA	NA	NA	NA	NA	NA	NA	NA	NA	NA	NA	+	+/-	NA
3.1 Measures be used to prevent PDPH	Provided	NA	NA	NA	NA	NA	NA	NA	NG	NA	NA	NA	NA	NA	NA	NA	NA	NA	NA	NA	+/-	NG	NA
-Noncutting spinal needles are associated with decreased PDPH risk	Agree	NA	NA	NA	NA	NA	NA	NA	NG	NA	NA	NA	NA	NA	NA	NA	NA	NA	NA	NA	+	NG	NA
-An intrathecal catheter may be considered to provide anesthesia or analgesia	Agree	NA	NA	NA	NA	NA	NA	NA	NG	NA	NA	NA	NA	NA	NA	NA	NA	NA	NA	NA	+	NG	NA
-A prophylactic epidural blood patch is not recommended as routine	Agree	NA	NA	NA	NA	NA	NA	NA	NG	NA	NA	NA	NA	NA	NA	NA	NA	NA	NA	NA	NG	NG	NA
-Bed rest is not routinely recommended	Agree	NA	NA	NA	NA	NA	NA	NA	NG	NA	NA	NA	NA	NA	NA	NA	NA	NA	NA	NA	NG	NG	NA

as prophylaxis against PDPH																							
-Routine injection of substance to prevent PDPH is not recommended	Agree	NA	NA	NA	NA	NA	NA	NA	NG	NA	NA	NA	NA	NA	NA	NA	NA	NA	NA	NA	NG	NG	NA
-Routine systemic drug for PDPH prophylaxis is not recommended	Agree	NA	NA	NA	NA	NA	NA	NA	NG	NA	NA	NA	NA	NA	NA	NA	NA	NA	NA	NA	NG	NG	NA
3.2 Measures be used to treat PDPH	Provided	NA	NA	NA	NA	NA	NA	NA	NG	NA	NA	NA	NA	NA	NA	NA	NA	NA	NA	NA	+/-	NG	NA
-Epidural blood patch	Agree	NA	NA	NA	NA	NA	NA	NA	+	NA	NA	NA	NA	NA	NA	NA	NA	NA	NA	NA	+	NG	NA
-The routine use of bed rest to treat PDPH	Disagree	NA	NA	NA	NA	NA	NA	NA	NG	NA	NA	NA	NA	NA	NA	NA	NA	NA	NA	NA	NG	NG	NA
-Adequate hydration should be maintained with oral fluids or intravenous	Agree	NA	NA	NA	NA	NA	NA	NA	NG	NA	NA	NA	NA	NA	NA	NA	NA	NA	NA	NA	+	NG	NA
-Routine use of abdominal binders or aromatherapy to treat PDPH	Disagree	NA	NA	NA	NA	NA	NA	NA	NG	NA	NA	NA	NA	NA	NA	NA	NA	NA	NA	NA	NG	NG	NA
-Regular multimodal analgesia	Agree	NA	NA	NA	NA	NA	NA	NA	NG	NA	NA	NA	NA	NA	NA	NA	NA	NA	NA	NA	+/-	NG	NA
-Short-term use of opioids if regular	Agree	NA	NA	NA	NA	NA	NA	NA	NG	NA	NA	NA	NA	NA	NA	NA	NA	NA	NA	NA	NG	NG	NA

multimodal analgesia is ineffective																							
-Long-term opioid use is not recommended in the treatment of PDPH	Agree	NA	NA	NA	NA	NA	NA	NA	NG	NA	NA	NA	NA	NA	NA	NA	NA	NA	NA	NA	NG	NG	NA
-Caffeine offered in the first 24h with a maximum dose of 900 mg per day	Agree	NA	NA	NA	NA	NA	NA	NA	NG	NA	NA	NA	NA	NA	NA	NA	NA	NA	NA	NA	+/-	NG	NA
-Caffeine offered 200-300 mg if breastfeeding	Agree	NA	NA	NA	NA	NA	NA	NA	NG	NA	NA	NA	NA	NA	NA	NA	NA	NA	NA	NA	NG	NG	NA
-Routine use of hydrocortisone, adrenocorticotropic hormone or cosyntropin	Disagree	NA	NA	NA	NA	NA	NA	NA	NG	NA	NA	NA	NA	NA	NA	NA	NA	NA	NA	NA	+/-	NG	NA
-Routine use of theophylline, neostigmine or atropine, piritramide, and gabapentin	Disagree	NA	NA	NA	NA	NA	NA	NA	NG	NA	NA	NA	NA	NA	NA	NA	NA	NA	NA	NA	NG	NG	NA
-Routine use of acupuncture to treat PDPH	Disagree	NA	NA	NA	NA	NA	NA	NA	NG	NA	NA	NA	NA	NA	NA	NA	NA	NA	NA	NA	NG	NG	NA
-Routine use of sphenopalatine ganglion blocks to treat PDPH	Disagree	NA	NA	NA	NA	NA	NA	NA	NG	NA	NA	NA	NA	NA	NA	NA	NA	NA	NA	NA	+	NG	NA

-Greater occipital nerve blocks be offered to patients with PDPH	Agree	NA	NA	NA	NA	NA	NA	NA	NG	NA	NA	NA	NA	NA	NA	NA	NA	NA	NA	NA	NG	NG	NA
-The use of spinal and epidural morphine to treat PDPH	Disagree	NA	NA	NA	NA	NA	NA	NA	NG	NA	NA	NA	NA	NA	NA	NA	NA	NA	NA	NA	NG	NG	NA
-Routine use of epidural dextran, gelatine, or hydroxyethyl starch to treat PDPH	Disagree	NA	NA	NA	NA	NA	NA	NA	NG	NA	NA	NA	NA	NA	NA	NA	NA	NA	NA	NA	NG	NG	NA
-Routine use of fibrin glue to treat PDPH	Disagree	NA	NA	NA	NA	NA	NA	NA	NG	NA	NA	NA	NA	NA	NA	NA	NA	NA	NA	NA	NG	NG	NA
-Fibrin glue should be reserved for management of PDPH refractory to EBP	Agree	NA	NA	NA	NA	NA	NA	NA	NG	NA	NA	NA	NA	NA	NA	NA	NA	NA	NA	NA	NG	NG	NA
-Fibrin glue be used when autologous blood injection is contraindicated	Agree	NA	NA	NA	NA	NA	NA	NA	NG	NA	NA	NA	NA	NA	NA	NA	NA	NA	NA	NA	NG	NG	NA

+: consistent the reference with content;

+/-: partial1y consistent the reference with content;

-: inconsistent the reference with content;

NA: not applicable;

NG: not given.

Supplementary Table 15. Summary of recommendations for the monitoring and management of obstetric and anesthetic emergencies by included guidance document.

		OG-OG-CMA_2014	OG-A-CMA_2016	ASA_2016	AAGBI_2017	ERAS_2018 (Part 1)	ERAS_2018 (Part 2)	ERAS_2018 (Part 3)	ACOG_2019	SOGC_2019	SOGC_2022	SOAP_2019	AA and OAA_2020	PROSPECT_2020	CHBSA_2020	CAA_2020	CAA_2020	SOAP_2020	NICE_2021	OAA_2022	ANNA_2023	RCoA_2023	French_2021
**Recommendations**	**Reference contents**																						
**1. Resources for management of Hemorrhagic emergencies**	Provided	NA	NA	+	NA	NA	NA	NA	NA	NA	NA	NA	NA	NA	NA	+/-	NA	NA	NA	NA	+	+/-	NA
-Large-bore IV catheters	Agree	NA	NA	+	NA	NA	NA	NA	NA	NA	NA	NA	NA	NA	NA	+	NA	NA	NA	NA	+	NG	NA
-Fluid warmer	Agree	NA	NA	+	NA	NA	NA	NA	NA	NA	NA	NA	NA	NA	NA	NG	NA	NA	NA	NA	NG	NG	NA
-Forced-air body warmer	Agree	NA	NA	+	NA	NA	NA	NA	NA	NA	NA	NA	NA	NA	NA	NG	NA	NA	NA	NA	NG	NG	NA
-Availability of blood bank resources	Agree	NA	NA	+	NA	NA	NA	NA	NA	NA	NA	NA	NA	NA	NA	NG	NA	NA	NA	NA	+	NG	NA
-Massive transfusion protocol (type-specific, or O-negative blood, or salvage)	Agree	NA	NA	+	NA	NA	NA	NA	NA	NA	NA	NA	NA	NA	NA	NG	NA	NA	NA	NA	+	NG	NA
-Equipment for infusing IV fluids and blood products rapidly	Agree	NA	NA	+	NA	NA	NA	NA	NA	NA	NA	NA	NA	NA	NA	NG	NA	NA	NA	NA	NG	NG	NA

**2. Equipment for management of airway emergencies**	Provided	NA	NA	+	NA	NA	NA	NA	+	+/-	NA	NA	NA	NA	NA	+	NA	NA	+	NA	+/-	+/-	NA
2.1 Laryngoscope and assorted blades	Agree	NA	NA	+	NA	NA	NA	NA	+	NG	NA	NA	NA	NA	NA	NG	NA	NA	NG	NA	+	NG	NA
2.2 Endotracheal tubes, with stylets	Agree	NA	NA	+	NA	NA	NA	NA	+	NG	NA	NA	NA	NA	NA	NG	NA	NA	NG	NA	+	NG	NA
2.3 Oxygen source	Agree	NA	NA	+	NA	NA	NA	NA	NG	NG	NA	NA	NA	NA	NA	NG	NA	NA	+	NA	+	NG	NA
2.4 Suction source with tubing and tonsil suction tip	Agree	NA	NA	+	NA	NA	NA	NA	NG	NG	NA	NA	NA	NA	NA	NG	NA	NA	+	NA	+	NG	NA
2.5 Self-inflating bag and mask for positive-pressure ventilation	Agree	NA	NA	+	NA	NA	NA	NA	NG	NG	NA	NA	NA	NA	NA	NG	NA	NA	NG	NA	+	NG	NA
2.6 Medications for blood pressure support, muscle relaxation, and hypnosis	Agree	NA	NA	+	NA	NA	NA	NA	NG	NG	NA	NA	NA	NA	NA	NG	NA	NA	NA	NA	NG	NG	NA
2.7 Tracheal intubation has failed protocol	Agree	NA	NA	+	NA	NA	NA	NA	NG	NG	NA	NA	NA	NA	NA	+	NA	NA	+	NA	+	NG	NA
**3. Cardiopulmonary Resuscitation**	Provided	NA	NA	+	NA	NA	NA	NA	NA	NA	NA	NA	NA	NA	NA	NA	NA	NA	NA	NA	+/-	+/-	NA
3.1 Basic and advanced life-support equipment	Agree	NA	NA	+	NA	NA	NA	NA	NA	NA	NA	NA	NA	NA	NA	NA	NA	NA	NA	NA	+	NG	NA

Huang L, *et al. BMJ Open* 2024; 14:e084759. doi: 10.1136/bmjopen-2024-084759

3.2 Cardiac arrest occurs	Provided	NA	NA	+	NA	NA	NA	NA	NA	NA	NA	NA	NA	NA	NA	NA	NA	NA	NA	NA	+	NG	NA
-Uterine displacement (usually left displacement) should be maintained	Agree	NA	NA	+	NA	NA	NA	NA	NA	NA	NA	NA	NA	NA	NA	NA	NA	NA	NA	NA	+	NG	NA
-If circulation is not restored within 4 min, cesarean should be performed	Agree	NA	NA	+	NA	NA	NA	NA	NA	NA	NA	NA	NA	NA	NA	NA	NA	NA	NA	NA	4-5 min	NG	NA
**4. Emergency cesarean delivery**	Provided	NA	NA	+	NA	NA	NA	NA	+	NA	NA	NA	NA	NA	NA	NA	NA	NA	+	NA	+/-	+/-	+
4.1 Response times limited	30 or 75 minutes	NA	NA	NG	NA	NA	NA	NA	NG	NA	NA	NA	NA	NA	NA	NA	NA	NA	+	NA	NG	NG	15-30 minutes
4.2 Anesthesia methods recommendation	Provided	NA	NA	+/-	NA	NA	NA	NA	+	NA	NA	NA	NA	NA	NA	NA	NA	NA	+/-	NA	NG	NG	+
-Spinal	Agree	NA	NA	+/-	NA	NA	NA	NA	+	NA	NA	NA	NA	NA	NA	NA	NA	NA	NG	NA	NG	NG	+
-Epidural	Agree	NA	NA	+/-	NA	NA	NA	NA	+	NA	NA	NA	NA	NA	NA	NA	NA	NA	NG	NA	NG	NG	+
-Combined spinal–epidural analgesia	Agree	NA	NA	+/-	NA	NA	NA	NA	+	NA	NA	NA	NA	NA	NA	NA	NA	NA	NG	NA	NG	NG	+
-General anesthesia	Agree	NA	NA	+/-	NA	NA	NA	NA	+	NA	NA	NA	NA	NA	NA	NA	NA	NA	NG	NA	NG	NG	+

+/-: partial1y consistent the reference with content;

-: inconsistent the reference with content;

NA: not applicable;

NG: not given.

Huang L, *et al. BMJ Open* 2024; 14:e084759. doi: 10.1136/bmjopen-2024-084759
