# Supplementary material for: Appraisal of clinical practice guidelines and consensus statements on obstetric anaesthesia: a systematic review using the AGREE II instrument
Source: BMJ Open. 2024 May 28;14(5):e084759. doi: 10.1136/bmjopen-2024-084759 (PMC11138284; doi:10.1136/bmjopen-2024-084759)
Supplement: Supplementary data [file bmjopen-2024-084759supp002.pdf]

C = ■ Consistent with the reference content
I = ■ Inconsistent with the reference content
P = ■ Partially consistent with the reference content
NG = ■Not given
NA = ■Not applicable

Recommendations	Reference contents	OG-OG-CMA_2014	OG-A-CMA_2016	ASA_2016	AAGBI_2017	ERAS_2018 (Part 1)	ERAS_2018 (Part 2)	ERAS_2018 (Part 3)	ACOG_2019	SOGC_2019	SOGC_2022	SOAP_2019	AA and OAA_2020	PROSPECT_2020	CHBSA_2020	CAA_2020	CAA_2020	SOAP_2020	NICE_2021	OAA_2022	ANNA_2023	RCoA_2023	French_2021
**Peri-anesthetic Evaluation and Preparation**																							
**1. History and physical examination before providing anesthetic care**	Yes	C	C	C	NA	NG	NA	NA	C	NA	NA	NA	NA	NA	C	C	C	NG	P	NA	C	NG	C
1.1 Maternal health and anesthetic history	Agree	C	C	C	NA	NG	NA	NA	C	NA	NA	NA	NA	NA	C	C	C	NG	NG	NA	P	NG	C
1.2 Relevant obstetric history	Agree	C	C	C	NA	NG	NA	NA	NG	NA	NA	NA	NA	NA	NG	C	C	NG	NG	NA	NG	NG	NG
1.3 Baseline blood pressure measurement	Agree	C	C	C	NA	NG	NA	NA	NG	NA	NA	NA	NA	NA	C	I	C	NG	NG	NA	P	NG	NG
1.4 Airway examination	Agree	NG	C	C	NA	NG	NA	NA	C	NA	NA	NA	NA	NA	NG	C	C	NG	NG	NA	C	NG	NG
1.5 Heart and lung examination	Agree	NG	NG	C	NA	NG	NA	NA	C	NA	NA	NA	NA	NA	C	C	C	NG	NG	NA	C	NG	NG
1.6 When a neuraxial anesthetic is planned, examine the patient's back	Agree	NG	C	C	NA	NG	NA	NA	C	NA	NA	NA	NA	NA	NG	C	C	NG	NG	NA	C	NG	NG
1.7 Consultation between the obstetrician and the anesthesiologist	Agree	C	NG	C	NA	C	NA	NA	C	NA	NA	NA	NA	NA	NG	C	C	NG	NG	NA	C	NG	C
**2. Preoperative communication**	Encouraged	C	NG	C	NA	C	NA	NA	NG	NA	NA	NA	NA	NA	C	C	I	P	C	NA	C	P	C
**3. Intrapartum platelet count**	Yes	C	C	C	NA	NA	NA	NA	C	NA	NA	NA	NA	NA	NG	C	NG	NG	C	NA	NG	NG	NA
-Routine	Agree	C	C	C	NA	NA	NA	NA	C	NA	NA	NA	NA	NA	NG	NG	NG	NG	I	NA	NG	NG	NA
-Individualized	Agree	I	I	C	NA	NA	NA	NA	C	NA	NA	NA	NA	NA	NG	NG	NG	NG	C	NA	NG	NG	NA
**4. Blood type and screen**	Yes	C	C	C	NA	NA	NA	NA	NG	NA	NA	NA	NA	NA	NG	C	NG	NG	C	NA	NG	NG	NA
-Routine	Agree	C	C	I	NA	NA	NA	NA	NG	NA	NA	NA	NA	NA	NG	C	NG	NG	I	NA	NG	NG	NA
-Individualized	Agree	I	I	C	NA	NA	NA	NA	NG	NA	NA	NA	NA	NA	NG	NG	NG	NG	C	NA	NG	NG	NA
**5. Blood cross-match**	Yes	C	NG	C	NA	NA	NA	NA	NG	NA	NA	NA	NA	NA	NG	NG	NG	NG	C	NA	NG	NG	NA
-Routine	Agree	C	NG	I	NA	NA	NA	NA	NG	NA	NA	NA	NA	NA	NG	NG	NG	NG	I	NA	NG	NG	NA
-Individualized	Agree	NG	NG	C	NA	NA	NA	NA	NG	NA	NA	NA	NA	NA	NG	NG	NG	NG	C	NA	NG	NG	NA
6. Peri-anesthetic recording of fetal heart rate	Yes	C	C	C	NA	NA	NA	NA	C	C	C	NA	NA	NA	NG	C	NG	NG	NG	NA	C	NG	NA
**7. Aspiration prevention**	Provided	C	NG	C	NA	C	NA	NA	NA	C	NA	NA	NA	NA	C	P	NG	P	P	NA	P	P	NA
7.1 Clear liquids	Up to 2 h	NG	NG	C	NA	C	NA	NA	NA	NG	NA	NA	NA	NA	C	C	NG	C	NG	NA	NG	P	NA
7.2 Solids	6-8 h	C	NG	C	NA	C	NA	NA	NA	NG	NA	NA	NA	NA	C	C	NG	C	NG	NA	NG	NG	NA
7.3 Antacids (surgical procedures-caesarean delivery or postpartum tubal ligation)	Agree	NG	NG	C	NA	C	NA	NA	NA	NG	NA	NA	NA	NA	C	C	NG	NG	C	NA	NG	C	NA
7.4 H_2-receptor antagonists (surgical procedures)	Agree	NG	NG	C	NA	C	NA	C	NA	NG	NA	NA	NA	NA	C	C	NG	NG	C	NA	NG	NG	NA
7.5 Metoclopramide (surgical procedures)	Agree	NG	NG	C	NA	NG	NA	C	NA	NG	NA	NA	NA	NA	NG	C	NG	NG	NG	NA	NG	NG	NA
**8. Preoperative heparin or low-molecular-weight heparin**	Yes	NG	C	NG	NA	NA	NA	NA	C	NA	NA	NA	NA	NA	NA	NG	P	P	NG	NA	P	P	NA
8.1 Prophylactic subcutaneous unfractionated heparin (<5,000 units twice daily)	Provided	NG	NG	NG	NA	NA	NA	NA	C	NA	NA	NA	NA	NA	NA	NG	NG	NG	NG	NA	NG	NG	NA
-Contraindication to neuraxial techniques	No	NG	NG	NG	NA	NA	NA	NA	C	NA	NA	NA	NA	NA	NA	NG	NG	NG	NG	NA	NG	NG	NA
-Catheter placement or removal since last dose	4-6 hours	NG	NG	NG	NA	NA	NA	NA	C	NA	NA	NA	NA	NA	NA	NG	NG	NG	NG	NA	NG	NG	NA
8.2 Intermediate unfractionated heparin (75,000-100,000 units)	Provided	NG	NG	NG	NA	NA	NA	NA	C	NA	NA	NA	NA	NA	NA	NG	NG	NG	NG	NA	NG	NG	NA
-Catheter placement or removal since last dose	> 12 hours	NG	NG	NG	NA	NA	NA	NA	C	NA	NA	NA	NA	NA	NA	NG	NG	NG	NG	NA	NG	NG	NA
8.3 High-dose unfractionated heparin (total daily dose greater than 20,000 units)	Provided	NG	NG	NG	NA	NA	NA	NA	C	NA	NA	NA	NA	NA	NA	NG	NG	NG	NG	NA	NG	NG	NA
-Catheter placement or removal since last dose	> 24 hours	NG	NG	NG	NA	NA	NA	NA	C	NA	NA	NA	NA	NA	NA	NG	NG	NG	NG	NA	NG	NG	NA
-With the activated partial thromboplastin time being within normal range	Yes	NG	NG	NG	NA	NA	NA	NA	C	NA	NA	NA	NA	NA	NA	NG	NG	NG	NG	NA	NG	NG	NA
**Anesthetic Care for labor and Vaginal Delivery**																							
**1. Equipment, facilities, and support personnel**	Agree	NA	C	C	NA	NA	NA	NA	C	NA	NA	NA	NA	NA	C	NA	C	NA	NA	NA	C	C	NA
**2. Timing of neuraxial analgesia and outcome of labor**	Provided	NA	C	C	NA	NA	NA	NA	NG	NA	NA	NA	NA	NA	NG	NA	C	NA	NA	NA	NG	NG	NA

2.1 Less 5 cm dilation	Agree
2.2 Offer neuraxial analgesia on an individualized basis	Agree
**3. Neuraxial analgesia and trial of labor after prior caesarean delivery**	Agree
**4. Continuous infusion epidural analgesia**	Provided
4.1 Using local anesthetic	Agree
4.2 Add opioid	Agree
4.3 Platelet concentration corresponding to contraindications to puncture	Less than $70*10^9$/L
4.4 Puncture location	L_{2-3} or L_{3-4}
4.5 Analgesic concentrations	Provided
-Bupivacaine	Provided
--Loading dose	0.04-0.125%
--Maintenance dose	0.05-0.125%
-Ropivacaine	Provided
--Loading dose	0.0625-0.15%
--Maintenance dose	0.0625-0.125%
-Levobupivacaine	Provided
--Loading dose	0.04-0.125%
--Maintenance dose	0.05-0.125%
4.6 Opioids concentrations	Provided
-Fentanyl	Provided
--Loading dose	0.5-2 μg/ml
--Maintenance dose	1-2 μg/ml
-Sufentanyl	Provided
--Loading dose	0.2~0.6 μg/ml
--Maintenance dose	0.3~0.6 μg/ml
**5. Combined spinal–epidural analgesia**	Provided
5.1 Using local anesthetic	Agree
5.2 Add opioid	Agree
5.3 Platelet concentration corresponding to contraindications to puncture	Less than $70*10^9$/L
5.4 Puncture location	L_{2-3} or L_{3-4}
5.5 Epidural anesthetics (consistent with 4.5 and 4.6)	Provided
5.6 Spinal anesthetics	Provided
-Protocol 1	Provided
--Single sufentanyl	2.5-7 μg
--Single ropivacaine	2.3-3 mg
--Combined sufentanyl and ropivacaine	2.5 μg / 2.5 mg
-Protocol 2	Provided
--Single fentanyl	15-25 μg
--Single bupivacaine	2.0-2.5 mg
--Combined fentanyl and bupivacaine	2.5 μg / 2.0 mg
**6. Single-injection spinal**	Provided
6.1 Using local anesthetic	Agree
6.2 Add opioid	Agree
6.3 Platelet concentration corresponding to contraindications to puncture	Less than $50*10^9$/L
6.4 Puncture location	L_{3-4}

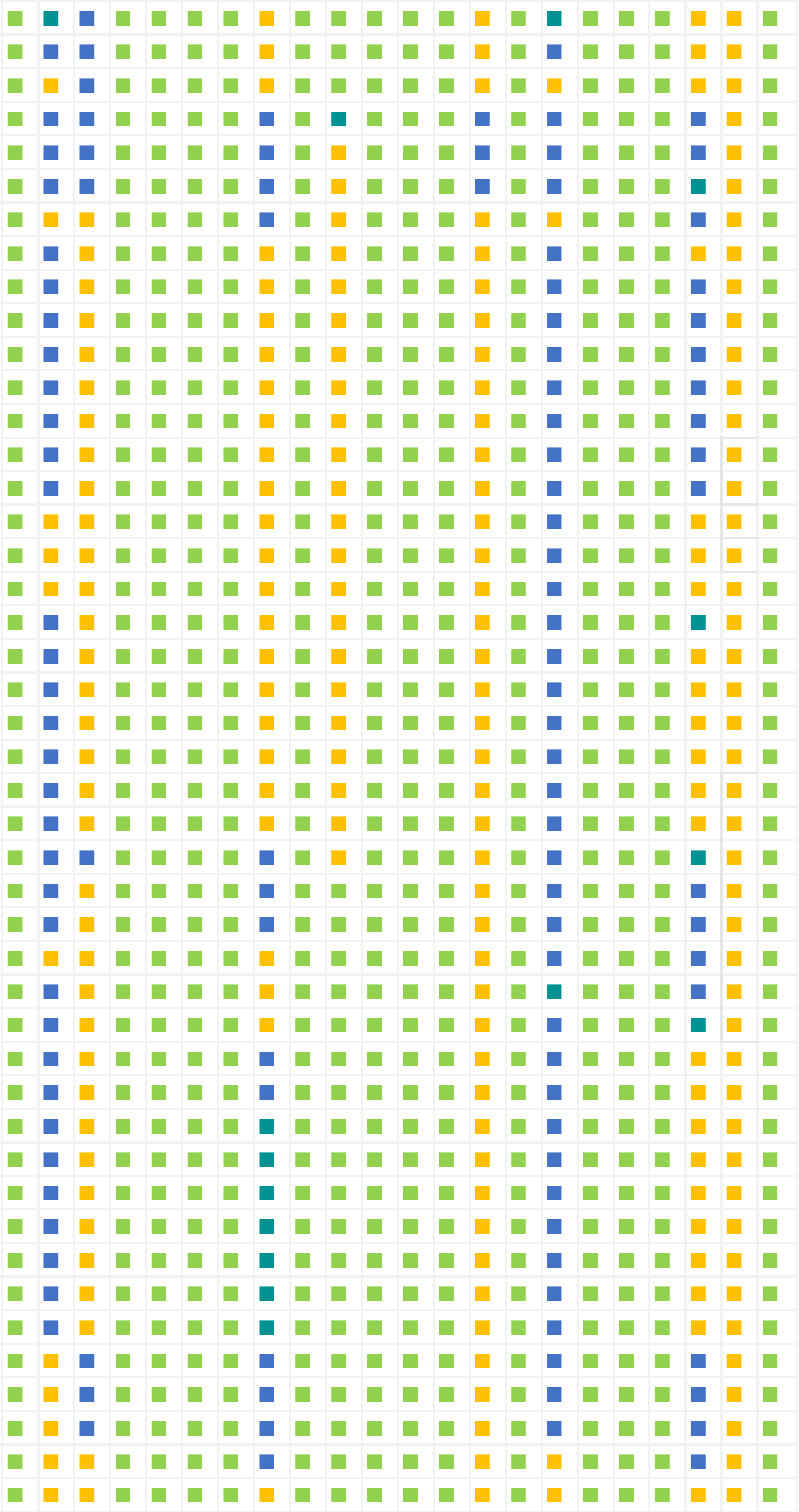

6.6 Analgesic concentrations (consistent with 5.6)	Agree
6.7 Spinal needles	Provided
-Cutting-bevel	Disagree
-Pencil-point	Agree
**7. Patient-controlled epidural analgesia (for labor analgesia)**	Agree
**Anesthetic Care for caesarean Delivery**	
**1. Equipment, facilities, and support personnel**	Agree
**2. Continuous infusion epidural analgesia**	Provided
2.1 Using local anesthetic	Agree
2.2 Add opioid	Agree
2.3 Platelet concentration corresponding to contraindications to puncture	Less than $70*10^9$/L
2.4 Puncture location	L_{1-2} or L_{2-3}
2.5 Recommend level of block	T_{6-4}
2.6 Analgesic concentrations	Provided
-Lidocaine	1.5%-2%
-Bupivacaine	0.50%
-Ropivacaine	0.5%-0.75%
-Levobupivacaine	0.5%-0.75%
-Cloprocaine	3%
2.7 Prevention of local anesthetic poisoning	Provided
-Withdrawal before injection	Agree
-Administration of test dose	Agree
-Addition of epinephrine	Agree
**3. Single-injection spinal**	Provided
3.1 Using local anesthetic	Agree
3.2 Add opioid	Agree
3.3 Platelet concentration corresponding to contraindications to puncture	Less than $70*10^9$/L
3.4 Puncture location	L_{3-4}
3.5 Recommend level of block	T_{6-4}
3.6 Analgesic concentrations	Provided
-Bupivacaine	5~15 mg
-Ropivacaine	10~20 mg
3.7 Spinal needles	Provided
-Cutting-bevel	Disagree
-Pencil-point	Agree
**4. Combined spinal–epidural analgesia**	Provided
4.1 Puncture location	Provided
-Single space technique	L_{3-4}
-Double-space technique	T_{12}-L_2/L_{3-5}
4.2 Anesthetics (consistent with spinal or epidural)	Agree
**5. General anesthesia**	Provided
5.1 General anesthesia selected condition	Provided
-Profound fetal bradycardia	Agree
-Ruptured uterus	Agree
-Severe hemorrhage	Agree

-Severe placental abruption	Agree
-Umbilical cord prolapse	Agree
-Preterm footling breech	Agree
-Inability to place neuraxial anesthesia	
5.2 Intravenous anesthetics recommendation	Provided
-Thiopental sodium	4-5 mg/kg
-Propofol	1.5-2.5 mg/kg
-Etomidate	0.2-0.3 mg/kg
-Ketamine	0.5-1 mg/kg
5.3 Opioids analgesic recommendation	Provided
-Fentanyl	2-5 μg/kg
-Sufentanyl	0.2-0.5 μg/kg
-Remifentanyl	0.5-1 μg/kg for IV
-Remifentanyl	4 ng/kg for target
5.4 Muscle relaxants recommendation	Provided
-Succinylcholine	1-1.5 mg/kg
-Rocuronium	0.6-1.2 mg/kg
5.5 Induction-delivery interval	within 10 min
**Neurological Monitoring Associated With Obstetric Neuraxial Block**	
**1. Screening method (During the recovery phase) -Straight-leg raising**	Agree
**2. Alerts incidences-unable to straight-leg raise at 4 h from the last dose**	Agree
**Respiratory Depression Monitoring With Neuraxial Block With Opioids**	
**1. Frequency and modality of respiratory monitoring should be based on:**	Provided
1.1 Low risk	Provided
-Neuraxial morphine dose (intrathecal ≤ 0.15 mg or epidural ≤ 3 mg)	Agree
1.2 High risk	Provided
-Cardiopulmonary/neurological co-morbidity	Agree
-Obesity (body mass index ≥ 40 kg/m^2)	Agree
-Known or suspected obstructive sleep apnea	Agree
-Chronic opioid use or abuse	Agree
-Hypertension	Agree
-Magnesium administration	Agree
-Neuraxial morphine dose (intrathecal > 0.15 mg or epidural > 3 mg)	Agree
1.3 Respiratory monitoring frequency and duration	Provided
-Low risk	Provided
--Routine postoperative vital sign monitoring	Agree
-- q2h clinical assessment for 12 h	Agree
-High risk	Provided
-- q1h clinical assessment for first 12 h	Agree
-- q2h clinical assessment for first 12-24 h	Agree
--Consider additional monitoring modalities (e.g., pulse oximetry, capnography)	Agree
**Management of Obstetric and anesthetic Emergencies or Adverse Events**	
**1. Resources for management of hemorrhagic emergencies**	Provided
-Large-bore IV catheters	Agree
-Fluid warmer	Agree
-Forced-air body warmer	Agree

-Availability of blood bank resources	Agree
-Massive transfusion protocol (type-specific, or O-negative blood, or salvage)	Agree
-Equipment for infusing IV fluids and blood products rapidly	Agree
**2. Hypotension prevention strategies**	Provided
2.1 Blood pressure target of systolic arterial pressure	⩾ 90% of baseline obtained
2.2 Position (uterine left-leaning)	Yes
2.3 Volume expansion (crystals or colloids)	500-1000 ml
2.4 Vasoactive drugs	Yes
-Pump infusion is recommended	Provided
-Type of drugs	Provided
--Phenylephrine	20-50 μg or 0.5 μg. kg^{-1}. min^{-1}
--Methoxymine	1-2 mg or 4 μg. kg-1. min-1
--Noradrenalin	4-6 μg or 0.08 μg. kg-1. min-1
--Efedrina	5-15 mg for IV
2.5 Prophylactic vasopressor infusion should not be recommended in pre-eclampsia	Agree
**3. Equipment for management of airway emergencies**	Provided
3.1 Laryngoscope and assorted blades	Agree
3.2 Endotracheal tubes, with stylets	Agree
3.3 Oxygen source	Agree
3.4 Suction source with tubing and tonsil suction tip	Agree
3.5 Self-inflating bag and mask for positive-pressure ventilation	Agree
3.6 Medications for blood pressure support, muscle relaxation, and hypnosis	Agree
3.7 Tracheal intubation has failed protocol	Agree
**4. Cardiopulmonary Resuscitation**	Provided
4.1 Basic and advanced life-support equipment	Agree
4.2 Cardiac arrest occurs	Provided
-Uterine displacement (usually left displacement) should be maintained	Agree
-If circulation is not restored within 4 min, caesarean should be performed	Agree
**5. Emergency caesarean delivery**	Provided
5.1 Response times limited	30 minutes
5.2 Anesthesia methods recommendation	Provided
-Spinal	Agree
-Epidural	Agree
-Combined spinal–epidural analgesia	Agree
-General anesthesia	Agree
**6. Inadequate neuraxial anesthesia**	Provided
6.1 Block assessment	Provided
-Testing sensory block after the evidence of motor block	Agree
-Check the block at relatively fixed time intervals	Agree
-Perform the first check early enough to allow positional changes	Agree
6.2 Rescue for inadequate neuraxial block (change anesthesia method)	Provided
-A second neuraxial technique (in the case of spinal anesthesia)	Agree
-Extending the neuraxial technique (in the case of combined spinal–epidural)	Agree
-Above options are not possible, general anesthesia should be recommended	Agree

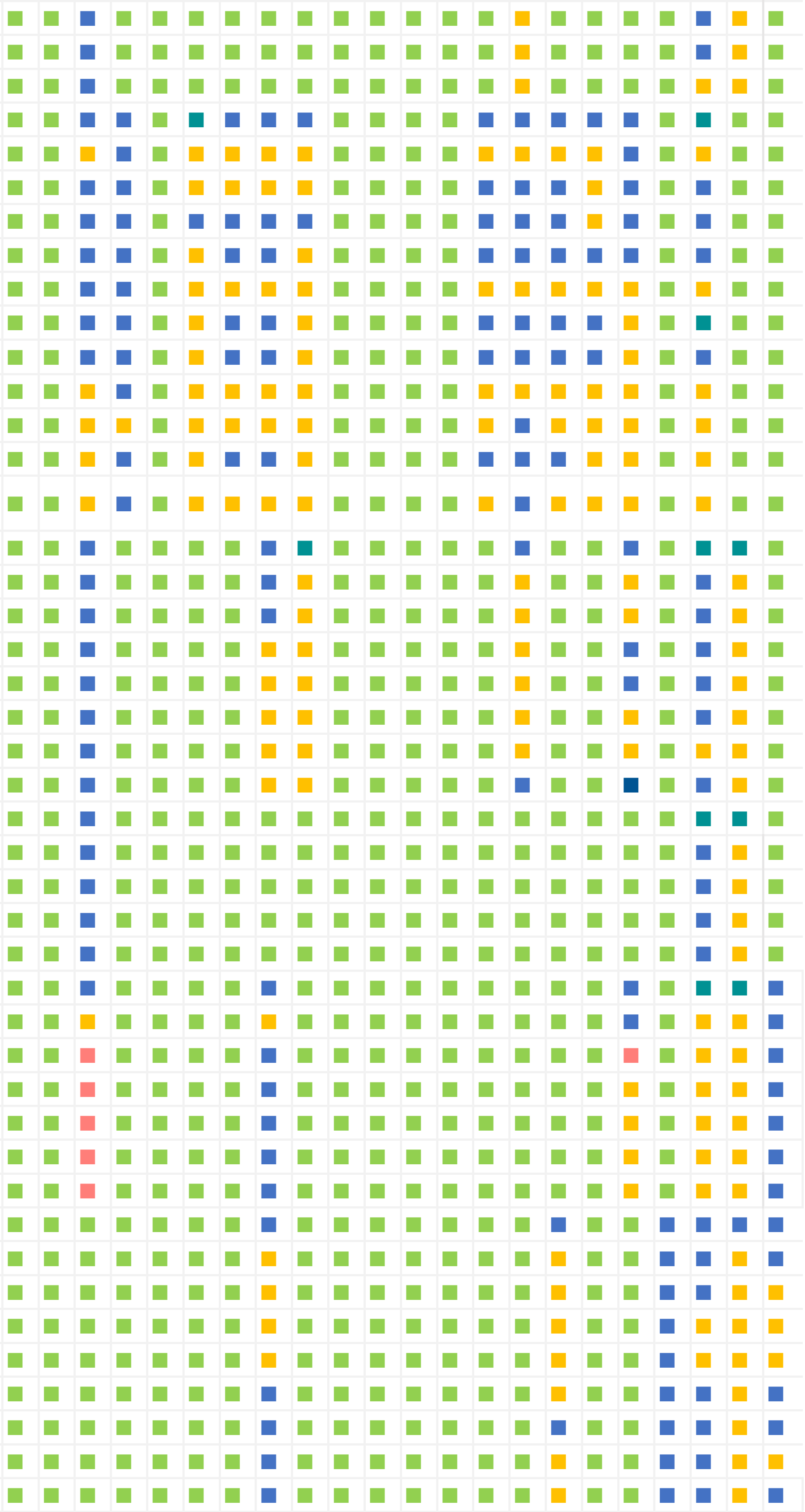

6.3 Rescue for inadequate neuraxial block (change anesthetic)	Provided
-Nitrous oxide and oxygen alone	Disagree
-Continue with neuraxial block, consider repeated boluses of fastacting opioids	Agree
--Fentanyl	25-50 μg
--Alfentanil	250-500 μg
--Ketamine	10 mg boluses
-Do not treat pain with anxiolytics	Agree
-Above options are not possible, general anesthesia should be recommended	Agree
**Management of Intra- or Postoperative Pain**	
**1. Implement strategies to minimize systemic opioid utilization**	Provided
**2. Neuraxial adjuvant drugs**	Provided
-Spinal anesthesia	Provided
--Morphine	50-100 μg
--Hydromorphone	75-150 μg
-Epidural or combined spinal-epidural technique.	Provided
--Morphine	2-3 mg
--Diamorphine	2-3 mg
**3. Others analgesic (after delivery)**	Provided
-Non-steroidal anti-inflammatory drug (e.g., paracetamol)	Agree
-A single dose of intravenous dexamethasone	Agree
**4. Local anesthetic infiltration**	Agree
**5. Continuous wound local anesthetic infusion**	Agree
**6. Fascial plane blocks (e.g., transversus abdominis plane, erector spinae plane)**	Agree
**7. Patient-controlled intravenous analgesia**	Agree
