# Supplementary material for: Appraisal of clinical practice guidelines and consensus statements on obstetric anaesthesia: a systematic review using the AGREE II instrument
Source: BMJ Open. 2024 May 28;14(5):e084759. doi: 10.1136/bmjopen-2024-084759 (PMC11138284; doi:10.1136/bmjopen-2024-084759)
Supplement: Author's manuscript [file bmjopen-2024-084759.draft_revisions.pdf]

BMJ Open is committed to open peer review. As part of this commitment we make the peer review history of every article we publish publicly available.

When an article is published we post the peer reviewers' comments and the authors' responses online. We also post the versions of the paper that were used during peer review. These are the versions that the peer review comments apply to.

The versions of the paper that follow are the versions that were submitted during the peer review process. They are not the versions of record or the final published versions. They should not be cited or distributed as the published version of this manuscript.

BMJ Open is an open access journal and the full, final, typeset and author-corrected version of record of the manuscript is available on our site with no access controls, subscription charges or pay-per-view fees (http://bmjopen.bmj.com).

If you have any questions on BMJ Open's open peer review process please email info.bmjopen@bmj.com

Appraisal of clinical practice guidelines and consensus statements on obstetric anesthesia: A systematic review using the AGREE II instrument

Journal:	*BMJ Open*
Manuscript ID	bmjopen-2024-084759
Article Type:	Original research
Date Submitted by the Author:	27-Jan-2024

Complete List of Authors:	Huang, Lu; West China Second Hospital of Sichuan University, Department of anesthesiology; Ministry of Education<br>Hu, Na; Sichuan University West China Second University Hospital, Department of Anesthesiology<br>Jiang, Ling; West China Second Hospital of Sichuan University, Department of anesthesiology; Ministry of Education<br>Xinglong, Xiong; Affiliated Hospital of Guizhou Medical University<br>Shi, Jing; The Affiliated Hospital of Guizhou Medical University<br>Chen, Dongxu; West China Second Hospital of Sichuan University, Department of anesthesiology; Ministry of Education

Keywords:	Anaesthesia in obstetrics < ANAESTHETICS, Pain management < ANAESTHETICS, PAIN MANAGEMENT

SCHOLARONE™
Manuscripts

I, the Submitting Author has the right to grant and does grant on behalf of all authors of the Work (as defined in the below author licence), an exclusive licence and/or a non-exclusive licence for contributions from authors who are: i) UK Crown employees; ii) where BMJ has agreed a CC-BY licence shall apply, and/or iii) in accordance with the terms applicable for US Federal Government officers or employees acting as part of their official duties; on a worldwide, perpetual, irrevocable, royalty-free basis to BMJ Publishing Group Ltd ("BMJ") its licensees and where the relevant Journal is co-owned by BMJ to the co-owners of the Journal, to publish the Work in this journal and any other BMJ products and to exploit all rights, as set out in our licence.

The Submitting Author accepts and understands that any supply made under these terms is made by BMJ to the Submitting Author unless you are acting as an employee on behalf of your employer or a postgraduate student of an affiliated institution which is paying any applicable article publishing charge ("APC") for Open Access articles. Where the Submitting Author wishes to make the Work available on an Open Access basis (and intends to pay the relevant APC), the terms of reuse of such Open Access shall be governed by a Creative Commons licence – details of these licences and which Creative Commons licence will apply to this Work are set out in our licence referred to above.

Other than as permitted in any relevant BMJ Author's Self Archiving Policies, I confirm this Work has not been accepted for publication elsewhere, is not being considered for publication elsewhere and does not duplicate material already published. I confirm all authors consent to publication of this Work and authorise the granting of this licence.

For peer review only

Appraisal of clinical practice guidelines and consensus statements on obstetric anesthesia: A systematic review using the AGREE II instrument

Lu Huang[1,2], Na Hu[1,2], Ling Jiang[1,2], Xing Long, Xiong[3], Jing Shi[3], Dong Xu Chen[1,2, *] ,

Affiliation Information for All Authors:

1. Department of anesthesiology, West China Second Hospital of Sichuan University, Sichuan Province, Chengdu, 610041, China
2. Key Laboratory of Birth Defects and Related Diseases of Women and Children (Sichuan University), Ministry of Education
3. Department of Anesthesiology, The Affiliated Hospital of Guizhou Medical University, Guiyang, China

Corresponding to:
Dong Xu Chen, PhD. Department of anesthesiology, West China Second Hospital of Sichuan University, Sichuan Province, Chengdu, 610041, China (e-mail: scucdx@foxmail.com)

For peer review only

Abstract

Objectives: Despite the publication of hundreds of trials on obstetric anesthesia, the management of these conditions remains suboptimal. We aimed to assess the quality and consistency of guidance documents for obstetric anesthesia.

Methods: A systematic literature search was conducted using the databases PubMed and EMBASE, three Chinese academic databases, six guideline databases, and Google and Google scholar. The latest version of guidance documents for the obstetric anesthetic management published in English or Chinese were included. Two reviewers independently screened the searched items and extracted data. Four reviewers independently scored documents using the appraisal of guidelines for research and evaluation (AGREE) II methodology.

Results: Twenty-one guidance documents (13 clinical practice guidelines and 8 consensus statements) published between 2004 and 2023 were included. Included documents performed well in the domains of scope and purpose [median 76.4%, interquartile range (IQR) 69.4 to 79.2%] and clarity of presentation (median 72.2%, IQR 69.1% to 80.6%), but were unsatisfactory in applicability (median 20.8%, IQR 13.5% to 27.1%) and editorial independence (median 47.9%, IQR 0.0% to 75.0%). The majority of obstetric anesthesia guidelines or consensus centered on different topics. Less than thirty percent of them specifically addressed the management of obstetric anesthesia perioperatively. Recommendations were concordant on the perioperative preparation, and on some indications for the choice of anesthesia method. Substantially inconsistent recommendations were provided for some items, especially for

For peer review only - http://bmjopen.bmj.com/site/about/guidelines.xhtml

preoperative blood type and screen, and for the types and doses of neuraxial administration.

Conclusions: The methodological quality in guidance documents for obstetric anesthesia necessitates enhancement. Despite numerous trials in this area, evidence gaps persist for specific clinical queries in this filed. One potential approach to mitigate these challenges involves the endorsement of standardized guidance development methods and the synthesis of robust clinical evidence, aimed at diminishing inconsistencies in recommendations.

For peer review only

Strengths and limitations of this study

1. This systematic review evaluated the quality and consistency of guidance documents in obstetric anesthesia.
2. Not all guidance documents were developed with AGREE II method and the quality necessitates enhancement.
3. Only English and Chinese documents were included.

For peer review only

Introduction

Obstetric anesthesia refers to labor analgesia, cesarean delivery anesthesia, pain management during the procedure of labor and delivery, and addressing obstetric emergencies. Labor epidural analgesia is considered the gold standard of intrapartum analgesia[1], with approximately 10%-83% of women giving birth under neuraxial anesthesia[2]. The rate of cesarean delivery varied from 26% to 58%, exceeding the World Health Organization's recommendation range of 10% to 15%[3, 4]. Despite technological advancements, anesthesiologists still encounter significant challenges in safely administering anesthesia to pregnant patients. Aligning with the 2021 World Patient Safety Day theme 'Safe Maternal and Newborn Care', the primary goal remains safeguarding the health of both mother and fetus during anesthesia administration.

To enhance anesthetic care for obstetric patients, many clinical guidelines and consensus statements have been created by healthcare institutions and professionals. They cover aspects like peri-anesthetic assessment, and management for vaginal and cesarean deliveries. For example, the American Society of Anesthesiologists (ASA) released obstetric anesthesia guidelines in 1998, with updates in 2007 and 2016[5, 6]. In China, the consensus on obstetric anesthesia was initially published in 2007, with subsequent updated in 2017 and 2020[7, 8]. Additionally, China published two editions of guidelines for labor analgesia in 2016 [9] and 2020[10].

Despite the variety of documents, current guidelines and consensuses on obstetric anesthesia and analgesia provide inconsistent recommendations, such as whether require a platelet count, whether to order or require a blood type and screen or cross-

For peer review only

match [6]. Furthermore, guideline development may be influenced by the experience and composition of the guideline development groups. Low-quality guidance documents would pose risk to individual patients and communities, and impede the application of guideline recommendations in clinical practice[11]. Some guidelines were not written clearly, which may lead to clinician misunderstanding or not identifying key areas clearly, contributing to misjudged prioritizing of clinical actions [12]. Hence, it is essential to summarize and compare existing obstetric anesthesia guidelines or consensus statements to ensure the safety of both maternal and neonates during obstetric anesthesia. In this study, we aimed to evaluate the scope, content, and consistency of current guidance documents on obstetric anesthesia and analgesia. The quality of included guidelines and consensus statements were evaluated using the Appraisal of Guidelines for Research and Evaluation (AGREE) II instrument and presented as scores.

Methods

This study was developed based on the Preferred Reporting Items for Systematic Reviews and Meta Analyses for Protocols (PRISMA-P)[13]. The protocol of this study was registered prospectively at the International Platform of Registered Systematic Review and Meta-analysis Protocols, with a registration number of INPLASY202360088 (https://inplasy.com/inplasy-2023-6-0088/).

Literature search and selection criteria

We conducted a systematically search of PubMed and EMBASE from inception

For peer review only

to 8 June, 2023, employing a comprehensive search strategy (see online **Supplementary Table 1 and 2**) to identify guidelines and consensus recommendations for anesthetic management of pregnant during labor, nonoperative delivery, operative delivery, and selected aspects of perioperative monitoring, postpartum care, and analgesia. We also searched three academic databases for Chinese publications (the Chinese Biomedical Literature Database, Wanfang Data, and Chinese Medical Association Database; see online **Supplementary Table 3**) and six guideline databases from inception to 7 June, 2023, using search strategies tailored to different databases (online **Supplementary Table 4**). To ensure thoroughness, we conducted additional searched on Google and Google Scholar on 1 August, 2023. Additionally, we scanned the reference lists of the most relevant clinical practice guidelines and review articles to identify any additional relevant guidelines. We included the latest versions of all international and national/regional clinical practice guidelines and consensus statements for the obstetric anesthetic management published in English or Chinese. We defined clinical practice guidelines and consensus statements as documents providing recommendations for patient care, which are derived from a systematic review of existing evidence or from collective opinions of an expert panel[14]. The exclusion criteria were: (1) original investigation, study protocols, comments on existing guidelines or consensus, and conference abstracts or posters; (2) draft documents that are under development or not finalized; (3) previous documents replaced by updated versions from the same organization.

Two reviewers (L.H and D.X.C) independently screened all searched documents.

For peer review only

Full texts for potentially relevant guidelines or consensus statements were retrieved and examined for eligibility. Reasons for exclusion were provided for documents excluded during the full-text review (online **Supplementary Table 5**). Disagreements were resolved through discussion with a third reviewer (J.S).

Data extraction

The following data was extracted from each included document, including publication year, funding body, and evidence base. Additionally, the data encompassed recommendations pertaining to monitoring and management, postpartum care, and analgesia. Data were extracted by one investigator and cross-checked by another investigator. In the event of any discrepancies, consensus was reached through discussion and resolution.

Appraisal of guidance documents

The included documents were evaluated by four independent reviewers (L.H, N.H, L.J., and D.X.C) using the Appraisal of Guidelines for Research and Evaluation (AGREE) II tool[15]. AGREE II is a validated instrument widely used to assess the quality of clinical practice guidelines and consensus statements[16]. The tool consists of 23 items categorized into six domains: scope and purpose, stakeholder involvement, rigor of development, clarity of presentation, applicability, and editorial independence. Each item is rated on a seven-point scale, ranging from 1 (Strongly Disagree) to 7 (Strongly Agree), indicating the level of agreement. The method for each domain appraised is

For peer review only

present in Supplementary methods. In the current study, all appraisers completed an online training tutorial (Available: http://ww center-agree-ii-training-tools/ [Accessed September 1, 2023]) prior to commencing the review process to ensure standardization. Detailed instruction from the AGREE II User's Manual[15] and objective scoring criteria (online **Supplementary Table 6**) for each item were used to promote scoring consistency.

Synthesis of recommendations

We meticulously extracted recommendations pertaining to critical clinical queries from a comprehensive set to guidance and consensus documents. Recommendations were extracted by two investigators (L.H and N. H) and checked by another (X.L.X and D.X.C). To visually represent these recommendations, we utilized a color-coded grid to illustrate any inconsistencies (online **Summary of Key Recommendations**).

Statistical analysis

All data was analyzed using the SPSS V.25.0 software. Median and interquartile range (IQR) were computed for the domain scores. The inter-rater agreement was computed using the intraclass correlation coefficient (ICC) with a two-way random effects model for the total score was computed. The level of agreement (ICC) was categorized according to widely recognized thresholds: poor (<0.40), fair (0.40–0.59), good (0.60–0.74) or excellent (0.75–1.00) [17]. Following the approach described in a previously published article, a domain is considered to have superior quality if its score

For peer review only

exceeds 50%.

Results

Search results

We retrieved 6919 citations, with 6797 being excluded after screening titles and abstracts for not meeting eligibility criteria (**Figure 1**). We thoroughly assessed the remaining 122 citations and excluded 101 as they did not align with our defined criteria. Ultimately, we included thirteen guidelines [6, 18-29] and eight consensus statements [8-10, 30-34].

Characteristics of included guidelines and consensus statements

A summary of the characteristics of the eligible guidelines and consensus statements is presented in **Table 1**. Thirteen guidelines [6, 18-29] and eight consensus statements [8-10, 30-34] were identified, with publication dates ranging from 2004 to the present, involving 15 national or multi-international organizations predominantly comprising anesthesiologists or obstetricians and gynecologists from the United States, United Kingdom, Europe, and China. Seven documents[19, 20, 24, 26, 28, 29, 31] clearly stated being externally reviewed. Eight of these guidelines[6, 8, 18, 20, 21, 23, 26, 27] included procedures for updating in response to emerging evidence, while only six[6, 18, 20, 21, 24, 33] disclosed their funding body. The focus of these guidelines was primarily on the obstetric patients undergoing vaginal delivery or cesarean delivery (**Figure 2**), with eight documents[6, 8, 10, 20, 24-27] intended for use by anesthesiologists or other health care

For peer review only

providers. Additionally, ten documents[6, 19-21, 23-25, 28, 29, 34] conducted systematic literature reviews as part of their development process, which eleven documents [6, 18-20, 23, 24, 26, 28, 29, 33, 34] reported the level of evidence supporting recommendations and grading the strength of the guidance recommendations themselves.

Appraisal of guidelines and consensus statements

The standardized AGREE II domain scores for each guidance document are presented in **Figure 3** and provided in value as online **Supplementary Table 6**. Mean scores for each AGREE II item can be found in **Supplementary Table 9**. The ICC were above 0.90. Group discussion modified 365/2208 (16.53%) of individual scores. The overall quality of guidance documents varied both between documents across domains and within documents across domains. The ASA guideline published in 2016 [6] had the highest domain scores, with five domains scoring in the upper quartile. This was followed by the guidelines published by the Enhanced Recovery After Surgery Society Recommendations (ERAS) (part2)[29], American College of Obstetricians and Gynecologists (ACOG)[18], and Royal College of Anesthetists (RcoA)[26], all with four domains scoring above the upper quartile. In total, 81.0% (17 of 21) of the included documents were excellent quality. Guidance documents received the highest scores for the scope and purpose domain (median 76.4%, IQR 69.4%–79.2%) and the clarity of presentation domain (median 72.2%, IQR 69.1%–80.6%). However, they received the lowest scores for the applicability domain (median 20.8%, IQR 13.5%–27.1%) and the editorial independence domain (median 47.9%, IQR 0.0%–75.0%). No tendency of

For peer review only

improvement in the quality score over time was observed.

Synthesis of recommendations

Approaches to the peri-anesthetic evaluation and preparation

Fifteen documents[6, 8-10, 18-20, 23, 24, 26-28, 30, 32, 33]were included in the peri-anesthetic evaluation and preparation. Key recommendations are presented in **Supplementary Table 10**. Nine documents[6, 8-10, 18, 26, 27, 30, 32] provided recommendations for history and physical examination in pregnant individuals, with an emphasis on maternal health, anesthetic history, and relevant obstetric history. Of these, six documents[6, 8-10, 18, 27] specially recommended examining the patient's back when a neuraxial anesthetic was planned. Seven documents[6, 8, 10, 18, 27, 28, 30] highlighted the important of communication between obstetric providers, and anesthesiologists to recognize significant anesthetic or obstetric risk factors.

Six guidelines [6, 8, 9, 18, 23, 30] referenced in the literature emphasize the important of platelet count in predicting anesthesia-related complications. Three of these guidelines [6, 18, 23] recommended individualizing the platelet count based on a patient's medical history. Furthermore, five documents [6, 8, 9, 23, 30] discussed the necessity of blood type and screen or cross-match, with two of them[6, 23] suggesting that routine test is not essential for healthy and uncomplicated parturient (**Table 2**). Additionally, nine documents [6, 8-10, 18, 20, 24, 27, 30] indicated that fatal heart rate patterns should be monitored during the anesthetic process.

Eleven documents [6, 8, 19, 20, 23, 26-28, 30, 32, 33] addressed aspiration prevention during

delivery. Regarding fasting, five documents [6, 8, 28, 32, 33] suggested that clear liquids up to 2 hours before anesthesia induction, and six documents [6, 8, 28, 30, 32, 33] recommended a fasting period of 6-8 hours for solids. The use of antacids, H_2-receptor antagonists, and metoclopramide were also recommended for aspiration prophylaxis during cesarean delivery [6, 8, 19, 23, 26, 28, 32].

Five documents [9, 10, 18, 26, 27] discussed the use of heparin or low-molecular weight heparin during the peri- and post- anesthesia, and the ACOG [18] explicitly stated that prophylactic subcutaneous unfractionated heparin at a dosage of 5,000 units twice daily did not contraindicate neuraxial techniques, as long as catheter placement or removal more than 4-6 hours since last dose. In the case of intermediate-dose unfractionated heparin (7,500–10,000 units) more than 12 hours after the last dose or high-dose unfractionated heparin (total daily dose greater than 20,000 units) more than 24 hours after the last dose with activated partial thromboplastin time, it was deemed low risk to proceed with neuraxial anesthesia.

Approaches to the anesthetic care for labor and vaginal delivery

Eight documents [6, 9, 10, 18, 24, 26, 27, 32] addressed anesthetic care for labor and vaginal delivery (summarizing in **Supplementary Table 11**). Three documents [6, 9, 10] explicitly indicated that neuraxial analgesia could be administrated based on the level of discomfort the parturient, without limitation of cervical dilation. The ASA suggested providing labor neuraxial analgesia for parturient in early labor (i.e. cervical dilations of less than 5 cm dilation) [6]. These documents [6, 9, 10] also recommended to offer

For peer review only

neuraxial analgesia on an individualized basis. The ASA also provided recommendations on the administration of neuraxial techniques for patients attempting vaginal birth after previous cesarean delivery, as well as early placement of a neuraxial catheter [6].

There were three methods for neuraxial anesthetic: continuous infusion epidural analgesia (CIEA), combined spinal–epidural analgesia (CSEA), and single-injection spinal. Five documents [6, 9, 10, 18, 27] covered both CIEA and CSEA and recommended using local anesthetic, as well as adding opioids. Before a neuraxial procedure, the platelet count was required less than $70*10^9$/L to reduce the risk of hematoma [10, 18, 27]. The puncture location was recommended on L2-3 or L3-4 in China published consensus [9, 10]. Local anesthetic including bupivacaine, ropivacaine, and levobupivacaine were used with loading dose or maintenance doses. In China, published consensus [9, 10] recommended using 0.04% to 0.125% bupivacaine or 0.0625% to 0.15% ropivacaine to initiate analgesia and maintain with 0.05% to 0.125% bupivacaine or 0.0625% to 0.15% ropivacaine (**Table 2**). The Chinese Associations of Anesthesiology (CAA) consensus also provided recommendations for 0.04% to 0.125% levobupivacaine for loading dose and 0.05% to 0.125% levobupivacaine for maintenance [10]. These two documents suggested that opioids, including fentanyl or sufentanyl, could be injected with local anesthetic, with recommended loading dose of fentanyl was 0.5-2 $\mu g.ml^{-1}$ and 1-2 $\mu g.ml^{-1}$ for maintenance, and the loading dose of sufentanyl was 0.2-0.6 $\mu g.ml^{-1}$ with maintenance dose 0.3-0.6 $\mu g.ml^{-1}$ [9, 10]. The American Association of Nurse Anesthesiology (ANNA) guideline suggested using 0.0625% to 0.125% bupivacaine or

For peer review only

0.08% to 0.2% ropivacaine with or without opioids in CIEA [27]. In CSEA, opioids and local anesthetics could be delivered in spinal, with two recommended protocols [9, 10, 18, 27]: sufentanyl or ropivacaine used singly or in combination. The single dose of sufentanyl was 2.5-7 μg, single dose of ropivacaine was 2.3-3 mg, and dose of combined sufentanyl and ropivacaine was 2.5 μg/2.5 mg, respectively [9, 10]. Another protocol was that fentanyl and bupivacaine was used singly or with combination. The single dose of fentanyl was 15-25 μg, single dose of bupivacaine was 2.0-2.5 mg, and dose of combined fentanyl and bupivacaine was 2.5 μg/2.0 mg, respectively [9, 10]. For CSEA and single-injection spinal, both documents recommended using a small-gauge, non-cutting, pencil point needle to access the subarachnoid space [6, 27]. Additionally, five documents [6, 9, 10, 27, 32] provided recommendations for using patient-controlled epidural analgesia for labor analgesia.

Approaches to the anesthetic care for cesarean delivery

Nine documents[6, 8, 18, 20, 26, 27, 29, 30, 32] covered the anesthetic care including neuraxial anesthesia and general anesthesia for cesarean delivery (summarizing in **Supplementary Table 12**). The recommendations for neuraxial anesthesia including CIEA, single-injection spinal, and CSEA were consistent with those for labor and vaginal delivery. Seven of the documents [6, 8, 18, 20, 26, 27, 29, 32] covered CIEA, CSEA, and single spinal injection, with three documents [8, 18, 27] recommended the use of local anesthetic with or without opioids. A platelet count less than $70*10^9$/L was recommended to reduce the risk of hematoma[8, 18, 27]. Puncture locations were

For peer review only

recommended as follows: L1-2 or L2-3 for CIEA, L3-4 for single-injection in spinal, and L3-4 in single space for CSEA, while in double space, the puncture location was recommended in T12-L2 or L3-5 [8, 27]. To test the effect of neuraxial anesthesia, the recommended level of block was T6-4 [8]. Various local anesthetic, including lidocaine, bupivacaine, ropivacaine, and levobupivacaine were recommended for use in CIEA. In CAA 2020 [8], lidocaine was recommended with 1.5%-2% concentration, bupivacaine with 0.5%, ropivacaine with 0.5%-0.75% (**Table 2**). And 3% chloroprocaine was also recommended. In contrast, ANNA [27] recommended lower concentrations, with 0.0625-0.125% for bupivacaine and 0.08-0.2% for ropivacaine. For single injection spinal procedures, the recommended doses were 5-15 mg bupivacaine or 10-20 mg ropivacaine[8]. To prevent local anesthetic poisoning, it was recommended that anesthesiologist withdraw before injection and administer a test dose [8, 18]. It was also suggested to add epinephrine to local anesthetic solutions to delay absorption and increase duration of blockade through vasoconstriction of the blood vessels in the area [8, 18]. Furthermore, the use of a small-gauge, non-cutting, pencil point needle was recommended [6, 8, 27].

Additionally, eight documents[6, 8, 18, 23, 26, 27, 30, 32] provided recommendations on general anesthesia for cesarean delivery. According to the ASA[6], general anesthesia may be the most appropriate choice in cases of profound fatal bradycardia, ruptured uterus, severe hemorrhage, severe placental abruption, umbilical cord prolapse, and preterm footling breech. Other documents[18, 27] recommended the use of general anesthesia if neuraxial anesthesia placement was unsuccessful. Induction-delivery was

For peer review only

recommended within 10 minutes, with specific intravenous anesthetics. CAA[8] detailly recommended intravenous anesthetics including thiopental sodium (4-5 $mg.kg^{-1}$), propofol (1.5-2.5 $mg.kg^{-1}$), etomidate (0.2-0.3 $mg.kg^{-1}$), and ketamine (0.5-1 $mg.kg^{-1}$) as induction agents. Fentanyl (2-5 $\mu g.kg^{-1}$), sufentanyl (0.2-0.5 $\mu g.kg^{-1}$), and remifentanil (4 $ng.kg^{-1}$ for target) were commonly applied in the general anesthesia. Succinylcholine (1-1.5 $mg.kg^{-1}$) or rocuronium (0.6-1.2 $mg.kg^{-1}$) was used as a muscle relaxant.

Approaches to the monitoring of obstetric and anesthetic or intra- or postoperative pain management

To mitigate post-cesarean delivery pain, nine documents[18, 19, 21, 23, 25, 27, 32-34] provided recommendations for the management of intra- or postoperative pain (summarizing in **Supplementary Table 13**). Six of these documents [18, 21, 23, 27, 32-34] recommended the administration of intrathecal morphine 50-150 µg or epidural 1-3 mg for analgesia. Two documents [21, 23] also suggested the use of epidural diamorphine (2-3mg or 2.5-5 mg) for analgesia. ANNA [27] recommended hydromorphone 75-150 µg in intrathecal administration as well. When morphine was utilized in neuraxial block, four documents [23, 27, 30, 34] suggested monitoring respiratory frequency and modality. The Society of Obstetric Anesthesia and Perinatology (SOAP) [34] explicitly stated that using morphine less than 0.15 mg or epidural morphine less than 3 mg in healthy parturient would pose low risk, and recommended monitoring respiratory rate and sedation measurement every 2 hours for 12 hours postoperatively. However, in high-risk

For peer review only

circumstances such as cardiopulmonary or neurological comorbidity, obesity (body mass index ≥ 40 kg/m^2), obstructive sleep apnea, chronic opioid use or abuse, and hypertension, or who was administrated with magnesium, as well as high doses of morphine (morphine greater than 0.15 mg or epidural morphine greater than 3 mg), more intensive monitoring (respiratory rate and sedation assessments were monitored for every 1 h for first 12 h, every 2 h for 12–24 h) and additional modalities such as pulse oximetry and capnography were suggested (**Table 2**). To minimize systemic opioids utilization, eight documents[18, 19, 21, 23, 27, 32-34] recommended the administration of non-steroidal anti-inflammatory drug for postoperative pain. Two guidelines[21, 27] suggested a single dose of intravenous dexamethasone for analgesia. Local anesthetic infiltration for the wound after cesarean delivery was recommended by six documents[18, 19, 21, 27, 32, 33], and continuous local anesthetic infusion with the wound was recommended by three documents[18, 21, 32]. Nerve blocks, including transversus abdominis plane block and erector spinae plane block, for improving post-cesarean delivery pain was recommended by five documents [18, 19, 21, 32, 33]. Patient-controlled intravenous analgesia used for pain management after cesarean was recommended by four documents[18, 21, 23, 32]. Regarding obstetric neuraxial block, The Association of Anesthetists and the Obstetric Anesthetists' Association(AA and OAA) [22] stated that straight-leg raising was used for neurological monitoring, and if a maternal was unable to straight-leg raise at 4 h from the last dose of epidural/spinal local anesthetic, neurological injury required be investigated.

For peer review only

Approaches to the adverse events during obstetric anesthesia

Twelve documents[6, 8, 10, 18-20, 23, 27, 29, 31-33] provided recommendations for preventing the hypotension during obstetric anesthesia (summarizing in **Supplementary Table 14**). Eleven documents [6, 8, 10, 18-20, 23, 27, 29, 31, 32] advised on using 500-1000 ml volume expansion with crystals or colloids, as well as seven documents [6, 8, 10, 23, 27, 31, 32] recommended utilizing left uterine displacement once the maternal was positioned supine for surgery to reduce inferior vena cava compression. Vasopressor management during neuraxial anesthesia was recommended by ten documents [6, 8, 10, 18, 19, 23, 27, 31-33], with vasoactive drugs such as phenylephrine, metaraminol, noradrenalin and ephedrine. Phenylephrine with 20-50 $\mu g.min^{-1}$ or 0.25-0.5 $\mu g.\ kg^{-1}.\ min^{-1}$ was recommended by nine documents [6, 8, 10, 18, 19, 27, 31-33]. Ephedrine 2-15 mg intravenous was recommended by six documents [8, 10, 18, 19, 31, 32] (**Table 2**). The Association of Anesthetists of Great Britain and Ireland (AAGBI) [31] recommended that metaraminol with 0.5 $mg.ml^{-1}$could be used to mediate blood pressure. CAA [8] also recommended that noradrenalin 4-6 μg or 0.08 $\mu g.\ kg^{-1}.\ min^{-1}$ was useful for preventing hypotension. The target of blood pressure was to maintain systolic arterial pressure greater than 90% and avoid a decrease to < 80% baseline [23, 31]. For maternal with pre-eclampsia, two documents [8, 31] advised that vasopressor infusion should not be prophylactically used.

Five documents[10, 18, 25-27] provided recommendations for inadequate neuraxial anesthesia, two guidelines [25, 27] suggested assessing other causes of pain, evaluating the catheter site, and reassessing the sensory block level. For rescuing inadequate anesthesia, three documents[18, 25, 27] provided recommendations including

For peer review only

administrating a second neuraxial technique or extending the neuraxial technique, repeating boluses of fast-acting opioids (fentanyl 25-50 μg, alfentanil 250-500 μg) or ketamine (10 mg boluses), and if necessary, recommending general anesthesia.

Unintentional dural puncture would lead to post-dural puncture headache (PDPH). AANA[27] provided recommendations on using small, non-cutting spinal needle or placing intrathecal catheter for preventing PDPH. Treatment options for PDPH included epidural blood patch, adequate hydration, multimodal analgesia, caffeine, sumatriptan, gabapentin, and sphenopalatine ganglion block.

Approaches to the management of obstetric and anesthetic emergencies

Four documents [6, 8, 26, 27] provided recommendations for managing hemorrhagic emergencies, with three of them [6, 8, 27] consistently recommending the use of large bore intravenous catheters. The ASA [6] and ANNA [27] suggested the implementation of a massive transfusion protocol. Additionally, the ASA [6] recommended the availability of blood bank resource, fluid warmer, forced -air body warmer, and equipment for rapid infusion of intravenous fluids and blood products.

Seven documents[6, 8, 18, 20, 23, 26, 27] addressed airway emergency in obstetric anesthesia, with three of them [6, 18, 27] recommending the availability of personnel and equipment, such as laryngoscope with assorted blades and endotracheal tubes with stylets. In cases where tracheal intubation fails, the use of emergency procedures such as cricothyrotomy was advised. Access to oxygen source and suction source with tubing were also recommended[6, 23, 27]. The ASA provided specific recommendations for

For peer review only

medications to support blood pressure, muscle relaxation, and hypnosis [6].

Three documents [6, 26, 27] covered cardiopulmonary resuscitation, with both the ASA [6] and ANNA [27] recommending the availability of basic and advanced life-support equipment during cardiac arrest. It was also recommended to maintain uterine left displacement during cardiac arrest. If circulation was not restored within 4-5 min, two guidelines suggested performing a cesarean section [6, 26, 27].

Five documents[6, 18, 23, 26, 27] addressed emergency cesarean delivery, with the National Institute for Health and Care Excellence (NICE)[23] providing specific recommendations for the time of unplanned and emergency cesarean births when there is a threat to the life of the woman or fetus, within 30 minutes. The use of neuraxial anesthesia and general anesthesia was recommended for emergency cesarean delivery [18, 23].

Discussion

In this study, we systematically reviewed thirteen guidelines [6, 18-29] and eight consensus statements[8-10, 30-34]. The comparison of the content of the twenty-one documents showed that several documents were very comprehensive, covering many aspects of obstetric anesthesia [6, 8-10, 18, 20, 23, 26, 27, 29, 32, 33],while other documents may focus on the specific contents[19, 21, 22, 24, 25, 28, 30, 31, 34]. We found generally average methodological quality and some inconsistent recommendations from included documents covering the preoperative screening and management of obstetric anesthesia and analgesia. We summarized all key recommendations, and compared and visualized

For peer review only

the inconsistencies among them, providing a concise but informative overview for clinicians and researchers.

Comparison with existing research

Our study systematically incorporated both guidelines and consensus statements in the field of obstetric anesthesia and analgesia, and we observed varied performance across different AGREE II domains for both types of documents. The assessment of the guidelines using the AGREE II tool revealed that the documents assessed in our study excelled in the domains of scope and purpose (domain 1) and clarity of presentation (domain 4); but performed poorly in the domain of applicability (domain 5) and editorial independence (domain 6). This distribution of AGREE II domain scores has been observed in previous guideline appraisal studies, with documents scoring higher in the scope and purpose domain and the clarity of presentation domain, and lower in the applicability domain and the editorial independence domain. This pattern was not only observed in guidance documents for endocrinology diseases[35, 36], but also in documents for other clinical specialties[37, 38]. Despite generally low and varied scores in the applicability domain, guidance documents for obstetric anesthesia and analgesia performed notably worse compared to documents for other conditions[35, 37], indicating that improving the applicability of guidance in this field may be more challenging. One major barrier to achieving good applicability of guidance documents is the time and cost required to perform economic evaluations and pilot studies. A stable and long-term task force for guideline development is needed to conduct these evaluations and studies.

For peer review only

While forming such a task force may be difficult in some regions and countries, guidance documents should at least inform the audience about the need to consider these issues. Low scores in the editorial independence domain often resulted from a lack of detailed information on the influence of the funding body and conflicts of interest, with 42.9% of included documents in the current study failing to provide this information. It is essential to disclose the potential influence of the pharmaceutical industry on the synthesis of clinical guidance and promote transparency in financial declarations.

Clinical implications and future research

Current guidance and consensus documents uniformly endorse thorough perioperative evaluations and tailored anesthesia methodologies for delivery, including cesarean sections. Notwithstanding these overarching agreements, variances in specific recommendations persist, likely stemming from the variable quality of the documents, gaps in current evidence, or reliance on antiquated studies. It is imperative to recognize that numerous clinical considerations remain underrepresented in these guidance and consensus documents. Among these, the post-neuraxial anesthesia evaluation of nerve blocks warrants particular attention. This includes the necessity for continuous postoperative sensory monitoring, vigilant respiratory depression surveillance, and strategies for addressing inadequate analgesia.

In obstetric practice, neuraxial anesthesia is a cornerstone of pain management. However, it is not without risk. Atypical presentations of motor or sensory block, such

For peer review only

as unexpectedly dense or prolonged effects, may herald complications including unintended intrathecal placement or, in rare instances, neurological pathologies. Our review reveals that among the documents evaluated, only the AA and OAA[22] provided recommendations for neurological monitoring, specifically endorsing the straight-leg raise test. There is a conspicuous need for enhanced vigilance in post-procedure monitoring, particularly in the immediate post-partum period. Current clinical practice often overlooks the importance of neuraxial monitoring; a survey conducted in the UK highlighted that only 56% of units had a formal protocol for postoperative neurological assessment, with a few units deferring evaluation until after the first post-delivery day [39].

Our review critically examined respiratory depression monitoring in the context of neuraxial anesthesia, particularly when neuraxial opioids such as morphine, fentanyl, sufentanyl, and remifentanil are administered. The ASA guideline[6] recommended continuous monitored of ventilation, oxygenation, and consciousness in patients receiving neuraxial opioids. They further suggested intensified monitoring for those with a heightened risk of respiratory depression. Although the widespread endorsement of opioids in neuraxial anesthesia, it seemed that the monitoring of respiratory depression wasn't sufficiently highlight by these guidance documents.

Neuraxial anesthesia, while widely employed, does not always successful. The definition of 'inadequate' varies within the literature, contributing to this broad prevalence range. The prevalence of inadequate neuraxial anesthesia ranged from 10.2% - 30.3%[40]. Although there were several guidelines[10, 18, 25-27] provided some

For peer review only

recommendations on the management of inadequate neuraxial anesthesia. Patel *et al.* [41] revealed that only 23% of clinicians have received formal training in management of inadequate neuraxial anesthesia. Notably, the literature indices a significant gap in both the identification and management of inadequate neuraxial analgesia. Future initiatives must prioritize the development and dissemination of standardized education and training protocols to effectively recognized and manage inadequate neuraxial anesthesia.

Emergency cesarean section, often performed in cases with life-threatening conditions for the mother and fetus. Decision-to-delivery intervals was recommended within 30 minutes in category 1 cesarean birth when there was immediate threat to the life of the woman or fetus, and within 75 minutes in category 2 cesarean birth when there was maternal or fatal compromise which is not immediately life-threatening [23]. Nevertheless, studies found that decision-to-delivery intervals were frequently unmet [42, 43]. There is significant heterogeneity in how decision-to-delivery intervals are defined and measured [11]. A pressing concern for anesthesiologists is the selection of anesthesia type- neuraxial anesthesia versus general anesthesia-in these emergent scenarios, for which the guidelines lack specific, universally applicable recommendations.

Limitations

Firstly, our inclusion of only English and Chinese documents may have overlooked key guidance from non-English/Chinese speaking regions, though we

For peer review only

sought English versions where possible. Secondly, unconscious bias might have affected our subjective AGREE II ratings, warranting caution in their clinical application. The subjective nature of AGREE II scoring also challenges the reproducibility and comparison of different reviews. Lastly, certain topics in obstetric anesthesia, like epidural-related maternal fever, postpartum depression, and intra-partum hypothermia, were not covered.

Conclusions

The current obstetric anesthesia and analgesia guidelines require significant improvements in methodological quality. They show inconsistencies in recommendations, highlighting the need for standardized guideline development and high-quality evidence synthesis. Crucially, areas like neuraxial analgesia management and monitoring of postoperative neurological functions and intrapartum fever are underrepresented, indicating a gap in evidence-based guidelines and necessitating comprehensive research.

Data availability statement

All data relevant to the study are included in the article or uploaded as supplementary information.

Acknowledgement

All analyses were based on previously published guidelines or consensus, thus no ethical approval is required. Systematic review registration number:

For peer review only

INPLASY202360088; URL: https://inplasy.com/inplasy-2023-6-0088/.

Funding information

This study was funded, in part, by a grant from the Science and Technology Department of Sichuan Province (No. 2023NSFSC1570).

Conflict of interest statement

The authors report no conflict of interest.

Author contributions

Dong Xu Chen designed the study. Lu Huang and Dong Xu Chen performed the literature search and screening and extracted the data. Lu Huang, Na Hu, Ling Jian, Xing Long Xiong, Jing Shi and Dong Xu Chen conducted the data analyses. Lu Huang and Dong Xu Chen created the figures and tables and drafted the manuscript. All authors participated in the interpretation of results, and critically revised the manuscript. All authors approved the final version of the manuscript for publication.

Reference

For peer review only

1. Anim-Somuah M, Smyth RM, Cyna AM, Cuthbert A. Epidural versus non-epidural or no analgesia for pain management in labour. *Cochrane Database of Syst Rev* 2018;5:Cd000331. http://doi.org/10.1002/14651858.CD000331.pub4.

2. Seijmonsbergen-Schermers AE, van den Akker T, Rydahl E, *et al*. Variations in use of childbirth interventions in 13 high-income countries: A multinational cross-sectional study. *PLOS Med* 2020;17:e1003103. http://doi.org/10.1371/journal.pmed.1003103.

3. Qiao J, Wang Y, Li X, *et al*. A Lancet Commission on 70 years of women's reproductive, maternal, newborn, child, and adolescent health in China. *Lancet* 2021;397:2497-536. http://doi.org/10.1016/s0140-6736(20)32708-2.

4. Wise J. Alarming global rise in cesarean births, figures show. *BMJ* 2018;363:k4319. http://doi.org/10.1136/bmj.k4319.

5. Practice guidelines for obstetric anesthesia: an updated report by the American Society of Anesthesiologists Task Force on Obstetric Anesthesia. *Anesthesiology* 2007;106:843-63. http://doi.org/10.1097/01.anes.0000264744.63275.10.

6. Practice Guidelines for Obstetric Anesthesia: An Updated Report by the American Society of Anesthesiologists Task Force on Obstetric Anesthesia and the Society for Obstetric Anesthesia and Perinatology. *Anesthesiology* 2016;124:270-300. http://doi.org/10.1097/ALN.0000000000000935.

7. Chinese Society of Anesthesiology. Chapter 8, expert consensus on the obstetric anesthesia. Expert consensus and guidelines for Anesthetic Management in China (original document in Chinese). Beijing: People′s Medical Publishing House; 2017.

8. Chinese Society of Anesthesiology. Chapter 8, expert consensus on the obstetric anesthesia. Expert consensus and guidelines for Anesthetic Management in China (original document in Chinese). Beijing: People′s Medical Publishing House; 2020.

9. Group of Obstetric Anesthesia of Chinese Society of Anesthesiology. Expert consensus on labour analgesia (original document in Chinese). *J Clin Anesthesiol* 2016; 32: 816-8.

10. Chinese Society of Anesthesiology. Chapter 9, expert consensus on the labour analgesia. Expert consensus and guidelines for Anesthetic Management in China

For peer review only

(original document in Chinese). Beijing: People's Medical Publishing House; 2020.

11. Winther LP, Mitchell AU, MØLler AM. Inconsistencies in clinical guidelines for obstetric anaesthesia for cesarean section. *Acta Anaesthesiol Scand* 2012;57:141-49. http://doi.org/10.1111/aas.12004.

12. Woolf SH, Fau. GR, Hutchinson A, A. H, M. E, Grimshaw J. Clinical guidelines: potential benefits, limitations, and harms of clinical guidelines. *BMJ* 1999;318:527-30. https://doi.org/10.1136/bmj.318.7182.527.

13. Shamseer L, Moher D, Clarke M, *et al*. Preferred reporting items for systematic review and meta-analysis protocols (PRISMA-P) 2015: elaboration and explanation. *BMJ* 2015;350:g7647. https://doi.org/10.1136/bmj.g7647.

14. Robin Graham, Michelle Mancher, Dianne Miller Wolman, Sheldon Greenfield, Earl Steinberg, editors. Clinical Practice Guidelines We Can Trust. Washington (DC): National Academies Press (US) 2011.

15. Brouwers MC, Kho ME, Browman GP, *et al*. AGREE II: advancing guideline development, reporting, and evaluation in health care. *Prev Med* 2010;51:421-4. http://doi.org/10.1016/j.ypmed.2010.08.005.

16. Deng Y, Luo L, Hu Y, Fang K, Liu J. Clinical practice guidelines for the management of neuropathic pain: a systematic review. *BMC anesthesiol* 2016;16:12. http://doi.org/10.1186/s12871-015-0150-5.

17. Brouwers MC, Kho ME, Browman GP, *et al*. Development of the AGREE II, part 1: performance, usefulness and areas for improvement. *CMAJ* 2010;182:1045-52. http://doi.org/10.1503/cmaj.091714.

18. American College of Obstetricians and Gynecologists' Committee on Practice Bulletins—Obstetrics. ACOG Practice Bulletin No. 209: Obstetric Analgesia and Anesthesia. *Obstet Gynecol* 2019;133:e208-e25. http://doi.org/10.1097/aog.0000000000003132.

19. Macones GA, Caughey AB, Wood SL, *et al*. Guidelines for postoperative care in cesarean delivery: Enhanced Recovery After Surgery (ERAS) Society recommendations (part 3). Am J Obstet Gynecol 2019;221:247.e1-e9.

For peer review only

http://doi.org/10.1016/j.ajog.2019.04.012.

20. Maxwell C, Gaudet L, Cassir G, *et al*. Guideline No. 392-Pregnancy and Maternal Obesity Part 2: Team Planning for Delivery and Postpartum Care. *J Obstet Gynaecol Can* 2019;41:1660-75. http://doi.org/10.1016/j.jogc.2019.03.027.

21. Roofthooft E, Joshi GP, Rawal N, *et al*. PROSPECT guideline for elective cesarean section: updated systematic review and procedure-specific postoperative pain management recommendations. *Anaesthesia* 2020;76:665-80. http://doi.org/10.1111/anae.15339.

22. Yentis SM, Lucas DN, Brigante L, *et al*. Safety guideline: neurological monitoring associated with obstetric neuraxial block 2020. *Anaesthesia* 2020;75:913-19. http://doi.org/10.1111/anae.14993.

23. National Institute for Clinical Excellence. Cesarean Section. Published: 31 March 2021, Available at: https://wwwniceorguk/guidance/NG192 2021.

24. Mei-Dan E, Jain V, Melamed N, *et al*. Guideline No. 428: Management of Dichorionic Twin Pregnancies. *J Obstet Gynaecol Can* 2022;44:819-34.e1. http://doi.org/10.1016/j.jogc.2022.05.002.

25. Plaat F, Stanford SER, Lucas DN, *et al*. Prevention and management of intra-operative pain during cesarean section under neuraxial anaesthesia: a technical and interpersonal approach. *Anaesthesia* 2022;77:588-97. http://doi.org/10.1111/anae.15717.

26. Royal College of Anaesthetists. Guidelines for the Provision of Anaesthetic Services: Chapter 9 Guidelines for the Provision of Anaesthesia Services for an Obstetric Population 2023. Available at: https://www.rcoa.ac.uk/gpas/chapter-9.

27. American Association of Nurse Anesthesiology. Analgesia and Anesthesia for the Obstetric Patient. Available at: https://issuu.com/aanapublishing/docs/analgesia_and_anesthesia_for_the_obstetric_patient?fr=sN2ZlNTU2NDAxMjU.

28. Wilson RD, Caughey AB, Wood SL, *et al*. Guidelines for Antenatal and Preoperative care in Cesarean Delivery: Enhanced Recovery After Surgery Society

For peer review only

Recommendations (Part 1). *Am J Obstet Gynecol* 2018;219:523.e1-e15. http://doi.org/10.1016/j.ajog.2018.09.015.

29. Caughey AB, Wood SL, Macones GA, *et al*. Guidelines for intraoperative care in cesarean delivery: Enhanced Recovery After Surgery Society Recommendations (Part 2). *Am J Obstet Gynecol* 2018;219:533-44. http://doi.org/10.1016/j.ajog.2018.08.006.

30. Obstetrics Subgroup, Society of Obstetrics and Gynecology, Chinese Medical Association.. Expert consensus on cesarean delivery. *Zhonghua fu chan ke za zhi* 2014;49:721-24.

31. Kinsella SM, Carvalho B, Dyer RA, *et al*. International consensus statement on the management of hypotension with vasopressors during cesarean section under spinal anaesthesia. *Anaesthesia* 2017;73:71-92. http://doi.org/10.1111/anae.14080.

32. China Healthy Birth Science Association. Expert Consensus on Enhanced Recovery after Delivery. *Progress in Obstetrics and Gynecology* 2020;29:561-67.

33. Bollag L, Lim G, Sultan P, *et al*. Society for Obstetric Anesthesia and Perinatology: Consensus Statement and Recommendations for Enhanced Recovery After Cesarean. *Anesth Analg* 2021;132:1362-77. http://doi.org/10.1213/ane.0000000000005257.

34. Bauchat JR, Weiniger CF, Sultan P, *et al*. Society for Obstetric Anesthesia and Perinatology Consensus Statement: Monitoring Recommendations for Prevention and Detection of Respiratory Depression Associated With Administration of Neuraxial Morphine for Cesarean Delivery Analgesia. *Anesth Analg* 2019;129:458-74. http://doi.org/10.1213/ane.0000000000004195.

35. Wu CM, Wu AM, Young BK, *et al*. An Appraisal of Clinical Practice Guidelines for Diabetic Retinopathy. *Am J Med* 2016;31:370-5. http://doi.org/10.1177/1062860615574863.

36. Fang Y, Yao L, Sun J, *et al*. Appraisal of clinical practice guidelines on the management of hypothyroidism in pregnancy using the Appraisal of Guidelines for Research and Evaluation II instrument. *Endocrine* 2018;60:4-14. http://doi.org/10.1007/s12020-018-1535-2.

37. Gavriilidis P, Roberts KJ, Askari A, *et al*. Evaluation of the current guidelines for

For peer review only

resection of hepatocellular carcinoma using the Appraisal of Guidelines for Research and Evaluation II instrument. *J Hepatol* 2017;67:991-98. http://doi.org/10.1016/j.jhep.2017.06.028.

38. Li Q, Li X, Wang J, *et al*. Diagnosis and treatment for hyperuricemia and gout: a systematic review of clinical practice guidelines and consensus statements. *BMJ open* 2019;9:e026677. http://doi.org/10.1136/bmjopen-2018-026677.

39. Roderick E, Hoyle J, Yentis SM. A national survey of neurological monitoring practice after obstetric regional anaesthesia in the UK. *Anaesthesia* 2017;72:755-59. http://doi.org/10.1111/anae.13800.

40. Patel R, Kua J, Sharawi N, *et al*. Inadequate neuraxial anaesthesia in patients undergoing elective cesarean section: a systematic review. *Anaesthesia* 2022;77:598-604. http://doi.org/10.1111/anae.15657.

41. Patel R, Russell R, Plaat F, *et al*. Inadequate neuraxial anaesthesia during cesarean delivery: a survey of practitioners. *Int J Obstet Anesth* 2023;56:103905. http://doi.org/10.1016/j.ijoa.2023.103905.

42. Ayeni OM, Aboyeji AP, Ijaiya MA, *et al*. Determinants of the decision-to-delivery interval and the effect on perinatal outcome after emergency cesarean delivery: a cross-sectional study. *Malawi Med J* 2021;33:28-36. http://doi.org/ 10.4314/mmj.v33i1.5.

43. Kitaw TM, Limenh SK, Chekole FA, *et al*. Decision to delivery interval and associated factors for emergency cesarean section: a cross-sectional study. *BMC Pregnancy and Childbirth* 2021;21:224. http://doi.org/10.1186/s12884-021-03706-8.

For peer review only

Table 1. Characteristics of included guidelines and consensus statements.

Document	Issuing organization	Country	Funding body	Target population	Target audience	Guideline development group	Guideline review	Guideline update	Evidence base	LOE	SOR
**Guidelines**											
**ASA_2016** [6]	American Society of Anesthesiologists	US	Institutional	Labor, nonoperative delivery, operative delivery, and selected aspects of postpartum care and analgesia (not apply to patients undergoing surgery during pregnancy, gynecological patients, or parturient with chronic medical disease)	Anesthesiologists	Multi	NG	1999-2006-2016	CS and SLR	+	+
**ERAS_2018 (Part 1, 2, 3)** [19,20,28]	Enhanced Recovery After Surgery	Multination	NG	Cesarean delivery patients	NG	ERAS	External review	NG	SLR	+	+
**ACOG_2019** [18]	American College of Obstetricians and Gynecologists	US	Institutional	NG	NG	ACOG	NG	2002-2007-2013-2017-2019	NG	+	+
**SOGC_2019** [20]	Society of Obstetricians and Gynecologists of Canada	Canada	Institutional	Women with obesity who are pregnant or planning pregnancies	Health care providers	SOGC	External review	5 years	SLR	+	+
**SOGC_2022** [24]	Society of Obstetricians and Gynecologists of Canada	Canada	Institutional	Pregnant women with a dichorionic twin pregnancy	Health care providers	SOGC	External review	NG	SLR	+	+

For peer review only

**AA and OAA_2020** [22]	Association of Anesthetists and the Obstetric Anesthetists' Association	UK	None	Obstetric neuraxial block patients	NG	AA and OAA	NG	NG	NG	-	-
**PROSPECT_2020** [21]	Procedure-specific postoperative pain management working group	Europe	OAA and European Society of Regional Anesthesia and Pain Therapy	Cesarean delivery patients (not be applied to emergency or unplanned cesarean section or surgery performed under general anesthesia.)	NG	Multi	NG	2014-2020	SLR	-	-
**NICE_2021** [23]	National Institute for Health and Care Excellence	UK	NG	Cesarean delivery patients	NG	NICE	NG	2011-2014-2021	SLR	+	+
**OAA_2022** [25]	Obstetric Anesthetists' Association	UK	None	Cesarean delivery patients	Anesthetists	OAA	NG	NG	CS and SLR	-	-
**ANNA_2023** [27]	American Association of Nurse Anesthesiology	US	NG	Obstetric patients during labor and delivery and related procedures	CRNAs	AANA	NG	2008-2023	NG	-	-
**RCoA_2023** [26]	Royal College of Anesthetists	UK	NG	All pregnant women (antenatal anesthesia, labor, peripartum operative procedures, high dependency care on labor ward, obstetric procedures during pregnancy, e.g. cervical sutures, fatal surgery.)	Health care providers	RCoA	External review	Planned in 2024	NG	+	+

For peer review only

**Consensus statements**											
**OG-OG-CMA_2014** [30]	Obstetrics Group, Obstetrics and Gynecology Branch of Chinese Medical Association	China	NG	Cesarean delivery patients	NG	OG-OG-CMA	NG	NG	NG	-	-
**OG-A-CMA_2016** [9]	Obstetrics Group, Anesthesiology Branch of Chinese Medical Association	China	NG	Labor analgesia	NG	OG-A-CMA	NG	NG	NG	-	-
**AAGBI_2017** [31]	Association of Anesthetists of Great Britain and Ireland	Europe (Ireland)	None	Cesarean delivery patients	NG	AAGBI	External review	NG	NG	-	-
**SOAP_2019** [34]	Society for Obstetric Anesthesia and Perinatology	US	None	Cesarean delivery patients	NG	SOAP	NG	NG	SLR	+	+
**CHBSA_2020** [32]	China Healthy Birth Science Association	China	NG	All pregnant women	NG	CHBSA	NG	NG	NG	-	-
**CAA_2020** [8]	Chinese Association of Anesthesiology	China	NG	All pregnant women	Anesthesiologists	CAA	NG	2008-2017-2020	NG	-	-
**CAA_2020** [10]	Chinese Association of Anesthesiology	China	NG	Labor and vaginal delivery	Anesthesiologists	CAA	NG	NG	NG	-	-
**SOAP_2020** [33]	Society for Obstetric Anesthesia and Perinatology	US	National Institutes of Health	Cesarean delivery patients	NG	SOAP	NG	NG	NG	+	+

ASA, American Society of Anesthesiologists; ERAS, Enhanced Recovery After Surgery; ACOG, American College of Obstetricians and

For peer review only

Gynecologists; SOGC, Society of Obstetricians and Gynecologists of Canada; AA and OAA, Association of Anesthetists and the Obstetric Anesthetists' Association; PROSPECT, Procedure-specific postoperative pain management working group; NICE, National Institute for Health and Care Excellence; OAA, Obstetric Anesthetists' Association; ANNA, American Association of Nurse Anesthesiology; RcoA, Royal College of Anesthetists; OG-OG-CMA, Obstetrics Group, Obstetrics and Gynecology Branch of Chinese Medical Association; OG-A-CMA, Obstetrics Group, Anesthesiology Branch of Chinese Medical Association; AAGBI, Association of Anesthetists of Great Britain and Ireland; SOAP, Society for Obstetric Anesthesia and Perinatology; CHBSA, China Healthy Birth Science Association; CAA, Chinese Association of Anesthesiology. LOE, level of evidence; SOR, strength of recommendation; SLR, systematic literature review. NG, not given.

For peer review only

Table2. Some inconsistent recommendations summarized.

		The most frequently recommended content	A partially consistent recommendation	Contrary recommendation
**Approaches to the peri-anesthetic evaluation and preparation**				
**Routine intrapartum platelet count**		Agree	-	Individualized
**Routine blood type and screen**		Agree	-	Individualized
**Routine blood cross-match**		Agree	-	Individualized
**Approaches to the anesthetic care for labor and vaginal delivery**				
**Bupivacaine**	Continuous infusion epidural analgesia	0.04% to 0.125%	0.0625% to 0.125%	-
	Single-injection spinal	No guideline	2.0-2.5mg	-
**Ropivacaine**	Continuous infusion epidural analgesia	0.0625% to 0.15%	0.08% to 0.2%	-
	Single-injection spinal	No guideline	2.3-3 mg	-
**Fentanyl**	Continuous infusion epidural analgesia	0.5 to 2 μg.ml^{-1}	1 to 2 μg.ml^{-1}	-
**Sufentanyl**	Continuous infusion epidural analgesia	0.2 to 0.6 μg.ml^{-1}	0.4 to 0.6 μg.ml^{-1}	-
**Approaches to the anesthetic care for cesarean delivery**				
**Bupivacaine**	Continuous infusion epidural analgesia	0.50%	0.0625% to 0.125%	
	Single-injection spinal	No guideline	5-15 mg	-
**Ropivacaine**	Continuous infusion epidural analgesia	0.5% to 0.75%	0.08% to 0.2%	
	Single-injection spinal	No guideline	10-20 mg	-
**Approaches to the monitoring of obstetric and anesthetic or intra- or postoperative pain management**				
**High risk maternal respiratory monitoring**		No guideline	q1h clinical assessment for first 12 h or half hour for 2 hours	-
**Neuraxial adjuvant drugs in spinal anesthesia**	Morphine	50 to 150 μg	150 to 500 μg	-
**Neuraxial adjuvant drugs in epidural or**	Diamorphine	2 to 3 mg	2.5 to 5 mg	-

**combined spinal-epidural technique.**				
**Approaches to the adverse events during obstetric anesthesia**				
**Vasoactive drugs used for preventing hypotension**	Phenylephrine	20-50 µg or 0.5 µg. kg $^{-1}$. min $^{-1}$	20~50 µg or 0.25. kg $^{-1}$. Min $^{-1}$	-
	Metaraminol	No guideline	1-2 mg or 4 µg. kg $^{-1}$. min^{-1} or 0.5 mg.ml^{-1}	-
	Noradrenalin	4-6 µg or 0.08 µg. kg $^{-1}$. min^{-1}	-	-
	Ephedrine	2 to 15 mg iv	2 to 4 mg	

Note: It is paramount to emphasize that the above contradictory recommendations serve as a concise overview, without claiming comprehensiveness.

For peer review only

Figures Legend

Figure 1. Flow diagram for literature search.

CBM, Chinese Biomedical Literature Database; CMAD, Chinese Medical Association Database; NGC, National Guideline Clearinghouse; GIN, Guidelines International Network; NICE, National Institute for Health and Care Excellence; SIGN, Scottish Intercollegiate Guidelines Network; TRIP, Turning Research into Practice Database. AGREE, appraisal of guidelines for research and evaluation.

Figure 2. A summary of the main focus of the included document.

ASA, American Society of Anesthesiologists; ERAS, Enhanced Recovery After Surgery; ACOG, American College of Obstetricians and Gynecologists; SOGC, Society of Obstetricians and Gynecologists of Canada; AA and OAA, Association of Anesthetists and the Obstetric Anesthetists' Association; PROSPECT, Procedure-specific postoperative pain management working group; NICE, National Institute for Health and Care Excellence; OAA, Obstetric Anesthetists' Association; ANNA, American Association of Nurse Anesthesiology; RcoA, Royal College of Anesthetists; OG-OG-CMA, Obstetrics Group, Obstetrics and Gynecology Branch of Chinese Medical Association; OG-A-CMA, Obstetrics Group, Anesthesiology Branch of Chinese Medical Association; AAGBI, Association of Anesthetists of Great Britain and Ireland; SOAP, Society for Obstetric Anesthesia and Perinatology; CHBSA, China Healthy Birth Science Association; CAA, Chinese Association of Anesthesiology.

Figure 3. Standardized domain scores for each document.

ASA, American Society of Anesthesiologists; ERAS, Enhanced Recovery After Surgery; ACOG, American College of Obstetricians and

For peer review only

Gynecologists; SOGC, Society of Obstetricians and Gynecologists of Canada; AA and OAA, Association of Anesthetists and the Obstetric Anesthetists' Association; PROSPECT, Procedure-specific postoperative pain management working group; NICE, National Institute for Health and Care Excellence; OAA, Obstetric Anesthetists' Association; ANNA, American Association of Nurse Anesthesiology; RcoA, Royal College of Anesthetists; OG-OG-CMA, Obstetrics Group, Obstetrics and Gynecology Branch of Chinese Medical Association; OG-A-CMA, Obstetrics Group, Anesthesiology Branch of Chinese Medical Association; AAGBI, Association of Anesthetists of Great Britain and Ireland; SOAP, Society for Obstetric Anesthesia and Perinatology; CHBSA, China Healthy Birth Science Association; CAA, Chinese Association of Anesthesiology.

For peer review only

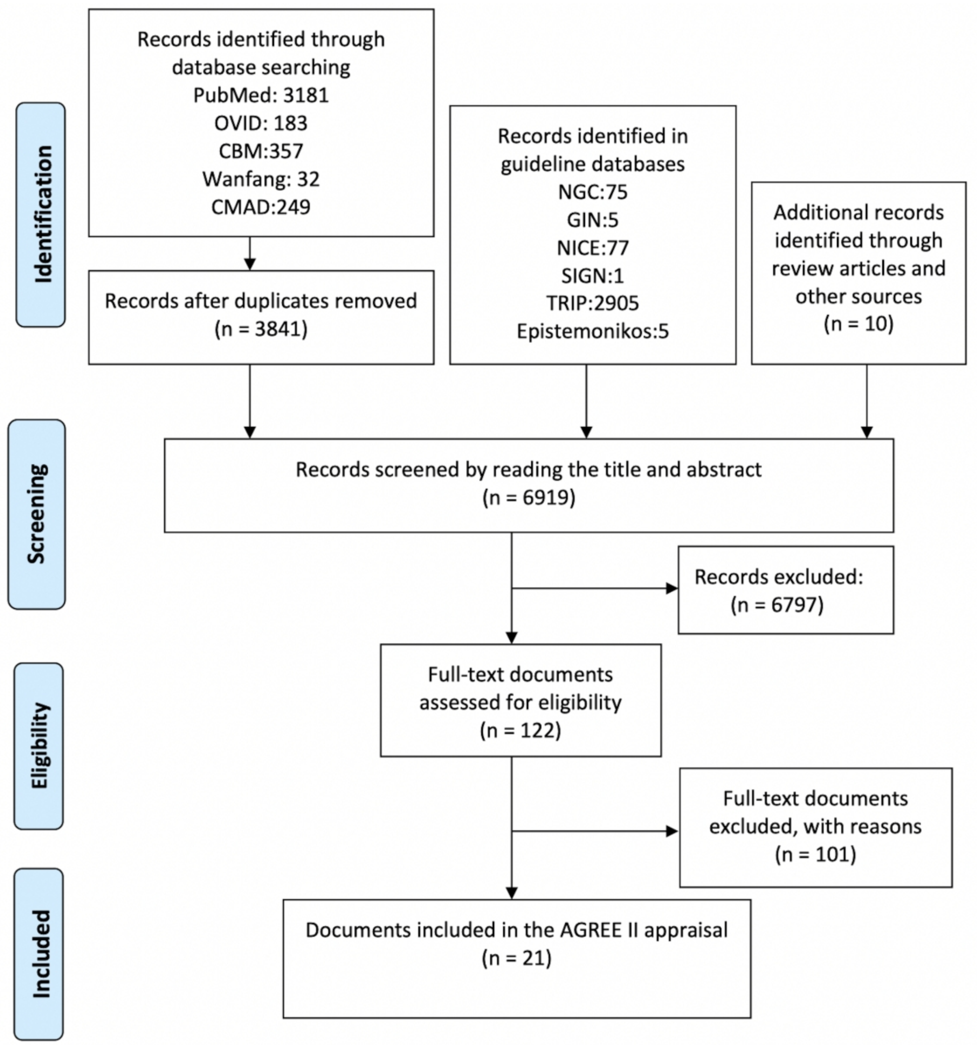

Flow diagram for literature search

233x244mm (300 x 300 DPI)

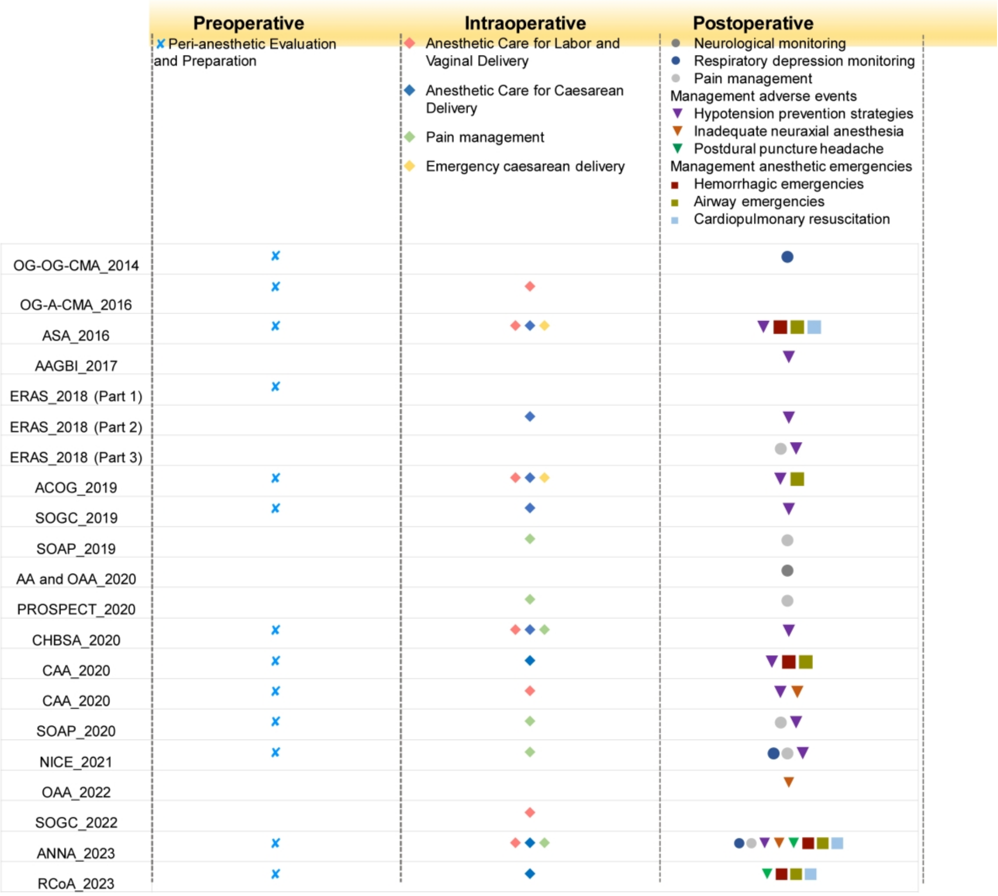

A summary of the main focus of the included document.

209x210mm (300 x 300 DPI)

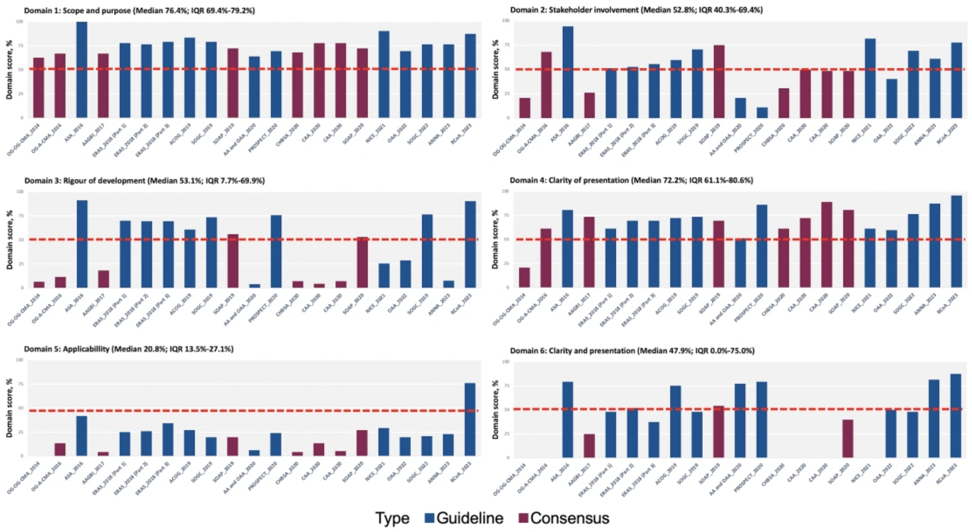

Standardized domain scores for each document.

437x235mm (300 x 300 DPI)

Supplementary Materials

Contents

Supplementary Table 1. Search strategy in PubMed.
Supplementary Table 2. Search strategy in EMBASE using the OVID interface.
Supplementary Table 3. Searches in Chinese databases.
Supplementary Table 4. Searches in guideline databases.
Supplementary Table 5. Excluded studies and reasons for exclusion.
Supplementary methods. How to calculate the scores.
Supplementary Table 6. Scores for each individual AGREE II domain items by each reviewer.
Supplementary Table 7. The intraclass correlation coefficient between reviewers for each individual AGREE II domain item.
Supplementary Table 8. Domain score for each included guidance document.
Supplementary Table 9. Mean scores across reviewers for the individual AGREE II domain items.
Supplementary Table 10. Summary of recommendations for the peri-anesthetic evaluation and preparation by included guidance document.
Supplementary Table 11. Summary of recommendations for the anesthetic care for labor and vaginal delivery by included guidance document.
Supplementary Table 12. Summary of recommendations for the anesthetic care for cesarean delivery by included guidance document.
Supplementary Table 13. Summary of recommendations for the monitoring and management of obstetric and anesthetic or intra- or postoperative pain management by included guidance document.
Supplementary Table 14. Summary of recommendations for the management of adverse events by included guidance document.
Supplementary Table 15. Summary of recommendations for the management of obstetric and anesthetic emergencies by included guidance document.

For peer review only

Supplementary Table 1. Search strategy in PubMed.

1	((((((cesarean[Title/Abstract]) OR (cesarean delivery[Title/Abstract])) OR (cesarean section[Title/Abstract])) OR (obstetric[Title/Abstract])) OR (obstetrical delivery[Title/Abstract])) OR (obstetric anesthesia[Title/Abstract])) OR (delivery[Title/Abstract])	633,790
2	((expert consensus[Title/Abstract]) OR (guideline[Title/Abstract])) OR (recommendation statement[Title/Abstract])	88,342
3	("2000/01/01"[Date - Publication]: "3000"[Date - Publication])	22,218,196
4	1 AND 2 AND 3	3,181

Date of literature search: June 6, 2023

For peer review only

For peer review only - http://bmjopen.bmj.com/site/about/guidelines.xhtml

Supplementary Table 2. Search strategy in EMBASE using the OVID interface.

1	cesarean.m_titl.	26,523
2	cesarean delivery.m_titl.	8,147
3	cesarean section.m_titl.	12,708
4	obstetric.m_titl.	23,566
5	obstetrical delivery.m_titl.	16
6	obstetric anesthesia.m_titl.	961
7	delivery.m_titl.	185,141
8	1 or 2 or 3 or 4 or 5 or 6 or 7	224,835
9	guideline.m_titl.	29,968
10	expert consensus.m_titl.	3,906
11	recommendation statement.m_titl.	538
26	9 or 10 or 11	34,373
21	8 and 12	183

Date of literature search: Embase <1974 to 2023 June 06>

For peer review only - http://bmjopen.bmj.com/site/about/guidelines.xhtml

Supplementary Table 3. Searches in Chinese databases.

**Chinese Biomedical Literature Database**			
[Original search term in Chinese] (cesarean OR obstetric OR obstetric anesthesia) AND (guideline OR consensus OR statement OR recommendation)	Results found	Full text screened	Included documents
	357	2	1
Date of literature search: June 8, 2023			
**Wanfang Data**			
[Original search term in Chinese] (cesarean OR obstetric OR obstetric anesthesia) AND (guideline OR consensus OR statement OR recommendation)	Results found	Full text screened	Included documents
	32	2	2
Date of literature search: June 8, 2023			
**Chinese Medical Association Database**			
[Original search term in Chinese] (cesarean OR obstetric OR obstetric anesthesia) AND (guideline OR consensus OR statement OR recommendation)	Results found	Full text screened	Included documents
	249	4	2
Date of literature search: June 8, 2023			

For peer review only - http://bmjopen.bmj.com/site/about/guidelines.xhtml

Supplementary Table 4. Searches in guideline databases.

Databases	Date of search	Search strategy	Results found	Full text screened	Included documents	URL
National Guideline Clearinghouse	2023/6/7	cesarean OR cesarean section OR cesarean delivery OR obstetric OR obstetrical delivery OR obstetric anesthesia OR delivery	75	1	0	www.guideline.gov
Guidelines International Network	2023/6/7	cesarean OR cesarean section OR cesarean delivery OR obstetric OR obstetrical delivery OR obstetric anesthesia OR delivery<br>Search mode: Guidelines	5	1	1	www.g-i-n.net
National Institute for Health and Care Excellence	2023/6/7	cesarean OR obstetric<br>filter type: guidance	77	3	1	www.nice.org.uk
Scottish Intercollegiate Guidelines Network	2023/6/7	Not applicable	1	0	0	www.sign.ac.uk/our-guidelines.html
Turning Research Into Practice Database	2023/6/7	cesarean OR cesarean section OR obstetric OR obstetric anesthesia<br>filter: Guidelines	2,905	11	6	www.tripdatabase.com
Epistemonikos database	2023/6/7	cesarean OR obstetric OR obstetric anesthesia<br>filter: Broad syntheses OR Structured summaries	5	1	1	www.epistemonikos.org

For peer review only - http://bmjopen.bmj.com/site/about/guidelines.xhtml

Supplementary Table 5. Excluded studies and reasons for exclusion.

Study	Reason for exclusion
Caughey AB, Sundaram V, Kaimal AJ, et al. Maternal and neonatal outcomes of elective induction of labor. Evidence Report/Technology Assessment No. 176. (Prepared by the Stanford University-UCSF Evidenced-based Practice Centre under contract No. 290-02-0017.) AHRQ Publication No. 09-E005. Rockville, MD.: Agency for Healthcare Research and Quality. March 2009.	Not providing specific recommendations for obstetric anesthesia
Association of Ontario Midwives. Vaginal birth after previous low-segment cesarean section. 2021; (Clinical Practice Guideline No. 14).	Not providing specific recommendations for obstetric anesthesia
Obstetric Care Consensus: Safe prevention of the primary cesarean delivery. March 2014	Not providing specific recommendations for obstetric anesthesia
Guidelines for neuraxial analgesia or anesthesia in obstetrics. American Society of Anesthesiologists, October 13, 2021.	Conference abstract
Optimal goals for anesthesia care in obstetrics. American Society of Anesthesiologists, October 13, 2021.	Conference abstract
NICE guideline: Preterm labor and birth. 10 June 2022	Not providing specific recommendations for obstetric anesthesia
Y Gu, YB Xu, CH, Wang, et al. The reading of the United States Society for Obstetric Anesthesia and Perinatology: consensus statement on the management of cardiac arrest in pregnancy. Journal of Chinese Physician 2017; 19(9): 1286-1293.	Secondary interpretation of foreign guidelines, not original guidelines
Interpretation of the American College of Obstetricians and Gynecologists: 2017 Obstetric Analgesia and Anesthesia Practice Guide. Journal of practical obstetrics and gynecology 2017; 33 (8):586-589.	Secondary interpretation of foreign guidelines, not original guidelines
Expert Consensus on Obstetric Anesthesia in China (2008) and (2017)	Replaced by updated versions from the same organization
Hawkins JL. et al. Practice guidelines for obstetric anesthesia: an update report by the American Society of Anesthesiologists Task Force on Obstetric Anesthesia.	Replaced by updated versions from the same organization

For peer review only - http://bmjopen.bmj.com/site/about/guidelines.xhtml

For peer review only

Anesthesiology 2007; 106: 843–63.	
Opderbecke HW. et al. Vereinbarung über die Zusammenarbeit in der operativen Gynäkologie und in der Geburtshilfe. Anästh Intensivmed 1996; 37: 414–8.	Not English
Opderbecke HW. et al. Airway management. Anästh Intensivmed 2004; 45: 302–6.	Not English
Gogarten W. et al. Regionalanästhesieverfahren in der Geburtshilfe. Anästh Intensivmed 2004; 45: 151–3.	Not English
T. Piepho1. et al. S1 guidelines on airway management. Guideline of the German Society of Anesthesiology and Intensive Care Medicine. Anesthetist. 2015 Dec;64 Supple 1:27-40.	Not providing specific recommendations for obstetric anesthesia
The National Institute of Clinical Excellence (NICE) guidelines for cesarean sections: implications for the Anesthetist (2004), (2011) and (2014)	Replaced by updated versions from the same organization
Intrapartum Care: Care of Healthy Women and Their Babies During Childbirth (2014)	Replaced by updated versions from the same organization
Osborne, L. et al. Evidence-based anesthesia: fever of unknown origin in parturient and neuraxial anesthesia.	Replaced by updated versions from the same organization
Kearsley, A. et al. The development of a local guideline for epidural insertion in obstetric patients with signs of maternal sepsis. Anesthesia 2015. 70: 56.	Abstract
Korneliussen, J. et al. Implementation of new guideline recommendations, using ultra-low dose intrathecal morphine, for post-cesarean analgesia and its impact on pain, nausea and pruritus. Acta anesthesiologic Scandinavica 2023. 67: 537-538.	Abstract
Nasser, L. et al. IN SITU SIMULATION TO FACILITATE INTRODUCTION OF A NEW OBSTETRIC RAPID SEQUENCE OF ANESTHESIA GUIDELINE. BMJ Simulation and Technology Enhanced Learning 2017. 3: A3.	Abstract
Cohen, J. et al. Implementation of the Association of Anesthetists safety guideline: neurological monitoring associated with obstetric neuraxial block (2020): improving patient safety in a district general hospital labor ward requires a multidisciplinary and multimodal approach. Anesthesia 2022. 77: 12.	Conference abstract
McGuire, R. et al. Changing of the weather? Introduction of an evidence-based guideline for obstetric general anesthesia after regional surveys and the use of simulation to facilitate guideline implementation at University	Conference abstract

For peer review only - http://bmjopen.bmj.com/site/about/guidelines.xhtml

Hospital South Manchester. Anesthesia 2017. 72: 87.	
Han, W. Y. et al. Anesthesia and perioperative management for cesarean section in patient with factor XI deficiency: a case report. Beijing da xue xue bao. Yi xue ban. Health sciences 2014. 46: 329-332.	Case report
McDonnell, N. J. et al. Acute magnesium toxicity in an obstetric patient undergoing general anesthesia for cesarean delivery. International journal of obstetric anesthesia 2010. 19: 226-231.	Case report
Oji-Zurmeyer, J. et al. National survey of obstetric anesthesia clinical practices in the republic of Austria. International journal of obstetric anesthesia 2019. 39: 95-98.	Primary study
Terblanche, N. C. et al. A survey of Anesthetists on uterotonic usage practices for elective cesarean section in Australia and New Zealand. Anesthesia and intensive care 2021. 49: 440-447.	Primary study
Terfasa, E. A. et al. Obstetric analgesia utilization in labor pain management and associated factors among obstetric care providers in the West Shewa Zone, Central Ethiopia. SAGE open medicine 2022. 10: 20503121221088705.	Primary study
Thuillier, C. et al. Impact of recommended changes in labor management for prevention of the primary cesarean delivery. American journal of obstetrics and gynecology 2018. 218: 341.e341-341.e349.	Primary study
Cobb, B. et al. Do obstetric Anesthesiologists provide more guideline-concordant care for patients undergoing cesarean delivery? Anesthesia and analgesia 2018. 126: 404-408.	Primary study
Fiedler, A. et al. Adverse effect of delayed pushing on postpartum blood loss in nulliparous women with epidural analgesia. International journal of gynecology and obstetrics: the official organ of the International Federation of Gynecology and Obstetrics 2020. 150: 92-97.	Primary study
Gunka, V. et al. Determination of ChloraPrep® drying time before neuraxial anesthesia in elective cesarean delivery. A prospective observational study. International journal of obstetric anesthesia 2019. 38: 19-24.	Primary study
Kinsella, S. M. et al. Category-1 cesarean section: a survey of anesthetic and peri-operative management in the UK. Anesthesia 2010. 65: 362-368.	Primary study
Lee, E. K. et al. Investigating a Needle-Based Epidural Procedure in Obstetric Anesthesia. AMIA ... Annual Symposium proceedings. AMIA Symposium 2018. 2018: 720-729.	Primary study
Nasrallah, F. K. et al. The 30-minute decision-to-incision interval for emergency cesarean delivery: fact or fiction? American journal of perinatology 2004. 21: 63-68.	Primary study
Ward, C. et al. Does the presence of epidural analgesia reduce the risk of neonatal sepsis in the setting of an	Primary study

For peer review only - http://bmjopen.bmj.com/site/about/guidelines.xhtml

intrapartum fever? The journal of maternal-fetal & neonatal medicine: the official journal of the European Association of Perinatal Medicine, the Federation of Asia and Oceania Perinatal Societies, the International Society of Perinatal Obstet 2022. 35: 2110-2115.	
Jejaw, M. et al. Comprehensive emergency management of obstetric and newborn care program implementation at University of Gondar Comprehensive Specialized Hospital, Northwest Ethiopia, 2021: an evaluation study. Reproductive health 2023. 20: 76.	Primary study
Ji, H. et al. Factors contributing to the rapid rise of cesarean section: a prospective study of primiparous Chinese women in Shanghai. BMJ open 2015. 5: e008994.	Primary study
Johri, M. et al. A cluster-randomized trial to reduce cesarean delivery rates in Quebec: cost-effectiveness analysis. BMC medicine 2017. 15: 96.	Primary study
McDougall, A. R. A. et al. Expert consensus on novel medicines to prevent preterm birth and manage preterm labor: Target product profiles. BJOG: an international journal of obstetrics and gynecology 2022.	Not providing specific recommendations for obstetric anesthesia
Sultan, P. et al. Expert Consensus Regarding Core Outcomes for Enhanced Recovery after Cesarean Delivery Studies: A Delphi Study. Anesthesiology 2022. 137: 201-211.	Not providing specific recommendations for obstetric anesthesia
Bremerich, D. et al. S1 Guideline: Obstetric analgesia and anesthesia. Anesthesiology und Intensivmedizin 2020. 61: S300-S340.	Not English
Lavery, J. P. et al. Is the obstetric guideline of 30 minutes from decision to incision for Cesarean delivery clinically significant? Journal of healthcare risk management: the journal of the American Society for Healthcare Risk Management 1999. 19: 11-20.	Pre 2000
Santerre, R. E. The effect of the ACOG guideline on vaginal births after cesarean. Medical care research and review : MCRR 1996. 53: 315-329.	Pre 2000
Morris, J. M. et al. Protocol for the immediate delivery versus expectant care of women with preterm prelabor rupture of the membranes close to term (PPROMT) Trial [ISRCTN44485060]. BMC pregnancy and childbirth 2006. 6: 9.	Protocol
Gochnour, G. et al. The UTAH VBAC Study. Maternal and child health journal 2005. 9: 181-188.	Review
King, V. J. et al. Clinical practice guideline executive summary: Labor after cesarean/planned vaginal birth	Review

For peer review only - http://bmjopen.bmj.com/site/about/guidelines.xhtml

after cesarean. Annals of family medicine 2015. 13: 80-81.	
Keag, O. E. et al. Long-term risks and benefits associated with cesarean delivery for mother, baby, and subsequent pregnancies: Systematic review and meta-analysis. PLoS medicine 2018. 15: e1002494.	Review
Gulbransen, K. et al. Scoping Review of Best Practice Guidelines for Care in the Labor and Birth Setting of Pregnant Women Who Use Methamphetamines. Journal of obstetric, gynecologic, and neonatal nursing: JOGNN 2022. 51: 141-152.	Review
Hocaloski, S. et al. Perinatal Care for Women with Spinal Cord Injuries: A Collaborative Workshop for Consensus on Care in Canada. Topics in spinal cord injury rehabilitation 2017. 23: 386-396.	Review
Hong, J. et al. Comparison of Maternal Labor-Related Complications and Neonatal Outcomes Following Elective Induction of Labor at 39 Weeks of Gestation vs Expectant Management: A Systematic Review and Meta-analysis. JAMA network open 2023. 6: e2313162.	Review
Indermuhle, P. et al. Use of Scheduled Nonopioid Analgesia to Decrease Inpatient Opioid Consumption After Scheduled Cesarean Birth. Nursing for women's health 2022. 26: 344-352.	Review
James, B. et al. Obstetric general anesthetic safety checklist: Guideline development through team simulation. International journal of obstetric anesthesia 2015. 24: S24.	Review
Kuehn, B. M. Obstetrics group relaxes guideline for trial of labor after cesarean delivery. Jama 2010. 304: 951-952.	Review
Lagro-Janssen, T. Pain management during labor--an informed choice. Nederlands tijdschrift voor geneeskunde 2009. 153: A742.	Review
Mash, R. et al. Alternative mechanisms for delivery of medication in South Africa: A scoping review. South African family practice : official journal of the South African Academy of Family Practice/Primary Care 2021. 63: e1-e8.	Review
Melman, S. et al. Identification of barriers and facilitators for optimal cesarean section care: perspective of professionals. BMC pregnancy and childbirth 2017. 17: 230.	Review
Morris, S. et al. Breech presentation management: A critical review of leading clinical practice guidelines. Women and birth : journal of the Australian College of Midwives 2022. 35: e233-e242.	Review
Mutlu, S. et al. Evaluation of pregnancy, delivery, and postpartum effectiveness of maternity school trainings organized based on the guideline of ministry of health in Turkey: A comparative study. International journal of	Review

For peer review only - http://bmjopen.bmj.com/site/about/guidelines.xhtml

preventive medicine 2021. 12: 173.	
Heslehurst, N. et al. GestationaL Obesity Weight management: Implementation of National Guidelines (GLOWING): a pilot cluster randomized controlled trial of a guideline implementation intervention for the management of maternal obesity by midwives. Pilot and feasibility studies 2018. 4: 47.	Review
Lyerly, A. D. et al. Toward an ethically responsible approach to vaginal birth after cesarean. Seminars in perinatology 2010. 34: 337-344.	Review
Nasser, L. et al. Farewell thiopentone? Northwest deanery surveys of obstetric general anesthesia highlight increasing popularity of induction with propofol. Would a region-wide guideline improve training and safety? Anesthesia 2017. 72: 94.	Review
Neuhaus, S. et al. "Why mothers die". Learning from the analysis of anesthesia-related maternal deaths (1985-2013). Der Anesthetist 2016. 65: 281-294.	Review
Panda, S. et al. Review of cesarean section on maternal request in a tertiary care institute; scenario in developing country. Kathmandu University medical journal (KUMJ) 2013. 11: 349-354.	Review
Reale, S. C. et al. The Most Influential Publications in Obstetric Anesthesiology, 1998-2017: Utilizing the Delphi Method for Expert Consensus. Anesthesia and analgesia 2020. 131: 239-244.	Review
Roderick, E. et al. A national survey of neurological monitoring practice after obstetric regional anesthesia in the UK. Anesthesia 2017. 72: 755-759.	Review
Rungreungvanich, M. et al. An analysis of intraoperative recall of awareness in Thai Anesthesia Incidents Study (THAI Study). Journal of the Medical Association of Thailand 2005. 88 Supple 7: S95-101.	Review
Sakoda, A. et al. Review of 197 cases of urgent cesarean section performed in 2010 using NICE classification. Masui. The Japanese journal of Anesthesiology 2014. 63: 1339-1343.	Review
Sommerness, S. A. et al. The Perinatal Birth Environment: Communication Strategies and Processes for Adherence to a Standardized Guideline in Women Undergoing Second-Stage Labor With Epidural Anesthesia. The Journal of perinatal & neonatal nursing 2017. 31: 41-50.	Review
Terui, K. Obstetric anesthesia: from basics to recent advances in neonatal resuscitation: from Apgar score to NCPR program. Masui. The Japanese journal of Anesthesiology 2010. 59: 293-302.	Review
Van Gorp, S. F. J. et al. Do we need a national guideline for obstetric analgesia in the Netherlands? Evaluation of a questionnaire among dutch Anesthetists. Nederlands Tijdschrift voor Anesthesiology 2005. 18: 100-103.	Review

For peer review only - http://bmjopen.bmj.com/site/about/guidelines.xhtml

Williamson, R. M. et al. Availability of lipid emulsion in obstetric anesthesia in the UK: a national questionnaire survey. Anesthesia 2008. 63: 385-388.	Review
Winther, L. P. et al. Failed intubation and subsequent airway management during Cesarean section in general anesthesia. Ugeskrift for laeger 2010. 172: 1914-1916.	Review
Friedman, C. L. et al. Resident Attitudes Towards Cesarean Delivery in Canadian Obstetrics and Gynecology Residency Programs. Journal of obstetrics and gynecology. JOGC 2020. 42: 16-24.	Review
Bagou, G. et al. Guidelines for the management of urgent obstetric situations in emergency medicine, 2022. Anesthesia, critical care & pain medicine 2022. 41: 101127.	Not providing specific recommendations for obstetric anesthesia
Crawford, C. New guideline covers labor, vaginal birth after cesarean. Annals of family medicine 2015. 13: 186-187.	Not providing specific recommendations for obstetric anesthesia
Elammary, M. N. et al. Middle eastern college of obstetricians and gynecologists (MCOG) practice guidelines: Role of prediction models in management of trial of labor after cesarean section. Practice guideline no. 05-O-22. Journal of gynecology obstetrics and human reproduction 2023. 52: 102598.	Not providing specific recommendations for obstetric anesthesia
Fischer, T. et al. From the OEGGG - S3 Guideline "Vaginal delivery at term": The position of the Austrian Society of Gynecology and Obstetrics (OEGGG). Geburtshilfe und Frauenheilkunde 2021. 81: 857-861.	Not providing specific recommendations for obstetric anesthesia
Gagnon, R. et al. SOGC CLINICAL PRACTICE GUIDELINE: guidelines for the management of vasa previa. International journal of gynecology and obstetrics: the official organ of the International Federation of Gynecology and Obstetrics 2010. 108: 85-89.	Not providing specific recommendations for obstetric anesthesia
Hey, J. Clinical practice guideline on vaginal delivery of breech presentation. Journal of obstetrics and gynecology Canada. 2009. 31: 1123.	Not providing specific recommendations for obstetric anesthesia
Jain, V. et al. Guideline No. 402: Diagnosis and Management of Placenta Previa. Journal of obstetrics and gynecology Canada. 2020. 42: 906-917.e901.	Not providing specific recommendations for obstetric anesthesia
Jain, V. et al. Guidelines for the Management of a Pregnant Trauma Patient. Journal of obstetrics and	Not providing specific

For peer review only - http://bmjopen.bmj.com/site/about/guidelines.xhtml

gynecology Canada. 2015. 37: 553-574.	recommendations for obstetric anesthesia
Jain, V. et al. Guideline No. 439: Diagnosis and Management of Vasa Previa. Journal of obstetrics and gynecology Canada. 2023.	Not providing specific recommendations for obstetric anesthesia
Ladhani, N. N. N. et al. No. 347-Obstetric Management at Borderline Viability. Journal of obstetrics and gynecology Canada. 2017. 39: 781-791.	Not providing specific recommendations for obstetric anesthesia
Lalonde, A. B. Vaginal breech delivery guideline: the time has come. Journal of obstetrics and gynecology Canada. 2009. 31: 483-486.	Not providing specific recommendations for obstetric anesthesia
Marte, K. et al. Reduction in the Cesarean Delivery Rate after Obstetric Care Consensus Guideline Implementation. Obstetrics and gynecology 2016. 128: 1445.	Not providing specific recommendations for obstetric anesthesia
Maxwell, C. et al. SOGC Clinical Practice Guideline. Management guidelines for obstetric patients and neonates born to mothers with suspected or probable severe acute respiratory syndrome (SARS). No. 225, April 2009. International journal of gynecology and obstetrics: the official organ of the International Federation of Gynecology and Obstetrics 2009. 107: 82-86.	Not providing specific recommendations for obstetric anesthesia
Nelson, G. et al. Guideline for perioperative obstetrical care highlights evidence gap related to timing of urinary catheter removal after elective cesarean delivery. American journal of obstetrics and gynecology 2020. 222: 635.	Not providing specific recommendations for obstetric anesthesia
Oliver, E. et al. Comparison of international guidelines on the management of twin pregnancy. European journal of obstetrics, gynecology, and reproductive biology 2023. 285: 97-104.	Not providing specific recommendations for obstetric anesthesia
Podrasky, A. E. et al. ACR appropriateness Criteria® second and third trimester bleeding. Ultrasound quarterly 2013. 29: 293-301.	Not providing specific recommendations for obstetric anesthesia
Robinson, D. et al. Guideline No. 431: Postpartum Hemorrhage and Hemorrhagic Shock. Journal of obstetrics	Not providing specific

For peer review only - http://bmjopen.bmj.com/site/about/guidelines.xhtml

and gynecology Canada. 2022. 44: 1293-1310.e1291.	recommendations for obstetric anesthesia
Scott-Warren, V. et al. Development of a fibrinogen concentrate guideline for use in major obstetric Hemorrhage. Transfusion Medicine 2016. 26: 30.	Not providing specific recommendations for obstetric anesthesia
Stafford, I. A. et al. Association between Maternal Obesity Class, Adherence to Labor Guidelines, and Perinatal Outcomes. AJP reports 2021. 11: e105-e112.	Not providing specific recommendations for obstetric anesthesia
Triebwasser, J. et al. Effect of Single-Unit Transfusion Guideline on Red Blood Cell Transfusion for Obstetric Patients. Obstetrics and gynecology 2023. 141: 59S.	Not providing specific recommendations for obstetric anesthesia
Zhu, L. et al. The Chinese guideline for prevention of pelvic and abdominal adhesions after obstetric and gynecologic surgery (2023 edition). Zhonghua fu chan ke za zhi 2023. 58: 161-169.	Not providing specific recommendations for obstetric anesthesia

Reason for exclusion: Review; Primary study; Editorial; Conference abstract; Not providing specific recommendations for obstetric anesthesia; Multimedia section; Replaced by updated versions from the same organization; Conference abstract; Comment letter; Technology appraisal; Book; Not released by a professional association.

For peer review only - http://bmjopen.bmj.com/site/about/guidelines.xhtml

For peer review only

Supplementary methods. How to calculate the scores.

The reviewers individually assessed each item using a Likert scale, and the scores were combined to calculate the domain scores. The total score for each domain was calculated by summing the scores of all the items within that domain for each reviewer. This total score was then standardized as a percentage of the maximum possible score for that specific domain. The calculation method is as follows:

$$\frac{Obtained\ score - Minimum\ possible\ score}{Maximum\ possible\ score - Minimum\ possible\ score} * 100\%$$

The minimum possible score is calculated as: (number of questions) × (number of reviewers) × 1. The maximum possible score is calculated as: (number of questions) × (number of reviewers) × 7.

Five guidance documents were selected for the pilot scoring phase, and discussions were held to clarify scoring criteria. After completing scoring for all included documents, a meeting was held to discuss items with scores differing by more than one point. Reviewers were then given the opportunity to revise their scores or maintain their original scores, with all original and revised scores, along with reasons for modifying scores, meticulously recorded for quality control purposes.

Supplementary Table 6. Scores for each individual AGREE II domain items by each reviewer.

For peer review only - http://bmjopen.bmj.com/site/about/guidelines.xhtml

Document	Domain 1			Domain 2			Domain 3								Domain 4			Domain 5				Domain 6	
**Item**	**1**	**2**	**3**	**4**	**5**	**6**	**7**	**8**	**9**	**10**	**11**	**12**	**13**	**14**	**15**	**16**	**17**	**18**	**19**	**20**	**21**	**22**	**23**
OG-OG-CMA_2014																							
Rev-1	4	4	7	2	1	3	1	1	1	1	3	1	1	1	5	1	1	1	1	1	1	1	1
Rev-2	4	3	7	2	3	2	1	1	1	1	3	1	1	1	5	1	1	1	1	1	1	1	1
Rev-3	4	3	7	3	3	2	1	1	1	1	4	1	1	1	5	1	1	1	1	1	1	1	1
Rev-4	3	4	7	2	1	3	1	1	1	1	3	1	1	1	4	1	1	1	1	1	1	1	1
OG-A-CMA_2016																							
Rev-1	5	6	5	6	6	3	1	1	1	1	6	1	1	1	5	6	3	1	5	1	1	1	1
Rev-2	6	4	4	6	6	4	1	1	1	1	6	1	1	1	6	6	3	1	4	1	1	1	1
Rev-3	5	6	5	6	6	3	1	1	1	1	5	1	1	1	5	6	3	1	4	1	1	1	1
Rev-4	5	4	5	6	6	3	1	1	1	1	6	1	1	1	5	5	3	1	4	1	1	1	1
ASA_2016																							
Rev-1	7	7	7	7	6	7	7	7	6	7	7	6	6	6	6	6	6	6	6	1	1	6	6
Rev-2	7	7	7	7	7	7	7	6	6	7	7	6	7	6	6	6	6	6	6	1	1	6	6
Rev-3	7	7	7	6	6	7	7	7	6	7	6	6	6	6	6	6	5	6	6	1	1	5	6
Rev-4	7	7	7	7	7	6	7	6	6	7	7	6	7	6	6	5	6	6	6	1	1	5	6
AAGBI_2017																							
Rev-1	5	5	5	6	1	1	2	2	2	4	2	1	1	1	6	5	5	2	1	1	1	2	3
Rev-2	5	5	5	6	1	1	2	2	2	4	2	1	1	1	6	5	5	2	1	1	1	2	3
Rev-3	4	5	5	5	1	1	3	2	2	5	2	1	1	1	5	6	5	2	1	1	1	3	2
Rev-4	5	5	6	6	1	1	2	3	2	4	3	1	1	1	6	5	6	2	1	1	1	2	3
ERAS_2018 (Part 1)																							
Rev-1	6	6	6	5	4	4	6	6	6	6	6	6	4	3	5	5	5	2	5	1	2	2	6
Rev-2	5	6	5	5	4	3	6	6	5	6	6	5	3	3	5	5	4	2	5	1	2	3	5
Rev-3	5	6	6	5	4	3	6	5	6	5	6	6	4	3	5	3	5	2	5	1	2	2	6
Rev-4	6	5	6	5	3	4	6	6	6	6	5	6	3	3	6	3	5	2	5	1	2	2	5

For peer review only - http://bmjopen.bmj.com/site/about/guidelines.xhtml

ERAS_2018 (Part 2)																							
Rev-1	6	6	6	5	4	4	6	6	6	6	6	6	4	3	5	5	5	2	5	1	2	2	6
Rev-2	6	6	4	5	3	4	5	6	4	6	5	6	4	3	5	6	5	2	4	1	2	3	6
Rev-3	5	5	6	5	4	3	5	6	5	6	6	5	4	3	5	5	6	2	5	1	2	2	5
Rev-4	6	6	5	5	4	4	6	6	5	6	6	6	4	3	4	6	5	2	6	1	3	3	6
ERAS_2018 (Part 3)																							
Rev-1	6	6	6	5	4	4	6	6	6	6	6	6	4	3	5	5	5	3	5	1	2	1	6
Rev-2	6	6	5	3	4	5	6	6	5	6	5	6	3	3	5	4	5	4	5	1	3	1	5
Rev-3	5	6	6	5	4	4	5	6	6	5	6	5	4	3	6	5	6	3	6	1	2	1	6
Rev-4	6	5	6	6	4	4	6	5	6	6	6	6	3	3	5	6	5	4	5	1	3	1	5
ACOG_2019																							
Rev-1	7	6	5	5	4	5	6	6	5	5	5	4	3	5	6	6	5	4	4	1	1	6	6
Rev-2	7	5	5	4	4	4	6	5	5	4	4	3	3	4	6	6	4	5	5	1	1	5	6
Rev-3	7	6	6	5	5	5	5	6	4	5	5	4	4	5	5	6	5	4	4	1	1	6	5
Rev-4	7	6	5	5	4	5	6	5	5	5	4	3	3	5	6	5	4	4	4	1	1	5	5
SOGC_2019 (Delivery and Postpartum Care)																							
Rev-1	6	6	7	6	3	7	7	6	6	6	6	6	3	6	6	6	6	4	3	1	1	2	6
Rev-2	5	5	7	5	3	7	7	5	5	5	6	5	4	5	4	5	5	3	3	1	1	2	6
Rev-3	6	5	6	5	4	7	7	5	4	5	5	6	3	5	5	6	5	4	4	1	1	2	5
Rev-4	5	5	6	6	3	7	7	5	6	6	5	5	4	6	6	5	6	3	3	1	1	2	6
SOGC_2022 (Twin Pregnancies)																							
Rev-1	6	6	6	6	3	7	7	6	6	6	6	6	5	6	6	6	6	4	3	1	1	2	6
Rev-2	5	5	6	6	3	7	7	5	5	6	6	5	4	5	6	5	5	3	3	1	1	2	6
Rev-3	5	5	5	5	4	6	7	5	4	6	5	6	4	5	5	6	5	4	4	1	1	2	5
Rev-4	6	6	6	5	3	7	7	5	6	6	5	5	5	6	6	5	6	4	3	1	1	2	6
SOAP_2019																							
Rev-1	6	6	5	6	6	5	4	4	4	4	5	5	6	3	5	5	5	4	3	1	1	2	7

For peer review only - http://bmjopen.bmj.com/site/about/guidelines.xhtml

Rev-2	6	5	5	6	6	5	3	4	4	4	5	5	6	3	5	6	5	3	3	1	1	2	6
Rev-3	5	5	5	5	5	5	4	4	3	4	5	6	5	3	5	5	5	4	3	1	1	2	7
Rev-4	6	5	5	6	6	5	4	4	4	4	5	5	6	3	5	6	5	4	3	1	1	2	6
AA and OAA_2020																							
Rev-1	5	5	5	4	1	1	2	1	1	1	1	1	1	1	4	4	5	2	1	1	1	6	6
Rev-2	5	5	4	6	1	1	2	1	1	1	1	1	1	1	4	3	4	2	2	1	1	5	6
Rev-3	6	5	4	4	1	1	2	1	1	1	1	1	1	1	4	4	5	2	1	1	1	5	5
Rev-4	5	5	4	5	1	1	2	1	1	1	1	1	1	1	4	3	5	2	2	1	1	6	6
PROSPECT_2020																							
Rev-1	5	6	5	2	1	2	7	7	6	7	7	7	1	1	7	6	7	6	1	1	1	6	6
Rev-2	5	5	5	2	1	2	7	7	7	7	7	7	3	1	6	6	6	6	3	1	1	6	6
Rev-3	5	6	5	2	1	2	7	7	6	6	7	7	1	1	6	6	6	5	1	1	1	5	6
Rev-4	5	5	5	2	1	2	7	7	7	7	6	7	3	1	6	6	6	6	3	1	1	6	5
CHBSA_2020																							
Rev-1	5	5	5	4	3	2	1	1	1	1	4	1	1	1	5	5	5	2	1	1	1	1	1
Rev-2	5	5	5	4	2	2	1	1	1	1	4	1	1	1	5	5	4	2	1	1	1	1	1
Rev-3	5	6	5	4	3	2	1	1	1	1	3	1	1	1	4	5	5	2	1	1	1	1	1
Rev-4	5	5	5	4	2	2	1	1	1	1	3	1	1	1	5	4	4	2	1	1	1	1	1
CAA_2020 (spinal)																							
Rev-1	5	6	6	5	1	7	1	1	1	1	3	1	1	1	6	6	6	1	5	1	1	1	1
Rev-2	6	6	6	5	1	6	1	1	1	1	2	1	1	1	5	5	5	1	4	1	1	1	1
Rev-3	5	6	5	5	1	6	1	1	1	1	2	1	1	1	5	6	5	1	4	1	1	1	1
Rev-4	5	6	6	4	1	6	1	1	1	1	2	1	1	1	5	5	5	1	4	1	1	1	1
CAA_2020 (delivery analgesia)																							
Rev-1	6	6	5	5	2	4	1	1	1	1	3	1	1	1	7	6	6	1	3	1	1	1	1
Rev-2	6	6	6	6	2	5	1	1	1	1	4	1	1	1	7	6	6	1	2	1	1	1	1
Rev-3	5	6	5	5	2	4	1	1	1	1	3	1	1	1	7	6	6	1	2	1	1	1	1

For peer review only - http://bmjopen.bmj.com/site/about/guidelines.xhtml

Rev-4	6	6	5	6	2	4	1	1	1	1	4	1	1	1	7	6	6	1	2	1	1	1	1
SOAP_2020																							
Rev-1	5	5	5	6	3	2	6	5	7	6	2	3	1	1	6	6	7	5	3	1	1	1	6
Rev-2	6	6	5	7	4	2	6	6	7	6	4	4	1	1	6	6	6	6	3	1	1	1	6
Rev-3	5	5	5	6	3	2	6	5	6	5	3	4	1	1	5	6	5	6	2	1	1	1	5
Rev-4	5	6	6	7	3	2	6	6	7	6	4	4	1	1	6	5	6	6	3	1	1	1	6
NICE_2021																							
Rev-1	5	7	7	6	6	5	1	1	1	1	6	1	1	7	3	5	3	3	4	1	1	1	1
Rev-2	6	6	7	7	6	6	1	1	1	1	7	1	1	7	5	6	6	5	6	1	1	1	1
Rev-3	6	7	7	6	5	5	1	1	1	1	7	1	1	6	3	4	4	3	4	1	1	1	1
Rev-4	6	6	7	7	6	6	1	1	1	1	7	1	1	7	5	6	6	5	6	1	1	1	1
OAA_2022																							
Rev-1	5	5	4	6	1	4	5	4	1	4	4	1	1	1	3	3	5	5	3	1	1	2	4
Rev-2	5	5	6	6	1	3	6	3	2	3	4	1	1	1	5	5	5	4	2	1	1	4	5
Rev-3	5	5	5	5	1	4	6	4	1	5	3	1	1	1	4	5	5	4	3	1	1	4	4
Rev-4	6	5	6	6	1	3	6	2	2	3	4	1	1	1	5	5	5	4	2	1	1	4	5
ANNA_2023																							
Rev-1	6	6	5	6	3	5	1	1	1	2	1	1	1	3	6	6	5	5	2	1	1	6	6
Rev-2	6	6	6	6	2	6	1	1	1	2	1	1	1	2	7	7	6	5	3	1	1	6	6
Rev-3	5	5	5	5	3	6	1	1	1	2	1	1	1	4	6	6	6	5	2	1	1	6	6
Rev-4	6	5	6	6	2	6	1	1	1	2	1	1	1	2	7	7	6	5	3	1	1	5	6
RCoA_2023																							
Rev-1	7	6	6	7	3	7	6	7	7	7	7	5	5	7	6	6	7	6	6	6	5	7	7
Rev-2	6	6	7	7	4	6	5	7	7	7	7	6	6	6	7	7	7	6	6	6	4	6	6
Rev-3	7	5	6	7	4	6	6	7	7	7	7	6	5	7	7	6	7	6	6	5	5	6	6
Rev-4	6	6	7	7	4	6	5	7	7	7	7	6	6	6	7	7	7	6	5	6	5	6	6

For peer review only - http://bmjopen.bmj.com/site/about/guidelines.xhtml

Supplementary Table 7. The intraclass correlation coefficient between reviewers for each individual AGREE II domain item.

Document	The intraclass correlation coefficient (95% confidence interval)	*P* valve
OG-OG-CMA_2014	0.987 (0.975, 0.994)	<0.001
OG-A-CMA_2016	0.993 (0.986, 0.997)	<0.001
ASA_2016	0.989 (0.980, 0.995)	<0.001
AAGBI_2017	0.990 (0.982, 0.995)	<0.001
ERAS_2018 (Part 1)	0.975 (0.952, 0.988)	<0.001
ERAS_2018 (Part 2)	0.971 (0.945, 0.986)	<0.001
ERAS_2018 (Part 3)	0.967 (0.938, 0.985)	<0.001
ACOG_2019	0.970 (0.942, 0.986)	<0.001
SOGC_2019 (Delivery and Postpartum Care)	0.974 (0.950, 0.988)	<0.001
SOGC_2022 (Twin Pregnancies)	0.976 (0.953, 0.989)	<0.001
SOAP_2019	0.986 (0.974, 0.994)	<0.001
AA and OAA_2020	0.991 (0.983, 0.996)	<0.001
PROSPECT_2020	0.990 (0.981, 0.995)	<0.001
CHBSA_2020	0.994 (0.988, 0.997)	<0.001
CAA_2020 (spinal)	0.995 (0.989, 0.998)	<0.001
CAA_2020 (delivery analgesia)	0.996 (0.993, 0.998)	<0.001
SOAP_2020	0.987 (0.972, 0.994)	<0.001
NICE_2021	0.984 (0.966, 0.993)	<0.001
OAA_2022	0.970 (0.944, 0.986)	<0.001
ANNA_2023	0.991 (0.984, 0.996)	<0.001
RCoA_2023	0.925 (0.859, 0.965)	<0.001

For peer review only - http://bmjopen.bmj.com/site/about/guidelines.xhtml

Supplementary Table 8. Domain score for each included guidance document.

Document	Domain 1, %	Domain 2, %	Domain 3, %	Domain 4, %	Domain 5, %	Domain 6, %
OG-OG-CMA_2014	62.5	20.8	6.6	20.8	0.0	0.0
OG-A-CMA_2016	66.7	68.1	11.7	61.1	13.5	0.0
ASA_2016	100.0	94.4	91.3	80.6	41.7	79.2
AAGBI_2017	66.7	26.4	18.4	73.6	4.2	25.0
ERAS_2018 (Part 1)	77.8	51.4	69.9	61.1	25.0	47.9
ERAS_2018 (Part 2)	76.4	52.8	69.4	69.4	26.0	52.1
ERAS_2018 (Part 3)	79.2	55.6	69.4	69.4	34.4	37.5
ACOG_2019	83.3	59.7	60.7	72.2	27.1	75.0
SOGC_2019 (Delivery and Postpartum Care)	79.2	70.8	73.5	73.6	19.8	47.9
SOGC_2022 (Twin Pregnancies)	76.4	69.4	76.5	76.4	20.8	47.9
SOAP_2019	72.2	75.0	56.1	69.4	19.8	54.2
AA and OAA_2020	63.9	20.8	4.1	51.4	6.3	77.1
PROSPECT_2020	69.4	11.1	75.5	86.1	24.0	79.2
CHBSA_2020	68.1	30.6	7.1	61.1	4.2	0.0
CAA_2020 (spinal)	77.8	50.0	4.6	72.2	13.5	0.0
CAA_2020 (delivery analgesia)	77.8	48.6	7.1	88.9	5.2	0.0
SOAP_2020	72.2	48.6	53.1	80.6	27.1	39.6
NICE_2021	90.3	81.9	25.5	61.1	29.2	0.0
OAA_2022	69.4	40.3	28.6	59.7	19.8	50.0
ANNA_2023	76.4	61.1	7.7	87.5	22.9	81.3
RCoA_2023	87.5	77.8	90.3	95.8	76.0	87.5

For peer review only - http://bmjopen.bmj.com/site/about/guidelines.xhtml

Supplementary Table 9. Mean scores across reviewers for the individual AGREE II domain items.

Document	The intraclass correlation coefficient, 95% confidence interval																						
	Domain 1			Domain 2			Domain 3								Domain 4			Domain 5				Domain 6	
	1	2	3	4	5	6	7	8	9	10	11	12	13	14	15	16	17	18	19	20	21	22	23
OG-OG-CMA_2014	3.8	4.7	7.0	2.3	2.0	2.5	1.0	1.0	1.0	1.0	3.3	1.0	1.0	1.0	4.8	1.0	1.0	1.0	1.0	1.0	1.0	1.0	1.0
OG-A-CMA_2016	5.3	5.0	6.0	6.0	6.0	3.3	1.0	1.0	1.0	1.0	5.8	1.0	1.0	1.0	5.3	5.8	3.0	1.0	4.3	1.0	1.0	1.0	1.0
ASA_2016	7.0	7.0	7.0	6.8	6.5	6.8	7.0	6.5	6.0	7.0	6.8	6.0	6.5	6.0	6.0	5.8	5.8	6.0	6.0	1.0	1.0	5.5	6.0
AAGBI_2017	4.8	5.0	5.3	5.8	1.0	1.0	2.3	2.3	2.0	4.3	2.3	41.0	1.0	1.0	5.8	5.3	5.3	1.0	1.0	1.0	2.3	2.3	2.8
ERAS_2018 (Part 1)	5.5	5.8	5.8	5.0	3.8	3.5	6.0	5.8	5.8	5.8	5.8	5.8	3.5	3.0	5.3	4.0	4.8	2.0	5.0	1.0	2.0	2.3	5.5
ERAS_2018 (Part 2)	5.8	5.8	5.3	5.0	3.8	3.8	5.5	6.0	5.0	6.0	5.8	5.8	4.0	3.0	4.8	5.5	5.3	2.0	5.0	1.0	2.3	2.5	5.8
ERAS_2018 (Part 3)	5.8	5.8	5.8	4.8	4.0	4.3	5.8	5.8	5.8	5.8	5.8	5.8	3.5	3.0	5.3	5.0	5.3	3.5	5.3	1.0	2.5	1.0	5.5
ACOG_2019	7.0	5.8	5.3	4.8	4.3	4.8	5.8	5.5	4.8	4.8	4.5	3.5	3.3	4.8	5.8	5.8	4.5	4.3	4.3	1.0	1.0	5.5	5.5
SOGC_2019 (Delivery and Postpartum Care)	5.5	5.3	6.5	5.5	3.3	7.0	7.0	5.3	5.3	5.5	5.5	5.5	3.5	5.5	5.3	5.5	5.5	3.5	3.3	1.0	1.0	2.0	5.8
SOGC_2022 (Twin Pregnancies)	5.5	5.5	5.8	5.5	3.3	6.8	7.0	5.3	5.3	6.0	5.5	5.5	4.5	5.5	5.8	5.5	5.5	3.8	3.3	1.0	1.0	2.0	5.8
SOAP_2019	5.8	5.3	5.0	5.8	5.8	5.0	3.8	4.0	3.8	4.0	5.0	5.3	5.8	3.0	5.0	5.5	5.0	3.8	3.0	1.0	1.0	2.0	6.5
AA and OAA_2020	5.3	5.0	4.3	4.8	1.0	1.0	2.0	1.0	1.0	1.0	1.0	1.0	1.0	1.0	4.0	3.5	4.8	2.0	1.5	1.0	1.0	5.5	5.8
PROSPECT_2020	5.0	5.5	5.0	2.0	1.0	2.0	7.0	7.0	6.5	6.8	6.8	7.0	2.0	1.0	6.3	6.0	6.3	5.8	2.0	1.0	1.0	5.8	5.8
GHBSA_2020	5.0	5.3	5.0	4.0	2.5	2.0	1.0	1.0	1.0	1.0	3.5	1.0	1.0	1.0	4.8	4.8	4.5	2.0	1.0	1.0	1.0	1.0	1.0
CAA_2020 (spinal)	5.3	6.0	5.8	4.8	1.0	6.3	1.0	1.0	1.0	1.0	2.3	1.0	1.0	1.0	5.3	5.5	5.3	1.0	4.3	1.0	1.0	1.0	1.0
CAA_2020 (delivery analgesia)	5.8	6.0	5.3	5.5	2.0	4.3	1.0	1.0	1.0	1.0	3.5	1.0	1.0	1.0	7.0	6.0	6.0	1.0	2.3	1.0	1.0	1.0	1.0
SOAP_2020	5.3	5.5	5.3	6.5	3.3	2.0	6.0	5.5	6.8	5.8	3.3	3.8	1.0	1.0	5.8	5.8	6.0	5.8	2.8	1.0	1.0	1.0	5.8
NICE_2021	5.8	6.5	7.0	6.5	5.8	5.5	1.0	1.0	1.0	1.0	6.8	1.0	1.0	6.8	4.0	5.3	4.8	4.0	5.0	1.0	1.0	1.0	1.0
OAA_2022	5.3	5.0	5.3	5.8	1.0	3.5	5.8	3.3	1.5	3.8	3.8	1.0	1.0	1.0	4.3	4.5	5.0	4.3	2.5	1.0	1.0	3.5	4.5
ANNA_2023	5.8	5.5	5.5	5.8	2.5	5.8	1.0	1.0	1.0	2.0	1.0	1.0	1.0	2.8	6.5	6.5	5.8	5.0	2.5	1.0	1.0	5.8	6.0
RCoA_2023	6.5	5.8	6.5	7.0	3.8	6.3	5.5	7.0	7.0	7.0	7.0	5.8	5.5	6.5	6.8	6.5	7.0	6.0	5.8	5.8	4.8	6.3	6.3

For peer review only - http://bmjopen.bmj.com/site/about/guidelines.xhtml

Supplementary Table 10. Summary of recommendations for the peri-anesthetic evaluation and preparation by included guidance document.

Recommendations	Reference contents	OG-OG-CMA_2014	OG-A-CMA_2016	ASA_2016	AAGBI_2017	ERAS_2018 (Part 1)	ERAS_2018 (Part 2)	ERAS_2018 (Part 3)	ACOG_2019	SOGC_2019	SOGC_2022	SOAP_2019	AA and OAA_2020	PROSPECT_2020	CHBSA_2020	CAA_2020	CAA_2020	SOAP_2020	NICE_2021	OAA_2022	ANNA_2023	RCoA_2023
**Peri-anesthetic Evaluation and Preparation**																						
**1. History and physical examination before providing anesthetic care**	Yes	+	+	+	NA	NG	NA	NA	+	NA	NA	NA	NA	NA	+	+	+	NG	+/-	NA	+	NG
1.1 Maternal health and anesthetic history	Agree	+	+	+	NA	NG	NA	NA	+	NA	NA	NA	NA	NA	+	+	+	NG	NG	NA	+/-	NG
1.2 Relevant obstetric history	Agree	+	+	+	NA	NG	NA	NA	NG	NA	NA	NA	NA	NA	NG	+	+	NG	NG	NA	NG	NG
1.3 Baseline blood pressure measurement	Agree	+	+	+	NA	NG	NA	NA	NG	NA	NA	NA	NA	NA	+	-	+	NG	NG	NA	+/-	NG
1.4 Airway examination	Agree	NG	+	+	NA	NG	NA	NA	+	NA	NA	NA	NA	NA	NG	+	+	NG	NG	NA	+	NG
1.5 Heart and lung examination	Agree	NG	NG	+	NA	NG	NA	NA	+	NA	NA	NA	NA	NA	+	+	+	NG	NG	NA	+	NG

For peer review only - http://bmjopen.bmj.com/site/about/guidelines.xhtml

1.6 When a neuraxial anesthetic is planned, examine the patient's back	Agree	NG	+	+	NA	NG	NA	NA	+	NA	NA	NA	NA	NA	NG	+	+	NG	NG	NA	+	NG
1.7 Consultation between the obstetrician and the anesthesiologist	Agree	+	NG	+	NA	+	NA	NA	+	NA	NA	NA	NA	NA	NG	+	+	NG	NG	NA	+	NG
**2. Preoperative communication**	Encouraged	+	NG	+	NA	+	NA	NA	NG	NA	NA	NA	NA	NA	+	+	-	+/-	+	NA	+	+/-
**3. Intrapartum platelet count**	Yes	+	+	+	NA	NA	NA	NA	+	NA	NA	NA	NA	NA	NG	+	NG	NG	+	NA	NG	NG
-Routine	Agree	+	+	-	NA	NA	NA	NA	+	NA	NA	NA	NA	NA	NG	NG	NG	NG	-	NA	NG	NG
-Individualized	Agree	-	-	+	NA	NA	NA	NA	+	NA	NA	NA	NA	NA	NG	NG	NG	NG	+	NA	NG	NG
**4. Blood type and screen**	Yes	+	+	+	NA	NA	NA	NA	NG	NA	NA	NA	NA	NA	NG	+	NG	NG	+	NA	NG	NG
-Routine	Agree	+	+	-	NA	NA	NA	NA	NG	NA	NA	NA	NA	NA	NG	+	NG	NG	+	NA	NG	NG
-Individualized	Agree	-	-	+	NA	NA	NA	NA	NG	NA	NA	NA	NA	NA	NG	NG	NG	NG	-	NA	NG	NG
**5. Blood cross-match**	Yes	+	NG	+	NA	NA	NA	NA	NG	NA	NA	NA	NA	NA	NG	NG	NG	NG	+	NA	NG	NG
-Routine	Agree	+	NG	-	NA	NA	NA	NA	NG	NA	NA	NA	NA	NA	NG	NG	NG	NG	-	NA	NG	NG
-Individualized	Agree	-	NG	+	NA	NA	NA	NA	NG	NA	NA	NA	NA	NA	NG	NG	NG	NG	+	NA	NG	NG
**6. Peri-anesthetic recording of fetal heart rate**	Yes	+	+	+	NA	NA	NA	NA	+	+	+	NA	NA	NA	NG	+	+	NG	NG	NA	+	NG
**7. Aspiration prevention**	Provided	+	NG	+	NA	+	NA	NG	NA	+	NA	NA	NA	NA	+	+/-	NG	+/-	+/-	NA	+/-	+/-
7.1 Clear liquids	Up to 2 h	NG	NG	Up to 2 h	NA	Up to 2 h	NA	NG	NA	NG	NA	NA	NA	NA	Up to 2 h	+	NG	+	NG	NA	NG	+/-
7.2 Solids	6-8 h	+	NG	6-8 h	NA	Light meal to 6	NA	NG	NA	NG	NA	NA	NA	NA	6 h	+	NG	+	NG	NA	NG	NG

For peer review only - http://bmjopen.bmj.com/site/about/guidelines.xhtml

						h																
7.3 Antacids (surgical procedures-cesarean delivery or postpartum tubal ligation)	Agree	NG	NG	+	NA	+	NA	+	NA	NG	NA	NA	NA	NA	+	+	NG	NG	+	NA	NG	+
7.4 H_2-receptor antagonists (surgical procedures)	Agree	NG	NG	+	NA	+	NA	+	NA	NG	NA	NA	NA	NA	+	+	NG	NG	+	NA	NG	NG
7.5 Metoclopramide (surgical procedures)	Agree	NG	NG	+	NA	NG	NA	20 mg	NA	NG	NA	NA	NA	NA	NG	+	NG	NG	NG	NA	NG	NG
**8. Preoperative heparin or low-molecular-weight heparin**	Yes	NA	+	NG	NA	NA	NA	NA	+	NA	NA	NA	NA	NA	NA	NG	+/-	NG	NG	NA	+/-	+/-
8.1 Prophylactic subcutaneous unfractionated heparin (<5,000 units twice daily)	Provided	NA	NG	NG	NA	NA	NA	NA	+	NA	NA	NA	NA	NA	NA	NG	NG	NG	NG	NA	NG	NG
-Contraindication to neuraxial techniques	No	NA	NG	NG	NA	NA	NA	NA	+	NA	NA	NA	NA	NA	NA	NG	NG	NG	NG	NA	NG	NG
-Catheter placement or removal since last dose	4-6 hours	NA	NG	NG	NA	NA	NA	NA	4-6 h	NA	NA	NA	NA	NA	NA	NG	NG	NG	NG	NA	NG	NG
8.2 Intermediate unfractionated heparin (75,000-100,000 units)	Provided	NA	NG	NG	NA	NA	NA	NA	+	NA	NA	NA	NA	NA	NA	NG	NG	NG	NG	NA	NG	NG
-Catheter placement or	> 12 hours		NG	NG	NA	NA	NA	NA	> 12	NA	NA	NA	NA	NA	NA	NG	NG	NG	NG	NA	NG	NG

For peer review only - http://bmjopen.bmj.com/site/about/guidelines.xhtml

removal since last dose		NA							hours													
8.3 High-dose unfractionated heparin (total daily dose greater than 20,000 units)	Provided	NA	NG	NG	NA	NA	NA	NA	+	NA	NA	NA	NA	NA	NA	NG	NG	NG	NG	NA	NG	NG
-Catheter placement or removal since last dose	> 24 hours	NA	NG	NG	NA	NA	NA	NA	> 24 hours	NA	NA	NA	NA	NA	NA	NG	NG	NG	NG	NA	NG	NG
-With the activated partial thromboplastin time being within normal range	Yes	NA	NG	NG	NA	NA	NA	NA	+	NA	NA	NA	NA	NA	NA	NG	NG	NG	NG	NA	NG	NG

+: consistent the reference with content;

+/-: partial1y consistent the reference with content;

-: inconsistent the reference with content;

NA: not applicable;

NG: not given.

Supplementary Table 11. Summary of recommendations for the anesthetic care for labor and vaginal delivery by included guidance document.

For peer review only - http://bmjopen.bmj.com/site/about/guidelines.xhtml

For peer review only

Recommendations	Reference contents	OG-OG-CMA_2014	OG-A-CMA_2016	ASA_2016	AAGBI_2017	ERAS_2018 (Part 1)	ERAS_2018 (Part 2)	ERAS_2018 (Part 3)	ACOG_2019	SOGC_2019	SOGC_2022	SOAP_2019	AA and OAA_2020	PROSPECT_2020	CHBSA_2020	CAA_2020	CAA_2020	SOAP_2020	NICE_2021	OAA_2022	ANNA_2023	RCoA_2023
**Anesthetic Care for Labor and Vaginal Delivery**																						
**1. Equipment, facilities, and support personnel**	Agree	NA	+	+	NA	NA	NA	NA	+	NA	NA	NA	NA	NA	+	NA	+	NA	NA	NA	+	+
**2. Timing of neuraxial analgesia and outcome of labor**	Provided	NA	+	+	NA	NA	NA	NA	NG	NA	NA	NA	NA	NA	NG	NA	+	NA	NA	NA	NG	NG
2.1 Less 5 cm dilation	Agree	NA	+/-	+	NA	NA	NA	NA	NG	NA	NA	NA	NA	NA	NG	NA	+/-	NA	NA	NA	NG	NG
2.2 Offer neuraxial analgesia on an individualized basis	Agree	NA	+	+	NA	NA	NA	NA	NG	NA	NA	NA	NA	NA	NG	NA	+	NA	NA	NA	NG	NG
**3. Neuraxial analgesia and trial of labor after**	Agree	NA	NG	+	NA	NA	NA	NA	NG	NA	NA	NA	NA	NA	NG	NA	NG	NA	NA	NA	NG	NG

For peer review only

**prior cesarean delivery**																						
**4. Continuous infusion epidural analgesia**	Provided	NA	+	+	NA	NA	NA	NA	+	NA	+/-	NA	NA	NA	+	NA	+	NA	NA	NA	+	NG
4.1 Using local anesthetic	Agree	NA	+	+	NA	NA	NA	NA	+	NA	NG	NA	NA	NA	+	NA	+	NA	NA	NA	+	NG
4.2 Add opioid	Agree	NA	+	+	NA	NA	NA	NA	+	NA	NG	NA	NA	NA	NG	NA	+	NA	NA	NA	+/-	NG
4.3 Platelet concentration corresponding to contraindications to puncture	Less than 70*10^9/L	NA	NG	NG	NA	NA	NA	NA	+	NA	NG	NA	NA	NA	NG	NA	NG	NA	NA	NA	+	NG
4.4 Puncture location	L_{2-3} or L_{3-4}	NA	+	NG	NA	NA	NA	NA	NG	NA	NG	NA	NA	NA	NG	NA	+	NA	NA	NA	NG	NG
4.5 Analgesic concentrations	Provided	NA	+	NG	NA	NA	NA	NA	NG	NA	NG	NA	NA	NA	NG	NA	+	NA	NA	NA	+	NG
-Bupivacaine	Provided	NA	+	NG	NA	NA	NA	NA	NG	NA	NG	NA	NA	NA	NG	NA	+	NA	NA	NA	+	NG
--Loading dose	0.04-0.125%	NA	+	NG	NA	NA	NA	NA	NG	NA	NG	NA	NA	NA	NG	NA	+	NA	NA	NA	0.0625-0.125%	NG
--Maintenance dose	0.05-0.125%	NA	+	NG	NA	NA	NA	NA	NG	NA	NG	NA	NA	NA	NG	NA	+	NA	NA	NA	0.0625-0.125%	NG
-Ropivacaine	Provided	NA	+	NG	NA	NA	NA	NA	NG	NA	NG	NA	NA	NA	NG	NA	+	NA	NA	NA	+	NG
--Loading dose	0.0625-0.15%	NA	+	NG	NA	NA	NA	NA	NG	NA	NG	NA	NA	NA	NG	NA	+	NA	NA	NA	0.08-0.2%	NG
--Maintenance dose	0.0625-0.125%	NA	+	NG	NA	NA	NA	NA	NG	NA	NG	NA	NA	NA	NG	NA	+	NA	NA	NA	0.08-0.2%	NG
-	Provided	NA	NG	NG	NA	NA	NA	NA	NG	NA	NG	NA	NA	NA	NG	NA	+	NA	NA	NA	NG	NG

For peer review only - http://bmjopen.bmj.com/site/about/guidelines.xhtml

Levobupivacaine																						
--Loading dose	0.04-0.125%	NA	NG	NG	NA	NA	NA	NA	NG	NA	NG	NA	NA	NA	NG	NA	+	NA	NA	NA	NG	NG
--Maintenance dose	0.05-0.125%	NA	NG	NG	NA	NA	NA	NA	NG	NA	NG	NA	NA	NA	NG	NA	+	NA	NA	NA	NG	NG
4.6 Opioids concentrations	Provided	NA	+	NG	NA	NA	NA	NA	NG	NA	NG	NA	NA	NA	NG	NA	+	NA	NA	NA	+/-	NG
-Fentanyl	Provided	NA	+	NG	NA	NA	NA	NA	NG	NA	NG	NA	NA	NA	NG	NA	+	NA	NA	NA	NG	NG
--Loading dose	0.5-2 $\mu g.ml^{-1}$	NA	1-2	NG	NA	NA	NA	NA	NG	NA	NG	NA	NA	NA	NG	NA	+	NA	NA	NA	NG	NG
--Maintenance dose	1-2 $\mu g.ml^{-1}$	NA	+	NG	NA	NA	NA	NA	NG	NA	NG	NA	NA	NA	NG	NA	+	NA	NA	NA	NG	NG
-Sufentanyl	Provided	NA	+	NG	NA	NA	NA	NA	NG	NA	NG	NA	NA	NA	NG	NA	+	NA	NA	NA	NG	NG
--Loading dose	0.2-0.6 $\mu g.ml^{-1}$	NA	0.4-0.6	NG	NA	NA	NA	NA	NG	NA	NG	NA	NA	NA	NG	NA	+	NA	NA	NA	NG	NG
--Maintenance dose	0.3-0.6 $\mu g.ml^{-1}$	NA	0.4-0.6	NG	NA	NA	NA	NA	NG	NA	NG	NA	NA	NA	NG	NA	+	NA	NA	NA	NG	NG
**5. Combined spinal–epidural analgesia**	Provided	NA	+	+	NA	NA	NA	NA	+	NA	NA	NA	NA	NA	NG	NA	+	NA	NA	NA	+/-	NG
5.1 Using local anesthetic	Agree	NA	+	NG	NA	NA	NA	NA	+	NA	NA	NA	NA	NA	NG	NA	+	NA	NA	NA	+	NG
5.2 Add opioid	Agree	NA	+	NG	NA	NA	NA	NA	+	NA	NA	NA	NA	NA	NG	NA	+	NA	NA	NA	+	NG
5.3 Platelet concentration corresponding to contraindications to puncture	Less than $70*10^9/L$	NA	NG	NG	NA	NA	NA	NA	+	NA	NA	NA	NA	NA	NG	NA	+	NA	NA	NA	+	NG

For peer review only - http://bmjopen.bmj.com/site/about/guidelines.xhtml

5.4 Puncture location	L_{2-3} or L_{3-4}	NA	+	NG	NA	NA	NA	NA	NG	NA	NA	NA	NA	NA	NG	NA	+/-	NA	NA	NA	NG	NG
5.5 Epidural anesthetics (consistent with 4.5 and 4.6)	Provided	NA	+	NG	NA	NA	NA	NA	NG	NA	NA	NA	NA	NA	NG	NA	+	NA	NA	NA	+/-	NG
5.6 Spinal anesthetics	Provided	NA	+	NG	NA	NA	NA	NA	+	NA	NA	NA	NA	NA	NG	NA	+	NA	NA	NA	NG	NG
-Protocol 1	Provided	NA	+	NG	NA	NA	NA	NA	+	NA	NA	NA	NA	NA	NG	NA	+	NA	NA	NA	NG	NG
--Single sufentanyl	2.5-7 μg	NA	+	NG	NA	NA	NA	NA	+/-	NA	NA	NA	NA	NA	NG	NA	+	NA	NA	NA	NG	NG
--Single ropivacaine	2.3-3 mg	NA	2.5-3	NG	NA	NA	NA	NA	+/-	NA	NA	NA	NA	NA	NG	NA	+	NA	NA	NA	NG	NG
--Combined sufentanyl and ropivacaine	2.5 μg / 2.5 mg	NA	+	NG	NA	NA	NA	NA	+/-	NA	NA	NA	NA	NA	NG	NA	+	NA	NA	NA	Sufentanyl 5-10 μg	NG
-Protocol 2	Provided	NA	+	NG	NA	NA	NA	NA	+/-	NA	NA	NA	NA	NA	NG	NA	+	NA	NA	NA	NG	NG
--Single fentanyl	15-25 μg	NA	+	NG	NA	NA	NA	NA	+/-	NA	NA	NA	NA	NA	NG	NA	+	NA	NA	NA	NG	NG
--Single bupivacaine	2.0-2.5 mg	NA	+	NG	NA	NA	NA	NA	+/-	NA	NA	NA	NA	NA	NG	NA	+	NA	NA	NA	NG	NG
--Combined fentanyl and bupivacaine	2.5 μg / 2.0 mg	NA	+	NG	NA	NA	NA	NA	+/-	NA	NA	NA	NA	NA	NG	NA	+	NA	NA	NA	Fentanyl 50-100 μg	NG
**6. Single-injection spinal**	Provided	NA	NG	+	NA	NA	NA	NA	+	NA	NA	NA	NA	NA	NG	NA	+	NA	NA	NA	+	NG
6.1 Using local anesthetic	Agree	NA	NG	+	NA	NA	NA	NA	+	NA	NA	NA	NA	NA	NG	NA	+	NA	NA	NA	NG	NG

For peer review only - http://bmjopen.bmj.com/site/about/guidelines.xhtml

6.2 Add opioid	Agree	NA	NG	+	NA	NA	NA	NA	+	NA	NA	NA	NA	NA	NG	NA	+	NA	NA	NA	NG	NG
6.3 Platelet concentration corresponding to contraindications to puncture	Less than $70*10^9$/L	NA	NG	NG	NA	NA	NA	NA	+	NA	NA	NA	NA	NA	NG	NA	NG	NA	NA	NA	+	NG
6.4 Puncture location	L_{3-4}	NA	NG	NG	NA	NA	NA	NA	NG	NA	NA	NA	NA	NA	NG	NA	NG	NA	NA	NA	NG	NG
6.6 Analgesic concentrations (consistent with 5.6)	Agree	NA	NG	NG	NA	NA	NA	NA	NG	NA	NA	NA	NA	NA	NG	NA	+	NA	NA	NA	NG	NG
6.7 Spinal needles	Provided	NA	NG	+	NA	NA	NA	NA	NG	NA	NA	NA	NA	NA	NG	NA	NG	NA	NA	NA	+	NG
-Cutting-bevel	Disagree	NA	NG	+	NA	NA	NA	NA	NG	NA	NA	NA	NA	NA	NG	NA	NG	NA	NA	NA	+	NG
-Pencil-point	Agree	NA	NG	+	NA	NA	NA	NA	NG	NA	NA	NA	NA	NA	NG	NA	NG	NA	NA	NA	+	NG
**7. Patient-controlled epidural analgesia (for labor analgesia)**	Agree	NA	+	+	NA	NA	NA	NA	NG	NA	NA	NA	NA	NA	+	NA	+	NA	NA	NA	+	NG

+: consistent the reference with content;

+/-: partial1y consistent the reference with content;

-: inconsistent the reference with content;

NA: not applicable;

NG: not given.

For peer review only - http://bmjopen.bmj.com/site/about/guidelines.xhtml

For peer review only

Supplementary Table 12. Summary of recommendations for the anesthetic care for cesarean delivery by included guidance document.

For peer review only - http://bmjopen.bmj.com/site/about/guidelines.xhtml

**Recommendations**	**Reference contents**	OG-OG-CMA_2014	OG-A-CMA_2016	ASA_2016	AAGBI_2017	ERAS_2018 (Part 1)	ERAS_2018 (Part 2)	ERAS_2018 (Part 3)	ACOG_2019	SOGC_2019	SOGC_2022	SOAP_2019	AA and OAA_2020	PROSPECT_2020	CHBSA_2020	CAA_2020	CAA_2020	SOAP_2020	NICE_2021	OAA_2022	ANNA_2023	RCoA_2023
**Anesthetic Care for Cesarean Delivery**																						
**1. Equipment, facilities, and support personnel**	Agree	+	NA	+	NA	NA	NG	NA	+	+	NA	NA	NA	NA	+	NG	NA	NA	NA	NA	+	+
**2. Continuous infusion epidural analgesia**	Provided	+	NA	+	NA	NA	+/-	NA	+	+/-	NA	NA	NA	NA	+	+	NA	NA	NA	NA	+	NG
2.1 Using local anesthetic	Agree	NG	NA	NG	NA	NA	NG	NA	+	NG	NA	NA	NA	NA	+	+	NA	NA	NA	NA	+	NG
2.2 Add opioid	Agree	NG	NA	NG	NA	NA	NG	NA	+	NG	NA	NA	NA	NA	NG	+	NA	NA	NA	NA	+	NG
2.3 Platelet concentration corresponding to contraindications to puncture	Less than 70*10^9/L	NG	NA	NG	NA	NA	NG	NA	+	NG	NA	NA	NA	NA	NG	+	NA	NA	NA	NA	+	NG
2.4 Puncture location	L_{1-2} or L_{2-3}	NG	NA	NG	NA	NA	NG	NA	NG	NG	NA	NA	NA	NA	NG	+	NA	NA	NA	NA	NG	NG
2.5 Recommend	T_{6-4}		NA	NG	NA	NA	NG	NA	T4	NG	NA	NA	NA	NA	NG	+	NA	NA	NA	NA	NG	NG

For peer review only - http://bmjopen.bmj.com/site/about/guidelines.xhtml

For peer review only

level of block		NG																				
2.6 Analgesic concentrations	Provided	NG	NA	NG	NA	NA	NG	NA	NG	NG	NA	NA	NA	NA	NG	+	NA	NA	NA	NA	+/-	NG
-Lidocaine	1.5%-2%	NG	NA	NG	NA	NA	NG	NA	NG	NG	NA	NA	NA	NA	NG	+	NA	NA	NA	NA	NG	NG
-Bupivacaine	0.50%	NG	NA	NG	NA	NA	NG	NA	NG	NG	NA	NA	NA	NA	NG	+	NA	NA	NA	NA	0.0625-0.125%	NG
-Ropivacaine	0.5%-0.75%	NG	NA	NG	NA	NA	NG	NA	NG	NG	NA	NA	NA	NA	NG	+	NA	NA	NA	NA	0.08-0.2%	NG
-Levobupivacaine	0.5%-0.75%	NG	NA	NG	NA	NA	NG	NA	NG	NG	NA	NA	NA	NA	NG	+	NA	NA	NA	NA	NG	NG
-Chloroprocaine	3%	NG	NA	NG	NA	NA	NG	NA	NG	NG	NA	NA	NA	NA	NG	+	NA	NA	NA	NA	NG	NG
2.7 Prevention of local anesthetic poisoning	Provided	NG	NA	NG	NA	NA	NG	NA	+	NG	NA	NA	NA	NA	NG	+	NA	NA	NA	NA	NA	NG
-Withdrawal before injection	Agree	NG	NA	NG	NA	NA	NG	NA	NG	NG	NA	NA	NA	NA	NG	+	NA	NA	NA	NA	NA	NG
-Administration of test dose	Agree	NG	NA	NG	NA	NA	NG	NA	NG	NG	NA	NA	NA	NA	NG	+	NA	NA	NA	NA	NA	NG
-Addition of epinephrine	Agree	NG	NA	NG	NA	NA	NG	NA	+	NG	NA	NA	NA	NA	NG	+	NA	NA	NA	NA	NA	NG

For peer review only

For peer review only - http://bmjopen.bmj.com/site/about/guidelines.xhtml

**3. Single-injection spinal**	Provided	NA	NA	+	+/-	NA	+/-	NA	+	+/-	NA	NA	NA	NA	+	+	NA	NA	NA	NA	+	NG
3.1 Using local anesthetic	Agree	NA	NA	NG	NG	NA	NG	NA	+	NG	NA	NA	NA	NA	+	+	NA	NA	NA	NA	+	NG
3.2 Add opioid	Agree	NA	NA	NG	NG	NA	NG	NA	+	NG	NA	NA	NA	NA	NG	+	NA	NA	NA	NA	+	NG
3.3 Platelet concentration corresponding to contraindications to puncture	Less than $70*10^9/L$	NA	NA	NG	NG	NA	NG	NA	+	NG	NA	NA	NA	NA	NG	+	NA	NA	NA	NA	+	NG
3.4 Puncture location	$L_{3\text{-}4}$	NA	NA	NG	NG	NA	NG	NA	NG	NG	NA	NA	NA	NA	NG	+	NA	NA	NA	NA	NG	NG
3.5 Recommend level of block	$T_{6\text{-}4}$	NA	NA	NG	NG	NA	NG	NA	NG	NG	NA	NA	NA	NA	NG	+	NA	NA	NA	NA	NG	NG
3.6 Analgesic dose	Provided	NA	NA	NG	NG	NA	NG	NA	NG	NG	NA	NA	NA	NA	NG	+	NA	NA	NA	NA	NG	NG
-Bupivacaine	5-15 mg	NA	NA	NG	NG	NA	NG	NA	NG	NG	NA	NA	NA	NA	NG	+	NA	NA	NA	NA	NG	NG
-Ropivacaine	10-20 mg	NA	NA	NG	NG	NA	NG	NA	NG	NG	NA	NA	NA	NA	NG	+	NA	NA	NA	NA	NG	NG
3.7 Spinal needles	Provided	NA	NA	+	NG	NA	NG	NA	NG	NG	NA	NA	NA	NA	NG	+	NA	NA	NA	NA	+	NG
-Cutting-bevel	Disagree	NA	NA	+	NG	NA	NG	NA	NG	NG	NA	NA	NA	NA	NG	+	NA	NA	NA	NA	+	NG
-Pencil-point	Agree	NA	NA	+	NG	NA	NG	NA	NG	NG	NA	NA	NA	NA	NG	+	NA	NA	NA	NA	+	NG
**4. Combined spinal–epidural analgesia**	Provided	+	NA	+	+	NA	+/-	NA	+	+	NA	NA	NA	NA	+	+	NA	NA	NA	NA	+/-	NG
4.1 Puncture location	Provided	NG	NA	NG	NG	NA	NG	NA	NG	NG	NA	NA	NA	NA	NG	+	NA	NA	NA	NA	+/-	NG
-Single space	$L_{3\text{-}4}$	NG	NA	NG	NG	NA	NG	NA	NG	NG	NA	NA	NA	NA	NG	+	NA	NA	NA	NA	+/-	NG

For peer review only - http://bmjopen.bmj.com/site/about/guidelines.xhtml

technique																						
-Double space technique	T_{12}-L_2/L_{3-5}	NG	NA	NG	NG	NA	NG	NA	NG	NG	NA	NA	NA	NA	NG	+	NA	NA	NA	NA	+/-	NG
4.2 Anesthetics (consistent with spinal or epidural)	Agree	NG	NA	NG	NG	NA	NG	NA	+	NG	NA	NA	NA	NA	NG	+	NA	NA	NA	NA	+/-	NG
**5. General anesthesia**	Provided	+	NA	+	NA	NA	NA	NA	+	NA	NA	NA	NA	NA	+	+	NA	NA	+	NA	+	+/-
5.1 General anesthesia selected condition	Provided	NG	NA	+	NA	NA	NA	NA	+	NA	NA	NA	NA	NA	NG	NG	NA	NA	NG	NA	+	NG
-Profound fetal bradycardia	Agree	NG	NA	+	NA	NA	NA	NA	NG	NA	NA	NA	NA	NA	NG	NG	NA	NA	NG	NA	NG	NG
-Ruptured uterus	Agree	NG	NA	+	NA	NA	NA	NA	NG	NA	NA	NA	NA	NA	NG	NG	NA	NA	NG	NA	NG	NG
-Severe Hemorrhage	Agree	NG	NA	+	NA	NA	NA	NA	NG	NA	NA	NA	NA	NA	NG	NG	NA	NA	NG	NA	NG	NG
-Severe placental abruption	Agree	NG	NA	+	NA	NA	NA	NA	NG	NA	NA	NA	NA	NA	NG	NG	NA	NA	NG	NA	NG	NG
-Umbilical cord prolapse	Agree	NG	NA	+	NA	NA	NA	NA	NG	NA	NA	NA	NA	NA	NG	NG	NA	NA	NG	NA	NG	NG
-Preterm footling breech	Agree	NG	NA	+	NA	NA	NA	NA	NG	NA	NA	NA	NA	NA	NG	NG	NA	NA	NG	NA	NG	NG
-Inability to place neuraxial	Agree	NG	NA	NG	NA	NA	NA	NA	+	NA	NA	NA	NA	NA	NG	NG	NA	NA	NG	NA	+	NG

For peer review only - http://bmjopen.bmj.com/site/about/guidelines.xhtml

anesthesia																						
5.2 Intravenous anesthetics recommendation	Provided	NG	NA	NG	NA	NA	NA	NA	+	NA	NA	NA	NA	NA	NG	+	NA	NA	NG	NA	NG	NG
-Thiopental sodium	4-5 $mg.kg^{-1}$	NG	NA	NG	NA	NA	NA	NA	NG	NA	NA	NA	NA	NA	NG	+	NA	NA	NG	NA	NG	NG
-Propofol	1.5-2.5 $mg.kg^{-1}$	NG	NA	NG	NA	NA	NA	NA	+	NA	NA	NA	NA	NA	NG	+	NA	NA	NG	NA	NG	NG
-Etomidate	0.2-0.3 $mg.kg^{-1}$	NG	NA	NG	NA	NA	NA	NA	NG	NA	NA	NA	NA	NA	NG	+	NA	NA	NG	NA	NG	NG
-Ketamine	0.5~1 $mg.kg^{-1}$	NG	NA	NG	NA	NA	NA	NA	+	NA	NA	NA	NA	NA	NG	+	NA	NA	NG	NA	NG	NG
5.3 Opioids analgesic recommendation	Provided	NG	NA	NG	NA	NA	NA	NA	NG	NA	NA	NA	NA	NA	NG	+	NA	NA	NG	NA	NG	NG
-Fentanyl	2-5 $\mu g.kg^{-1}$	NG	NA	NG	NA	NA	NA	NA	NG	NA	NA	NA	NA	NA	NG	+	NA	NA	NG	NA	NG	NG
-Sufentanyl	0.2~0.5 $\mu g.kg^{-1}$	NG	NA	NG	NA	NA	NA	NA	NG	NA	NA	NA	NA	NA	NG	+	NA	NA	NG	NA	NG	NG
-Remifentanyl	0.5-1 $\mu g.kg^{-1}$ for IV	NG	NA	NG	NA	NA	NA	NA	NG	NA	NA	NA	NA	NA	NG	+	NA	NG	NG	NA	NG	NG
-Remifentanyl	4 $ng.kg^{-1}$ for target	NG	NA	NG	NA	NA	NA	NA	NG	NA	NA	NA	NA	NA	NG	+	NA	NG	NG	NA	NG	NG

For peer review only

5.4 Muscle relaxants recommendation	Provided	NG	NA	NG	NA	NA	NA	NA	+	NA	NA	NA	NA	NA	NG	+	NA	NG	NG	NA	NG	NG
-Succinylcholine	1-1.5 mg.kg^{-1}	NG	NA	NG	NA	NA	NA	NA	NG	NA	NA	NA	NA	NA	NG	+	NA	NG	NG	NA	NG	NG
-Rocuronium	0.6-1.2 mg.kg^{-1}	NG	NA	NG	NA	NA	NA	NA	NG	NA	NA	NA	NA	NA	NG	+	NA	NG	NG	NA	NG	NG
5.5 Induction-delivery interval	within 10 min	NG	NA	NG	NA	NA	NA	NA	NG	NA	NA	NA	NA	NA	NG	+	NA	NG	NG	NA	NG	NG

+: consistent the reference with content;

+/-: partial1y consistent the reference with content;

-: inconsistent the reference with content;

NA: not applicable;

NG: not given.

For peer review only

Supplementary Table 13. Summary of recommendations for the monitoring and management of obstetric and anesthetic or intra- or postoperative pain management by included guidance document.

		OG-OG-CMA_2014	OG-A-CMA_2016	ASA_2016	AAGBI_2017	ERAS_2018 (Part 1)	ERAS_2018 (Part 2)	ERAS_2018 (Part 3)	ACOG_2019	SOGC_2019	SOGC_2022	SOAP_2019	AA and OAA_2020	PROSPECT_2020	CHBSA_2020	CAA_2020	CAA_2020	SOAP_2020	NICE_2021	OAA_2022	ANNA_2023	RCoA_2023
**Recommendations**	**Reference contents**																					
**Neurological Monitoring Associated with Obstetric Neuraxial Block**																						
**1. Screening method (During the recovery phase) - Straight-leg raising**	Agree	NA	NA	NA	NA	NA	NA	NA	NA	NA	NA	NA	+	NA	NA	NA	NA	NA	NA	NA	NA	NA
**2. Alerts incidences-unable to straight-leg raise at 4 h from the last dose**	Agree	NA	NA	NA	NA	NA	NA	NA	NA	NA	NA	NA	+	NA	NA	NA	NA	NA	NA	NA	NA	NA
**Respiratory Depression Monitoring with Neuraxial Block With Opioids**																						
**1. Frequency and**	Provided	+	NA	NA	NA	NA	NA	NA	NA	NA	NA	+	NA	NA	NA	NA	NA	NA	+/-	NA	+/-	NA

For peer review only

**modality of respiratory monitoring should be based on:**																						
1.1 Low risk	Provided	NG	NA	NA	NA	NA	NA	NA	NA	NA	NA	+	NA	NA	NA	NA	NA	NA	+/-	NA	NG	NA
-Neuraxial morphine dose (intrathecal ≤ 0.15 mg or epidural ≤ 3 mg)	Agree	NG	NA	NA	NA	NA	NA	NA	NA	NA	NA	+	NA	NA	NA	NA	NA	NA	NG	NA	NG	NA
1.2 High risk	Provided	NG	NA	NA	NA	NA	NA	NA	NA	NA	NA	+	NA	NA	NA	NA	NA	NA	+/-	NA	NG	NA
Cardiopulmonary/ neurological comorbidity	Agree	NG	NA	NA	NA	NA	NA	NA	NA	NA	NA	+	NA	NA	NA	NA	NA	NA	NG	NA	NG	NA
-Obesity (body mass index ≥ 40 kg/m^2)	Agree	NG	NA	NA	NA	NA	NA	NA	NA	NA	NA	+	NA	NA	NA	NA	NA	NA	NG	NA	NG	NA
-Known or suspected obstructive sleep apnea	Agree	NG	NA	NA	NA	NA	NA	NA	NA	NA	NA	+	NA	NA	NA	NA	NA	NA	NG	NA	NG	NA
-Chronic opioid use or abuse	Agree	NG	NA	NA	NA	NA	NA	NA	NA	NA	NA	+	NA	NA	NA	NA	NA	NA	NG	NA	NG	NA
-Hypertension	Agree	NG	NA	NA	NA	NA	NA	NA	NA	NA	NA	+	NA	NA	NA	NA	NA	NA	NG	NA	NG	NA
-Magnesium administration	Agree	NG	NA	NA	NA	NA	NA	NA	NA	NA	NA	+	NA	NA	NA	NA	NA	NA	NG	NA	NG	NA
-Neuraxial morphine dose	Agree	NG	NA	NA	NA	NA	NA	NA	NA	NA	NA	+	NA	NA	NA	NA	NA	NA	NG	NA	NG	NA

For peer review only

(intrathecal > 0.15 mg or epidural > 3 mg)																						
1.3 Respiratory monitoring frequency and duration	Provided	NG	NA	NA	NA	NA	NA	NA	NA	NA	NA	+	NA	NA	NA	NA	NA	NA	+/-	NA	NG	NA
-Low risk	Provided	NG	NA	NA	NA	NA	NA	NA	NA	NA	NA	+	NA	NA	NA	NA	NA	NA	+/-	NA	NG	NA
--Routine postoperative vital sign monitoring	Agree	NG	NA	NA	NA	NA	NA	NA	NA	NA	NA	+	NA	NA	NA	NA	NA	NA	+	NA	NG	NA
-- q2h clinical assessment for 12 h	Agree	+/-	NA	NA	NA	NA	NA	NA	NA	NA	NA	+	NA	NA	NA	NA	NA	NA	+	NA	NG	NA
-High risk	Provided	NG	NA	NA	NA	NA	NA	NA	NA		NA	+		NA	NA	NA	NA	NA	+	NA	NG	NA
-- q1h clinical assessment for first 12 h	Agree	NG	NA	NA	NA	NA	NA	NA	NA	NA	NA	+	NA	NA	NA	NA	NA	NA	half hour for 2 hours	NA	NG	NA
-- q2h clinical assessment for first 12-24 h	Agree	+/-	NA	NA	NA	NA	NA	NA	NA	NA	NA	+	NA	NA	NA	NA	NA	NA	NG	NA	NG	NA
--Consider additional monitoring modalities (e.g., pulse oximetry, capnography)	Agree	NG	NA	NA	NA	NA	NA	NA	NA	NA	NA	+	NA	NA	NA	NA	NA	NA	+/-	NA	NG	NA
**Management of**																						

For peer review only - http://bmjopen.bmj.com/site/about/guidelines.xhtml

**Intra- or Postoperative Pain**																						
**1. Implement strategies to minimize systemic opioid utilization**	Provided	NA	NA	NA	NA	NA	NA	+	+	NA	NA	+	NA	+	+	NA	NA	+	+	+	+/-	NA
**2. Neuraxial adjuvant drugs**	Provided	NA	NA	NA	NA	NA	NA	+	+	NA	NA	+	NA	+	+	NA	NA	+	+	NG	+	NA
-Spinal anesthesia	Provided	NA	NA	NA	NA	NA	NA	NG	+	NA	NA	+	NA	+	+	NA	NA	+	+	NG	+	NA
--Morphine	50-150 µg	NA	NA	NA	NA	NA	NA	NG	+	NA	NA	50-150 µg	NA	+	+	NA	NA	50-150 µg	NG	NG	150-500µg	NA
-Hydromorphone	75-150 µg	NA	NA	NA	NA	NA	NA	NG	NG	NA	NA	NG	NA	NG	NG	NA	NA	NG	NG	NG	+	NA
-Epidural or combined spinal-epidural technique.	Provided	NA	NA	NA	NA	NA	NA	NG	+	NA	NA	+	NA	+	NG	NA	NA	+	+	NG	+	NA
--Morphine	1-3 mg	NA	NA	NA	NA	NA	NA	NG	+	NA	NA	1-3 mg	NA	+	NG	NA	NA	1~3 mg	NG	NG	1-3mg	NA
--Diamorphine	2-3 mg	NA	NA	NA	NA	NA	NA	NG	NG	NA	NA	NG	NA	+	NG	NA	NA	NG	2.5-5 mg	NG	NG	NA
**3. Others analgesic (after delivery)**	Provided	NA	NA	NA	NA	NA	NA	+	+	NA	NA	+	NA	+	+	NA	NA	+	+	NG	+	NA
-Non-steroidal anti-inflammatory	Agree	NA	NA	NA	NA	NA	NA	+	+	NA	NA	+	NA	+	+	NA	NA	+	+	NG	+	NA

For peer review only - http://bmjopen.bmj.com/site/about/guidelines.xhtml

drug (e.g., paracetamol)																						
-A single dose of intravenous dexamethasone	Agree	NA	NA	NA	NA	NA	NA	NG	NG	NA	NA	NG	NA	+	NG	NA	NA	NG	NG	NG	+	NA
**4. Local anesthetic infiltration**	Agree	NA	NA	NA	NA	NA	NA	+	+	NA	NA	NG	NA	+	+	NA	NA	+	NG	NG	+	NA
**5. Continuous wound local anesthetic infusion**	Agree	NA	NA	NA	NA	NA	NA	NG	+	NA	NA	NG	NA	+	+	NA	NA	NG	NG	NG	NG	NA
**6. Fascial plane blocks (e.g., transversus abdominis plane, erector spinae plane)**	Agree	NA	NA	NA	NA	NA	NA	+	+	NA	NA	NG	NA	+	+	NA	NA	+	NG	NG	NG	NA
**7. Patient-controlled intravenous analgesia**	Agree	NA	NA	NA	NA	NA	NA	NG	+	NA	NA	NG	NA	+	+	NA	NA	NG	+	NG	NG	NA

+: consistent the reference with content;
+/-: partial1y consistent the reference with content;
-: inconsistent the reference with content;
NA: not applicable;
NG: not given.

For peer review only - http://bmjopen.bmj.com/site/about/guidelines.xhtml

For peer review only

Supplementary Table 14. Summary of recommendations for the adverse events by included guidance document.

For peer review only - http://bmjopen.bmj.com/site/about/guidelines.xhtml

Recommendations	Reference contents	OG-OG-CMA_2014	OG-A-CMA_2016	ASA_2016	AAGBI_2017	ERAS_2018 (Part 1)	ERAS_2018 (Part 2)	ERAS_2018 (Part 3)	ACOG_2019	SOGC_2019	SOGC_2022	SOAP_2019	AA and OAA_2020	PROSPECT_2020	CHBSA_2020	CAA_2020	CAA_2020	SOAP_2020	NICE_2021	OAA_2022	ANNA_2023	RCoA_2023
**1. Hypotension prevention strategies**	Provided	NA	NA	+	+	NA	+/-	+	+	+	NA	NA	NA	NA	+	+	+	+	+	NA	+/-	NA
1.1 Blood pressure target of systolic arterial pressure	≥90% of baseline and avoid decrease to < 80% baseline	NA	NA	NG	+	NA	NG	NG	NG	NG	NA	NA	NA	NA	NG	NG	NG	NG	+	NA	NG	NA
1.2 Position (uterine left-leaning)	Yes	NA	NA	+	+	NA	NG	NG	NG	NG	NA	NA	NA	NA	+	+	+	NG	+	NA	+	NA
1.3 Volume expansion (crystals or colloids)	500-1000 ml	NA	NA	+	+	NA	+	+	+	+	NA	NA	NA	NA	+	+	+	NG	+	NA	+	NA
1.4 Vasoactive drugs	Yes	NA	NA	+	+	NA	NG	+	+	NG	NA	NA	NA	NA	+	+	+	+	+	NA	+	NA
-Pump infusion is recommended	Provided	NA	NA	+	+	NA	NG	NG	NG	NG	NA	NA	NA	NA	NG	NG	NG	NG	NG	NA	NG	NA
-Type of drugs	Provided	NA	NA	+	+	NA	NG	+	+	NG	NA	NA	NA	NA	+	+	+	+	NG	NA	+/-	NA

For peer review only

--Phenylephrine	20-50 µg Or 0.5 µg . kg^{-1}. min^{-1}	NA	NA	+	20~50 µg or 0.25. kg^{-1}. min^{-1}	NA	NG	+	+	NG	NA	NA	NA	NA	+	+	+	+	NG	NA	+	NA
--Metaraminol	1-2 mg or 4 µg.kg^{-1}. min^{-1}	NA	NA	NG	0.5mg.ml^{-1}	NA	NG	NG	NG	NG	NA	NA	NA	NA	NG	NG	NG	NG	NG	NA	NG	NA
--Noradrenalin	4-6 µg or 0.08 µg kg^{-1}. min^{-1}	NA	NA	NG	NG	NA	NG	NG	NG	NG	NA	NA	NA	NA	NG	+	NG	NG	NG	NA	NG	NA
--Ephedrine	2-15 mg for IV	NA	NA	NG	2-4mg	NA	NG	+	+	NG	NA	NA	NA	NA	+	+	+	NG	NG	NA	NG	NA
1.5 Prophylactic vasopressor infusion should not be recommended in pre-eclampsia	Agree	NA	NA	NG	+	NA	NG	NG	NG	NG	NA	NA	NA	NA	NG	+	NG	NG	NG	NA	NG	NA
**2. Inadequate neuraxial anesthesia**	Provided	NA	NA	NA	NA	NA	NA	NA	+	NA	NA	NA	NA	NA	NA	NA	+	NA	NA	+	+	+
2.1 Block assessment	Provided	NA	NA	NA	NA	NA	NA	NA	NG	NA	NA	NA	NA	NA	NA	NA	NG	NA	NA	+	+	NG
-Testing sensory block after the evidence of motor block	Agree	NA	NA	NA	NA	NA	NA	NA	NG	NA	NA	NA	NA	NA	NA	NA	NG	NA	NA	+	+	NG
-Check the block relatively fixed	Agree	NA	NA	NA	NA	NA	NA	NA	NG	NA	NA	NA	NA	NA	NA	NA	NG	NA	NA	+	NG	NG

For peer review only

time intervals																						
-Perform the first check early enough to allow positional changes	Agree	NA	NA	NA	NA	NA	NA	NA	NG	NA	NA	NA	NA	NA	NA	NA	NG	NA	NA	+	NG	NG
2.2 Rescue for inadequate neuraxial block (change anesthesia method)	Provided	NA	NA	NA	NA	NA	NA	NA	+	NA	NA	NA	NA	NA	NA	NA	NG	NA	NA	+	+	NG
-A second neuraxial technique (in the case of spinal anesthesia)	Agree	NA	NA	NA	NA	NA	NA	NA	+	NA	NA	NA	NA	NA	NA	NA	+	NA	NA	+	+	NG
-Extending the neuraxial technique (in the case of combined spinal–epidural)	Agree	NA	NA	NA	NA	NA	NA	NA	+	NA	NA	NA	NA	NA	NA	NA	NG	NA	NA	+	+	NG
-Above options are not possible, general anesthesia should be recommended	Agree	NA	NA	NA	NA	NA	NA	NA	+	NA	NA	NA	NA	NA	NA	NA	NG	NA	NA	+	+	NG
2.3 Rescue for inadequate neuraxial block (change anesthetic)	Provided	NA	NA	NA	NA	NA	NA	NA	+	NA	NA	NA	NA	NA	NA	NA	NG	NA	NA	+	+	NG

For peer review only

-Nitrous oxide and oxygen alone	Disagree	NA	NA	NA	NA	NA	NA	NA	NG	NA	NA	NA	NA	NA	NA	NA	NG	NA	NA	+	NG	NG
-Continue with neuraxial block, consider repeated boluses of fast acting opioids	Agree	NA	NA	NA	NA	NA	NA	NA	NG	NA	NA	NA	NA	NA	NA	NA	NG	NA	NA	+	NG	NG
--Fentanyl	25-50 µg	NA	NA	NA	NA	NA	NA	NA	NG	NA	NA	NA	NA	NA	NA	NA	NG	NA	NA	+	NG	NG
--Alfentanil	250-500 µg	NA	NA	NA	NA	NA	NA	NA	NG	NA	NA	NA	NA	NA	NA	NA	NG	NA	NA	+	NG	NG
--Ketamine	10 mg boluses	NA	NA	NA	NA	NA	NA	NA	NG	NA	NA	NA	NA	NA	NA	NA	NG	NA	NA	+	10 mg or 0.15 mg .kg^{-1}	NG
-Do not treat pain with anxiolytics	Agree	NA	NA	NA	NA	NA	NA	NA	NG	NA	NA	NA	NA	NA	NA	NA	NG	NA	NA	+	NG	NG
-Above options are not possible, general anesthesia should be recommended	Agree	NA	NA	NA	NA	NA	NA	NA	+	NA	NA	NA	NA	NA	NA	NA	NG	NA	NA	+	+	NG
**3. Post dural puncture headache (PDPH)**	Provided	NA	NA	NA	NA	NA	NA	NA	+	NA	NA	NA	NA	NA	NA	NA	NA	NA	NA	NA	+	+/-
3.1 Measures be used to prevent PDPH	Provided	NA	NA	NA	NA	NA	NA	NA	NG	NA	NA	NA	NA	NA	NA	NA	NA	NA	NA	NA	+/-	NG
-Noncutting	Agree	NA	NA	NA	NA	NA	NA	NA	NG	NA	NA	NA	NA	NA	NA	NA	NA	NA	NA	NA	+	NG

For peer review only - http://bmjopen.bmj.com/site/about/guidelines.xhtml

spinal needles are associated with decreased PDPH risk																						
-An intrathecal catheter may be considered to provide anesthesia or analgesia	Agree	NA	NA	NA	NA	NA	NA	NA	NG	NA	NA	NA	NA	NA	NA	NA	NA	NA	NA	NA	+	NG
-A prophylactic epidural blood patch is not recommended as routine	Agree	NA	NA	NA	NA	NA	NA	NA	NG	NA	NA	NA	NA	NA	NA	NA	NA	NA	NA	NA	NG	NG
-Bed rest is not routinely recommended as prophylaxis against PDPH	Agree	NA	NA	NA	NA	NA	NA	NA	NG	NA	NA	NA	NA	NA	NA	NA	NA	NA	NA	NA	NG	NG
-Routine injection of substance to prevent PDPH is not recommended	Agree	NA	NA	NA	NA	NA	NA	NA	NG	NA	NA	NA	NA	NA	NA	NA	NA	NA	NA	NA	NG	NG
-Routine systemic drug for PDPH prophylaxis is not	Agree	NA	NA	NA	NA	NA	NA	NA	NG	NA	NA	NA	NA	NA	NA	NA	NA	NA	NA	NA	NG	NG

For peer review only

recommended																						
3.2 Measures be used to treat PDPH	Provided	NA	NA	NA	NA	NA	NA	NA	NG	NA	NA	NA	NA	NA	NA	NA	NA	NA	NA	NA	+/-	NG
-Epidural blood patch	Agree	NA	NA	NA	NA	NA	NA	NA	+	NA	NA	NA	NA	NA	NA	NA	NA	NA	NA	NA	+	NG
-The routine use of bed rest to treat PDPH	Disagree	NA	NA	NA	NA	NA	NA	NA	NG	NA	NA	NA	NA	NA	NA	NA	NA	NA	NA	NA	NG	NG
-Adequate hydration should be maintained with oral fluids or intravenous	Agree	NA	NA	NA	NA	NA	NA	NA	NG	NA	NA	NA	NA	NA	NA	NA	NA	NA	NA	NA	+	NG
-Routine use of abdominal binders or aromatherapy to treat PDPH	Disagree	NA	NA	NA	NA	NA	NA	NA	NG	NA	NA	NA	NA	NA	NA	NA	NA	NA	NA	NA	NG	NG
-Regular multimodal analgesia	Agree	NA	NA	NA	NA	NA	NA	NA	NG	NA	NA	NA	NA	NA	NA	NA	NA	NA	NA	NA	+/-	NG
-Short-term use of opioids if regular multimodal analgesia is ineffective	Agree	NA	NA	NA	NA	NA	NA	NA	NG	NA	NA	NA	NA	NA	NA	NA	NA	NA	NA	NA	NG	NG
-Long-term opioid use is not recommended in	Agree	NA	NA	NA	NA	NA	NA	NA	NG	NA	NA	NA	NA	NA	NA	NA	NA	NA	NA	NA	NG	NG

For peer review only - http://bmjopen.bmj.com/site/about/guidelines.xhtml

the treatment of PDPH																						
-Caffeine offered in the first 24h with a maximum dose of 900 mg per day	Agree	NA	NA	NA	NA	NA	NA	NA	NG	NA	NA	NA	NA	NA	NA	NA	NA	NA	NA	NA	+/-	NG
-Caffeine offered 200-300 mg if breastfeeding	Agree	NA	NA	NA	NA	NA	NA	NA	NG	NA	NA	NA	NA	NA	NA	NA	NA	NA	NA	NA	NG	NG
-Routine use of hydrocortisone, adrenocorticotropic hormone or cosyntropin	Disagree	NA	NA	NA	NA	NA	NA	NA	NG	NA	NA	NA	NA	NA	NA	NA	NA	NA	NA	NA	+/-	NG
-Routine use of theophylline, neostigmine or atropine, piritramide, and gabapentin	Disagree	NA	NA	NA	NA	NA	NA	NA	NG	NA	NA	NA	NA	NA	NA	NA	NA	NA	NA	NA	NG	NG
-Routine use of acupuncture to treat PDPH	Disagree	NA	NA	NA	NA	NA	NA	NA	NG	NA	NA	NA	NA	NA	NA	NA	NA	NA	NA	NA	NG	NG
-Routine use of sphenopalatine ganglion blocks to treat PDPH	Disagree	NA	NA	NA	NA	NA	NA	NA	NG	NA	NA	NA	NA	NA	NA	NA	NA	NA	NA	NA	+	NG

For peer review only

-Greater occipital nerve blocks be offered to patients with PDPH	Agree	NA	NA	NA	NA	NA	NA	NA	NG	NA	NA	NA	NA	NA	NA	NA	NA	NA	NA	NA	NG	NG
-The use of spinal and epidural morphine to treat PDPH	Disagree	NA	NA	NA	NA	NA	NA	NA	NG	NA	NA	NA	NA	NA	NA	NA	NA	NA	NA	NA	NG	NG
-Routine use of epidural dextran, gelatine, or hydroxyethyl starch to treat PDPH	Disagree	NA	NA	NA	NA	NA	NA	NA	NG	NA	NA	NA	NA	NA	NA	NA	NA	NA	NA	NA	NG	NG
-Routine use of fibrin glue to treat PDPH	Disagree	NA	NA	NA	NA	NA	NA	NA	NG	NA	NA	NA	NA	NA	NA	NA	NA	NA	NA	NA	NG	NG
-Fibrin glue should be reserved for management of PDPH refractory to EBP	Agree	NA	NA	NA	NA	NA	NA	NA	NG	NA	NA	NA	NA	NA	NA	NA	NA	NA	NA	NA	NG	NG
-Fibrin glue be used when autologous blood injection is contraindicated	Agree	NA	NA	NA	NA	NA	NA	NA	NG	NA	NA	NA	NA	NA	NA	NA	NA	NA	NA	NA	NG	NG

For peer review only

+: consistent the reference with content;
+/-: partial1y consistent the reference with content;
-: inconsistent the reference with content;
NA: not applicable;
NG: not given.

For peer review only

Supplementary Table 15. Summary of recommendations for the monitoring and management of obstetric and anesthetic emergencies by included guidance document.

		OG-OG-CMA_2014	OG-A-CMA_2016	ASA_2016	AAGBI_2017	ERAS_2018 (Part 1)	ERAS_2018 (Part 2)	ERAS_2018 (Part 3)	ACOG_2019	SOGC_2019	SOGC_2022	SOAP_2019	AA and OAA_2020	PROSPECT_2020	CHBSA_2020	CAA_2020	CAA_2020	SOAP_2020	NICE_2021	OAA_2022	ANNA_2023	RCoA_2023

For peer review only - http://bmjopen.bmj.com/site/about/guidelines.xhtml

Recommendations	Reference contents																					
**1. Resources for management of Hemorrhagic emergencies**	Provided	NA	NA	+	NA	NA	NA	NA	NA	NA	NA	NA	NA	NA	NA	+/-	NA	NA	NA	NA	+	+/-
-Large-bore IV catheters	Agree	NA	NA	+	NA	NA	NA	NA	NA	NA	NA	NA	NA	NA	NA	+	NA	NA	NA	NA	+	NG
-Fluid warmer	Agree	NA	NA	+	NA	NA	NA	NA	NA	NA	NA	NA	NA	NA	NA	NG	NA	NA	NA	NA	NG	NG
-Forced-air body warmer	Agree	NA	NA	+	NA	NA	NA	NA	NA	NA	NA	NA	NA	NA	NA	NG	NA	NA	NA	NA	NG	NG
-Availability of blood bank resources	Agree	NA	NA	+	NA	NA	NA	NA	NA	NA	NA	NA	NA	NA	NA	NG	NA	NA	NA	NA	+	NG
-Massive transfusion protocol (type-specific, or O-negative blood, or salvage)	Agree	NA	NA	+	NA	NA	NA	NA	NA	NA	NA	NA	NA	NA	NA	NG	NA	NA	NA	NA	+	NG
-Equipment for infusing IV fluids and blood products rapidly	Agree	NA	NA	+	NA	NA	NA	NA	NA	NA	NA	NA	NA	NA	NA	NG	NA	NA	NA	NA	NG	NG
**2. Equipment for management of airway emergencies**	Provided	NA	NA	+	NA	NA	NA	NA	+	+/-	NA	NA	NA	NA	NA	+	NA	NA	+	NA	+/-	+/-
2.1 Laryngoscope and assorted blades	Agree	NA	NA	+	NA	NA	NA	NA	+	NG	NA	NA	NA	NA	NA	NG	NA	NA	NG	NA	+	NG
2.2 Endotracheal tubes, with stylets	Agree	NA	NA	+	NA	NA	NA	NA	+	NG	NA	NA	NA	NA	NA	NG	NA	NA	NG	NA	+	NG

For peer review only - http://bmjopen.bmj.com/site/about/guidelines.xhtml

2.3 Oxygen source	Agree	NA	NA	+	NA	NA	NA	NA	NG	NG	NA	NA	NA	NA	NA	NG	NA	NA	+	NA	+	NG
2.4 Suction source with tubing and tonsil suction tip	Agree	NA	NA	+	NA	NA	NA	NA	NG	NG	NA	NA	NA	NA	NA	NG	NA	NA	+	NA	+	NG
2.5 Self-inflating bag and mask for positive-pressure ventilation	Agree	NA	NA	+	NA	NA	NA	NA	NG	NG	NA	NA	NA	NA	NA	NG	NA	NA	NG	NA	+	NG
2.6 Medications for blood pressure support, muscle relaxation, and hypnosis	Agree	NA	NA	+	NA	NA	NA	NA	NG	NG	NA	NA	NA	NA	NA	NG	NA	NA	NA	NA	NG	NG
2.7 Tracheal intubation has failed protocol	Agree	NA	NA	+	NA	NA	NA	NA	NG	NG	NA	NA	NA	NA	NA	+	NA	NA	+	NA	+	NG
**3. Cardiopulmonary Resuscitation**	Provided	NA	NA	+	NA	NA	NA	NA	NA	NA	NA	NA	NA	NA	NA	NA	NA	NA	NA	NA	+/-	+/-
3.1 Basic and advanced life-support equipment	Agree	NA	NA	+	NA	NA	NA	NA	NA	NA	NA	NA	NA	NA	NA	NA	NA	NA	NA	NA	+	NG
3.2 Cardiac arrest occurs	Provided	NA	NA	+	NA	NA	NA	NA	NA	NA	NA	NA	NA	NA	NA	NA	NA	NA	NA	NA	+	NG
-Uterine displacement (usually left displacement) should be maintained	Agree	NA	NA	+	NA	NA	NA	NA	NA	NA	NA	NA	NA	NA	NA	NA	NA	NA	NA	NA	+	NG
-If circulation is not restored within 4 min, cesarean should be performed	Agree	NA	NA	+	NA	NA	NA	NA	NA	NA	NA	NA	NA	NA	NA	NA	NA	NA	NA	NA	4-5 min	NG
**4. Emergency cesarean delivery**	Provided	NA	NA	+	NA	NA	NA	NA	+	NA	NA	NA	NA	NA	NA	NA	NA	NA	+	NA	+/-	+/-
4.1 Response times	30 or 75	NA	NA	NG	NA	NA	NA	NA	NG	NA	NA	NA	NA	NA	NA	NA	NA	NA	+	NA	NG	NG

For peer review only - http://bmjopen.bmj.com/site/about/guidelines.xhtml

limited	minutes																					
4.2 Anesthesia methods recommendation	Provided	NA	NA	+/-	NA	NA	NA	NA	+	NA	NA	NA	NA	NA	NA	NA	NA	NA	+/-	NA	NG	NG
-Spinal	Agree	NA	NA	+/-	NA	NA	NA	NA	+	NA	NA	NA	NA	NA	NA	NA	NA	NA	NG	NA	NG	NG
-Epidural	Agree	NA	NA	+/-	NA	NA	NA	NA	+	NA	NA	NA	NA	NA	NA	NA	NA	NA	NG	NA	NG	NG
-Combined spinal–epidural analgesia	Agree	NA	NA	+/-	NA	NA	NA	NA	+	NA	NA	NA	NA	NA	NA	NA	NA	NA	NG	NA	NG	NG
-General anesthesia	Agree	NA	NA	+/-	NA	NA	NA	NA	+	NA	NA	NA	NA	NA	NA	NA	NA	NA	NG	NA	NG	NG

+/-: partial1y consistent the reference with content;

-: inconsistent the reference with content;

NA: not applicable;

NG: not given.

For peer review only

For peer review only - http://bmjopen.bmj.com/site/about/guidelines.xhtml

■ Consistent with the reference content
■ Inconsistent with the reference content
■ Partially consistent with the reference content
■ Not given
■ Not applicable

Recommendations	Reference contents	OG-OG-CMA_2014	OG-A-CMA_2016	ASA_2016	AAGBI_2017	ERAS_2018 (Part 1)	ERAS_2018 (Part 2)	ERAS_2018 (Part 3)	ACOG_2019	SOGC_2019	SOGC_2022	SOAP_2019
**Peri-anesthetic Evaluation and Preparation**												
**1. History and physical examination before providing anesthetic care**	Yes	Consistent	Consistent	Consistent	Not applicable	Not given	Not applicable	Not applicable	Consistent	Not applicable	Not applicable	Not applicable
1.1 Maternal health and anesthetic history	Agree	Consistent	Consistent	Consistent	Not applicable	Not given	Not applicable	Not applicable	Consistent	Not applicable	Not applicable	Not applicable
1.2 Relevant obstetric history	Agree	Consistent	Consistent	Consistent	Not applicable	Not given	Not applicable	Not applicable	Not given	Not applicable	Not applicable	Not applicable
1.3 Baseline blood pressure measurement	Agree	Consistent	Consistent	Consistent	Not applicable	Not given	Not applicable	Not applicable	Not given	Not applicable	Not applicable	Not applicable
1.4 Airway examination	Agree	Not given	Consistent	Consistent	Not applicable	Not given	Not applicable	Not applicable	Consistent	Not applicable	Not applicable	Not applicable
1.5 Heart and lung examination	Agree	Not given	Not given	Consistent	Not applicable	Not given	Not applicable	Not applicable	Consistent	Not applicable	Not applicable	Not applicable
1.6 When a neuraxial anesthetic is planned, examine the patient’s back	Agree	Not given	Consistent	Consistent	Not applicable	Not given	Not applicable	Not applicable	Consistent	Not applicable	Not applicable	Not applicable
1.7 Consultation between the obstetrician and the anesthesiologist	Agree	Consistent	Not given	Consistent	Not applicable	Consistent	Not applicable	Not applicable	Consistent	Not applicable	Not applicable	Not applicable
**2. Preoperative communication**	Encouraged	Consistent	Not given	Consistent	Not applicable	Consistent	Not applicable	Not applicable	Not given	Not applicable	Not applicable	Not applicable
**3. Intrapartum platelet count**	Yes	Consistent	Consistent	Consistent	Not applicable	Not applicable	Not applicable	Not applicable	Consistent	Not applicable	Not applicable	Not applicable
-Routine	Agree	Consistent	Consistent	Consistent	Not applicable	Not applicable	Not applicable	Not applicable	Consistent	Not applicable	Not applicable	Not applicable
-Individualized	Agree	Inconsistent	Inconsistent	Consistent	Not applicable	Not applicable	Not applicable	Not applicable	Consistent	Not applicable	Not applicable	Not applicable
**4. Blood type and screen**	Yes	Consistent	Consistent	Consistent	Not applicable	Not applicable	Not applicable	Not applicable	Not given	Not applicable	Not applicable	Not applicable
-Routine	Agree	Consistent	Consistent	Inconsistent	Not applicable	Not applicable	Not applicable	Not applicable	Not given	Not applicable	Not applicable	Not applicable
-Individualized	Agree	Inconsistent	Inconsistent	Consistent	Not applicable	Not applicable	Not applicable	Not applicable	Not given	Not applicable	Not applicable	Not applicable
**5. Blood cross-match**	Yes	Consistent	Not given	Consistent	Not applicable	Not applicable	Not applicable	Not applicable	Not given	Not applicable	Not applicable	Not applicable
-Routine	Agree	Consistent	Not given	Inconsistent	Not applicable	Not applicable	Not applicable	Not applicable	Not given	Not applicable	Not applicable	Not applicable
-Individualized	Agree	Not given	Not given	Consistent	Not applicable	Not applicable	Not applicable	Not applicable	Not given	Not applicable	Not applicable	Not applicable
6. Peri-anesthetic recording of fetal heart rate	Yes	Consistent	Consistent	Consistent	Not applicable	Not applicable	Not applicable	Not applicable	Consistent	Consistent	Consistent	Not applicable
**7. Aspiration prevention**	Provided	Consistent	Not given	Consistent	Not applicable	Consistent	Not applicable	Not applicable	Not applicable	Consistent	Not applicable	Not applicable
7.1 Clear liquids	Up to 2 h	Not given	Not given	Consistent	Not applicable	Consistent	Not applicable	Not applicable	Not applicable	Not given	Not applicable	Not applicable

7.2 Solids	6-8 h
7.3 Antacids (surgical procedures-caesarean delivery or postpartum tubal ligation)	Agree
7.4 H_2-receptor antagonists (surgical procedures)	Agree
7.5 Metoclopramide (surgical procedures)	Agree
**8. Preoperative heparin or low-molecular-weight heparin**	Yes
8.1 Prophylactic subcutaneous unfractionated heparin (<5,000 units twice daily)	Provided
-Contraindication to neuraxial techniques	No
-Catheter placement or removal since last dose	4-6 hours
8.2 Intermediate unfractionated heparin (75,000-100,000 units)	Provided
-Catheter placement or removal since last dose	> 12 hours
8.3 High-dose unfractionated heparin (total daily dose greater than 20,000 units)	Provided
-Catheter placement or removal since last dose	> 24 hours
-With the activated partial thromboplastin time being within normal range	Yes
**anesthetic Care for labor and Vaginal Delivery**	
**1. Equipment, facilities, and support personnel**	Agree
**2. Timing of neuraxial analgesia and outcome of labor**	Provided
2.1 Less 5 cm dilation	Agree
2.2 Offer neuraxial analgesia on an individualized basis	Agree
**3. Neuraxial analgesia and trial of labor after prior caesarean delivery**	Agree
**4. Continuous infusion epidural analgesia**	Provided
4.1 Using local anesthetic	Agree
4.2 Add opioid	Agree
4.3 Platelet concentration corresponding to contraindications to puncture	Less than $70*10^9$/L
4.4 Puncture location	L_{2-3} or L_{3-4}
4.5 Analgesic concentrations	Provided
-Bupivacaine	Provided
--Loading dose	0.04-0.125%
--Maintenance dose	0.05-0.125%
-Ropivacaine	Provided
--Loading dose	0.0625-0.15%

--Maintenance dose	0.0625-0.125%
-Levobupivacaine	Provided
--Loading dose	0.04-0.125%
--Maintenance dose	0.05-0.125%
4.6 Opioids concentrations	Provided
-Fentanyl	Provided
--Loading dose	0.5-2 μg/ml
--Maintenance dose	1-2 μg/ml
-Sufentanyl	Provided
--Loading dose	0.2~0.6 μg/ml
--Maintenance dose	0.3~0.6 μg/ml
**5. Combined spinal–epidural analgesia**	Provided
5.1 Using local anesthetic	Agree
5.2 Add opioid	Agree
5.3 Platelet concentration corresponding to contraindications to puncture	Less than $70*10^9$/L
5.4 Puncture location	L_{2-3} or L_{3-4}
5.5 Epidural anesthetics (consistent with 4.5 and 4.6)	Provided
5.6 Spinal anesthetics	Provided
-Protocol 1	Provided
--Single sufentanyl	2.5-7 μg
--Single ropivacaine	2.3-3 mg
--Combined sufentanyl and ropivacaine	2.5 μg / 2.5 mg
-Protocol 2	Provided
--Single fentanyl	15-25 μg
--Single bupivacaine	2.0-2.5 mg
--Combined fentanyl and bupivacaine	2.5 μg / 2.0 mg
**6. Single-injection spinal**	Provided
6.1 Using local anesthetic	Agree
6.2 Add opioid	Agree
6.3 Platelet concentration corresponding to contraindications to puncture	Less than $50*10^9$/L

For peer review

6.4 Puncture location	$L_{3\text{-}4}$
6.6 Analgesic concentrations (consistent with 5.6)	Agree
6.7 Spinal needles	Provided
-Cutting-bevel	Disagree
-Pencil-point	Agree
**7. Patient-controlled epidural analgesia (for labor analgesia)**	Agree
**anesthetic Care for caesarean Delivery**	
**1. Equipment, facilities, and support personnel**	Agree
**2. Continuous infusion epidural analgesia**	Provided
2.1 Using local anesthetic	Agree
2.2 Add opioid	Agree
2.3 Platelet concentration corresponding to contraindications to puncture	Less than $70*10^9$/L
2.4 Puncture location	$L_{1\text{-}2}$ or $L_{2\text{-}3}$
2.5 Recommend level of block	$T_{6\text{-}4}$
2.6 Analgesic concentrations	Provided
-Lidocaine	1.5%-2%
-Bupivacaine	0.50%
-Ropivacaine	0.5%-0.75%
-Levobupivacaine	0.5%-0.75%
-Cloprocaine	3%
2.7 Prevention of local anesthetic poisoning	Provided
-Withdrawal before injection	Agree
-Administration of test dose	Agree
-Addition of epinephrine	Agree
**3. Single-injection spinal**	Provided
3.1 Using local anesthetic	Agree
3.2 Add opioid	Agree
3.3 Platelet concentration corresponding to contraindications to puncture	Less than $70*10^9$/L
3.4 Puncture location	$L_{3\text{-}4}$
3.5 Recommend level of block	$T_{6\text{-}4}$

3.6 Analgesic concentrations	Provided
-Bupivacaine	5~15 mg
-Ropivacaine	10~20 mg
3.7 Spinal needles	Provided
-Cutting-bevel	Disagree
-Pencil-point	Agree
**4. Combined spinal–epidural analgesia**	Provided
4.1 Puncture location	Provided
-Single space technique	L_{3-4}
-Double-space technique	T_{12}-L_2/L_{3-5}
4.2 anesthetics (consistent with spinal or epidural)	Agree
**5. General anesthesia**	Provided
5.1 General anesthesia selected condition	Provided
-Profound fetal bradycardia	Agree
-Ruptured uterus	Agree
-Severe hemorrhage	Agree
-Severe placental abruption	Agree
-Umbilical cord prolapse	Agree
-Preterm footling breech	Agree
-Inability to place neuraxial anesthesia	
5.2 Intravenous anesthetics recommendation	Provided
-Thiopental sodium	4-5 mg/kg
-Propofol	1.5-2.5 mg/kg
-Etomidate	0.2-0.3 mg/kg
-Ketamine	0.5-1 mg/kg
5.3 Opioids analgesic recommendation	Provided
-Fentanyl	2-5 μg/kg
-Sufentanyl	0.2-0.5 μg/kg
-Remifentanyl	0.5-1 μg/kg for IV
-Remifentanyl	4 ng/kg for target

5.4 Muscle relaxants recommendation	Provided	Orange	Green	Orange	Green	Green	Green	Green	Blue	Green	Green	Green
-Succinylcholine	1-1.5 mg/kg	Orange	Green	Orange	Green	Green	Green	Green	Orange	Green	Green	Green
-Rocuronium	0.6-1.2 mg/kg	Orange	Green	Orange	Green	Green	Green	Green	Orange	Green	Green	Green
5.5 Induction-delivery interval	within 10 min	Orange	Green	Orange	Green	Green	Green	Green	Orange	Green	Green	Green
**Neurological Monitoring Associated With Obstetric Neuraxial Block**												
**1. Screening method (During the recovery phase) -Straight-leg raising**	Agree	Green	Green	Green	Green	Green	Green	Green	Green	Green	Green	Green
**2. Alerts incidences-unable to straight-leg raise at 4 h from the last dose**	Agree	Green	Green	Green	Green	Green	Green	Green	Green	Green	Green	Green
**Respiratory Depression Monitoring With Neuraxial Block With Opioids**												
**1. Frequency and modality of respiratory monitoring should be based on:**	Provided	Blue	Green	Green	Green	Green	Green	Green	Green	Green	Green	Blue
1.1 Low risk	Provided	Orange	Green	Green	Green	Green	Green	Green	Green	Green	Green	Blue
-Neuraxial morphine dose (intrathecal ≤ 0.15 mg or epidural ≤ 3 mg)	Agree	Orange	Green	Green	Green	Green	Green	Green	Green	Green	Green	Blue
1.2 High risk	Provided	Orange	Green	Green	Green	Green	Green	Green	Green	Green	Green	Blue
-Cardiopulmonary/neurological co-morbidity	Agree	Orange	Green	Green	Green	Green	Green	Green	Green	Green	Green	Blue
-Obesity (body mass index ≥ 40 kg/m^2)	Agree	Orange	Green	Green	Green	Green	Green	Green	Green	Green	Green	Blue
-Known or suspected obstructive sleep apnea	Agree	Orange	Green	Green	Green	Green	Green	Green	Green	Green	Green	Blue
-Chronic opioid use or abuse	Agree	Orange	Green	Green	Green	Green	Green	Green	Green	Green	Green	Blue
-Hypertension	Agree	Orange	Green	Green	Green	Green	Green	Green	Green	Green	Green	Blue
-Magnesium administration	Agree	Orange	Green	Green	Green	Green	Green	Green	Green	Green	Green	Blue
-Neuraxial morphine dose (intrathecal > 0.15 mg or epidural > 3 mg)	Agree	Orange	Green	Green	Green	Green	Green	Green	Green	Green	Green	Blue
1.3 Respiratory monitoring frequency and duration	Provided	Orange	Green	Green	Green	Green	Green	Green	Green	Green	Green	Blue
-Low risk	Provided	Orange	Green	Green	Green	Green	Green	Green	Green	Green	Green	Blue
--Routine postoperative vital sign monitoring	Agree	Orange	Green	Green	Green	Green	Green	Green	Green	Green	Green	Blue
-- q2h clinical assessment for 12 h	Agree	Orange	Green	Green	Green	Green	Green	Green	Green	Green	Green	Blue
-High risk	Provided	Orange	Green	Green	Green	Green	Green	Green	Green	Green	Green	Blue
-- q1h clinical assessment for first 12 h	Agree	Orange	Green	Green	Green	Green	Green	Green	Green	Green	Green	Blue
-- q2h clinical assessment for first 12-24 h	Agree	Orange	Green	Green	Green	Green	Green	Green	Green	Green	Green	Blue
--Consider additional monitoring modalities (e.g., pulse oximetry, capnography)	Agree	Orange	Green	Green	Green	Green	Green	Green	Green	Green	Green	Blue
**Management of Obstetric and anesthetic Emergencies or Adverse Events**												
**1. Resources for management of hemorrhagic emergencies**	Provided	Green	Green	Blue	Green	Green	Green	Green	Green	Green	Green	Green
-Large-bore IV catheters	Agree	Green	Green	Blue	Green	Green	Green	Green	Green	Green	Green	Green
-Fluid warmer	Agree	Green	Green	Blue	Green	Green	Green	Green	Green	Green	Green	Green

-Forced-air body warmer	Agree	green	green	blue	green	green	green	green	green	green	green	green
-Availability of blood bank resources	Agree	green	green	blue	green	green	green	green	green	green	green	green
-Massive transfusion protocol (type-specific, or O-negative blood, or salvage)	Agree	green	green	blue	green	green	green	green	green	green	green	green
-Equipment for infusing IV fluids and blood products rapidly	Agree	green	green	blue	green	green	green	green	green	green	green	green
**2. Hypotension prevention strategies**	Provided	green	green	blue	blue	green	teal	blue	blue	blue	green	green
2.1 Blood pressure target of systolic arterial pressure	⩾ 90% of baseline obtained	green	green	yellow	blue	green	yellow	yellow	yellow	yellow	green	green
2.2 Position (uterine left-leaning)	Yes	green	green	blue	blue	green	yellow	yellow	yellow	yellow	green	green
2.3 Volume expansion (crystals or colloids)	500-1000 ml	green	green	blue	blue	green	blue	blue	blue	blue	green	green
2.4 Vasoactive drugs	Yes	green	green	blue	blue	green	yellow	blue	blue	yellow	green	green
-Pump infusion is recommended	Provided	green	green	blue	blue	green	yellow	yellow	yellow	yellow	green	green
-Type of drugs	Provided	green	green	blue	blue	green	yellow	blue	blue	yellow	green	green
--Phenylephrine	20-50 μg or 0.5 μg. kg^{-1}. min^{-1}	green	green	blue	blue	green	yellow	blue	blue	yellow	green	green
--Methoxymine	1-2 mg or 4 μg. kg-1. min-1	green	green	yellow	blue	green	yellow	yellow	yellow	yellow	green	green
--Noradrenalin	4-6 μg or 0.08 μg. kg-1. min-1	green	green	yellow	yellow	green	yellow	yellow	yellow	yellow	green	green
--Efedrina	5-15 mg for IV	green	green	yellow	blue	green	yellow	blue	blue	yellow	green	green
2.5 Prophylactic vasopressor infusion should not be recommended in pre-eclampsia	Agree	green	green	yellow	blue	green	yellow	yellow	yellow	yellow	green	green
**3. Equipment for management of airway emergencies**	Provided	green	green	blue	green	green	green	green	blue	teal	green	green
3.1 Laryngoscope and assorted blades	Agree	green	green	blue	green	green	green	green	blue	yellow	green	green
3.2 Endotracheal tubes, with stylets	Agree	green	green	blue	green	green	green	green	blue	yellow	green	green
3.3 Oxygen source	Agree	green	green	blue	green	green	green	green	yellow	yellow	green	green
3.4 Suction source with tubing and tonsil suction tip	Agree	green	green	blue	green	green	green	green	yellow	yellow	green	green
3.5 Self-inflating bag and mask for positive-pressure ventilation	Agree	green	green	blue	green	green	green	green	yellow	yellow	green	green
3.6 Medications for blood pressure support, muscle relaxation, and hypnosis	Agree	green	green	blue	green	green	green	green	yellow	yellow	green	green
3.7 Tracheal intubation has failed protocol	Agree	green	green	blue	green	green	green	green	yellow	yellow	green	green
**4. Cardiopulmonary Resuscitation**	Provided	green	green	blue	green	green	green	green	green	green	green	green
4.1 Basic and advanced life-support equipment	Agree	green	green	blue	green	green	green	green	green	green	green	green
4.2 Cardiac arrest occurs	Provided	green	green	blue	green	green	green	green	green	green	green	green
-Uterine displacement (usually left displacement) should be maintained	Agree	green	green	blue	green	green	green	green	green	green	green	green
-If circulation is not restored within 4 min, caesarean should be performed	Agree	green	green	blue	green	green	green	green	green	green	green	green
**5. Emergency caesarean delivery**	Provided	green	green	blue	green	green	green	green	blue	green	green	green

5.1 Response times limited	30 minutes
5.2 anesthesia methods recommendation	Provided
-Spinal	Agree
-Epidural	Agree
-Combined spinal–epidural analgesia	Agree
-General anesthesia	Agree
**6. Inadequate neuraxial anesthesia**	Provided
6.1 Block assessment	Provided
-Testing sensory block after the evidence of motor block	Agree
-Check the block at relatively fixed time intervals	Agree
-Perform the first check early enough to allow positional changes	Agree
6.2 Rescue for inadequate neuraxial block (change anesthesia method)	Provided
-A second neuraxial technique (in the case of spinal anesthesia)	Agree
-Extending the neuraxial technique (in the case of combined spinal–epidural)	Agree
-Above options are not possible, general anesthesia should be recommended	Agree
6.3 Rescue for inadequate neuraxial block (change anesthetic)	Provided
-Nitrous oxide and oxygen alone	Disagree
-Continue with neuraxial block, consider repeated boluses of fastacting opioids	Agree
--Fentanyl	25-50 μg
--Alfentanil	250-500 μg
--Ketamine	10 mg boluses
-Do not treat pain with anxiolytics	Agree
-Above options are not possible, general anesthesia should be recommended	Agree
**Management of Intra- or Postoperative Pain**	
**1. Implement strategies to minimize systemic opioid utilization**	Provided
**2. Neuraxial adjuvant drugs**	Provided
-Spinal anesthesia	Provided
--Morphine	50-100 μg
--Hydromorphone	75-150 μg
-Epidural or combined spinal-epidural technique.	Provided

For peer review only

--Morphine	2-3 mg
--Diamorphine	2-3 mg
**3. Others analgesic (after delivery)**	Provided
-Non-steroidal anti-inflammatory drug (e.g., paracetamol)	Agree
-A single dose of intravenous dexamethasone	Agree
**4. Local anesthetic infiltration**	Agree
**5. Continuous wound local anesthetic infusion**	Agree
**6. Fascial plane blocks (e.g., transversus abdominis plane, erector spinae plane)**	Agree
**7. Patient-controlled intravenous analgesia**	Agree

For peer review only

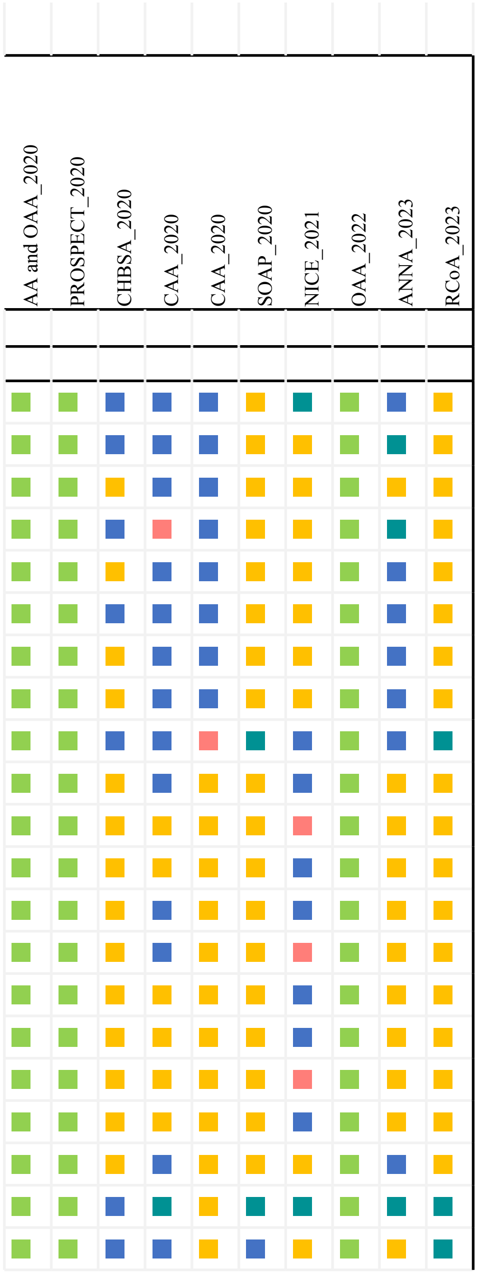

r peer review only

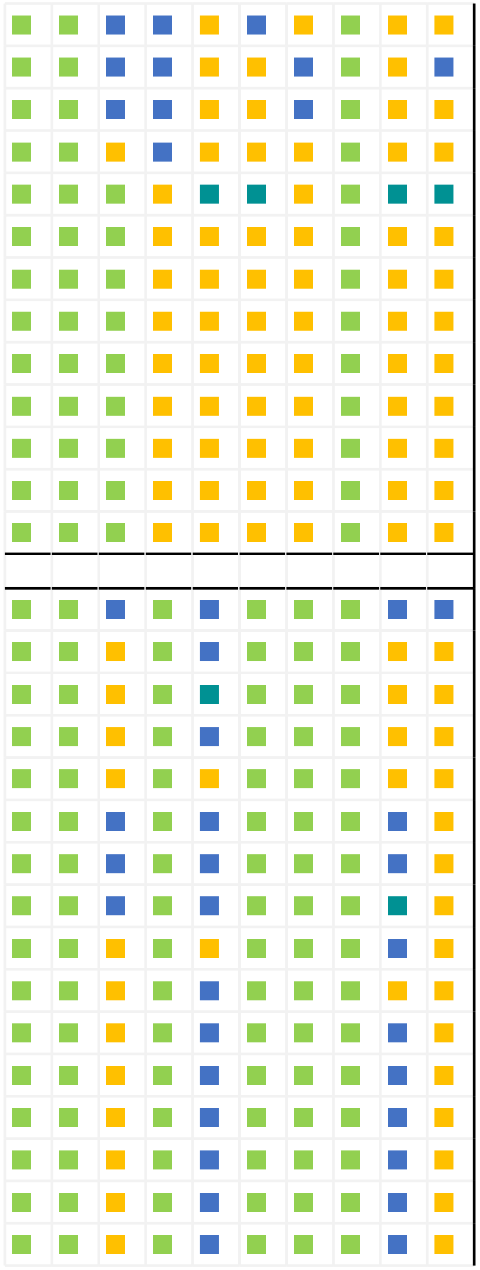

r peer review only

For peer review only - http://bmjopen.bmj.com/site/about/guidelines.xhtml

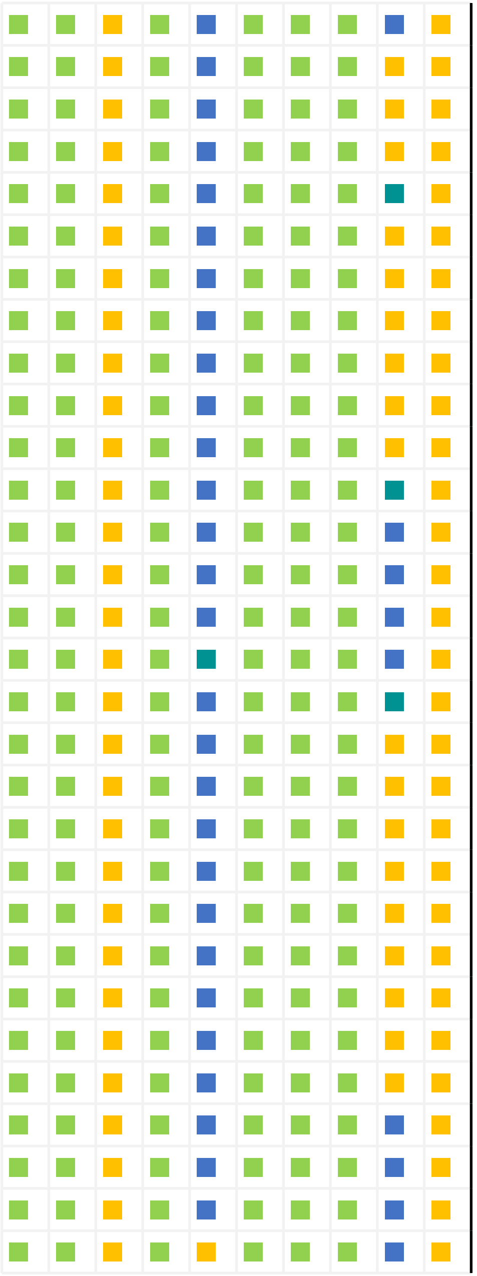

r peer review only

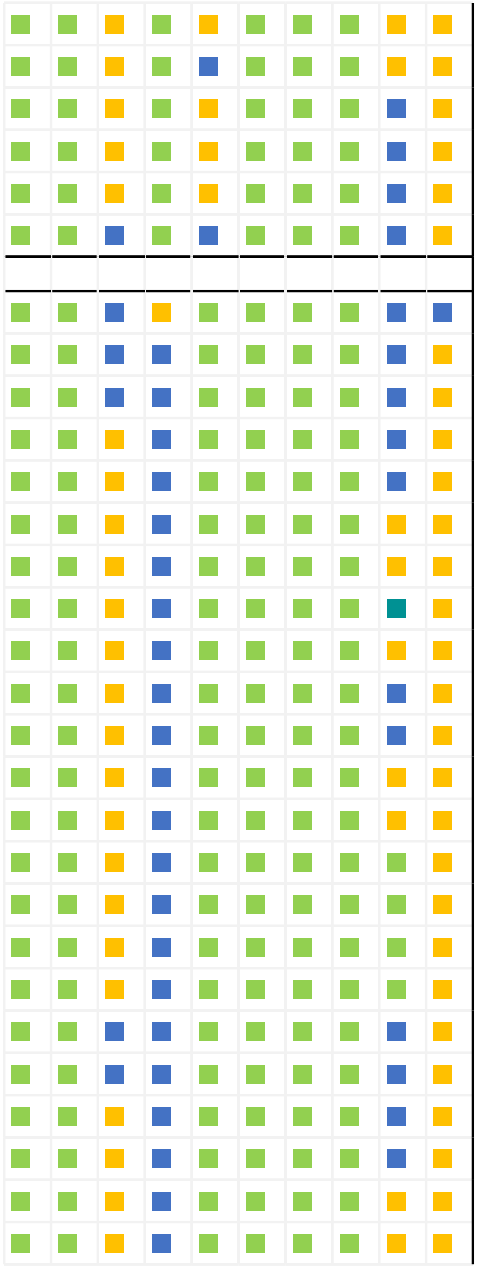

r peer review only

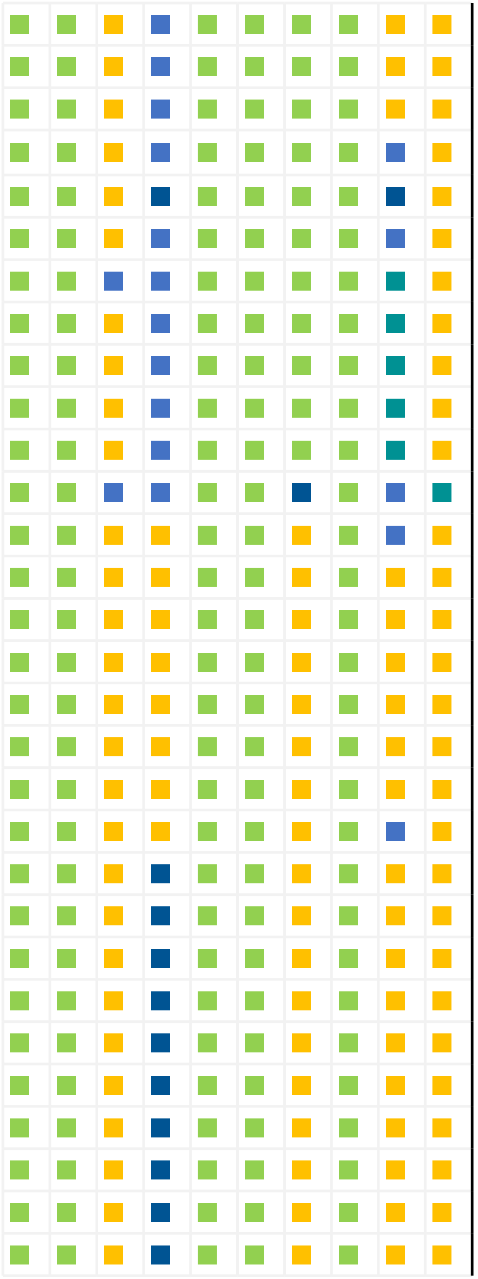

r peer review only

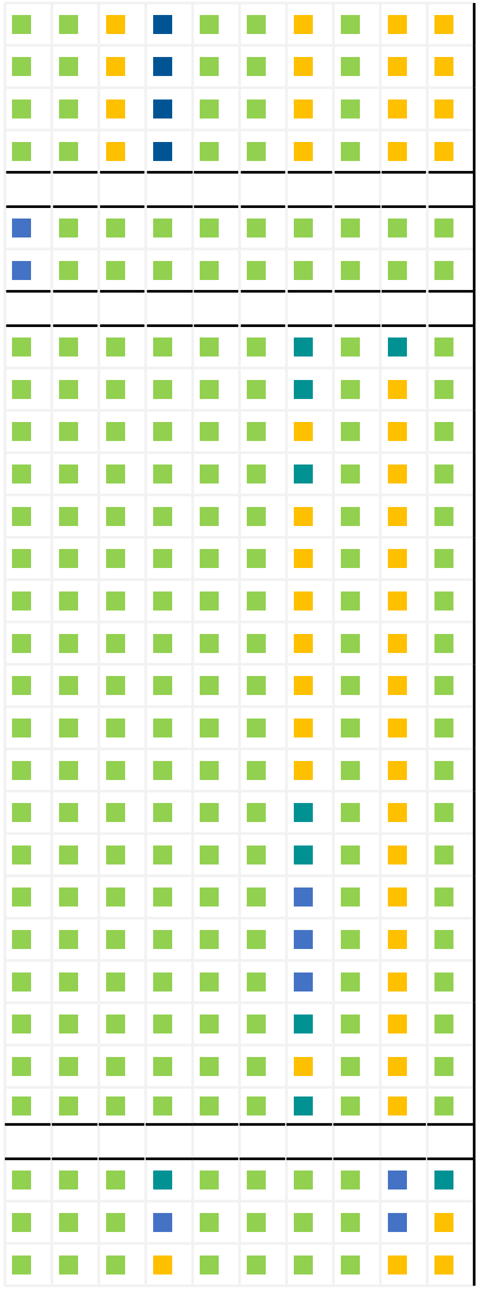

For peer review only

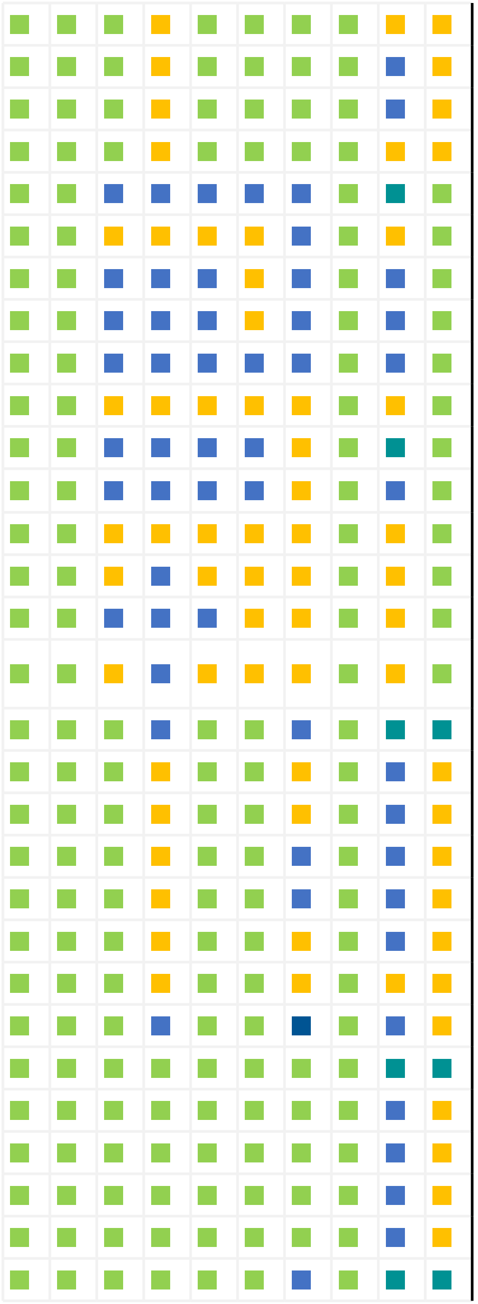

r peer review only

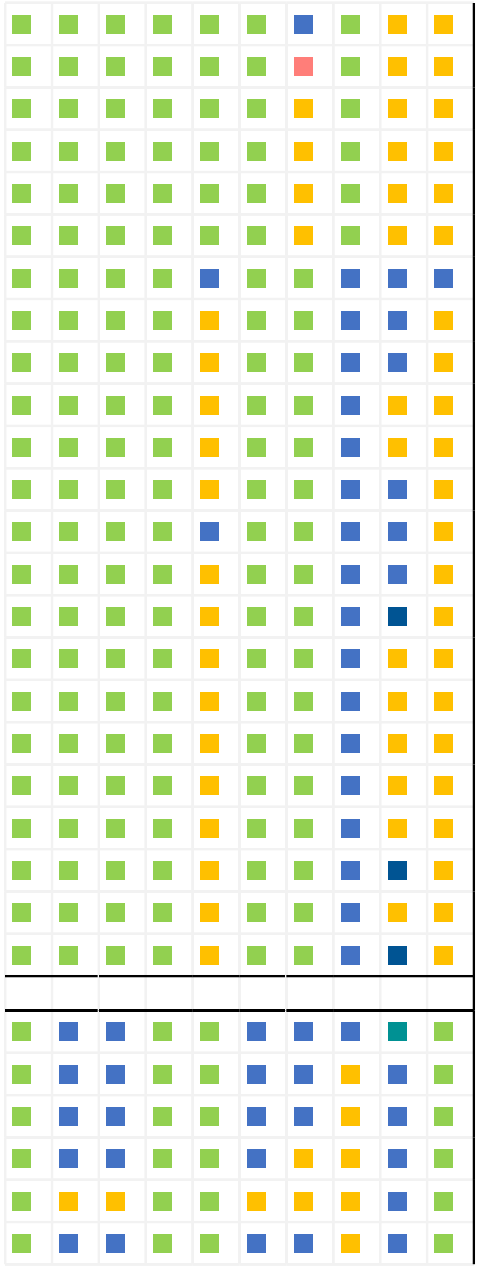

r peer review only

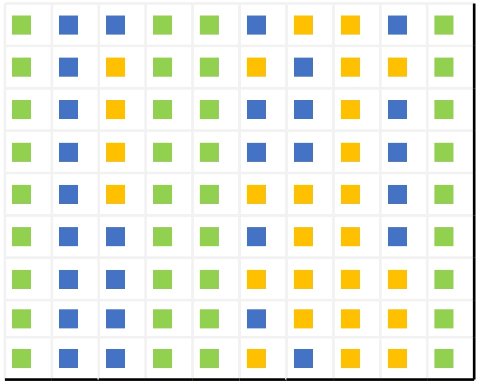

For peer review only

PRISMA 2020 Checklist

Section and Topic	Item #	Checklist item	Location where item is reported
**TITLE**			Page 1
Title	1	Identify the report as a systematic review.	
**ABSTRACT**			Page 2
Abstract	2	See the PRISMA 2020 for Abstracts checklist.	
**INTRODUCTION**			Page 5
Rationale	3	Describe the rationale for the review in the context of existing knowledge.	
Objectives	4	Provide an explicit statement of the objective(s) or question(s) the review addresses.	
**METHODS**			Page 6 to 9
Eligibility criteria	5	Specify the inclusion and exclusion criteria for the review and how studies were grouped for the syntheses.	
Information sources	6	Specify all databases, registers, websites, organisations, reference lists and other sources searched or consulted to identify studies. Specify the date when each source was last searched or consulted.	
Search strategy	7	Present the full search strategies for all databases, registers and websites, including any filters and limits used.	
Selection process	8	Specify the methods used to decide whether a study met the inclusion criteria of the review, including how many reviewers screened each record and each report retrieved, whether they worked independently, and if applicable, details of automation tools used in the process.	
Data collection process	9	Specify the methods used to collect data from reports, including how many reviewers collected data from each report, whether they worked independently, any processes for obtaining or confirming data from study investigators, and if applicable, details of automation tools used in the process.	
Data items	10a	List and define all outcomes for which data were sought. Specify whether all results that were compatible with each outcome domain in each study were sought (e.g. for all measures, time points, analyses), and if not, the methods used to decide which results to collect.	
	10b	List and define all other variables for which data were sought (e.g. participant and intervention characteristics, funding sources). Describe any assumptions made about any missing or unclear information.	
Study risk of bias assessment	11	Specify the methods used to assess risk of bias in the included studies, including details of the tool(s) used, how many reviewers assessed each study and whether they worked independently, and if applicable, details of automation tools used in the process.	
Effect measures	12	Specify for each outcome the effect measure(s) (e.g. risk ratio, mean difference) used in the synthesis or presentation of results.	
Synthesis methods	13a	Describe the processes used to decide which studies were eligible for each synthesis (e.g. tabulating the study intervention characteristics and comparing against the planned groups for each synthesis (item #5)).	
	13b	Describe any methods required to prepare the data for presentation or synthesis, such as handling of missing summary statistics, or data conversions.	
	13c	Describe any methods used to tabulate or visually display results of individual studies and syntheses.	
	13d	Describe any methods used to synthesize results and provide a rationale for the choice(s). If meta-analysis was performed, describe the model(s), method(s) to identify the presence and extent of statistical heterogeneity, and software package(s) used.	
	13e	Describe any methods used to explore possible causes of heterogeneity among study results (e.g. subgroup analysis, meta-regression).	
	13f	Describe any sensitivity analyses conducted to assess robustness of the synthesized results.	
Reporting bias assessment	14	Describe any methods used to assess risk of bias due to missing results in a synthesis (arising from reporting biases).	
Certainty	15	Describe any methods used to assess certainty (or confidence) in the body of evidence for an outcome.	

For peer review only - http://bmjopen.bmj.com/site/about/guidelines.xhtml

For peer review only

PRISMA 2020 Checklist

Section and Topic	Item #	Checklist item	Location where item is reported
assessment			
**RESULTS**			Page 10 to 21
Study selection	16a	Describe the results of the search and selection process, from the number of records identified in the search to the number of studies included in the review, ideally using a flow diagram.	
	16b	Cite studies that might appear to meet the inclusion criteria, but which were excluded, and explain why they were excluded.	
Study characteristics	17	Cite each included study and present its characteristics.	
Risk of bias in studies	18	Present assessments of risk of bias for each included study.	
Results of individual studies	19	For all outcomes, present, for each study: (a) summary statistics for each group (where appropriate) and (b) an effect estimate and its precision (e.g. confidence/credible interval), ideally using structured tables or plots.	
Results of syntheses	20a	For each synthesis, briefly summarise the characteristics and risk of bias among contributing studies.	
	20b	Present results of all statistical syntheses conducted. If meta-analysis was done, present for each the summary estimate and its precision (e.g. confidence/credible interval) and measures of statistical heterogeneity. If comparing groups, describe the direction of the effect.	
	20c	Present results of all investigations of possible causes of heterogeneity among study results.	
	20d	Present results of all sensitivity analyses conducted to assess the robustness of the synthesized results.	
Reporting biases	21	Present assessments of risk of bias due to missing results (arising from reporting biases) for each synthesis assessed.	
Certainty of evidence	22	Present assessments of certainty (or confidence) in the body of evidence for each outcome assessed.	
**DISCUSSION**			Page 21 to 26
Discussion	23a	Provide a general interpretation of the results in the context of other evidence.	
	23b	Discuss any limitations of the evidence included in the review.	
	23c	Discuss any limitations of the review processes used.	
	23d	Discuss implications of the results for practice, policy, and future research.	
**OTHER INFORMATION**			Page 27
Registration and protocol	24a	Provide registration information for the review, including register name and registration number, or state that the review was not registered.	
	24b	Indicate where the review protocol can be accessed, or state that a protocol was not prepared.	
	24c	Describe and explain any amendments to information provided at registration or in the protocol.	
Support	25	Describe sources of financial or non-financial support for the review, and the role of the funders or sponsors in the review.	
Competing interests	26	Declare any competing interests of review authors.	
Availability of data, code and other materials	27	Report which of the following are publicly available and where they can be found: template data collection forms; data extracted from included studies; data used for all analyses; analytic code; any other materials used in the review.	

For peer review only - http://bmjopen.bmj.com/site/about/guidelines.xhtml

PRISMA 2020 Checklist

From: Page MJ, McKenzie JE, Bossuyt PM, Boutron I, Hoffmann TC, Mulrow CD, et al. The PRISMA 2020 statement: an updated guideline for reporting systematic reviews. BMJ 2021;372:n71. doi: 10.1136/bmj.n71

For more information, visit: http://www.prisma-statement.org/

For peer review only

BMJ Open

Appraisal of clinical practice guidelines and consensus statements on obstetric anesthesia: A systematic review using the AGREE II instrument

Journal:	*BMJ Open*
Manuscript ID	bmjopen-2024-084759.R1
Article Type:	Original research
Date Submitted by the Author:	16-Apr-2024
Complete List of Authors:	Huang, Lu; West China Second Hospital of Sichuan University, Department of anesthesiology; Ministry of Education<br>Hu, Na; Sichuan University West China Second University Hospital, Department of Anesthesiology<br>Jiang, Ling; West China Second Hospital of Sichuan University, Department of anesthesiology; Ministry of Education<br>Xiong, Xinglong; Affiliated Hospital of Guizhou Medical University<br>Shi, Jing; The Affiliated Hospital of Guizhou Medical University<br>Chen, Dongxu; West China Second Hospital of Sichuan University, Department of anesthesiology; Ministry of Education
**Primary Subject Heading**:	Anaesthesia
Secondary Subject Heading:	Obstetrics and gynaecology
Keywords:	Anaesthesia in obstetrics < ANAESTHETICS, Pain management < ANAESTHETICS, PAIN MANAGEMENT

SCHOLARONE™
Manuscripts

I, the Submitting Author has the right to grant and does grant on behalf of all authors of the Work (as defined in the below author licence), an exclusive licence and/or a non-exclusive licence for contributions from authors who are: i) UK Crown employees; ii) where BMJ has agreed a CC-BY licence shall apply, and/or iii) in accordance with the terms applicable for US Federal Government officers or employees acting as part of their official duties; on a worldwide, perpetual, irrevocable, royalty-free basis to BMJ Publishing Group Ltd ("BMJ") its licensees and where the relevant Journal is co-owned by BMJ to the co-owners of the Journal, to publish the Work in this journal and any other BMJ products and to exploit all rights, as set out in our licence.

The Submitting Author accepts and understands that any supply made under these terms is made by BMJ to the Submitting Author unless you are acting as an employee on behalf of your employer or a postgraduate student of an affiliated institution which is paying any applicable article publishing charge ("APC") for Open Access articles. Where the Submitting Author wishes to make the Work available on an Open Access basis (and intends to pay the relevant APC), the terms of reuse of such Open Access shall be governed by a Creative Commons licence – details of these licences and which Creative Commons licence will apply to this Work are set out in our licence referred to above.

Other than as permitted in any relevant BMJ Author's Self Archiving Policies, I confirm this Work has not been accepted for publication elsewhere, is not being considered for publication elsewhere and does not duplicate material already published. I confirm all authors consent to publication of this Work and authorise the granting of this licence.

For peer review only

Appraisal of clinical practice guidelines and consensus statements on obstetric anesthesia: A systematic review using the AGREE II instrument

Lu Huang[1,2], Na Hu[1,2], Ling Jiang[1,2], Xing Long, Xiong[3], Jing Shi[3], Dong Xu Chen[1,2,*],

Affiliation Information for All Authors:

1. Department of anesthesiology, West China Second Hospital of Sichuan University, Sichuan Province, Chengdu, 610041, China
2. Key Laboratory of Birth Defects and Related Diseases of Women and Children (Sichuan University), Ministry of Education
3. Department of Anesthesiology, The Affiliated Hospital of Guizhou Medical University, Guiyang, China

Corresponding to:
Dong Xu Chen, PhD. Department of anesthesiology, West China Second Hospital of Sichuan University, Sichuan Province, Chengdu, 610041, China (e-mail: scucdx@foxmail.com)

Abstract

Objectives: Despite the publication of hundreds of trials on obstetric anesthesia, the management of these conditions remains suboptimal. We aimed to assess the quality and consistency of guidance documents for obstetric anesthesia.

Design: Systematic review and quality assessment using the appraisal of guidelines for research and evaluation (AGREE) II methodology.

Data sources: PubMed and EMBASE (8 June 2023), three Chinese academic databases, six guideline databases (7 June 2023), and Google and Google scholar (1 August 2023).

Eligibility criteria: We included the latest version of international and national/regional clinical practice guidelines and consensus statements for the anesthetic management of pregnant patients during labor, nonoperative delivery, operative delivery, and selected aspects of perioperative monitoring, postpartum care, and analgesia, published in English or Chinese.

Data extraction and synthesis: Two reviewers independently screened the searched items and extracted data. Four reviewers independently scored documents using AGREE II. Recommendations from all documents were tabulated and visualized in a colored grid.

Results: Twenty-two guidance documents (14 clinical practice guidelines and 8 consensus statements) were included. Included documents performed well in the domains of scope and purpose [median 76.4%, interquartile range (IQR) 69.4 to 79.2%] and clarity of presentation (median 72.2%, IQR 61.1% to 80.6%), but were unsatisfactory in applicability (median 21.9%, IQR 13.5% to 27.1%) and editorial

For peer review only

independence (median 47.9%, IQR 6.3% to 73.2%). The majority of obstetric anesthesia guidelines or consensus centered on different topics. Less than thirty percent of them specifically addressed the management of obstetric anesthesia perioperatively. Recommendations were concordant on the perioperative preparation, and on some indications for the choice of anesthesia method. Substantially different recommendations were provided for some items, especially for preoperative blood type and screen, and for the types and doses of neuraxial administration.

Conclusions: The methodological quality in guidance documents for obstetric anesthesia necessitates enhancement. Despite numerous trials in this area, evidence gaps persist for specific clinical queries in this filed. One potential approach to mitigate these challenges involves the endorsement of standardized guidance development methods and the synthesis of robust clinical evidence, aimed at diminishing difference in recommendations.

For peer review only

Strengths and limitations of this study

1. This systematic review is the inaugural effort to consolidate best practice recommendations for obstetric anesthesia.

2. The review was restricted to documents published in English and Chinese.

3. The potential for unconscious bias arose from the subjective assessment of the documents included.

4. Caution is warranted in interpreting the AGREE II results, as the AGREE framework assigns equal weighting to all six domains, irrespective of their individual significance.

For peer review only

Introduction

Obstetric anesthesia refers to labor analgesia, cesarean delivery anesthesia, pain management during the procedure of labor and delivery, and addressing obstetric emergencies. Epidural analgesia is considered the gold standard of intrapartum analgesia[1], with approximately 10%-83% of women giving birth under neuraxial anesthesia[2]. Despite technological advancements, anesthesiologists still encounter significant challenges in safely administering anesthesia to pregnant patients. Aligning with the 2021 World Patient Safety Day theme 'Safe Maternal and Newborn Care', the primary goal remains safeguarding the health of both mother and fetus during anesthesia administration.

To enhance anesthetic care for obstetric patients, many clinical guidelines and consensus statements have been created by healthcare institutions and professionals. They cover aspects like peri-anesthetic assessment, and management for vaginal and cesarean deliveries. For example, the American Society of Anesthesiologists (ASA) released obstetric anesthesia guidelines in 1998, with updates in 2007 and 2016[3, 4]. In China, the consensus on obstetric anesthesia was initially published in 2007, with subsequent updated in 2017 and 2020[5, 6]. Additionally, China published two editions of guidelines for labor analgesia in 2016 [7] and 2020[8].

Despite the variety of documents, current guidelines and consensuses on obstetric anesthesia and analgesia provide varied recommendations, such as whether require a platelet count, whether to order or require a blood type and screen or cross-match [4]. Furthermore, guideline development may be influenced by the experience and

For peer review only

composition of the guideline development groups. Low-quality guidance documents would pose risk to individual patients and communities, and impede the application of guideline recommendations in clinical practice[9]. Some guidelines were not written clearly, which may lead to clinician misunderstanding or not identifying key areas clearly, contributing to misjudged prioritizing of clinical actions [10]. Hence, it is essential to summarize and compare existing obstetric anesthesia guidelines or consensus statements to ensure the safety of both maternal and neonates during obstetric anesthesia. In this study, we aimed to evaluate the scope, content, and consistency of current guidance documents on obstetric anesthesia and analgesia. The quality of included guidelines and consensus statements were evaluated using the Appraisal of Guidelines for Research and Evaluation (AGREE) II instrument and presented as scores.

Methods

This study was developed based on the Preferred Reporting Items for Systematic Reviews and Meta Analyses for Protocols (PRISMA-P)[11]. The protocol of this study was registered prospectively at the International Platform of Registered Systematic Review and Meta-analysis Protocol.

Literature search and selection criteria

We conducted a systematically search of PubMed and EMBASE from inception to 8 June, 2023, employing a comprehensive search strategy (see online **Supplementary Table 1 and 2**) to identify guidelines and consensus recommendations

For peer review only

for anesthetic management of pregnant during labor, nonoperative delivery, operative delivery, and selected aspects of perioperative monitoring, postpartum care, and analgesia. We also searched three academic databases for Chinese publications (the Chinese Biomedical Literature Database, Wanfang Data, and Chinese Medical Association Database; see online **Supplementary Table 3**) and six guideline databases from inception to 7 June, 2023, using search strategies tailored to different databases (online **Supplementary Table 4**). To ensure thoroughness, we conducted additional searched on Google and Google Scholar on 1 August, 2023. Additionally, we scanned the reference lists of the most relevant clinical practice guidelines and review articles to identify any additional relevant guidelines. We included the latest versions of all international and national/regional clinical practice guidelines and consensus statements for the obstetric anesthetic management published in English or Chinese. We defined clinical practice guidelines and consensus statements as documents providing recommendations for patient care, which are derived from a systematic review of existing evidence or from collective opinions of an expert panel[12]. The exclusion criteria were: (1) original investigation, study protocols, comments on existing guidelines or consensus, and conference abstracts or posters; (2) draft documents that are under development or not finalized; (3) previous documents replaced by updated versions from the same organization.

Two reviewers (L.H and D.X.C) independently screened all searched documents. Full texts for potentially relevant guidelines or consensus statements were retrieved and examined for eligibility. Reasons for exclusion were provided for documents excluded

For peer review only - http://bmjopen.bmj.com/site/about/guidelines.xhtml

during the full-text review (online **Supplementary Table 5**). Disagreements were resolved through discussion with a third reviewer (J.S).

Data extraction

The following data was extracted from each included document, including publication year, funding body, and evidence base. Additionally, the data encompassed recommendations pertaining to monitoring and management, postpartum care, and analgesia. Data were extracted by one investigator and cross-checked by another investigator. In the event of any discrepancies, consensus was reached through discussion and resolution.

Appraisal of guidance documents

The included documents were evaluated by four independent reviewers (L.H, N.H, L.J., and D.X.C) using the Appraisal of Guidelines for Research and Evaluation (AGREE) II tool[13]. AGREE II is a validated instrument widely used to assess the quality of clinical practice guidelines and consensus statements[14]. The tool consists of 23 items categorized into six domains: scope and purpose, stakeholder involvement, rigor of development, clarity of presentation, applicability, and editorial independence. Each item is rated on a seven-point scale, ranging from 1 (Strongly Disagree) to 7 (Strongly Agree), indicating the level of agreement. The method for each domain appraised is present in Supplementary methods. In the current study, all appraisers completed an online training tutorial (Available: http://ww center-agree-ii-training-tools/ [Accessed

For peer review only

September 1, 2023]) prior to commencing the review process to ensure standardization. Detailed instruction from the AGREE II User's Manual[13] and objective scoring criteria (online **Supplementary Table 6**) for each item were used to promote scoring consistency.

Synthesis of recommendations

We meticulously extracted recommendations pertaining to critical clinical queries from a comprehensive set to guidance and consensus documents. Recommendations were extracted by two investigators (L.H and N. H) and checked by another (X.L.X and D.X.C). To visually represent these recommendations, we utilized a color-coded grid to illustrate any difference (online **Summary of Key Recommendations**).

Statistical analysis

All data was analyzed using the SPSS V.25.0 software. Median and interquartile range (IQR) were computed for the domain scores. The inter-rater agreement was computed using the intraclass correlation coefficient (ICC) with a two-way random effects model for the total score was computed. The level of agreement (ICC) was categorized according to widely recognized thresholds: poor (<0.40), fair (0.40–0.59), good (0.60–0.74) or excellent (0.75–1.00) [15]. Following the approach described in a previously published article, a domain is considered to have superior quality if its score exceeds 50%.

Results

Search results

We retrieved 6921 citations, with 6797 being excluded after screening titles and abstracts for not meeting eligibility criteria (**Figure 1**). We thoroughly assessed the remaining 124 citations and excluded 102 as they did not align with our defined criteria. Ultimately, we included fourteen guidelines [4, 16-28] and eight consensus statements [6-8, 29-33].

Characteristics of included guidelines and consensus statements

A summary of the characteristics of the eligible guidelines and consensus statements is presented in **Table 1**. Fourteen of guidelines [4, 16-28] and eight consensus statements [6-8, 29-33] with the latest versions were identified, published from 2014 to 2023, involving 15 national or multi-international organizations predominantly comprising anesthesiologists or obstetricians and gynecologists from the United States, United Kingdom, Europe, and China. Eight documents[17, 18, 22, 24, 26-28, 30] clearly stated being externally reviewed. Eight of these guidelines[4, 6, 16, 18, 19, 21, 24, 25] included procedures for updating in response to emerging evidence, while only six[4, 16, 19, 22, 29, 32] disclosed their funding body. The focus of these guidelines was primarily on the obstetric patients undergoing vaginal delivery or cesarean delivery (**Figure 2**), with eight documents[4, 6, 8, 18, 22-25] intended for use by anesthesiologists or other health care providers. Additionally, ten documents[4, 17-19, 21-23, 26, 27, 33] conducted systematic literature reviews as part of their development process, which eleven documents [4, 16-18, 21, 22, 24, 26, 27, 32, 33]

For peer review only

reported the level of evidence supporting recommendations and grading the strength of the guidance recommendations themselves.

Appraisal of guidelines and consensus statements

The standardized AGREE II domain scores for each guidance document are presented in **Figure 3** and provided in value as online **Supplementary Table 6**. Mean scores for each AGREE II item can be found in **Supplementary Table 9**. The ICC were above 0.90. The overall quality of guidance documents varied both between documents across domains and within documents across domains. The ASA guideline published in 2016 [4] had the highest domain scores, with five domains scoring in the upper quartile. This was followed by the guidelines published by the Enhanced Recovery After Surgery Society Recommendations (ERAS) (part2)[27], American College of Obstetricians and Gynecologists (ACOG)[16], and Royal College of Anesthetists (RcoA)[24], all with four domains scoring above the upper quartile. In total, 81.8% (18 of 22) of the included documents were excellent quality. Guidance documents received the highest scores for the scope and purpose domain (median 76.4%, IQR 69.4%–79.2%) and the clarity of presentation domain (median 72.2%, IQR 61.1 %–80.6%). However, they received the lowest scores for the applicability domain (median 21.9%, IQR 13.5%–27.1%) and the editorial independence domain (median 47.9%, IQR 6.3%–73.2%). No tendency of improvement in the quality score over time was observed.

For peer review only

Synthesis of recommendations

Approaches to the peri-anesthetic evaluation and preparation

Sixteen documents[4, 6-8, 16-18, 21, 22, 24-26, 28, 29, 31, 32]were included in the peri-anesthetic evaluation and preparation. Key recommendations are presented in **Supplementary Table 10**. Ten documents[4, 6-8, 16, 24, 25, 28, 29, 31] provided recommendations for history and physical examination in pregnant individuals, with an emphasis on maternal health, anesthetic history, and relevant obstetric history. Of these, six documents[4, 6-8, 16, 25] specially recommended examining the patient's back when a neuraxial anesthetic was planned. Eight documents[4, 6, 8, 16, 25, 26, 28, 29] highlighted the important of communication between obstetric providers, and anesthesiologists to recognize significant anesthetic or obstetric risk factors.

Six guidelines [4, 6, 7, 16, 21, 29] referenced in the literature emphasize the important of platelet count in predicting anesthesia-related complications. Three of these guidelines [4, 16, 21] recommended individualizing the platelet count based on a patient's medical history. Furthermore, five documents [4, 6, 7, 21, 29] discussed the necessity of blood type and screen or cross-match, with two of them[4, 21] suggesting that routine test is not essential for healthy and uncomplicated parturient (**Table 2**). Additionally, nine documents [4, 6-8, 16, 18, 22, 25, 29] indicated that fetal heart rate patterns should be monitored during the anesthetic process.

Eleven documents [4, 6, 17, 18, 21, 24-26, 29, 31, 32] addressed aspiration prevention during delivery. Regarding fasting, five documents [4, 6, 26, 31, 32] suggested that clear liquids up to 2 hours before anesthesia induction, and six documents [4, 6, 26, 29, 31, 32] recommended

For peer review only

a fasting period of 6-8 hours for solids. The use of antacids, H_2-receptor antagonists, and metoclopramide were also recommended for aspiration prophylaxis during cesarean delivery [4, 6, 17, 21, 24, 26, 31].

Five documents [7, 8, 16, 24, 25] discussed the use of heparin or low-molecular weight heparin during the peri- and post- anesthesia, and the ACOG [16] explicitly stated that prophylactic subcutaneous unfractionated heparin at a dosage of 5,000 units twice daily did not contraindicate neuraxial techniques, as long as catheter placement or removal more than 4-6 hours since last dose. In the case of intermediate-dose unfractionated heparin (7,500–10,000 units) more than 12 hours after the last dose or high-dose unfractionated heparin (total daily dose greater than 20,000 units) more than 24 hours after the last dose with activated partial thromboplastin time, it was deemed low risk to proceed with neuraxial anesthesia.

Approaches to the anesthetic care for labor and vaginal delivery

Eight documents [4, 7, 8, 16, 22, 24, 25, 31] addressed anesthetic care for labor and vaginal delivery (summarizing in **Supplementary Table 11**). Three documents [4, 7, 8] explicitly indicated that neuraxial analgesia could be administrated based on the level of discomfort the parturient, without limitation of cervical dilation. The ASA suggested providing labor neuraxial analgesia for parturient in early labor (i.e. cervical dilations of less than 5 cm dilation) [4]. These documents [4, 7, 8] also recommended to offer neuraxial analgesia on an individualized basis. The ASA also provided recommendations on the administration of neuraxial techniques for patients attempting vaginal birth after

For peer review only

previous cesarean delivery, as well as early placement of a neuraxial catheter [4].

There were three methods for neuraxial anesthetic: continuous infusion epidural analgesia (CIEA), combined spinal–epidural analgesia (CSEA), and single-injection spinal. Five documents [4, 7, 8, 16, 25] covered both CIEA and CSEA and recommended using local anesthetic, as well as adding opioids. Before a neuraxial procedure, the platelet count was required more than $70*10^9$/L to reduce the risk of hematoma [8, 16, 25]. The puncture location was recommended on L2-3 or L3-4 in China published consensus [7, 8]. Local anesthetic including bupivacaine, ropivacaine, and levobupivacaine were used with loading dose or maintenance doses. In China, published consensus [7, 8] recommended using 0.04% to 0.125% bupivacaine or 0.0625% to 0.15% ropivacaine to initiate analgesia and maintain with 0.05% to 0.125% bupivacaine or 0.0625% to 0.15% ropivacaine (**Table 2**). The Chinese Associations of Anesthesiology (CAA) consensus also provided recommendations for 0.04% to 0.125% levobupivacaine for loading dose and 0.05% to 0.125% levobupivacaine for maintenance [8]. These two documents suggested that opioids, including fentanyl or sufentanyl, could be injected with local anesthetic, with recommended loading dose of fentanyl was 0.5-2 $\mu g.ml^{-1}$ and 1-2 $\mu g.ml^{-1}$ for maintenance, and the loading dose of sufentanyl was 0.2-0.6 $\mu g.ml^{-1}$ with maintenance dose 0.3-0.6 $\mu g.ml^{-1}$ [7, 8]. The American Association of Nurse Anesthesiology (ANNA) guideline suggested using 0.0625% to 0.125% bupivacaine or 0.08% to 0.2% ropivacaine with or without opioids in CIEA [25]. In CSEA, opioids and local anesthetics could be delivered in spinal, with two recommended protocols [7, 8, 16, 25]: sufentanyl or ropivacaine used singly or in combination. The single dose of

For peer review only

sufentanyl was 2.5-7 μg, single dose of ropivacaine was 2.3-3 mg, and dose of combined sufentanyl and ropivacaine was 2.5 μg/2.5 mg, respectively [7, 8]. Another protocol was that fentanyl and bupivacaine was used singly or with combination. The single dose of fentanyl was 15-25 μg, single dose of bupivacaine was 2.0-2.5 mg, and dose of combined fentanyl and bupivacaine was 2.5 μg/2.0 mg, respectively [7, 8]. For CSEA and single-injection spinal, both documents recommended using a small-gauge, non-cutting, pencil point needle to access the subarachnoid space [4, 25]. Additionally, five documents [4, 7, 8, 25, 31] provided recommendations for using patient-controlled epidural analgesia for labor analgesia.

Approaches to the anesthetic care for cesarean delivery

Ten documents[4, 6, 16, 18, 24, 25, 27-29, 31] covered the anesthetic care including neuraxial anesthesia and general anesthesia for cesarean delivery (summarizing in **Supplementary Table 12**). The recommendations for neuraxial anesthesia including CIEA, single-injection spinal, and CSEA were consistent with those for labor and vaginal delivery. Eight of the documents [4, 6, 16, 18, 24, 25, 27, 28, 31] covered CIEA, CSEA, and single spinal injection, with four documents [6, 16, 25, 28] recommended the use of local anesthetic with or without opioids. A platelet count more than $70*10^9$/L was recommended to reduce the risk of hematoma[6, 16, 25]. Puncture locations were recommended as follows: L1-2 or L2-3 for CIEA, L3-4 for single-injection in spinal, and L3-4 in single space for CSEA, while in double space, the puncture location was recommended in T12-L2 or L3-5 [6, 25]. To test the effect of neuraxial anesthesia, the

For peer review only

recommended level of block was T6-4 [6]. Various local anesthetic, including lidocaine, bupivacaine, ropivacaine, and levobupivacaine were recommended for use in CIEA. In CAA 2020 [6], lidocaine was recommended with 1.5%-2% concentration, bupivacaine with 0.5%, ropivacaine with 0.5%-0.75% (**Table 2**). And 3% chloroprocaine was also recommended. In contrast, ANNA [25] recommended lower concentrations, with 0.0625-0.125% for bupivacaine and 0.08-0.2% for ropivacaine. For single injection spinal procedures, the recommended doses were 5-15 mg bupivacaine or 10-20 mg ropivacaine[6, 28]. To prevent local anesthetic poisoning, it was recommended that anesthesiologist withdraw before injection and administer a test dose [6, 16]. It was also suggested to add epinephrine to local anesthetic solutions to delay absorption and increase duration of blockade through vasoconstriction of the blood vessels in the area [6, 16]. Furthermore, the use of a small-gauge, non-cutting, pencil point needle was recommended [4, 6, 25].

Additionally, eight documents[4, 6, 16, 21, 24, 25, 29, 31] provided recommendations on general anesthesia for cesarean delivery. According to the ASA[4], general anesthesia may be the most appropriate choice in cases of profound fetal bradycardia, ruptured uterus, severe hemorrhage, severe placental abruption, umbilical cord prolapse, and preterm footling breech. Other documents[16, 25] recommended the use of general anesthesia if neuraxial anesthesia placement was unsuccessful. Induction-delivery was recommended within 10 minutes, with specific intravenous anesthetics. CAA[6] detailly recommended intravenous anesthetics including thiopental sodium (4-5 $mg.kg^{-1}$), propofol (1.5-2.5 $mg.kg^{-1}$), etomidate (0.2-0.3 $mg.kg^{-1}$), and ketamine (0.5-1 $mg.kg^{-1}$)

For peer review only

as induction agents. Fentanyl (2-5 μg.kg^{-1}), sufentanyl (0.2-0.5 μg.kg^{-1}), and remifentanil (4 ng.kg^{-1} for target) were commonly applied in the general anesthesia. Succinylcholine (1-1.5 mg.kg^{-1}) or rocuronium (0.6-1.2 mg.kg^{-1}) was used as a muscle relaxant.

Approaches to the monitoring of obstetric and anesthetic or intra- or postoperative pain management

To mitigate post-cesarean delivery pain, nine documents[16, 17, 19, 21, 23, 25, 31-33] provided recommendations for the management of intra- or postoperative pain (summarizing in **Supplementary Table 13**). Six of these documents [16, 19, 21, 25, 31-33] recommended the administration of intrathecal morphine 50-150 μg or epidural 1-3 mg for analgesia. Two documents [19, 21] also suggested the use of epidural diamorphine (2-3mg or 2.5-5 mg) for analgesia. ANNA [25] recommended hydromorphone 75-150 μg in intrathecal administration as well. When morphine was utilized in neuraxial block, four documents [21, 25, 29, 33] suggested monitoring respiratory frequency and modality. The Society of Obstetric Anesthesia and Perinatology (SOAP) [33] explicitly stated that using morphine less than 0.15 mg or epidural morphine less than 3 mg in healthy parturient would pose low risk, and recommended monitoring respiratory rate and sedation measurement every 2 hours for 12 hours postoperatively. However, in high-risk circumstances such as cardiopulmonary or neurological comorbidity, obesity (body mass index ≥ 40 kg/m^2), obstructive sleep apnea, chronic opioid use or abuse, and hypertension, or who was administrated with magnesium, as well as high doses of

For peer review only

morphine (morphine greater than 0.15 mg or epidural morphine greater than 3 mg), more intensive monitoring (respiratory rate and sedation assessments were monitored for every 1 h for first 12 h, every 2 h for 12–24 h) and additional modalities such as pulse oximetry and capnography were suggested (**Table 2**). To minimize systemic opioids utilization, eight documents[16, 17, 19, 21, 25, 31-33] recommended the administration of non-steroidal anti-inflammatory drug for postoperative pain. Two guidelines[19, 25] suggested a single dose of intravenous dexamethasone for analgesia. Local anesthetic infiltration for the wound after cesarean delivery was recommended by six documents[16, 17, 19, 25, 31, 32], and continuous local anesthetic infusion with the wound was recommended by three documents[16, 19, 31]. Nerve blocks, including transversus abdominis plane block and erector spinae plane block, for improving post-cesarean delivery pain was recommended by five documents [16, 17, 19, 31, 32]. Patient-controlled intravenous analgesia used for pain management after cesarean was recommended by four documents[16, 19, 21, 31]. Regarding obstetric neuraxial block, The Association of Anesthetists and the Obstetric Anesthetists' Association(AA and OAA) [20] stated that straight-leg raising was used for neurological monitoring, and if a maternal was unable to straight-leg raise at 4 h from the last dose of epidural/spinal local anesthetic, neurological injury required be investigated.

Approaches to the adverse events during obstetric anesthesia

Twelve documents[4, 6, 8, 16-18, 21, 25, 27, 30-32] provided recommendations for preventing the hypotension during obstetric anesthesia (summarizing in **Supplementary Table**

For peer review only

14). Eleven documents [4, 6, 8, 16-18, 21, 25, 27, 30, 31] advised on using 500-1000 ml volume expansion with crystals or colloids, as well as seven documents [4, 6, 8, 21, 25, 30, 31] recommended utilizing left uterine displacement once the maternal was positioned supine for surgery to reduce inferior vena cava compression. Vasopressor management during neuraxial anesthesia was recommended by ten documents [4, 6, 8, 16, 17, 21, 25, 30-32], with vasoactive drugs such as phenylephrine, metaraminol, noradrenalin and ephedrine. Phenylephrine with 20-50 $\mu g.min^{-1}$ or 0.25-0.5 $\mu g.\ kg^{-1}.\ min^{-1}$ was recommended by nine documents [4, 6, 8, 16, 17, 25, 30-32]. Ephedrine 2-15 mg intravenous was recommended by six documents [6, 8, 16, 17, 30, 31] (**Table 2**). The Association of Anesthetists of Great Britain and Ireland (AAGBI) [30] recommended that metaraminol with 0.5 $mg.ml^{-1}$could be used to mediate blood pressure. CAA [6] also recommended that noradrenalin 4-6 μg or 0.08 $\mu g.\ kg^{-1}.\ min^{-1}$ was useful for preventing hypotension. The target of blood pressure was to maintain systolic arterial pressure greater than 90% and avoid a decrease to < 80% baseline [21, 30]. For maternal with pre-eclampsia, two documents [6, 30] advised that vasopressor infusion should not be prophylactically used.

Six documents[8, 16, 23-25, 28] provided recommendations for inadequate neuraxial anesthesia, three guidelines [23, 25, 28] suggested assessing other causes of pain, evaluating the catheter site, and reassessing the sensory block level. For rescuing inadequate anesthesia, four documents[16, 23, 25, 28] provided recommendations including administrating a second neuraxial technique or extending the neuraxial technique, repeating boluses of fast-acting opioids (fentanyl 25-50 μg, alfentanil 250-500 μg) or ketamine (10 mg boluses), and if necessary, recommending general anesthesia.

For peer review only

Unintentional dural puncture would lead to post-dural puncture headache (PDPH). AANA[25] provided recommendations on using small, non-cutting spinal needle or placing intrathecal catheter for preventing PDPH. Treatment options for PDPH included epidural blood patch, adequate hydration, multimodal analgesia, caffeine, sumatriptan, gabapentin, and sphenopalatine ganglion block.

Approaches to the management of obstetric and anesthetic emergencies

Four documents [4, 6, 24, 25] provided recommendations for managing hemorrhagic emergencies, with three of them [4, 6, 25] consistently recommending the use of large bore intravenous catheters. The ASA [4] and ANNA [25] suggested the implementation of a massive transfusion protocol. Additionally, the ASA [4] recommended the availability of blood bank resource, fluid warmer, forced -air body warmer, and equipment for rapid infusion of intravenous fluids and blood products.

Seven documents[4, 6, 16, 18, 21, 24, 25] addressed airway emergency in obstetric anesthesia, with three of them [4, 16, 25] recommending the availability of personnel and equipment, such as laryngoscope with assorted blades and endotracheal tubes with stylets. In cases where tracheal intubation fails, the use of emergency procedures such as cricothyrotomy was advised. Access to oxygen source and suction source with tubing were also recommended[4, 21, 25]. The ASA provided specific recommendations for medications to support blood pressure, muscle relaxation, and hypnosis [4].

Three documents [4, 24, 25] covered cardiopulmonary resuscitation, with both the ASA [4] and ANNA [25] recommending the availability of basic and advanced life-support

For peer review only

equipment during cardiac arrest. It was also recommended to maintain uterine left displacement during cardiac arrest. If circulation was not restored within 4-5 min, two guidelines suggested performing a cesarean section [4, 24, 25].

Six documents[4, 16, 21, 24, 25, 28] addressed emergency cesarean delivery, with the National Institute for Health and Care Excellence (NICE)[21] providing specific recommendations for the time of unplanned and emergency cesarean births when there is a threat to the life of the woman or fetus, within 30 minutes. The use of neuraxial anesthesia and general anesthesia was recommended for emergency cesarean delivery [16, 21, 28].

Discussion

In this study, we systematically reviewed fourteen guidelines [4, 16-28] and eight consensus statements[6-8, 29-33]. The comparison of the content of the twenty-two documents showed that several documents were very comprehensive, covering many aspects of obstetric anesthesia [4, 6-8, 16, 18, 21, 24, 25, 27, 28, 31, 32],while other documents may focus on the specific contents[17, 19, 20, 22, 23, 26, 29, 30, 33]. We found generally average methodological quality and some varied recommendations from included documents covering the preoperative screening and management of obstetric anesthesia and analgesia. We summarized all key recommendations, and compared and visualized the difference among them, providing a concise but informative overview for clinicians and researchers.

For peer review only - http://bmjopen.bmj.com/site/about/guidelines.xhtml

Comparison with existing research

Our study systematically incorporated both guidelines and consensus statements in the field of obstetric anesthesia and analgesia, and we observed varied performance across different AGREE II domains for both types of documents. The assessment of the guidelines using the AGREE II tool revealed that the documents assessed in our study excelled in the domains of scope and purpose (domain 1) and clarity of presentation (domain 4); but performed poorly in the domain of applicability (domain 5) and editorial independence (domain 6). This distribution of AGREE II domain scores has been observed in previous guideline appraisal studies, with documents scoring higher in the scope and purpose domain and the clarity of presentation domain, and lower in the applicability domain and the editorial independence domain. This pattern was not only observed in guidance documents for endocrinology diseases[34, 35], but also in documents for other clinical specialties[36, 37]. Despite generally low and varied scores in the applicability domain, guidance documents for obstetric anesthesia and analgesia performed notably worse compared to documents for other conditions[34, 36], indicating that improving the applicability of guidance in this field may be more challenging. One major barrier to achieving good applicability of guidance documents is the time and cost required to perform economic evaluations and pilot studies. A stable and long-term task force for guideline development is needed to conduct these evaluations and studies. While forming such a task force may be difficult in some regions and countries, guidance documents should at least inform the audience about the need to consider these issues. Low scores in the editorial independence domain often resulted from a

For peer review only

lack of detailed information on the influence of the funding body and conflicts of interest, with 45.5% of included documents in the current study failing to provide this information. It is essential to disclose the potential influence of the pharmaceutical industry on the synthesis of clinical guidance and promote transparency in financial declarations.

Gaps, clinical implications and future research

Current guidance and consensus documents uniformly endorse thorough perioperative evaluations and tailored anesthesia methodologies for delivery, including cesarean sections. Notwithstanding these overarching agreements, variances in specific recommendations persist, likely stemming from the variable quality of the documents, gaps in current evidence, or reliance on antiquated studies. It is important to acknowledge that discrepancies in recommendations could stem from differing practices or organizational approaches internationally. For instance, some countries initiate blood transfusions following a type and screen with different procedures[38]. It is also imperative to recognize that numerous clinical considerations remain underrepresented in these guidance and consensus documents. Among these, the post-neuraxial anesthesia evaluation of nerve blocks warrants particular attention. This includes the necessity for continuous postoperative sensory monitoring, vigilant respiratory depression surveillance, and strategies for addressing inadequate analgesia.

In obstetric practice, neuraxial anesthesia is a cornerstone of pain management. However, it is not without risk. Atypical presentations of motor or sensory block, such

For peer review only

as unexpectedly dense or prolonged effects, may herald complications including unintended intrathecal placement or, in rare instances, neurological pathologies. Our review reveals that among the documents evaluated, only the AA and OAA[20] provided recommendations for neurological monitoring, specifically endorsing the straight-leg raise test. There is a conspicuous need for enhanced vigilance in post-procedure monitoring, particularly in the immediate post-partum period. Current clinical practice often overlooks the importance of neuraxial monitoring; a survey conducted in the UK highlighted that only 56% of units had a formal protocol for postoperative neurological assessment, with a few units deferring evaluation until after the first post-delivery day [39]. This discrepancy indicates that despite adequate guideline recommendations, clinical care often fails to meet expected standards. Therefore, when well-supported guidelines are introduced, it is crucial to intensify efforts to enhance the clinical adoption process. Shortening the timeframe for unified practice and enhancing the implementation efficacy of these guidelines can be achieved through the development of clinical practice pathways and the organization of training sessions to disseminate guideline updates. Additionally, greater emphasis should be placed on promoting the implementation of these guidelines. As the adage goes, there is much more to be gained in improving global health by applying existing knowledge than by investing in the development of new drugs.

Our review critically examined respiratory depression monitoring in the context of neuraxial anesthesia, particularly when neuraxial opioids such as morphine, fentanyl, sufentanyl, and remifentanil are administered. The ASA guideline[4] recommended

continuous monitored of ventilation, oxygenation, and consciousness in patients receiving neuraxial opioids. They further suggested intensified monitoring for those with a heightened risk of respiratory depression. Although the widespread endorsement of opioids in neuraxial anesthesia, it seemed that the monitoring of respiratory depression wasn't sufficiently highlight by these guidance documents.

Neuraxial anesthesia, while widely employed, does not always successful. The definition of 'inadequate' varies within the literature, contributing to this broad prevalence range. The prevalence of inadequate neuraxial anesthesia ranged from 10.2% - 30.3%[40]. Although there were several guidelines[8, 16, 23-25, 28] provided some recommendations on the management of inadequate neuraxial anesthesia. Patel *et al.* [41] revealed that only 23% of clinicians have received formal training in management of inadequate neuraxial anesthesia. Notably, the literature indices a significant gap in both the identification and management of inadequate neuraxial analgesia. Future initiatives must prioritize the development and dissemination of standardized education and training protocols to effectively recognized and manage inadequate neuraxial anesthesia.

Emergency cesarean section, often performed in cases with life-threatening conditions for the mother and fetus. Decision-to-delivery intervals was recommended within 30 minutes in category 1 cesarean birth when there was immediate threat to the life of the woman or fetus, and within 75 minutes in category 2 cesarean birth when there was maternal or fetal compromise which is not immediately life-threatening [21]. Nevertheless, studies found that decision-to-delivery intervals were frequently unmet

For peer review only

[42, 43]. There is significant heterogeneity in how decision-to-delivery intervals are defined and measured [9]. A pressing concern for anesthesiologists is the selection of anesthesia type- neuraxial anesthesia versus general anesthesia-in these emergent scenarios, for which the guidelines lack specific, universally applicable recommendations.

Limitations

Firstly, our inclusion of only English and Chinese documents may have overlooked key guidance from non-English/Chinese speaking regions, though we sought English versions where possible. Secondly, we focused on the anesthetic management for pregnant, some specialty guidelines which was also involved in pregnant (i.e. guidelines for cardiac arrest for maternal[44]) were not included. Thirdly, unconscious bias might have affected our subjective AGREE II ratings, warranting caution in their clinical application. The subjective nature of AGREE II scoring also challenges the reproducibility and comparison of different reviews. Lastly, certain topics in obstetric anesthesia, like epidural-related maternal fever, postpartum depression, and intra-partum hypothermia, were not covered.

Conclusions

The current obstetric anesthesia and analgesia guidelines require significant improvements in methodological quality. They showed inconsistencies in recommendations, highlighting the need for standardized guideline development and

For peer review only

high-quality evidence synthesis. Crucially, areas like neuraxial analgesia management and monitoring of postoperative neurological functions and intrapartum fever are underrepresented, indicating a gap in evidence-based guidelines and necessitating comprehensive research.

Data availability statement

All data relevant to the study are included in the article or uploaded as supplementary information.

Acknowledgement

All analyses were based on previously published guidelines or consensus, thus no ethical approval is required.

Funding information

This study was funded, in part, by a grant from the Science and Technology Department of Sichuan Province (No. 2023NSFSC1570 and 24NSFSC6388).

Conflict of interest statement

The authors report no conflict of interest.

Author contributions

Dong Xu Chen designed the study. Lu Huang and Dong Xu Chen performed the literature search and screening and extracted the data. Lu Huang, Na Hu, Ling Jian, Xing Long Xiong, Jing Shi and Dong Xu Chen conducted the data analyses. Lu Huang and Dong Xu Chen created the figures and tables and drafted the manuscript. All authors participated in the interpretation of results, and critically revised the manuscript.

For peer review only

All authors approved the final version of the manuscript for publication.

Reference

1. Anim-Somuah M, Smyth RM, Cyna AM, Cuthbert A. Epidural versus non-epidural or no analgesia for pain management in labour. *Cochrane Database of Syst Rev* 2018;5:Cd000331. http://doi.org/10.1002/14651858.CD000331.pub4.

2. Seijmonsbergen-Schermers AE, van den Akker T, Rydahl E, *et al*. Variations in use of childbirth interventions in 13 high-income countries: A multinational cross-sectional study. *PLOS Med* 2020;17:e1003103. http://doi.org/10.1371/journal.pmed.1003103.

3. Practice guidelines for obstetric anesthesia: an updated report by the American Society of Anesthesiologists Task Force on Obstetric Anesthesia. *Anesthesiology* 2007;106:843-63. http://doi.org/10.1097/01.anes.0000264744.63275.10.

4. Practice Guidelines for Obstetric Anesthesia: An Updated Report by the American Society of Anesthesiologists Task Force on Obstetric Anesthesia and the Society for Obstetric Anesthesia and Perinatology. *Anesthesiology* 2016;124:270-300. http://doi.org/10.1097/ALN.0000000000000935.

5. Chinese Society of Anesthesiology. Chapter 8, expert consensus on the obstetric anesthesia. Expert consensus and guidelines for Anesthetic Management in China (original document in Chinese). Beijing: People′s Medical Publishing House; 2017.

6. Chinese Society of Anesthesiology. Chapter 8, expert consensus on the obstetric anesthesia. Expert consensus and guidelines for Anesthetic Management in China (original document in Chinese). Beijing: People′s Medical Publishing House; 2020.

7. Group of Obstetric Anesthesia of Chinese Society of Anesthesiology. Expert consensus on labour analgesia (original document in Chinese). *J Clin Anesthesiol* 2016; 32: 816-8.

8. Chinese Society of Anesthesiology. Chapter 9, expert consensus on the labour analgesia. Expert consensus and guidelines for Anesthetic Management in China (original document in Chinese). Beijing: People′s Medical Publishing House; 2020.

9. Winther LP, Mitchell AU, MØLler AM. Inconsistencies in clinical guidelines for obstetric anaesthesia for cesarean section. *Acta Anaesthesiol Scand* 2012;57:141-49.

For peer review only

http://doi.org/10.1111/aas.12004.

10. Woolf SH, Fau. GR, Hutchinson A, A. H, M. E, Grimshaw J. Clinical guidelines: potential benefits, limitations, and harms of clinical guidelines. *BMJ* 1999;318:527-30. https://doi.org/10.1136/bmj.318.7182.527.

11. Shamseer L, Moher D, Clarke M, *et al*. Preferred reporting items for systematic review and meta-analysis protocols (PRISMA-P) 2015: elaboration and explanation. *BMJ* 2015;350:g7647. https://doi.org/10.1136/bmj.g7647.

12. Robin Graham, Michelle Mancher, Dianne Miller Wolman, Sheldon Greenfield, Earl Steinberg, editors. Clinical Practice Guidelines We Can Trust. Washington (DC): National Academies Press (US) 2011.

13. Brouwers MC, Kho ME, Browman GP, *et al*. AGREE II: advancing guideline development, reporting, and evaluation in health care. *Prev Med* 2010;51:421-4. http://doi.org/10.1016/j.ypmed.2010.08.005.

14. Deng Y, Luo L, Hu Y, Fang K, Liu J. Clinical practice guidelines for the management of neuropathic pain: a systematic review. *BMC anesthesiol* 2016;16:12. http://doi.org/10.1186/s12871-015-0150-5.

15. Brouwers MC, Kho ME, Browman GP, *et al*. Development of the AGREE II, part 1: performance, usefulness and areas for improvement. *CMAJ* 2010;182:1045-52. http://doi.org/10.1503/cmaj.091714.

16. American College of Obstetricians and Gynecologists' Committee on Practice Bulletins—Obstetrics. ACOG Practice Bulletin No. 209: Obstetric Analgesia and Anesthesia. *Obstet Gynecol* 2019;133:e208-e25. http://doi.org/10.1097/aog.0000000000003132.

17. Macones GA, Caughey AB, Wood SL, *et al*. Guidelines for postoperative care in cesarean delivery: Enhanced Recovery After Surgery (ERAS) Society recommendations (part 3). Am J Obstet Gynecol 2019;221:247.e1-e9. http://doi.org/10.1016/j.ajog.2019.04.012.

18. Maxwell C, Gaudet L, Cassir G, *et al*. Guideline No. 392-Pregnancy and Maternal Obesity Part 2: Team Planning for Delivery and Postpartum Care. *J Obstet Gynaecol*

For peer review only

Can 2019;41:1660-75. http://doi.org/10.1016/j.jogc.2019.03.027.

19. Roofthooft E, Joshi GP, Rawal N, *et al*. PROSPECT guideline for elective cesarean section: updated systematic review and procedure-specific postoperative pain management recommendations. *Anaesthesia* 2020;76:665-80. http://doi.org/10.1111/anae.15339.

20. Yentis SM, Lucas DN, Brigante L, *et al*. Safety guideline: neurological monitoring associated with obstetric neuraxial block 2020. *Anaesthesia* 2020;75:913-19. http://doi.org/10.1111/anae.14993.

21. National Institute for Clinical Excellence. Cesarean Section. Published: 31 March 2021, Available at: https://wwwniceorguk/guidance/NG192 2021.

22. Mei-Dan E, Jain V, Melamed N, *et al*. Guideline No. 428: Management of Dichorionic Twin Pregnancies. *J Obstet Gynaecol Can* 2022;44:819-34.e1. http://doi.org/10.1016/j.jogc.2022.05.002.

23. Plaat F, Stanford SER, Lucas DN, *et al*. Prevention and management of intra-operative pain during cesarean section under neuraxial anaesthesia: a technical and interpersonal approach. *Anaesthesia* 2022;77:588-97. http://doi.org/10.1111/anae.15717.

24. Royal College of Anaesthetists. Guidelines for the Provision of Anaesthetic Services: Chapter 9 Guidelines for the Provision of Anaesthesia Services for an Obstetric Population 2023. Available at: https://www.rcoa.ac.uk/gpas/chapter-9.

25. American Association of Nurse Anesthesiology. Analgesia and Anesthesia for the Obstetric Patient. Available at: https://issuu.com/aanapublishing/docs/analgesia_and_anesthesia_for_the_obstetric_patient?fr=sN2ZlNTU2NDAxMjU.

26. Wilson RD, Caughey AB, Wood SL, *et al*. Guidelines for Antenatal and Preoperative care in Cesarean Delivery: Enhanced Recovery After Surgery Society Recommendations (Part 1). *Am J Obstet Gynecol* 2018;219:523.e1-e15. http://doi.org/10.1016/j.ajog.2018.09.015.

27. Caughey AB, Wood SL, Macones GA, *et al*. Guidelines for intraoperative care in

For peer review only

cesarean delivery: Enhanced Recovery After Surgery Society Recommendations (Part 2). *Am J Obstet Gynecol* 2018;219:533-44. http://doi.org/10.1016/j.ajog.2018.08.006.

28. Keita H, Deruelle P, Bouvet L, *et al*. Raising awareness to prevent, recognise and manage acute pain during caesarean delivery: The French Practice Bulletin. *Anaesth Crit Care Pain Med* 2021;40:100934. https://doi.org/10.1016/j.accpm.2021.100934.

29. Obstetrics Subgroup, Society of Obstetrics and Gynecology, Chinese Medical Association.. Expert consensus on cesarean delivery. *Zhonghua fu chan ke za zhi* 2014;49:721-24.

30. Kinsella SM, Carvalho B, Dyer RA, *et al*. International consensus statement on the management of hypotension with vasopressors during cesarean section under spinal anaesthesia. *Anaesthesia* 2017;73:71-92. http://doi.org/10.1111/anae.14080.

31. China Healthy Birth Science Association. Expert Consensus on Enhanced Recovery after Delivery. *Progress in Obstetrics and Gynecology* 2020;29:561-67.

32. Bollag L, Lim G, Sultan P, *et al*. Society for Obstetric Anesthesia and Perinatology: Consensus Statement and Recommendations for Enhanced Recovery After Cesarean. *Anesth Analg* 2021;132:1362-77. http://doi.org/10.1213/ane.0000000000005257.

33. Bauchat JR, Weiniger CF, Sultan P, *et al*. Society for Obstetric Anesthesia and Perinatology Consensus Statement: Monitoring Recommendations for Prevention and Detection of Respiratory Depression Associated With Administration of Neuraxial Morphine for Cesarean Delivery Analgesia. *Anesth Analg* 2019;129:458-74. http://doi.org/10.1213/ane.0000000000004195.

34. Wu CM, Wu AM, Young BK, *et al*. An Appraisal of Clinical Practice Guidelines for Diabetic Retinopathy. *Am J Med* 2016;31:370-5. http://doi.org/10.1177/1062860615574863.

35. Fang Y, Yao L, Sun J, *et al*. Appraisal of clinical practice guidelines on the management of hypothyroidism in pregnancy using the Appraisal of Guidelines for Research and Evaluation II instrument. *Endocrine* 2018;60:4-14. http://doi.org/10.1007/s12020-018-1535-2.

36. Gavriilidis P, Roberts KJ, Askari A, *et al*. Evaluation of the current guidelines for

For peer review only

resection of hepatocellular carcinoma using the Appraisal of Guidelines for Research and Evaluation II instrument. *J Hepatol* 2017;67:991-98. http://doi.org/10.1016/j.jhep.2017.06.028.

37. Li Q, Li X, Wang J, *et al*. Diagnosis and treatment for hyperuricemia and gout: a systematic review of clinical practice guidelines and consensus statements. *BMJ open* 2019;9:e026677. http://doi.org/10.1136/bmjopen-2018-026677.

38. Pei Z, Szallasi A. Prevention of surgical delays by pre-admission type and screen in patients with scheduled surgical procedures: improved efficiency. *Blood transfus* 2015;13:310-2. http://doi.org/10.2450/2014.0172-14.

39. Roderick E, Hoyle J, Yentis SM. A national survey of neurological monitoring practice after obstetric regional anaesthesia in the UK. *Anaesthesia* 2017;72:755-59. http://doi.org/10.1111/anae.13800.

40. Patel R, Kua J, Sharawi N, *et al*. Inadequate neuraxial anaesthesia in patients undergoing elective cesarean section: a systematic review. *Anaesthesia* 2022;77:598-604. http://doi.org/10.1111/anae.15657.

41. Patel R, Russell R, Plaat F, *et al*. Inadequate neuraxial anaesthesia during cesarean delivery: a survey of practitioners. *Int J Obstet Anesth* 2023;56:103905. http://doi.org/10.1016/j.ijoa.2023.103905.

42. Ayeni OM, Aboyeji AP, Ijaiya MA, *et al*. Determinants of the decision-to-delivery interval and the effect on perinatal outcome after emergency cesarean delivery: a cross-sectional study. *Malawi Med J* 2021;33:28-36. http://doi.org/ 10.4314/mmj.v33i1.5.

43. Kitaw TM, Limenh SK, Chekole FA, *et al*. Decision to delivery interval and associated factors for emergency cesarean section: a cross-sectional study. *BMC Pregnancy and Childbirth* 2021;21:224. http://doi.org/10.1186/s12884-021-03706-8.

44. Lott C, Truhlář A, Alfonzo A, *et al*. European Resuscitation Council Guidelines 2021: Cardiac arrest in special circumstances. *Resuscitation* 2021;161:152-219. https://doi.org/10.1016/j.resuscitation.2021.02.011.

For peer review only

Table 1. Characteristics of included guidelines and consensus statements.

Document	Issuing organization	Country	Funding body	Target population	Target audience	Guideline development group	Guideline review	Guideline update	Evidence base	LOE	SOR
**Guidelines**											
**ASA_2016** [4]	American Society of Anesthesiologists	US	Institutional	Labor, nonoperative delivery, operative delivery, and selected aspects of postpartum care and analgesia (not apply to patients undergoing surgery during pregnancy, gynecological patients, or parturient with chronic medical disease)	Anesthesiologists	Multi	NG	1999-2006-2016	CS and SLR	+	+
**ERAS_2018 (Part 1, 2, 3)** [17,26,27]	Enhanced Recovery After Surgery	Multination	NG	Cesarean delivery patients	NG	ERAS	External review	NG	SLR	+	+
**ACOG_2019** [16]	American College of Obstetricians and Gynecologists	US	Institutional	NG	NG	ACOG	NG	2002-2007-2013-2017-2019	NG	+	+
**SOGC_2019** [18]	Society of Obstetricians and Gynecologists of Canada	Canada	NG	Women with obesity who are pregnant or planning pregnancies	Health care providers	SOGC	External review	5 years	SLR	+	+
**SOGC_2022** [22]	Society of Obstetricians and Gynecologists of	Canada	Institutional	Pregnant women with a dichorionic twin pregnancy	Health care providers	SOGC	External review	NG	SLR	+	+

	Canada										
**AA and OAA_2020** [20]	Association of Anesthetists and the Obstetric Anesthetists' Association	UK	None	Obstetric neuraxial block patients	NG	AA and OAA	NG	NG	NG	-	-
**PROSPECT_2020** [19]	Procedure-specific postoperative pain management working group	Europe	OAA and European Society of Regional Anesthesia and Pain Therapy	Cesarean delivery patients (not be applied to emergency or unplanned cesarean section or surgery performed under general anesthesia.)	NG	Multi	NG	2014-2020	SLR	-	-
**NICE_2021** [21]	National Institute for Health and Care Excellence	UK	NG	Cesarean delivery patients	NG	NICE	NG	2011-2014-2021	SLR	+	+
**OAA_2022** [23]	Obstetric Anesthetists' Association	UK	None	Cesarean delivery patients	Anesthetists	OAA	NG	NG	CS and SLR	-	-
**ANNA_2023** [25]	American Association of Nurse Anesthesiology	US	NG	Obstetric patients during labor and delivery and related procedures	CRNAs	AANA	NG	2008-2023	NG	-	-
**RCoA_2023** [24]	Royal College of Anesthetists	UK	NG	All pregnant women (antenatal anesthesia, labor, peripartum operative procedures, high dependency care on labor ward, obstetric procedures during pregnancy, e.g. cervical sutures,	Health care providers	RCoA	External review	Planned in 2024	NG	+	+

For peer review only

				fetal surgery.)							
**French_2021**[28]	French Club anesthe´sie-re´animation en obste´trique	France	NG	Cesarean delivery patients	Stakeholders involved in the care of women undergoing caesarean section	Multi	External review	NG	NG	-	-
**Consensus statements**											
**OG-OG-CMA_2014**[29]	Obstetrics Group, Obstetrics and Gynecology Branch of Chinese Medical Association	China	The Research Special Fund for Public Welfare Industry of Health of the Ministry of Health of China	Cesarean delivery patients	NG	OG-OG-CMA	NG	NG	NG	-	-
**OG-A-CMA_2016**[7]	Obstetrics Group, Anesthesiology Branch of Chinese Medical Association	China	NG	Labor analgesia	NG	OG-A-CMA	NG	NG	NG	-	-
**AAGBI_2017**[30]	Association of Anesthetists of Great Britain and Ireland	Europe (Ireland)	None	Cesarean delivery patients	NG	AAGBI	External review	NG	NG	-	-
**SOAP_2019**[33]	Society for Obstetric Anesthesia and Perinatology	US	None	Cesarean delivery patients	NG	SOAP	NG	NG	SLR	+	+
**CHBSA_2020**[31]	China Healthy Birth	China	NG	All pregnant women	NG	CHBSA	NG	NG	NG	-	-

For peer review only

	Science Association										
**CAA_2020** [6]	Chinese Association of Anesthesiology	China	NG	All pregnant women	Anesthesiologists	CAA	NG	2008-2017-2020	NG	-	-
**CAA_2020** [8]	Chinese Association of Anesthesiology	China	NG	Labor and vaginal delivery	Anesthesiologists	CAA	NG	NG	NG	-	-
**SOAP_2020** [32]	Society for Obstetric Anesthesia and Perinatology	US	National Institutes of Health	Cesarean delivery patients	NG	SOAP	NG	NG	NG	+	+

ASA, American Society of Anesthesiologists; ERAS, Enhanced Recovery After Surgery; ACOG, American College of Obstetricians and Gynecologists; SOGC, Society of Obstetricians and Gynecologists of Canada; AA and OAA, Association of Anesthetists and the Obstetric Anesthetists' Association; PROSPECT, Procedure-specific postoperative pain management working group; NICE, National Institute for Health and Care Excellence; OAA, Obstetric Anesthetists' Association; ANNA, American Association of Nurse Anesthesiology; RcoA, Royal College of Anesthetists; OG-OG-CMA, Obstetrics Group, Obstetrics and Gynecology Branch of Chinese Medical Association; OG-A-CMA, Obstetrics Group, Anesthesiology Branch of Chinese Medical Association; AAGBI, Association of Anesthetists of Great Britain and Ireland; SOAP, Society for Obstetric Anesthesia and Perinatology; CHBSA, China Healthy Birth Science Association; CAA, Chinese Association of Anesthesiology. LOE, level of evidence; SOR, strength of recommendation; SLR, systematic literature review. NG, not given.

For peer review only

Table2. Some different recommendations summarized.

		The most frequently recommended content	A partially consistent recommendation	Contrary recommendation
**Approaches to the peri-anesthetic evaluation and preparation**				
**Routine intrapartum platelet count**		Agree	-	Individualized
**Routine blood type and screen**		Agree	-	Individualized
**Routine blood cross-match**		Agree	-	Individualized
**Approaches to the anesthetic care for labor and vaginal delivery**				
**Bupivacaine**	Continuous infusion epidural analgesia	0.04% to 0.125%	0.0625% to 0.125%	-
	Single-injection spinal	No guideline	2.0-2.5mg	-
**Ropivacaine**	Continuous infusion epidural analgesia	0.0625% to 0.15%	0.08% to 0.2%	-
	Single-injection spinal	No guideline	2.3-3 mg	-
**Fentanyl**	Continuous infusion epidural analgesia	0.5 to 2 $\mu g.ml^{-1}$	1 to 2 $\mu g.ml^{-1}$	-
**Sufentanyl**	Continuous infusion epidural analgesia	0.2 to 0.6 $\mu g.ml^{-1}$	0.4 to 0.6 $\mu g.ml^{-1}$	-
**Approaches to the anesthetic care for cesarean delivery**				
**Bupivacaine**	Continuous infusion epidural analgesia	0.50%	0.0625% to 0.125%	
	Single-injection spinal	No guideline	5-15 mg	-
**Ropivacaine**	Continuous infusion epidural analgesia	0.5% to 0.75%	0.08% to 0.2%	
	Single-injection spinal	No guideline	10-20 mg	-
**Approaches to the monitoring of obstetric and anesthetic or intra- or postoperative pain management**				
**High risk maternal respiratory monitoring**		No guideline	q1h clinical assessment for first 12 h or half hour for 2 hours	-
**Neuraxial adjuvant drugs in spinal anesthesia**	Morphine	50 to 150 µg	150 to 500 µg	-
**Neuraxial adjuvant drugs in epidural or**	Diamorphine	2 to 3 mg	2.5 to 5 mg	-

For peer review only

**combined spinal-epidural technique.**				
**Approaches to the adverse events during obstetric anesthesia**				
**Vasoactive drugs used for preventing hypotension**	Phenylephrine	20-50 μg or 0.5 μg. kg $^{-1}$. min $^{-1}$	20~50 μg or 0.25. kg $^{-1}$. Min $^{-1}$	-
	Metaraminol	No guideline	1-2 mg or 4 μg. kg $^{-1}$. min^{-1} or 0.5 mg.ml^{-1}	-
	Noradrenalin	4-6 μg or 0.08 μg. kg $^{-1}$. min^{-1}	-	-
	Ephedrine	2 to 15 mg iv	2 to 4 mg	

Note: It is paramount to emphasize that the above contradictory recommendations serve as a concise overview, without claiming comprehensiveness.

For peer review only

Figures Legend

Figure 1. Flow diagram for literature search.

CBM, Chinese Biomedical Literature Database; CMAD, Chinese Medical Association Database; NGC, National Guideline Clearinghouse; GIN, Guidelines International Network; NICE, National Institute for Health and Care Excellence; SIGN, Scottish Intercollegiate Guidelines Network; TRIP, Turning Research into Practice Database. AGREE, appraisal of guidelines for research and evaluation.

Figure 2. A summary of the main focus of the included document.

ASA, American Society of Anesthesiologists; ERAS, Enhanced Recovery After Surgery; ACOG, American College of Obstetricians and Gynecologists; SOGC, Society of Obstetricians and Gynecologists of Canada; AA and OAA, Association of Anesthetists and the Obstetric Anesthetists' Association; PROSPECT, Procedure-specific postoperative pain management working group; NICE, National Institute for Health and Care Excellence; OAA, Obstetric Anesthetists' Association; ANNA, American Association of Nurse Anesthesiology; RcoA, Royal College of Anesthetists; OG-OG-CMA, Obstetrics Group, Obstetrics and Gynecology Branch of Chinese Medical Association; OG-A-CMA, Obstetrics Group, Anesthesiology Branch of Chinese Medical Association; AAGBI, Association of Anesthetists of Great Britain and Ireland; SOAP, Society for Obstetric Anesthesia and Perinatology; CHBSA, China Healthy Birth Science Association; CAA, Chinese Association of Anesthesiology.

Figure 3. Standardized domain scores for each document.

ASA, American Society of Anesthesiologists; ERAS, Enhanced Recovery After Surgery; ACOG, American College of Obstetricians and

For peer review only

Gynecologists; SOGC, Society of Obstetricians and Gynecologists of Canada; AA and OAA, Association of Anesthetists and the Obstetric Anesthetists' Association; PROSPECT, Procedure-specific postoperative pain management working group; NICE, National Institute for Health and Care Excellence; OAA, Obstetric Anesthetists' Association; ANNA, American Association of Nurse Anesthesiology; RcoA, Royal College of Anesthetists; OG-OG-CMA, Obstetrics Group, Obstetrics and Gynecology Branch of Chinese Medical Association; OG-A-CMA, Obstetrics Group, Anesthesiology Branch of Chinese Medical Association; AAGBI, Association of Anesthetists of Great Britain and Ireland; SOAP, Society for Obstetric Anesthesia and Perinatology; CHBSA, China Healthy Birth Science Association; CAA, Chinese Association of Anesthesiology.

For peer review only

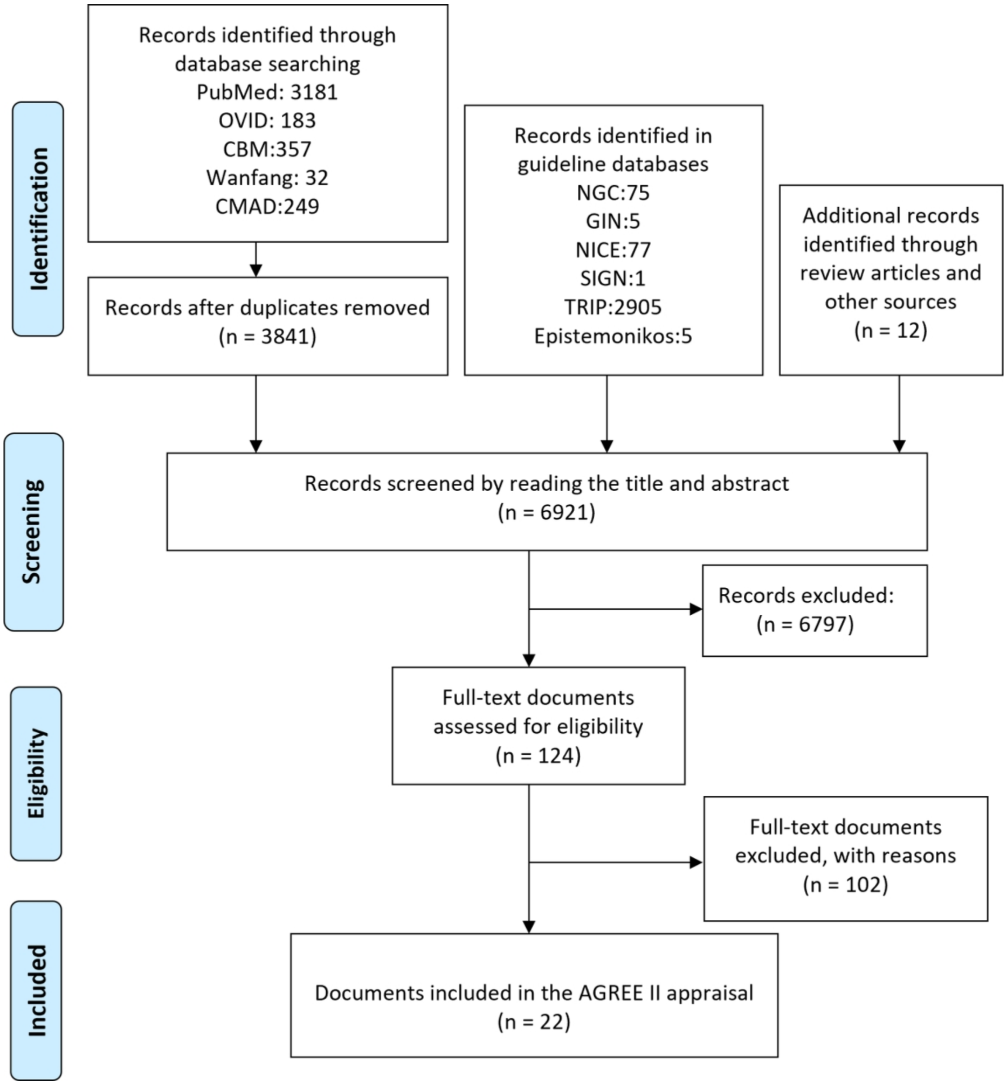

Flow diagram for literature search.

162x175mm (300 x 300 DPI)

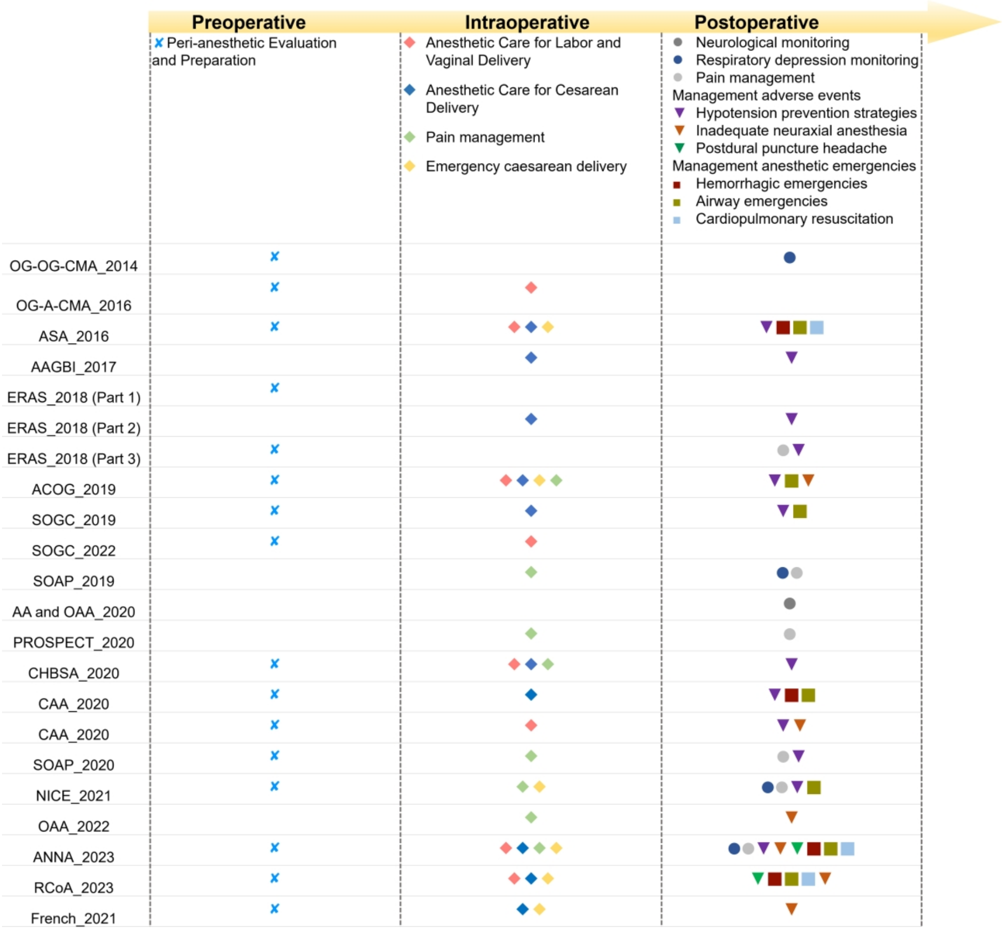

A summary of the main focus of the included document.

336x311mm (300 x 300 DPI)

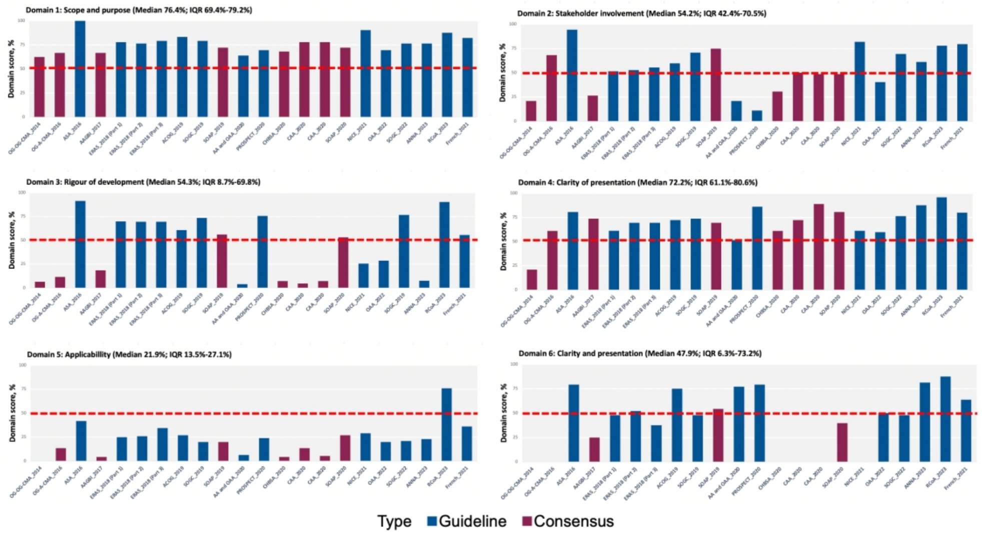

Standardized domain scores for each document

440x250mm (166 x 166 DPI)

Supplementary Materials

Contents

Supplementary Table 1. Search strategy in PubMed.
Supplementary Table 2. Search strategy in EMBASE using the OVID interface.
Supplementary Table 3. Searches in Chinese databases.
Supplementary Table 4. Searches in guideline databases.
Supplementary Table 5. Excluded studies and reasons for exclusion.
Supplementary methods. How to calculate the scores.
Supplementary Table 6. Scores for each individual AGREE II domain items by each reviewer.
Supplementary Table 7. The intraclass correlation coefficient between reviewers for each individual AGREE II domain item.
Supplementary Table 8. Domain score for each included guidance document.
Supplementary Table 9. Mean scores across reviewers for the individual AGREE II domain items.
Supplementary Table 10. Summary of recommendations for the peri-anesthetic evaluation and preparation by included guidance document.
Supplementary Table 11. Summary of recommendations for the anesthetic care for labor and vaginal delivery by included guidance document.
Supplementary Table 12. Summary of recommendations for the anesthetic care for cesarean delivery by included guidance document.
Supplementary Table 13. Summary of recommendations for the monitoring and management of obstetric and anesthetic or intra- or postoperative pain management by included guidance document.
Supplementary Table 14. Summary of recommendations for the management of adverse events by included guidance document.
Supplementary Table 15. Summary of recommendations for the management of obstetric and anesthetic emergencies by included guidance document.

For peer review only

Supplementary Table 1. Search strategy in PubMed.

1	((((((cesarean[Title/Abstract]) OR (cesarean delivery[Title/Abstract])) OR (cesarean section[Title/Abstract])) OR (obstetric[Title/Abstract])) OR (obstetrical delivery[Title/Abstract])) OR (obstetric anesthesia[Title/Abstract])) OR (delivery[Title/Abstract])	633,790
2	((expert consensus[Title/Abstract]) OR (guideline[Title/Abstract])) OR (recommendation statement[Title/Abstract])	88,342
3	("2000/01/01"[Date - Publication]: "3000"[Date - Publication])	22,218,196
4	1 AND 2 AND 3	3,181

Date of literature search: June 6, 2023

For peer review only - http://bmjopen.bmj.com/site/about/guidelines.xhtml

Supplementary Table 2. Search strategy in EMBASE using the OVID interface.

1	cesarean.m_titl.	26,523
2	cesarean delivery.m_titl.	8,147
3	cesarean section.m_titl.	12,708
4	obstetric.m_titl.	23,566
5	obstetrical delivery.m_titl.	16
6	obstetric anesthesia.m_titl.	961
7	delivery.m_titl.	185,141
8	1 or 2 or 3 or 4 or 5 or 6 or 7	224,835
9	guideline.m_titl.	29,968
10	expert consensus.m_titl.	3,906
11	recommendation statement.m_titl.	538
26	9 or 10 or 11	34,373
21	8 and 12	183

Date of literature search: Embase <1974 to 2023 June 06>

For peer review only - http://bmjopen.bmj.com/site/about/guidelines.xhtml

Supplementary Table 3. Searches in Chinese databases.

**Chinese Biomedical Literature Database**			
[Original search term in Chinese] (cesarean OR obstetric OR obstetric anesthesia) AND (guideline OR consensus OR statement OR recommendation)	Results found	Full text screened	Included documents
	357	2	1
Date of literature search: June 8, 2023			
**Wanfang Data**			
[Original search term in Chinese] (cesarean OR obstetric OR obstetric anesthesia) AND (guideline OR consensus OR statement OR recommendation)	Results found	Full text screened	Included documents
	32	2	2
Date of literature search: June 8, 2023			
**Chinese Medical Association Database**			
[Original search term in Chinese] (cesarean OR obstetric OR obstetric anesthesia) AND (guideline OR consensus OR statement OR recommendation)	Results found	Full text screened	Included documents
	249	4	2
Date of literature search: June 8, 2023			

For peer review only

Supplementary Table 4. Searches in guideline databases.

Databases	Date of search	Search strategy	Results found	Full text screened	Included documents	URL
National Guideline Clearinghouse	2023/6/7	cesarean OR cesarean section OR cesarean delivery OR obstetric OR obstetrical delivery OR obstetric anesthesia OR delivery	75	1	0	www.guideline.gov
Guidelines International Network	2023/6/7	cesarean OR cesarean section OR cesarean delivery OR obstetric OR obstetrical delivery OR obstetric anesthesia OR delivery<br>Search mode: Guidelines	5	1	1	www.g-i-n.net
National Institute for Health and Care Excellence	2023/6/7	cesarean OR obstetric<br>filter type: guidance	77	3	1	www.nice.org.uk
Scottish Intercollegiate Guidelines Network	2023/6/7	Not applicable	1	0	0	www.sign.ac.uk/our-guidelines.html
Turning Research Into Practice Database	2023/6/7	cesarean OR cesarean section OR obstetric OR obstetric anesthesia<br>filter: Guidelines	2,905	11	6	www.tripdatabase.com
Epistemonikos database	2023/6/7	cesarean OR obstetric OR obstetric anesthesia<br>filter: Broad syntheses OR Structured summaries	5	1	1	www.epistemonikos.org

For peer review only - http://bmjopen.bmj.com/site/about/guidelines.xhtml

Supplementary Table 5. Excluded studies and reasons for exclusion.

Study	Reason for exclusion
Caughey AB, Sundaram V, Kaimal AJ, et al. Maternal and neonatal outcomes of elective induction of labor. Evidence Report/Technology Assessment No. 176. (Prepared by the Stanford University-UCSF Evidenced-based Practice Centre under contract No. 290-02-0017.) AHRQ Publication No. 09-E005. Rockville, MD.: Agency for Healthcare Research and Quality. March 2009.	Not providing specific recommendations for obstetric anesthesia
Association of Ontario Midwives. Vaginal birth after previous low-segment cesarean section. 2021; (Clinical Practice Guideline No. 14).	Not providing specific recommendations for obstetric anesthesia
Obstetric Care Consensus: Safe prevention of the primary cesarean delivery. March 2014	Not providing specific recommendations for obstetric anesthesia
Guidelines for neuraxial analgesia or anesthesia in obstetrics. American Society of Anesthesiologists, October 13, 2021.	Conference abstract
Optimal goals for anesthesia care in obstetrics. American Society of Anesthesiologists, October 13, 2021.	Conference abstract
NICE guideline: Preterm labor and birth. 10 June 2022	Not providing specific recommendations for obstetric anesthesia
Y Gu, YB Xu, CH, Wang, et al. The reading of the United States Society for Obstetric Anesthesia and Perinatology: consensus statement on the management of cardiac arrest in pregnancy. Journal of Chinese Physician 2017; 19(9): 1286-1293.	Secondary interpretation of foreign guidelines, not original guidelines
Interpretation of the American College of Obstetricians and Gynecologists: 2017 Obstetric Analgesia and Anesthesia Practice Guide. Journal of practical obstetrics and gynecology 2017; 33 (8):586-589.	Secondary interpretation of foreign guidelines, not original guidelines
Expert Consensus on Obstetric Anesthesia in China (2008) and (2017)	Replaced by updated versions from the same organization
Hawkins JL. et al. Practice guidelines for obstetric anesthesia: an update report by the American Society of Anesthesiologists Task Force on Obstetric Anesthesia.	Replaced by updated versions from the same organization

For peer review only - http://bmjopen.bmj.com/site/about/guidelines.xhtml

Anesthesiology 2007; 106: 843–63.	
Opderbecke HW. et al. Vereinbarung über die Zusammenarbeit in der operativen Gynäkologie und in der Geburtshilfe. Anästh Intensivmed 1996; 37: 414–8.	Not English
Opderbecke HW. et al. Airway management. Anästh Intensivmed 2004; 45: 302–6.	Not English
Gogarten W. et al. Regionalanästhesieverfahren in der Geburtshilfe. Anästh Intensivmed 2004; 45: 151–3.	Not English
T. Piepho1. et al. S1 guidelines on airway management. Guideline of the German Society of Anesthesiology and Intensive Care Medicine. Anesthetist. 2015 Dec;64 Supple 1:27-40.	Not providing specific recommendations for obstetric anesthesia
The National Institute of Clinical Excellence (NICE) guidelines for cesarean sections: implications for the Anesthetist (2004), (2011) and (2014)	Replaced by updated versions from the same organization
Intrapartum Care: Care of Healthy Women and Their Babies During Childbirth (2014)	Replaced by updated versions from the same organization
Osborne, L. et al. Evidence-based anesthesia: fever of unknown origin in parturient and neuraxial anesthesia.	Replaced by updated versions from the same organization
Kearsley, A. et al. The development of a local guideline for epidural insertion in obstetric patients with signs of maternal sepsis. Anesthesia 2015. 70: 56.	Abstract
Korneliussen, J. et al. Implementation of new guideline recommendations, using ultra-low dose intrathecal morphine, for post-cesarean analgesia and its impact on pain, nausea and pruritus. Acta anesthesiologic Scandinavica 2023. 67: 537-538.	Abstract
Nasser, L. et al. IN SITU SIMULATION TO FACILITATE INTRODUCTION OF A NEW OBSTETRIC RAPID SEQUENCE OF ANESTHESIA GUIDELINE. BMJ Simulation and Technology Enhanced Learning 2017. 3: A3.	Abstract
Cohen, J. et al. Implementation of the Association of Anesthetists safety guideline: neurological monitoring associated with obstetric neuraxial block (2020): improving patient safety in a district general hospital labor ward requires a multidisciplinary and multimodal approach. Anesthesia 2022. 77: 12.	Conference abstract
McGuire, R. et al. Changing of the weather? Introduction of an evidence-based guideline for obstetric general anesthesia after regional surveys and the use of simulation to facilitate guideline implementation at University	Conference abstract

For peer review only - http://bmjopen.bmj.com/site/about/guidelines.xhtml

Hospital South Manchester. Anesthesia 2017. 72: 87.	
Han, W. Y. et al. Anesthesia and perioperative management for cesarean section in patient with factor XI deficiency: a case report. Beijing da xue xue bao. Yi xue ban. Health sciences 2014. 46: 329-332.	Case report
McDonnell, N. J. et al. Acute magnesium toxicity in an obstetric patient undergoing general anesthesia for cesarean delivery. International journal of obstetric anesthesia 2010. 19: 226-231.	Case report
Oji-Zurmeyer, J. et al. National survey of obstetric anesthesia clinical practices in the republic of Austria. International journal of obstetric anesthesia 2019. 39: 95-98.	Primary study
Terblanche, N. C. et al. A survey of Anesthetists on uterotonic usage practices for elective cesarean section in Australia and New Zealand. Anesthesia and intensive care 2021. 49: 440-447.	Primary study
Terfasa, E. A. et al. Obstetric analgesia utilization in labor pain management and associated factors among obstetric care providers in the West Shewa Zone, Central Ethiopia. SAGE open medicine 2022. 10: 20503121221088705.	Primary study
Thuillier, C. et al. Impact of recommended changes in labor management for prevention of the primary cesarean delivery. American journal of obstetrics and gynecology 2018. 218: 341.e341- e349.	Primary study
Cobb, B. et al. Do obstetric Anesthesiologists provide more guideline-concordant care for patients undergoing cesarean delivery? Anesthesia and analgesia 2018. 126: 404-408.	Primary study
Fiedler, A. et al. Adverse effect of delayed pushing on postpartum blood loss in nulliparous women with epidural analgesia. International journal of gynecology and obstetrics: the official organ of the International Federation of Gynecology and Obstetrics 2020. 150: 92-97.	Primary study
Gunka, V. et al. Determination of ChloraPrep® drying time before neuraxial anesthesia in elective cesarean delivery. A prospective observational study. International journal of obstetric anesthesia 2019. 38: 19-24.	Primary study
Kinsella, S. M. et al. Category-1 cesarean section: a survey of anesthetic and peri-operative management in the UK. Anesthesia 2010. 65: 362-368.	Primary study
Lee, E. K. et al. Investigating a Needle-Based Epidural Procedure in Obstetric Anesthesia. AMIA ... Annual Symposium proceedings. AMIA Symposium 2018. 2018: 720-729.	Primary study
Nasrallah, F. K. et al. The 30-minute decision-to-incision interval for emergency cesarean delivery: fact or fiction? American journal of perinatology 2004. 21: 63-68.	Primary study
Ward, C. et al. Does the presence of epidural analgesia reduce the risk of neonatal sepsis in the setting of an	Primary study

For peer review only - http://bmjopen.bmj.com/site/about/guidelines.xhtml

intrapartum fever? The journal of maternal-fetal & neonatal medicine: the official journal of the European Association of Perinatal Medicine, the Federation of Asia and Oceania Perinatal Societies, the International Society of Perinatal Obstet 2022. 35: 2110-2115.	
Jejaw, M. et al. Comprehensive emergency management of obstetric and newborn care program implementation at University of Gondar Comprehensive Specialized Hospital, Northwest Ethiopia, 2021: an evaluation study. Reproductive health 2023. 20: 76.	Primary study
Ji, H. et al. Factors contributing to the rapid rise of cesarean section: a prospective study of primiparous Chinese women in Shanghai. BMJ open 2015. 5: e008994.	Primary study
Johri, M. et al. A cluster-randomized trial to reduce cesarean delivery rates in Quebec: cost-effectiveness analysis. BMC medicine 2017. 15: 96.	Primary study
McDougall, A. R. A. et al. Expert consensus on novel medicines to prevent preterm birth and manage preterm labor: Target product profiles. BJOG: an international journal of obstetrics and gynecology 2022.	Not providing specific recommendations for obstetric anesthesia
Sultan, P. et al. Expert Consensus Regarding Core Outcomes for Enhanced Recovery after Cesarean Delivery Studies: A Delphi Study. Anesthesiology 2022. 137: 201-211.	Not providing specific recommendations for obstetric anesthesia
Bremerich, D. et al. S1 Guideline: Obstetric analgesia and anesthesia. Anesthesiology und Intensivmedizin 2020. 61: S300-S340.	Not English
Lavery, J. P. et al. Is the obstetric guideline of 30 minutes from decision to incision for Cesarean delivery clinically significant? Journal of healthcare risk management: the journal of the American Society for Healthcare Risk Management 1999. 19: 11-20.	Pre 2000
Santerre, R. E. The effect of the ACOG guideline on vaginal births after cesarean. Medical care research and review : MCRR 1996. 53: 315-329.	Pre 2000
Morris, J. M. et al. Protocol for the immediate delivery versus expectant care of women with preterm prelabor rupture of the membranes close to term (PPROMT) Trial [ISRCTN44485060]. BMC pregnancy and childbirth 2006. 6: 9.	Protocol
Gochnour, G. et al. The UTAH VBAC Study. Maternal and child health journal 2005. 9: 181-188.	Review
King, V. J. et al. Clinical practice guideline executive summary: Labor after cesarean/planned vaginal birth after	Review

For peer review only - http://bmjopen.bmj.com/site/about/guidelines.xhtml

cesarean. Annals of family medicine 2015. 13: 80-81.	
Keag, O. E. et al. Long-term risks and benefits associated with cesarean delivery for mother, baby, and subsequent pregnancies: Systematic review and meta-analysis. PLoS medicine 2018. 15: e1002494.	Review
Gulbransen, K. et al. Scoping Review of Best Practice Guidelines for Care in the Labor and Birth Setting of Pregnant Women Who Use Methamphetamines. Journal of obstetric, gynecologic, and neonatal nursing: JOGNN 2022. 51: 141-152.	Review
Hocaloski, S. et al. Perinatal Care for Women with Spinal Cord Injuries: A Collaborative Workshop for Consensus on Care in Canada. Topics in spinal cord injury rehabilitation 2017. 23: 386-396.	Review
Hong, J. et al. Comparison of Maternal Labor-Related Complications and Neonatal Outcomes Following Elective Induction of Labor at 39 Weeks of Gestation vs Expectant Management: A Systematic Review and Meta-analysis. JAMA network open 2023. 6: e2313162.	Review
Indermuhle, P. et al. Use of Scheduled Nonopioid Analgesia to Decrease Inpatient Opioid Consumption After Scheduled Cesarean Birth. Nursing for women's health 2022. 26: 344-352.	Review
James, B. et al. Obstetric general anesthetic safety checklist: Guideline development through team simulation. International journal of obstetric anesthesia 2015. 24: S24.	Review
Kuehn, B. M. Obstetrics group relaxes guideline for trial of labor after cesarean delivery. Jama 2010. 304: 951-952.	Review
Lagro-Janssen, T. Pain management during labor--an informed choice. Nederlands tijdschrift voor geneeskunde 2009. 153: A742.	Review
Mash, R. et al. Alternative mechanisms for delivery of medication in South Africa: A scoping review. South African family practice : official journal of the South African Academy of Family Practice/Primary Care 2021. 63: e1-e8.	Review
Melman, S. et al. Identification of barriers and facilitators for optimal cesarean section care: perspective of professionals. BMC pregnancy and childbirth 2017. 17: 230.	Review
Morris, S. et al. Breech presentation management: A critical review of leading clinical practice guidelines. Women and birth : journal of the Australian College of Midwives 2022. 35: e233-e242.	Review
Mutlu, S. et al. Evaluation of pregnancy, delivery, and postpartum effectiveness of maternity school trainings organized based on the guideline of ministry of health in Turkey: A comparative study. International journal of	Review

For peer review only - http://bmjopen.bmj.com/site/about/guidelines.xhtml

preventive medicine 2021. 12: 173.	
Heslehurst, N. et al. GestationaL Obesity Weight management: Implementation of National Guidelines (GLOWING): a pilot cluster randomized controlled trial of a guideline implementation intervention for the management of maternal obesity by midwives. Pilot and feasibility studies 2018. 4: 47.	Review
Lyerly, A. D. et al. Toward an ethically responsible approach to vaginal birth after cesarean. Seminars in perinatology 2010. 34: 337-344.	Review
Nasser, L. et al. Farewell thiopentone? Northwest deanery surveys of obstetric general anesthesia highlight increasing popularity of induction with propofol. Would a region-wide guideline improve training and safety? Anesthesia 2017. 72: 94.	Review
Neuhaus, S. et al. "Why mothers die". Learning from the analysis of anesthesia-related maternal deaths (1985-2013). Der Anesthetist 2016. 65: 281-294.	Review
Panda, S. et al. Review of cesarean section on maternal request in a tertiary care institute; scenario in developing country. Kathmandu University medical journal (KUMJ) 2013. 11: 349-354.	Review
Reale, S. C. et al. The Most Influential Publications in Obstetric Anesthesiology, 1998-2017: Utilizing the Delphi Method for Expert Consensus. Anesthesia and analgesia 2020. 131: 239-244.	Review
Roderick, E. et al. A national survey of neurological monitoring practice after obstetric regional anesthesia in the UK. Anesthesia 2017. 72: 755-759.	Review
Rungreungvanich, M. et al. An analysis of intraoperative recall of awareness in Thai Anesthesia Incidents Study (THAI Study). Journal of the Medical Association of Thailand 2005. 88 Supple 7: S95-101.	Review
Sakoda, A. et al. Review of 197 cases of urgent cesarean section performed in 2010 using NICE classification. Masui. The Japanese journal of Anesthesiology 2014. 63: 1339-1343.	Review
Sommerness, S. A. et al. The Perinatal Birth Environment: Communication Strategies and Processes for Adherence to a Standardized Guideline in Women Undergoing Second-Stage Labor With Epidural Anesthesia. The Journal of perinatal & neonatal nursing 2017. 31: 41-50.	Review
Terui, K. Obstetric anesthesia: from basics to recent advances in neonatal resuscitation: from Apgar score to NCPR program. Masui. The Japanese journal of Anesthesiology 2010. 59: 293-302.	Review
Van Gorp, S. F. J. et al. Do we need a national guideline for obstetric analgesia in the Netherlands? Evaluation of a questionnaire among dutch Anesthetists. Nederlands Tijdschrift voor Anesthesiology 2005. 18: 100-103.	Review

For peer review only - http://bmjopen.bmj.com/site/about/guidelines.xhtml

Williamson, R. M. et al. Availability of lipid emulsion in obstetric anesthesia in the UK: a national questionnaire survey. Anesthesia 2008. 63: 385-388.	Review
Winther, L. P. et al. Failed intubation and subsequent airway management during Cesarean section in general anesthesia. Ugeskrift for laeger 2010. 172: 1914-1916.	Review
Friedman, C. L. et al. Resident Attitudes Towards Cesarean Delivery in Canadian Obstetrics and Gynecology Residency Programs. Journal of obstetrics and gynecology. JOGC 2020. 42: 16-24.	Review
Bagou, G. et al. Guidelines for the management of urgent obstetric situations in emergency medicine, 2022. Anesthesia, critical care & pain medicine 2022. 41: 101127.	Not providing specific recommendations for obstetric anesthesia
Crawford, C. New guideline covers labor, vaginal birth after cesarean. Annals of family medicine 2015. 13: 186-187.	Not providing specific recommendations for obstetric anesthesia
Elammary, M. N. et al. Middle eastern college of obstetricians and gynecologists (MCOG) practice guidelines: Role of prediction models in management of trial of labor after cesarean section. Practice guideline no. 05-O-22. Journal of gynecology obstetrics and human reproduction 2023. 52: 102598.	Not providing specific recommendations for obstetric anesthesia
Fischer, T. et al. From the OEGGG - S3 Guideline "Vaginal delivery at term": The position of the Austrian Society of Gynecology and Obstetrics (OEGGG). Geburtshilfe und Frauenheilkunde 2021. 81: 857-861.	Not providing specific recommendations for obstetric anesthesia
Gagnon, R. et al. SOGC CLINICAL PRACTICE GUIDELINE: guidelines for the management of vasa previa. International journal of gynecology and obstetrics: the official organ of the International Federation of Gynecology and Obstetrics 2010. 108: 85-89.	Not providing specific recommendations for obstetric anesthesia
Hey, J. Clinical practice guideline on vaginal delivery of breech presentation. Journal of obstetrics and gynecology Canada. 2009. 31: 1123.	Not providing specific recommendations for obstetric anesthesia
Jain, V. et al. Guideline No. 402: Diagnosis and Management of Placenta Previa. Journal of obstetrics and gynecology Canada. 2020. 42: 906-917.e901.	Not providing specific recommendations for obstetric anesthesia
Jain, V. et al. Guidelines for the Management of a Pregnant Trauma Patient. Journal of obstetrics and	Not providing specific

For peer review only - http://bmjopen.bmj.com/site/about/guidelines.xhtml

gynecology Canada. 2015. 37: 553-574.	recommendations for obstetric anesthesia
Jain, V. et al. Guideline No. 439: Diagnosis and Management of Vasa Previa. Journal of obstetrics and gynecology Canada. 2023.	Not providing specific recommendations for obstetric anesthesia
Ladhani, N. N. N. et al. No. 347-Obstetric Management at Borderline Viability. Journal of obstetrics and gynecology Canada. 2017. 39: 781-791.	Not providing specific recommendations for obstetric anesthesia
Lalonde, A. B. Vaginal breech delivery guideline: the time has come. Journal of obstetrics and gynecology Canada. 2009. 31: 483-486.	Not providing specific recommendations for obstetric anesthesia
Marte, K. et al. Reduction in the Cesarean Delivery Rate after Obstetric Care Consensus Guideline Implementation. Obstetrics and gynecology 2016. 128: 1445.	Not providing specific recommendations for obstetric anesthesia
Maxwell, C. et al. SOGC Clinical Practice Guideline. Management guidelines for obstetric patients and neonates born to mothers with suspected or probable severe acute respiratory syndrome (SARS). No. 225, April 2009. International journal of gynecology and obstetrics: the official organ of the International Federation of Gynecology and Obstetrics 2009. 107: 82-86.	Not providing specific recommendations for obstetric anesthesia
Nelson, G. et al. Guideline for perioperative obstetrical care highlights evidence gap related to timing of urinary catheter removal after elective cesarean delivery. American journal of obstetrics and gynecology 2020. 222: 635.	Not providing specific recommendations for obstetric anesthesia
Oliver, E. et al. Comparison of international guidelines on the management of twin pregnancy. European journal of obstetrics, gynecology, and reproductive biology 2023. 285: 97-104.	Not providing specific recommendations for obstetric anesthesia
Podrasky, A. E. et al. ACR appropriateness Criteria® second and third trimester bleeding. Ultrasound quarterly 2013. 29: 293-301.	Not providing specific recommendations for obstetric anesthesia
Robinson, D. et al. Guideline No. 431: Postpartum Hemorrhage and Hemorrhagic Shock. Journal of obstetrics	Not providing specific

For peer review only - http://bmjopen.bmj.com/site/about/guidelines.xhtml

and gynecology Canada. 2022. 44: 1293-1310.e1291.	recommendations for obstetric anesthesia
Scott-Warren, V. et al. Development of a fibrinogen concentrate guideline for use in major obstetric Hemorrhage. Transfusion Medicine 2016. 26: 30.	Not providing specific recommendations for obstetric anesthesia
Stafford, I. A. et al. Association between Maternal Obesity Class, Adherence to Labor Guidelines, and Perinatal Outcomes. AJP reports 2021. 11: e105-e112.	Not providing specific recommendations for obstetric anesthesia
Triebwasser, J. et al. Effect of Single-Unit Transfusion Guideline on Red Blood Cell Transfusion for Obstetric Patients. Obstetrics and gynecology 2023. 141: 59S.	Not providing specific recommendations for obstetric anesthesia
Zhu, L. et al. The Chinese guideline for prevention of pelvic and abdominal adhesions after obstetric and gynecologic surgery (2023 edition). Zhonghua fu chan ke za zhi 2023. 58: 161-169.	Not providing specific recommendations for obstetric anesthesia
Bonnet, M. P. et al. Reprint of: Severe pre-eclampsia: guidelines for clinical practice from the French Society of anesthesiology and intensive care (SFAR) and the French College of gynaecologists and obstetricians (CNGOF). Gynecol Obstet Fertil Senol. 2022 Jan;50(1):2-25.	Not English

Reason for exclusion: Review; Primary study; Editorial; Conference abstract; Not providing specific recommendations for obstetric anesthesia; Multimedia section; Replaced by updated versions from the same organization; Conference abstract; Comment letter; Technology appraisal; Book; Not released by a professional association.

For peer review only - http://bmjopen.bmj.com/site/about/guidelines.xhtml

Supplementary methods. How to calculate the scores.

The reviewers individually assessed each item using a Likert scale, and the scores were combined to calculate the domain scores. The total score for each domain was calculated by summing the scores of all the items within that domain for each reviewer. This total score was then standardized as a percentage of the maximum possible score for that specific domain. The calculation method is as follows:

$$\frac{Obtained\ score - Minimum\ possible\ score}{Maximum\ possible\ score - Minimum\ possible\ score} * 100\%$$

The minimum possible score is calculated as: (number of questions) × (number of reviewers) × 1. The maximum possible score is calculated as: (number of questions) × (number of reviewers) × 7.

Five guidance documents were selected for the pilot scoring phase, and discussions were held to clarify scoring criteria. After completing scoring for all included documents, a meeting was held to discuss items with scores differing by more than one point. Reviewers were then given the opportunity to revise their scores or maintain their original scores, with all original and revised scores, along with reasons for modifying scores, meticulously recorded for quality control purposes.

For peer review only - http://bmjopen.bmj.com/site/about/guidelines.xhtml

Supplementary Table 6. Scores for each individual AGREE II domain items by each reviewer.

Document	Domain 1			Domain 2			Domain 3								Domain 4			Domain 5				Domain 6	
**Item**	**1**	**2**	**3**	**4**	**5**	**6**	**7**	**8**	**9**	**10**	**11**	**12**	**13**	**14**	**15**	**16**	**17**	**18**	**19**	**20**	**21**	**22**	**23**
OG-OG-CMA_2014																							
Rev-1	4	4	7	2	1	3	1	1	1	1	3	1	1	1	5	1	1	1	1	1	1	1	1
Rev-2	4	3	7	2	3	2	1	1	1	1	3	1	1	1	5	1	1	1	1	1	1	1	1
Rev-3	4	3	7	3	3	2	1	1	1	1	4	1	1	1	5	1	1	1	1	1	1	1	1
Rev-4	3	4	7	2	1	3	1	1	1	1	3	1	1	1	4	1	1	1	1	1	1	1	1
OG-A-CMA_2016																							
Rev-1	5	6	5	6	6	3	1	1	1	1	6	1	1	1	5	6	3	1	5	1	1	1	1
Rev-2	6	4	4	6	6	4	1	1	1	1	6	1	1	1	6	6	3	1	4	1	1	1	1
Rev-3	5	6	5	6	6	3	1	1	1	1	5	1	1	1	5	6	3	1	4	1	1	1	1
Rev-4	5	4	5	6	6	3	1	1	1	1	6	1	1	1	5	5	3	1	4	1	1	1	1
ASA_2016																							
Rev-1	7	7	7	7	6	7	7	7	6	7	7	6	6	6	6	6	6	6	6	1	1	6	6
Rev-2	7	7	7	7	7	7	7	6	6	7	7	6	7	6	6	6	6	6	6	1	1	6	6
Rev-3	7	7	7	6	6	7	7	7	6	7	6	6	6	6	6	6	5	6	6	1	1	5	6
Rev-4	7	7	7	7	7	6	7	6	6	7	7	6	7	6	6	5	6	6	6	1	1	5	6
AAGBI_2017																							
Rev-1	5	5	5	6	1	1	2	2	2	4	2	1	1	1	6	5	5	2	1	1	1	2	3
Rev-2	5	5	5	6	1	1	2	2	2	4	2	1	1	1	6	5	5	2	1	1	1	2	3
Rev-3	4	5	5	5	1	1	3	2	2	5	2	1	1	1	5	6	5	2	1	1	1	3	2
Rev-4	5	5	6	6	1	1	2	3	2	4	3	1	1	1	6	5	6	2	1	1	1	2	3
ERAS_2018 (Part 1)																							
Rev-1	6	6	6	5	4	4	6	6	6	6	6	6	4	3	5	5	5	2	5	1	2	2	6
Rev-2	5	6	5	5	4	3	6	6	5	6	6	5	3	3	5	5	4	2	5	1	2	3	5
Rev-3	5	6	6	5	4	3	6	5	6	5	6	6	4	3	5	3	5	2	5	1	2	2	6
Rev-4	6	5	6	5	3	4	6	6	6	6	5	6	3	3	6	3	5	2	5	1	2	2	5

For peer review only - http://bmjopen.bmj.com/site/about/guidelines.xhtml

ERAS_2018 (Part 2)																							
Rev-1	6	6	6	5	4	4	6	6	6	6	6	6	4	3	5	5	5	2	5	1	2	2	6
Rev-2	6	6	4	5	3	4	5	6	4	6	5	6	4	3	5	6	5	2	4	1	2	3	6
Rev-3	5	5	6	5	4	3	5	6	5	6	6	5	4	3	5	5	6	2	5	1	2	2	5
Rev-4	6	6	5	5	4	4	6	6	5	6	6	6	4	3	4	6	5	2	6	1	3	3	6
ERAS_2018 (Part 3)																							
Rev-1	6	6	6	5	4	4	6	6	6	6	6	6	4	3	5	5	5	3	5	1	2	1	6
Rev-2	6	6	5	3	4	5	6	6	5	6	5	6	3	3	5	4	5	4	5	1	3	1	5
Rev-3	5	6	6	5	4	4	5	6	6	5	6	5	4	3	6	5	6	3	6	1	2	1	6
Rev-4	6	5	6	6	4	4	6	5	6	6	6	6	3	3	5	6	5	4	5	1	3	1	5
ACOG_2019																							
Rev-1	7	6	5	5	4	5	6	6	5	5	5	4	3	5	6	6	5	4	4	1	1	6	6
Rev-2	7	5	5	4	4	4	6	5	5	4	4	3	3	4	6	6	4	5	5	1	1	5	6
Rev-3	7	6	6	5	5	5	5	6	4	5	5	4	4	5	5	6	5	4	4	1	1	6	5
Rev-4	7	6	5	5	4	5	6	5	5	5	4	3	3	5	6	5	4	4	4	1	1	5	5
SOGC_2019 (Delivery and Postpartum Care)																							
Rev-1	6	6	7	6	3	7	7	6	6	6	6	6	3	6	6	6	6	4	3	1	1	2	6
Rev-2	5	5	7	5	3	7	7	5	5	5	6	5	4	5	4	5	5	3	3	1	1	2	6
Rev-3	6	5	6	5	4	7	7	5	4	5	5	6	3	5	5	6	5	4	4	1	1	2	5
Rev-4	5	5	6	6	3	7	7	5	6	6	5	5	4	6	6	5	6	3	3	1	1	2	6
SOGC_2022 (Twin Pregnancies)																							
Rev-1	6	6	6	6	3	7	7	6	6	6	6	6	5	6	6	6	6	4	3	1	1	2	6
Rev-2	5	5	6	6	3	7	7	5	5	6	6	5	4	5	6	5	5	3	3	1	1	2	6
Rev-3	5	5	5	5	4	6	7	5	4	6	5	6	4	5	5	6	5	4	4	1	1	2	5
Rev-4	6	6	6	5	3	7	7	5	6	6	5	5	5	6	6	5	6	4	3	1	1	2	6
SOAP_2019																							
Rev-1	6	6	5	6	6	5	4	4	4	4	5	5	6	3	5	5	5	4	3	1	1	2	7

For peer review only - http://bmjopen.bmj.com/site/about/guidelines.xhtml

Rev-2	6	5	5	6	6	5	3	4	4	4	5	5	6	3	5	6	5	3	3	1	1	2	6
Rev-3	5	5	5	5	5	5	4	4	3	4	5	6	5	3	5	5	5	4	3	1	1	2	7
Rev-4	6	5	5	6	6	5	4	4	4	4	5	5	6	3	5	6	5	4	3	1	1	2	6
AA and OAA_2020																							
Rev-1	5	5	5	4	1	1	2	1	1	1	1	1	1	1	4	4	5	2	1	1	1	6	6
Rev-2	5	5	4	6	1	1	2	1	1	1	1	1	1	1	4	3	4	2	2	1	1	5	6
Rev-3	6	5	4	4	1	1	2	1	1	1	1	1	1	1	4	4	5	2	1	1	1	5	5
Rev-4	5	5	4	5	1	1	2	1	1	1	1	1	1	1	4	3	5	2	2	1	1	6	6
PROSPECT_2020																							
Rev-1	5	6	5	2	1	2	7	7	6	7	7	7	1	1	7	6	7	6	1	1	1	6	6
Rev-2	5	5	5	2	1	2	7	7	7	7	7	7	3	1	6	6	6	6	3	1	1	6	6
Rev-3	5	6	5	2	1	2	7	7	6	6	7	7	1	1	6	6	6	5	1	1	1	5	6
Rev-4	5	5	5	2	1	2	7	7	7	7	6	7	3	1	6	6	6	6	3	1	1	6	5
CHBSA_2020																							
Rev-1	5	5	5	4	3	2	1	1	1	1	4	1	1	1	5	5	5	2	1	1	1	1	1
Rev-2	5	5	5	4	2	2	1	1	1	1	4	1	1	1	5	5	4	2	1	1	1	1	1
Rev-3	5	6	5	4	3	2	1	1	1	1	3	1	1	1	4	5	5	2	1	1	1	1	1
Rev-4	5	5	5	4	2	2	1	1	1	1	3	1	1	1	5	4	4	2	1	1	1	1	1
CAA_2020 (spinal)																							
Rev-1	5	6	6	5	1	7	1	1	1	1	3	1	1	1	6	6	6	1	5	1	1	1	1
Rev-2	6	6	6	5	1	6	1	1	1	1	2	1	1	1	5	5	5	1	4	1	1	1	1
Rev-3	5	6	5	5	1	6	1	1	1	1	2	1	1	1	5	6	5	1	4	1	1	1	1
Rev-4	5	6	6	4	1	6	1	1	1	1	2	1	1	1	5	5	5	1	4	1	1	1	1
CAA_2020 (delivery analgesia)																							
Rev-1	6	6	5	5	2	4	1	1	1	1	3	1	1	1	7	6	6	1	3	1	1	1	1
Rev-2	6	6	6	6	2	5	1	1	1	1	4	1	1	1	7	6	6	1	2	1	1	1	1
Rev-3	5	6	5	5	2	4	1	1	1	1	3	1	1	1	7	6	6	1	2	1	1	1	1

For peer review only - http://bmjopen.bmj.com/site/about/guidelines.xhtml

Rev-4	6	6	5	6	2	4	1	1	1	1	4	1	1	1	7	6	6	1	2	1	1	1	1
SOAP_2020																							
Rev-1	5	5	5	6	3	2	6	5	7	6	2	3	1	1	6	6	7	5	3	1	1	1	6
Rev-2	6	6	5	7	4	2	6	6	7	6	4	4	1	1	6	6	6	6	3	1	1	1	6
Rev-3	5	5	5	6	3	2	6	5	6	5	3	4	1	1	5	6	5	6	2	1	1	1	5
Rev-4	5	6	6	7	3	2	6	6	7	6	4	4	1	1	6	5	6	6	3	1	1	1	6
NICE_2021																							
Rev-1	5	7	7	6	6	5	1	1	1	1	6	1	1	7	3	5	3	3	4	1	1	1	1
Rev-2	6	6	7	7	6	6	1	1	1	1	7	1	1	7	5	6	6	5	6	1	1	1	1
Rev-3	6	7	7	6	5	5	1	1	1	1	7	1	1	6	3	4	4	3	4	1	1	1	1
Rev-4	6	6	7	7	6	6	1	1	1	1	7	1	1	7	5	6	6	5	6	1	1	1	1
OAA_2022																							
Rev-1	5	5	4	6	1	4	5	4	1	4	4	1	1	1	3	3	5	5	3	1	1	2	4
Rev-2	5	5	6	6	1	3	6	3	2	3	4	1	1	1	5	5	5	4	2	1	1	4	5
Rev-3	5	5	5	5	1	4	6	4	1	5	3	1	1	1	4	5	5	4	3	1	1	4	4
Rev-4	6	5	6	6	1	3	6	2	2	3	4	1	1	1	5	5	5	4	2	1	1	4	5
ANNA_2023																							
Rev-1	6	6	5	6	3	5	1	1	1	2	1	1	1	3	6	6	5	5	2	1	1	6	6
Rev-2	6	6	6	6	2	6	1	1	1	2	1	1	1	2	7	7	6	5	3	1	1	6	6
Rev-3	5	5	5	5	3	6	1	1	1	2	1	1	1	4	6	6	6	5	2	1	1	6	6
Rev-4	6	5	6	6	2	6	1	1	1	2	1	1	1	2	7	7	6	5	3	1	1	5	6
RCoA_2023																							
Rev-1	7	6	6	7	3	7	6	7	7	7	7	5	5	7	6	6	7	6	6	6	5	7	7
Rev-2	6	6	7	7	4	6	5	7	7	7	7	6	6	6	7	7	7	6	6	6	4	6	6
Rev-3	7	5	6	7	4	6	6	7	7	7	7	6	5	7	7	6	7	6	6	5	5	6	6
Rev-4	6	6	7	7	4	6	5	7	7	7	7	6	6	6	7	7	7	6	5	6	5	6	6
French_2021																							

For peer review only - http://bmjopen.bmj.com/site/about/guidelines.xhtml

Rev-1	6	4	6	6	3	4	1	1	1	4	1	1	4	1	5	3	5	1	3	1	1	1	4
Rev-2	6	5	7	7	2	5	1	1	1	5	2	1	5	1	6	4	6	2	2	1	1	1	5
Rev-3	5	5	6	7	3	5	1	1	1	6	1	1	5	1	6	3	6	3	3	1	1	1	5
Rev-4	7	4	7	7	4	6	1	1	1	5	1	1	4	1	7	2	7	1	2	1	1	1	4

For peer review only

For peer review only - http://bmjopen.bmj.com/site/about/guidelines.xhtml

Supplementary Table 7. The intraclass correlation coefficient between reviewers for each individual AGREE II domain item.

**Document**	**The intraclass correlation coefficient (95% confidence interval)**	***P* valve**
OG-OG-CMA_2014	0.987 (0.975, 0.994)	<0.001
OG-A-CMA_2016	0.993 (0.986, 0.997)	<0.001
ASA_2016	0.989 (0.980, 0.995)	<0.001
AAGBI_2017	0.990 (0.982, 0.995)	<0.001
ERAS_2018 (Part 1)	0.975 (0.952, 0.988)	<0.001
ERAS_2018 (Part 2)	0.971 (0.945, 0.986)	<0.001
ERAS_2018 (Part 3)	0.967 (0.938, 0.985)	<0.001
ACOG_2019	0.970 (0.942, 0.986)	<0.001
SOGC_2019 (Delivery and Postpartum Care)	0.974 (0.950, 0.988)	<0.001
SOGC_2022 (Twin Pregnancies)	0.976 (0.953, 0.989)	<0.001
SOAP_2019	0.986 (0.974, 0.994)	<0.001
AA and OAA_2020	0.991 (0.983, 0.996)	<0.001
PROSPECT_2020	0.990 (0.981, 0.995)	<0.001
CHBSA_2020	0.994 (0.988, 0.997)	<0.001
CAA_2020 (spinal)	0.995 (0.989, 0.998)	<0.001
CAA_2020 (delivery analgesia)	0.996 (0.993, 0.998)	<0.001
SOAP_2020	0.987 (0.972, 0.994)	<0.001
NICE_2021	0.984 (0.966, 0.993)	<0.001
OAA_2022	0.970 (0.944, 0.986)	<0.001
ANNA_2023	0.991 (0.984, 0.996)	<0.001
RCoA_2023	0.925 (0.859, 0.965)	<0.001
French_2021	0.941 (0.905, 0.986)	<0.001

For peer review only - http://bmjopen.bmj.com/site/about/guidelines.xhtml

Supplementary Table 8. Domain score for each included guidance document.

Document	Domain 1, %	Domain 2, %	Domain 3, %	Domain 4, %	Domain 5, %	Domain 6, %
OG-OG-CMA_2014	62.5	20.8	6.6	20.8	0.0	0.0
OG-A-CMA_2016	66.7	68.1	11.7	61.1	13.5	0.0
ASA_2016	100.0	94.4	91.3	80.6	41.7	79.2
AAGBI_2017	66.7	26.4	18.4	73.6	4.2	25.0
ERAS_2018 (Part 1)	77.8	51.4	69.9	61.1	25.0	47.9
ERAS_2018 (Part 2)	76.4	52.8	69.4	69.4	26.0	52.1
ERAS_2018 (Part 3)	79.2	55.6	69.4	69.4	34.4	37.5
ACOG_2019	83.3	59.7	60.7	72.2	27.1	75.0
SOGC_2019 (Delivery and Postpartum Care)	79.2	70.8	73.5	73.6	19.8	47.9
SOGC_2022 (Twin Pregnancies)	76.4	69.4	76.5	76.4	20.8	47.9
SOAP_2019	72.2	75.0	56.1	69.4	19.8	54.2
AA and OAA_2020	63.9	20.8	4.1	51.4	6.3	77.1
PROSPECT_2020	69.4	11.1	75.5	86.1	24.0	79.2
CHBSA_2020	68.1	30.6	7.1	61.1	4.2	0.0
CAA_2020 (spinal)	77.8	50.0	4.6	72.2	13.5	0.0
CAA_2020 (delivery analgesia)	77.8	48.6	7.1	88.9	5.2	0.0
SOAP_2020	72.2	48.6	53.1	80.6	27.1	39.6
NICE_2021	90.3	81.9	25.5	61.1	29.2	0.0
OAA_2022	69.4	40.3	28.6	59.7	19.8	50.0
ANNA_2023	76.4	61.1	7.7	87.5	22.9	81.3
RCoA_2023	87.5	77.8	90.3	95.8	76.0	87.5
French_2021	82.35	79.66	55.56	80.0	36.0	63.64

For peer review only

For peer review only - http://bmjopen.bmj.com/site/about/guidelines.xhtml

Supplementary Table 9. Mean scores across reviewers for the individual AGREE II domain items.

Document	The intraclass correlation coefficient, 95% confidence interval																						
	Domain 1			Domain 2			Domain 3								Domain 4			Domain 5				Domain 6	
	1	2	3	4	5	6	7	8	9	10	11	12	13	14	15	16	17	18	19	20	21	22	23
OG-OG-CMA_2014	3.8	4.7	7.0	2.3	2.0	2.5	1.0	1.0	1.0	1.0	3.3	1.0	1.0	1.0	4.8	1.0	1.0	1.0	1.0	1.0	1.0	1.0	1.0
OG-A-CMA_2016	5.3	5.0	6.0	6.0	6.0	3.3	1.0	1.0	1.0	1.0	5.8	1.0	1.0	1.0	5.3	5.8	3.0	1.0	4.3	1.0	1.0	1.0	1.0
ASA_2016	7.0	7.0	7.0	6.8	6.5	6.8	7.0	6.5	6.0	7.0	6.8	6.0	6.5	6.0	6.0	5.8	5.8	6.0	6.0	1.0	1.0	5.5	6.0
AAGBI_2017	4.8	5.0	5.3	5.8	1.0	1.0	2.3	2.3	2.0	4.3	2.3	41.0	1.0	1.0	5.8	5.3	5.3	1.0	1.0	1.0	2.3	2.3	2.8
ERAS_2018 (Part 1)	5.5	5.8	5.8	5.0	3.8	3.5	6.0	5.8	5.8	5.8	5.8	5.8	3.5	3.0	5.3	4.0	4.8	2.0	5.0	1.0	2.0	2.3	5.5
ERAS_2018 (Part 2)	5.8	5.8	5.3	5.0	3.8	3.8	5.5	6.0	5.0	6.0	5.8	5.8	4.0	3.0	4.8	5.5	5.3	2.0	5.0	1.0	2.3	2.5	5.8
ERAS_2018 (Part 3)	5.8	5.8	5.8	4.8	4.0	4.3	5.8	5.8	5.8	5.8	5.8	5.8	3.5	3.0	5.3	5.0	5.3	3.5	5.3	1.0	2.5	1.0	5.5
ACOG_2019	7.0	5.8	5.3	4.8	4.3	4.8	5.8	5.5	4.8	4.8	4.5	3.5	3.3	4.8	5.8	5.8	4.5	4.3	4.3	1.0	1.0	5.5	5.5
SOGC_2019 (Delivery and Postpartum Care)	5.5	5.3	6.5	5.5	3.3	7.0	7.0	5.3	5.3	5.5	5.5	5.5	3.5	5.5	5.3	5.5	5.5	3.5	3.3	1.0	1.0	2.0	5.8
SOGC_2022 (Twin Pregnancies)	5.5	5.5	5.8	5.5	3.3	6.8	7.0	5.3	5.3	6.0	5.5	5.5	4.5	5.5	5.8	5.5	5.5	3.8	3.3	1.0	1.0	2.0	5.8
SOAP_2019	5.8	5.3	5.0	5.8	5.8	5.0	3.8	4.0	3.8	4.0	5.0	5.3	5.8	3.0	5.0	5.5	5.0	3.8	3.0	1.0	1.0	2.0	6.5
AA and OAA_2020	5.3	5.0	4.3	4.8	1.0	1.0	2.0	1.0	1.0	1.0	1.0	1.0	1.0	1.0	4.0	3.5	4.8	2.0	1.5	1.0	1.0	5.5	5.8
PROSPECT_2020	5.0	5.5	5.0	2.0	1.0	2.0	7.0	7.0	6.5	6.8	6.8	7.0	2.0	1.0	6.3	6.0	6.3	5.8	2.0	1.0	1.0	5.8	5.8
GHBSA_2020	5.0	5.3	5.0	4.0	2.5	2.0	1.0	1.0	1.0	1.0	3.5	1.0	1.0	1.0	4.8	4.8	4.5	2.0	1.0	1.0	1.0	1.0	1.0
CAA_2020 (spinal)	5.3	6.0	5.8	4.8	1.0	6.3	1.0	1.0	1.0	1.0	2.3	1.0	1.0	1.0	5.3	5.5	5.3	1.0	4.3	1.0	1.0	1.0	1.0
CAA_2020 (delivery analgesia)	5.8	6.0	5.3	5.5	2.0	4.3	1.0	1.0	1.0	1.0	3.5	1.0	1.0	1.0	7.0	6.0	6.0	1.0	2.3	1.0	1.0	1.0	1.0
SOAP_2020	5.3	5.5	5.3	6.5	3.3	2.0	6.0	5.5	6.8	5.8	3.3	3.8	1.0	1.0	5.8	5.8	6.0	5.8	2.8	1.0	1.0	1.0	5.8
NICE_2021	5.8	6.5	7.0	6.5	5.8	5.5	1.0	1.0	1.0	1.0	6.8	1.0	1.0	6.8	4.0	5.3	4.8	4.0	5.0	1.0	1.0	1.0	1.0
OAA_2022	5.3	5.0	5.3	5.8	1.0	3.5	5.8	3.3	1.5	3.8	3.8	1.0	1.0	1.0	4.3	4.5	5.0	4.3	2.5	1.0	1.0	3.5	4.5
ANNA_2023	5.8	5.5	5.5	5.8	2.5	5.8	1.0	1.0	1.0	2.0	1.0	1.0	1.0	2.8	6.5	6.5	5.8	5.0	2.5	1.0	1.0	5.8	6.0
RCoA_2023	6.5	5.8	6.5	7.0	3.8	6.3	5.5	7.0	7.0	7.0	7.0	5.8	5.5	6.5	6.8	6.5	7.0	6.0	5.8	5.8	4.8	6.3	6.3

For peer review only - http://bmjopen.bmj.com/site/about/guidelines.xhtml

French_2021	6.0	4.5	6.5	6.75	3.0	5.0	1.0	1.0	1.0	5.0	1.25	1.0	4.5	1.0	6.0	3.0	6.0	1.75	2.5	1.0	1.0	1.0	4.5

For peer review only

Supplementary Table 10. Summary of recommendations for the peri-anesthetic evaluation and preparation by included guidance document.

		OG-OG-CMA_2014	OG-A-CMA_2016	ASA_2016	AAGBI_2017	ERAS_2018 (Part 1)	ERAS_2018 (Part 2)	ERAS_2018 (Part 3)	ACOG_2019	SOGC_2019	SOGC_2022	SOAP_2019	AA and OAA_2020	PROSPECT_2020	CHBSA_2020	CAA_2020	CAA_2020	SOAP_2020	NICE_2021	OAA_2022	ANNA_2023	RCoA_2023	French_2021
**Recommendations**	**Reference contents**																						
**Peri-anesthetic Evaluation and Preparation**																							
**1. History and physical examination before providing anesthetic care**	Yes	+	+	+	NA	NG	NA	NA	+	NA	NA	NA	NA	NA	+	+	+	NG	+/-	NA	+	NG	+
1.1 Maternal health and anesthetic history	Agree	+	+	+	NA	NG	NA	NA	+	NA	NA	NA	NA	NA	+	+	+	NG	NG	NA	+/-	NG	+
1.2 Relevant obstetric history	Agree	+	+	+	NA	NG	NA	NA	NG	NA	NA	NA	NA	NA	NG	+	+	NG	NG	NA	NG	NG	NG
1.3 Baseline blood pressure measurement	Agree	+	+	+	NA	NG	NA	NA	NG	NA	NA	NA	NA	NA	+	-	+	NG	NG	NA	+/-	NG	NG
1.4 Airway examination	Agree	NG	+	+	NA	NG	NA	NA	+	NA	NA	NA	NA	NA	NG	+	+	NG	NG	NA	+	NG	NG
1.5 Heart and lung examination	Agree	NG	NG	+	NA	NG	NA	NA	+	NA	NA	NA	NA	NA	+	+	+	NG	NG	NA	+	NG	NG

For peer review only - http://bmjopen.bmj.com/site/about/guidelines.xhtml

1.6 When a neuraxial anesthetic is planned, examine the patient's back	Agree	NG	+	+	NA	NG	NA	NA	+	NA	NA	NA	NA	NA	NG	+	+	NG	NG	NA	+	NG	NG
1.7 Consultation between the obstetrician and the anesthesiologist	Agree	+	NG	+	NA	+	NA	NA	+	NA	NA	NA	NA	NA	NG	+	+	NG	NG	NA	+	NG	+
**2. Preoperative communication**	Encouraged	+	NG	+	NA	+	NA	NA	NG	NA	NA	NA	NA	NA	+	+	-	+/-	+	NA	+	+/-	+
**3. Intrapartum platelet count**	Yes	+	+	+	NA	NA	NA	NA	+	NA	NA	NA	NA	NA	NG	+	NG	NG	+	NA	NG	NG	NA
-Routine	Agree	+	+	-	NA	NA	NA	NA	+	NA	NA	NA	NA	NA	NG	NG	NG	NG	-	NA	NG	NG	NA
-Individualized	Agree	-	-	+	NA	NA	NA	NA	+	NA	NA	NA	NA	NA	NG	NG	NG	NG	+	NA	NG	NG	NA
**4. Blood type and screen**	Yes	+	+	+	NA	NA	NA	NA	NG	NA	NA	NA	NA	NA	NG	+	NG	NG	+	NA	NG	NG	NA
-Routine	Agree	+	+	-	NA	NA	NA	NA	NG	NA	NA	NA	NA	NA	NG	+	NG	NG	+	NA	NG	NG	NA
-Individualized	Agree	-	-	+	NA	NA	NA	NA	NG	NA	NA	NA	NA	NA	NG	NG	NG	NG	-	NA	NG	NG	NA
**5. Blood cross-match**	Yes	+	NG	+	NA	NA	NA	NA	NG	NA	NA	NA	NA	NA	NG	NG	NG	NG	+	NA	NG	NG	NA
-Routine	Agree	+	NG	-	NA	NA	NA	NA	NG	NA	NA	NA	NA	NA	NG	NG	NG	NG	-	NA	NG	NG	NA
-Individualized	Agree	-	NG	+	NA	NA	NA	NA	NG	NA	NA	NA	NA	NA	NG	NG	NG	NG	+	NA	NG	NG	NA
**6. Peri-anesthetic recording of fetal heart rate**	Yes	+	+	+	NA	NA	NA	NA	+	+	+	NA	NA	NA	NG	+	+	NG	NG	NA	+	NG	+
**7. Aspiration prevention**	Provided	+	NG	+	NA	+	NA	NG	NA	+	NA	NA	NA	NA	+	+/-	NG	+/-	+/-	NA	+/-	+/-	NA
7.1 Clear liquids	Up to 2 h	NG	NG	Up to 2 h	NA	Up to 2 h	NA	NG	NA	NG	NA	NA	NA	NA	Up to 2 h	+	NG	+	NG	NA	NG	+/-	NA
7.2 Solids	6-8 h	+	NG	6-8 h	NA	Light meal	NA	NG	NA	NG	NA	NA	NA	NA	6 h	+	NG	+	NG	NA	NG	NG	NA

For peer review only - http://bmjopen.bmj.com/site/about/guidelines.xhtml

						to 6 h																	
7.3 Antacids (surgical procedures-cesarean delivery or postpartum tubal ligation)	Agree	NG	NG	+	NA	+	NA	+	NA	NG	NA	NA	NA	NA	+	+	NG	NG	+	NA	NG	+	NA
7.4 H_2-receptor antagonists (surgical procedures)	Agree	NG	NG	+	NA	+	NA	+	NA	NG	NA	NA	NA	NA	+	+	NG	NG	+	NA	NG	NG	NA
7.5 Metoclopramide (surgical procedures)	Agree	NG	NG	+	NA	NG	NA	20 mg	NA	NG	NA	NA	NA	NA	NG	+	NG	NG	NG	NA	NG	NG	NA
**8. Preoperative heparin or low-molecular-weight heparin**	Yes	NA	+	NG	NA	NA	NA	NA	+	NA	NA	NA	NA	NA	NA	NG	+/-	NG	NG	NA	+/-	+/-	NA
8.1 Prophylactic subcutaneous unfractionated heparin (<5,000 units twice daily)	Provided	NA	NG	NG	NA	NA	NA	NA	+	NA	NA	NA	NA	NA	NA	NG	NG	NG	NG	NA	NG	NG	NA
-Contraindication to neuraxial techniques	No	NA	NG	NG	NA	NA	NA	NA	+	NA	NA	NA	NA	NA	NA	NG	NG	NG	NG	NA	NG	NG	NA
-Catheter placement or removal since last dose	4-6 hours	NA	NG	NG	NA	NA	NA	NA	4-6 h	NA	NA	NA	NA	NA	NA	NG	NG	NG	NG	NA	NG	NG	NA
8.2 Intermediate unfractionated heparin (75,000-100,000 units)	Provided	NA	NG	NG	NA	NA	NA	NA	+	NA	NA	NA	NA	NA	NA	NG	NG	NG	NG	NA	NG	NG	NA
-Catheter placement or removal since last dose	> 12 hours	NA	NG	NG	NA	NA	NA	NA	> 12 hours	NA	NA	NA	NA	NA	NA	NG	NG	NG	NG	NA	NG	NG	NA

For peer review only

8.3 High-dose unfractionated heparin (total daily dose greater than 20,000 units)	Provided	NA	NG	NG	NA	NA	NA	NA	+	NA	NA	NA	NA	NA	NA	NG	NG	NG	NG	NA	NG	NG	NA
-Catheter placement or removal since last dose	> 24 hours	NA	NG	NG	NA	NA	NA	NA	> 24 hours	NA	NA	NA	NA	NA	NA	NG	NG	NG	NG	NA	NG	NG	NA
-With the activated partial thromboplastin time being within normal range	Yes	NA	NG	NG	NA	NA	NA	NA	+	NA	NA	NA	NA	NA	NA	NG	NG	NG	NG	NA	NG	NG	NA

+: consistent the reference with content;

+/-: partial1y consistent the reference with content;

-: inconsistent the reference with content;

NA: not applicable;

NG: not given.

For peer review only

Supplementary Table 11. Summary of recommendations for the anesthetic care for labor and vaginal delivery by included guidance document.

Recommendations	Reference contents	OG-OG-CMA_2014	OG-A-CMA_2016	ASA_2016	AAGBI_2017	ERAS_2018 (Part 1)	ERAS_2018 (Part 2)	ERAS_2018 (Part 3)	ACOG_2019	SOGC_2019	SOGC_2022	SOAP_2019	AA and OAA_2020	PROSPECT_2020	CHBSA_2020	CAA_2020	CAA_2020	SOAP_2020	NICE_2021	OAA_2022	ANNA_2023	RCoA_2023	French_2021
**Anesthetic Care for Labor and Vaginal Delivery**																							
**1. Equipment, facilities, and support personnel**	Agree	NA	+	+	NA	NA	NA	NA	+	NA	NA	NA	NA	NA	+	NA	+	NA	NA	NA	+	+	NA
**2. Timing of neuraxial analgesia and outcome of labor**	Provided	NA	+	+	NA	NA	NA	NA	NG	NA	NA	NA	NA	NA	NG	NA	+	NA	NA	NA	NG	NG	NA
2.1 Less 5 cm dilation	Agree	NA	+/-	+	NA	NA	NA	NA	NG	NA	NA	NA	NA	NA	NG	NA	+/-	NA	NA	NA	NG	NG	NA
2.2 Offer neuraxial analgesia on an individualized basis	Agree	NA	+	+	NA	NA	NA	NA	NG	NA	NA	NA	NA	NA	NG	NA	+	NA	NA	NA	NG	NG	NA
**3. Neuraxial analgesia and trial of labor after prior cesarean delivery**	Agree	NA	NG	+	NA	NA	NA	NA	NG	NA	NA	NA	NA	NA	NG	NA	NG	NA	NA	NA	NG	NG	NA
**4. Continuous infusion epidural analgesia**	Provided	NA	+	+	NA	NA	NA	NA	+	NA	+/-	NA	NA	NA	+	NA	+	NA	NA	NA	+	NG	NA
4.1 Using local anesthetic	Agree	NA	+	+	NA	NA	NA	NA	+	NA	NG	NA	NA	NA	+	NA	+	NA	NA	NA	+	NG	NA
4.2 Add opioid	Agree	NA	+	+	NA	NA	NA	NA	+	NA	NG	NA	NA	NA	NG	NA	+	NA	NA	NA	+/-	NG	NA
4.3 Platelet concentration corresponding to	Less than $70*10^9/L$	NA	NG	NG	NA	NA	NA	NA	+	NA	NG	NA	NA	NA	NG	NA	NG	NA	NA	NA	+	NG	NA

For peer review only

contraindications to puncture																							
4.4 Puncture location	L_{2-3} or L_{3-4}	NA	+	NG	NA	NA	NA	NA	NG	NA	NG	NA	NA	NA	NG	NA	+	NA	NA	NA	NG	NG	NA
4.5 Analgesic concentrations	Provided	NA	+	NG	NA	NA	NA	NA	NG	NA	NG	NA	NA	NA	NG	NA	+	NA	NA	NA	+	NG	NA
-Bupivacaine	Provided	NA	+	NG	NA	NA	NA	NA	NG	NA	NG	NA	NA	NA	NG	NA	+	NA	NA	NA	+	NG	NA
--Loading dose	0.04-0.125%	NA	+	NG	NA	NA	NA	NA	NG	NA	NG	NA	NA	NA	NG	NA	+	NA	NA	NA	0.0625-0.125%	NG	NA
--Maintenance dose	0.05-0.125%	NA	+	NG	NA	NA	NA	NA	NG	NA	NG	NA	NA	NA	NG	NA	+	NA	NA	NA	0.0625-0.125%	NG	NA
-Ropivacaine	Provided	NA	+	NG	NA	NA	NA	NA	NG	NA	NG	NA	NA	NA	NG	NA	+	NA	NA	NA	+	NG	NA
--Loading dose	0.0625-0.15%	NA	+	NG	NA	NA	NA	NA	NG	NA	NG	NA	NA	NA	NG	NA	+	NA	NA	NA	0.08-0.2%	NG	NA
--Maintenance dose	0.0625-0.125%	NA	+	NG	NA	NA	NA	NA	NG	NA	NG	NA	NA	NA	NG	NA	+	NA	NA	NA	0.08-0.2%	NG	NA
-Levobupivacaine	Provided	NA	NG	NG	NA	NA	NA	NA	NG	NA	NG	NA	NA	NA	NG	NA	+	NA	NA	NA	NG	NG	NA
--Loading dose	0.04-0.125%	NA	NG	NG	NA	NA	NA	NA	NG	NA	NG	NA	NA	NA	NG	NA	+	NA	NA	NA	NG	NG	NA
--Maintenance dose	0.05-0.125%	NA	NG	NG	NA	NA	NA	NA	NG	NA	NG	NA	NA	NA	NG	NA	+	NA	NA	NA	NG	NG	NA
4.6 Opioids concentrations	Provided	NA	+	NG	NA	NA	NA	NA	NG	NA	NG	NA	NA	NA	NG	NA	+	NA	NA	NA	+/-	NG	NA
-Fentanyl	Provided	NA	+	NG	NA	NA	NA	NA	NG	NA	NG	NA	NA	NA	NG	NA	+	NA	NA	NA	NG	NG	NA
--Loading dose	0.5-2 $\mu g.ml^{-1}$	NA	1-2	NG	NA	NA	NA	NA	NG	NA	NG	NA	NA	NA	NG	NA	+	NA	NA	NA	NG	NG	NA
--Maintenance dose	1-2 $\mu g.ml^{-1}$	NA	+	NG	NA	NA	NA	NA	NG	NA	NG	NA	NA	NA	NG	NA	+	NA	NA	NA	NG	NG	NA
-Sufentanyl	Provided	NA	+	NG	NA	NA	NA	NA	NG	NA	NG	NA	NA	NA	NG	NA	+	NA	NA	NA	NG	NG	NA

--Loading dose	0.2-0.6 $\mu g.ml^{-1}$	NA	0.4-0.6	NG	NA	NA	NA	NA	NG	NA	NG	NA	NA	NA	NG	NA	+	NA	NA	NA	NG	NG	NA
--Maintenance dose	0.3-0.6 $\mu g.ml^{-1}$	NA	0.4-0.6	NG	NA	NA	NA	NA	NG	NA	NG	NA	NA	NA	NG	NA	+	NA	NA	NA	NG	NG	NA
**5. Combined spinal–epidural analgesia**	Provided	NA	+	+	NA	NA	NA	NA	+	NA	NA	NA	NA	NA	NG	NA	+	NA	NA	NA	+/-	NG	NA
5.1 Using local anesthetic	Agree	NA	+	NG	NA	NA	NA	NA	+	NA	NA	NA	NA	NA	NG	NA	+	NA	NA	NA	+	NG	NA
5.2 Add opioid	Agree	NA	+	NG	NA	NA	NA	NA	+	NA	NA	NA	NA	NA	NG	NA	+	NA	NA	NA	+	NG	NA
5.3 Platelet concentration corresponding to contraindications to puncture	Less than $70*10^9/L$	NA	NG	NG	NA	NA	NA	NA	+	NA	NA	NA	NA	NA	NG	NA	+	NA	NA	NA	+	NG	NA
5.4 Puncture location	L_{2-3} or L_{3-4}	NA	+	NG	NA	NA	NA	NA	NG	NA	NA	NA	NA	NA	NG	NA	+/-	NA	NA	NA	NG	NG	NA
5.5 Epidural anesthetics (consistent with 4.5 and 4.6)	Provided	NA	+	NG	NA	NA	NA	NA	NG	NA	NA	NA	NA	NA	NG	NA	+	NA	NA	NA	+/-	NG	NA
5.6 Spinal anesthetics	Provided	NA	+	NG	NA	NA	NA	NA	+	NA	NA	NA	NA	NA	NG	NA	+	NA	NA	NA	NG	NG	NA
-Protocol 1	Provided	NA	+	NG	NA	NA	NA	NA	+	NA	NA	NA	NA	NA	NG	NA	+	NA	NA	NA	NG	NG	NA
--Single sufentanyl	2.5-7 µg	NA	+	NG	NA	NA	NA	NA	+/-	NA	NA	NA	NA	NA	NG	NA	+	NA	NA	NA	NG	NG	NA
--Single ropivacaine	2.3-3 mg	NA	2.5-3	NG	NA	NA	NA	NA	+/-	NA	NA	NA	NA	NA	NG	NA	+	NA	NA	NA	NG	NG	NA
--Combined sufentanyl and ropivacaine	2.5 µg / 2.5 mg	NA	+	NG	NA	NA	NA	NA	+/-	NA	NA	NA	NA	NA	NG	NA	+	NA	NA	NA	Sufentanyl 5-10 µg	NG	NA
-Protocol 2	Provided	NA	+	NG	NA	NA	NA	NA	+/-	NA	NA	NA	NA	NA	NG	NA	+	NA	NA	NA	NG	NG	NA
--Single fentanyl	15-25 µg	NA	+	NG	NA	NA	NA	NA	+/-	NA	NA	NA	NA	NA	NG	NA	+	NA	NA	NA	NG	NG	NA
--Single bupivacaine	2.0-2.5 mg	NA	+	NG	NA	NA	NA	NA	+/-	NA	NA	NA	NA	NA	NG	NA	+	NA	NA	NA	NG	NG	NA
--Combined fentanyl and bupivacaine	2.5 µg / 2.0 mg	NA	+	NG	NA	NA	NA	NA	+/-	NA	NA	NA	NA	NA	NG	NA	+	NA	NA	NA	Fentanyl 50-100 µg	NG	NA

For peer review only - http://bmjopen.bmj.com/site/about/guidelines.xhtml

**6. Single-injection spinal**	Provided	NA	NG	+	NA	NA	NA	NA	+	NA	NA	NA	NA	NA	NG	NA	+	NA	NA	NA	+	NG	NA
6.1 Using local anesthetic	Agree	NA	NG	+	NA	NA	NA	NA	+	NA	NA	NA	NA	NA	NG	NA	+	NA	NA	NA	NG	NG	NA
6.2 Add opioid	Agree	NA	NG	+	NA	NA	NA	NA	+	NA	NA	NA	NA	NA	NG	NA	+	NA	NA	NA	NG	NG	NA
6.3 Platelet concentration corresponding to contraindications to puncture	Less than $70*10^9/L$	NA	NG	NG	NA	NA	NA	NA	+	NA	NA	NA	NA	NA	NG	NA	NG	NA	NA	NA	+	NG	NA
6.4 Puncture location	L_{3-4}	NA	NG	NG	NA	NA	NA	NA	NG	NA	NA	NA	NA	NA	NG	NA	NG	NA	NA	NA	NG	NG	NA
6.6 Analgesic concentrations (consistent with 5.6)	Agree	NA	NG	NG	NA	NA	NA	NA	NG	NA	NA	NA	NA	NA	NG	NA	+	NA	NA	NA	NG	NG	NA
6.7 Spinal needles	Provided	NA	NG	+	NA	NA	NA	NA	NG	NA	NA	NA	NA	NA	NG	NA	NG	NA	NA	NA	+	NG	NA
-Cutting-bevel	Disagree	NA	NG	+	NA	NA	NA	NA	NG	NA	NA	NA	NA	NA	NG	NA	NG	NA	NA	NA	+	NG	NA
-Pencil-point	Agree	NA	NG	+	NA	NA	NA	NA	NG	NA	NA	NA	NA	NA	NG	NA	NG	NA	NA	NA	+	NG	NA
**7. Patient-controlled epidural analgesia (for labor analgesia)**	Agree	NA	+	+	NA	NA	NA	NA	NG	NA	NA	NA	NA	NA	+	NA	+	NA	NA	NA	+	NG	NA

+: consistent the reference with content;

+/-: partial1y consistent the reference with content;

-: inconsistent the reference with content;

NA: not applicable;

NG: not given.

Supplementary Table 12. Summary of recommendations for the anesthetic care for cesarean delivery by included guidance document.

For peer review only - http://bmjopen.bmj.com/site/about/guidelines.xhtml

Recommendations	Reference contents	OG-OG-CMA_2014	OG-A-CMA_2016	ASA_2016	AAGBI_2017	ERAS_2018 (Part 1)	ERAS_2018 (Part 2)	ERAS_2018 (Part 3)	ACOG_2019	SOGC_2019	SOGC_2022	SOAP_2019	AA and OAA_2020	PROSPECT_2020	CHBSA_2020	CAA_2020	CAA_2020	SOAP_2020	NICE_2021	OAA_2022	ANNA_2023	RCoA_2023	French_2021
**Anesthetic Care for Cesarean Delivery**																							
**1. Equipment, facilities, and support personnel**	Agree	+	NA	+	NA	NA	NG	NA	+	+	NA	NA	NA	NA	+	NG	NA	NA	NA	NA	+	+	NA
**2. Continuous infusion epidural analgesia**	Provided	+	NA	+	NA	NA	+/-	NA	+	+/-	NA	NA	NA	NA	+	+	NA	NA	NA	NA	+	NG	NA
2.1 Using local anesthetic	Agree	NG	NA	NG	NA	NA	NG	NA	+	NG	NA	NA	NA	NA	+	+	NA	NA	NA	NA	+	NG	NA
2.2 Add opioid	Agree	NG	NA	NG	NA	NA	NG	NA	+	NG	NA	NA	NA	NA	NG	+	NA	NA	NA	NA	+	NG	NA
2.3 Platelet concentration corresponding to contraindications to puncture	Less than 70*10^9/L	NG	NA	NG	NA	NA	NG	NA	+	NG	NA	NA	NA	NA	NG	+	NA	NA	NA	NA	+	NG	NA
2.4 Puncture location	L_{1-2} or L_{2-3}	NG	NA	NG	NA	NA	NG	NA	NG	NG	NA	NA	NA	NA	NG	+	NA	NA	NA	NA	NG	NG	NA
2.5 Recommend level of block	T_{6-4}	NG	NA	NG	NA	NA	NG	NA	T4	NG	NA	NA	NA	NA	NG	+	NA	NA	NA	NA	NG	NG	NA
2.6 Analgesic concentrations	Provided	NG	NA	NG	NA	NA	NG	NA	NG	NG	NA	NA	NA	NA	NG	+	NA	NA	NA	NA	+/-	NG	NA
-Lidocaine	1.5%-2%	NG	NA	NG	NA	NA	NG	NA	NG	NG	NA	NA	NA	NA	NG	+	NA	NA	NA	NA	NG	NG	NA
-Bupivacaine	0.50%	NG	NA	NG	NA	NA	NG	NA	NG	NG	NA	NA	NA	NA	NG	+	NA	NA	NA	NA	0.0625-0.125%	NG	NA

For peer review only

-Ropivacaine	0.5%-0.75%	NG	NA	NG	NA	NA	NG	NA	NG	NG	NA	NA	NA	NA	NG	+	NA	NA	NA	NA	0.08-0.2%	NG	NA
-Levobupivacaine	0.5%-0.75%	NG	NA	NG	NA	NA	NG	NA	NG	NG	NA	NA	NA	NA	NG	+	NA	NA	NA	NA	NG	NG	NA
-Chloroprocaine	3%	NG	NA	NG	NA	NA	NG	NA	NG	NG	NA	NA	NA	NA	NG	+	NA	NA	NA	NA	NG	NG	NA
2.7 Prevention of local anesthetic poisoning	Provided	NG	NA	NG	NA	NA	NG	NA	+	NG	NA	NA	NA	NA	NG	+	NA	NA	NA	NA	NA	NG	NA
-Withdrawal before injection	Agree	NG	NA	NG	NA	NA	NG	NA	NG	NG	NA	NA	NA	NA	NG	+	NA	NA	NA	NA	NA	NG	NA
-Administration of test dose	Agree	NG	NA	NG	NA	NA	NG	NA	NG	NG	NA	NA	NA	NA	NG	+	NA	NA	NA	NA	NA	NG	NA
-Addition of epinephrine	Agree	NG	NA	NG	NA	NA	NG	NA	+	NG	NA	NA	NA	NA	NG	+	NA	NA	NA	NA	NA	NG	NA
**3. Single-injection spinal**	Provided	NA	NA	+	+/-	NA	+/-	NA	+	+/-	NA	NA	NA	NA	+	+	NA	NA	NA	NA	+	NG	+
3.1 Using local anesthetic	Agree	NA	NA	NG	NG	NA	NG	NA	+	NG	NA	NA	NA	NA	+	+	NA	NA	NA	NA	+	NG	+
3.2 Add opioid	Agree	NA	NA	NG	NG	NA	NG	NA	+	NG	NA	NA	NA	NA	NG	+	NA	NA	NA	NA	+	NG	+
3.3 Platelet concentration corresponding to contraindications to puncture	Less than $70*10^9$/L	NA	NA	NG	NG	NA	NG	NA	+	NG	NA	NA	NA	NA	NG	+	NA	NA	NA	NA	+	NG	NG
3.4 Puncture location	L_{3-4}	NA	NA	NG	NG	NA	NG	NA	NG	NG	NA	NA	NA	NA	NG	+	NA	NA	NA	NA	NG	NG	NG
3.5 Recommend level of block	T_{6-4}	NA	NA	NG	NG	NA	NG	NA	NG	NG	NA	NA	NA	NA	NG	+	NA	NA	NA	NA	NG	NG	NG
3.6 Analgesic dose	Provided	NA	NA	NG	NG	NA	NG	NA	NG	NG	NA	NA	NA	NA	NG	+	NA	NA	NA	NA	NG	NG	+
-Bupivacaine	5-15 mg	NA	NA	NG	NG	NA	NG	NA	NG	NG	NA	NA	NA	NA	NG	+	NA	NA	NA	NA	NG	NG	+
-Ropivacaine	10-20 mg	NA	NA	NG	NG	NA	NG	NA	NG	NG	NA	NA	NA	NA	NG	+	NA	NA	NA	NA	NG	NG	NG
3.7 Spinal needles	Provided	NA	NA	+	NG	NA	NG	NA	NG	NG	NA	NA	NA	NA	NG	+	NA	NA	NA	NA	+	NG	NG
-Cutting-bevel	Disagree	NA	NA	+	NG	NA	NG	NA	NG	NG	NA	NA	NA	NA	NG	+	NA	NA	NA	NA	+	NG	NG
-Pencil-point	Agree	NA	NA	+	NG	NA	NG	NA	NG	NG	NA	NA	NA	NA	NG	+	NA	NA	NA	NA	+	NG	NG

For peer review only

**4. Combined spinal–epidural analgesia**	Provided	+	NA	+	+	NA	+/-	NA	+	+	NA	NA	NA	NA	+	+	NA	NA	NA	NA	+/-	NG	+
4.1 Puncture location	Provided	NG	NA	NG	NG	NA	NG	NA	NG	NG	NA	NA	NA	NA	NG	+	NA	NA	NA	NA	+/-	NG	NG
-Single space technique	L_{3-4}	NG	NA	NG	NG	NA	NG	NA	NG	NG	NA	NA	NA	NA	NG	+	NA	NA	NA	NA	+/-	NG	NG
-Double space technique	T_{12}-L_2/L_{3-5}	NG	NA	NG	NG	NA	NG	NA	NG	NG	NA	NA	NA	NA	NG	+	NA	NA	NA	NA	+/-	NG	NG
4.2 Anesthetics (consistent with spinal or epidural)	Agree	NG	NA	NG	NG	NA	NG	NA	+	NG	NA	NA	NA	NA	NG	+	NA	NA	NA	NA	+/-	NG	+/-
**5. General anesthesia**	Provided	+	NA	+	NA	NA	NA	NA	+	NA	NA	NA	NA	NA	+	+	NA	NA	+	NA	+	+/-	NA
5.1 General anesthesia selected condition	Provided	NG	NA	+	NA	NA	NA	NA	+	NA	NA	NA	NA	NA	NG	NG	NA	NA	NG	NA	+	NG	NA
-Profound fetal bradycardia	Agree	NG	NA	+	NA	NA	NA	NA	NG	NA	NA	NA	NA	NA	NG	NG	NA	NA	NG	NA	NG	NG	NA
-Ruptured uterus	Agree	NG	NA	+	NA	NA	NA	NA	NG	NA	NA	NA	NA	NA	NG	NG	NA	NA	NG	NA	NG	NG	NA
-Severe Hemorrhage	Agree	NG	NA	+	NA	NA	NA	NA	NG	NA	NA	NA	NA	NA	NG	NG	NA	NA	NG	NA	NG	NG	NA
-Severe placental abruption	Agree	NG	NA	+	NA	NA	NA	NA	NG	NA	NA	NA	NA	NA	NG	NG	NA	NA	NG	NA	NG	NG	NA
-Umbilical cord prolapse	Agree	NG	NA	+	NA	NA	NA	NA	NG	NA	NA	NA	NA	NA	NG	NG	NA	NA	NG	NA	NG	NG	NA
-Preterm footling breech	Agree	NG	NA	+	NA	NA	NA	NA	NG	NA	NA	NA	NA	NA	NG	NG	NA	NA	NG	NA	NG	NG	NA
-Inability to place neuraxial anesthesia	Agree	NG	NA	NG	NA	NA	NA	NA	+	NA	NA	NA	NA	NA	NG	NG	NA	NA	NG	NA	+	NG	NA
5.2 Intravenous anesthetics recommendation	Provided	NG	NA	NG	NA	NA	NA	NA	+	NA	NA	NA	NA	NA	NG	+	NA	NA	NG	NA	NG	NG	NA

For peer review only - http://bmjopen.bmj.com/site/about/guidelines.xhtml

-Thiopental sodium	4-5 mg.kg^{-1}	NG	NA	NG	NA	NA	NA	NA	NG	NA	NA	NA	NA	NA	NG	+	NA	NA	NG	NA	NG	NG	NA
-Propofol	1.5-2.5 mg.kg^{-1}	NG	NA	NG	NA	NA	NA	NA	+	NA	NA	NA	NA	NA	NG	+	NA	NA	NG	NA	NG	NG	NA
-Etomidate	0.2-0.3 mg.kg^{-1}	NG	NA	NG	NA	NA	NA	NA	NG	NA	NA	NA	NA	NA	NG	+	NA	NA	NG	NA	NG	NG	NA
-Ketamine	0.5~1 mg.kg^{-1}	NG	NA	NG	NA	NA	NA	NA	+	NA	NA	NA	NA	NA	NG	+	NA	NA	NG	NA	NG	NG	NA
5.3 Opioids analgesic recommendation	Provided	NG	NA	NG	NA	NA	NA	NA	NG	NA	NA	NA	NA	NA	NG	+	NA	NA	NG	NA	NG	NG	NA
-Fentanyl	2-5 μg.kg^{-1}	NG	NA	NG	NA	NA	NA	NA	NG	NA	NA	NA	NA	NA	NG	+	NA	NA	NG	NA	NG	NG	NA
-Sufentanyl	0.2~0.5 μg.kg^{-1}	NG	NA	NG	NA	NA	NA	NA	NG	NA	NA	NA	NA	NA	NG	+	NA	NA	NG	NA	NG	NG	NA
-Remifentanyl	0.5-1 μg.kg^{-1} for IV	NG	NA	NG	NA	NA	NA	NA	NG	NA	NA	NA	NA	NA	NG	+	NA	NG	NG	NA	NG	NG	NA
-Remifentanyl	4 ng.kg^{-1} for target	NG	NA	NG	NA	NA	NA	NA	NG	NA	NA	NA	NA	NA	NG	+	NA	NG	NG	NA	NG	NG	NA
5.4 Muscle relaxants recommendation	Provided	NG	NA	NG	NA	NA	NA	NA	+	NA	NA	NA	NA	NA	NG	+	NA	NG	NG	NA	NG	NG	NA
-Succinylcholine	1-1.5 mg.kg^{-1}	NG	NA	NG	NA	NA	NA	NA	NG	NA	NA	NA	NA	NA	NG	+	NA	NG	NG	NA	NG	NG	NA
-Rocuronium	0.6-1.2 mg.kg^{-1}	NG	NA	NG	NA	NA	NA	NA	NG	NA	NA	NA	NA	NA	NG	+	NA	NG	NG	NA	NG	NG	NA
5.5 Induction-delivery interval	within 10 min	NG	NA	NG	NA	NA	NA	NA	NG	NA	NA	NA	NA	NA	NG	+	NA	NG	NG	NA	NG	NG	NA

+: consistent the reference with content;

+/-: partial1y consistent the reference with content;

-: inconsistent the reference with content;

NA: not applicable;

For peer review only - http://bmjopen.bmj.com/site/about/guidelines.xhtml

NG: not given.

Supplementary Table 13. Summary of recommendations for the monitoring and management of obstetric and anesthetic or intra- or postoperative pain management by included guidance document. []

		OG-OG-CMA_2014	OG-A-CMA_2016	ASA_2016	AAGBI_2017	ERAS_2018 (Part 1)	ERAS_2018 (Part 2)	ERAS_2018 (Part 3)	ACOG_2019	SOGC_2019	SOGC_2022	SOAP_2019	AA and OAA_2020	PROSPECT_2020	CHBSA_2020	CAA_2020	CAA_2020	SOAP_2020	NICE_2021	OAA_2022	ANNA_2023	RCoA_2023	French_2021
**Recommendations**	**Reference contents**																						
**Neurological Monitoring Associated with Obstetric Neuraxial Block**																							
**1. Screening method (During the recovery phase) - Straight-leg raising**	Agree	NA	NA	NA	NA	NA	NA	NA	NA	NA	NA	NA	+	NA	NA	NA	NA	NA	NA	NA	NA	NA	NA
**2. Alerts incidences-unable to straight-leg raise at 4 h from the last dose**	Agree	NA	NA	NA	NA	NA	NA	NA	NA	NA	NA	NA	+	NA	NA	NA	NA	NA	NA	NA	NA	NA	NA

For peer review only

**Respiratory Depression Monitoring with Neuraxial Block With Opioids**																							
**1. Frequency and modality of respiratory monitoring should be based on:**	Provided	+	NA	NA	NA	NA	NA	NA	NA	NA	NA	+	NA	NA	NA	NA	NA	NA	+/-	NA	+/-	NA	NA
1.1 Low risk	Provided	NG	NA	NA	NA	NA	NA	NA	NA	NA	NA	+	NA	NA	NA	NA	NA	NA	+/-	NA	NG	NA	NA
-Neuraxial morphine dose (intrathecal ≤ 0.15 mg or epidural ≤ 3 mg)	Agree	NG	NA	NA	NA	NA	NA	NA	NA	NA	NA	+	NA	NA	NA	NA	NA	NA	NG	NA	NG	NA	NA
1.2 High risk	Provided	NG	NA	NA	NA	NA	NA	NA	NA	NA	NA	+	NA	NA	NA	NA	NA	NA	+/-	NA	NG	NA	NA
Cardiopulmonary/ neurological comorbidity	Agree	NG	NA	NA	NA	NA	NA	NA	NA	NA	NA	+	NA	NA	NA	NA	NA	NA	NG	NA	NG	NA	NA
-Obesity (body mass index ≥ 40 kg/m^2)	Agree	NG	NA	NA	NA	NA	NA	NA	NA	NA	NA	+	NA	NA	NA	NA	NA	NA	NG	NA	NG	NA	NA
-Known or suspected obstructive sleep apnea	Agree	NG	NA	NA	NA	NA	NA	NA	NA	NA	NA	+	NA	NA	NA	NA	NA	NA	NG	NA	NG	NA	NA

For peer review only - http://bmjopen.bmj.com/site/about/guidelines.xhtml

-Chronic opioid use or abuse	Agree	NG	NA	NA	NA	NA	NA	NA	NA	NA	NA	+	NA	NA	NA	NA	NA	NA	NG	NA	NG	NA	NA
-Hypertension	Agree	NG	NA	NA	NA	NA	NA	NA	NA	NA	NA	+	NA	NA	NA	NA	NA	NA	NG	NA	NG	NA	NA
-Magnesium administration	Agree	NG	NA	NA	NA	NA	NA	NA	NA	NA	NA	+	NA	NA	NA	NA	NA	NA	NG	NA	NG	NA	NA
-Neuraxial morphine dose (intrathecal > 0.15 mg or epidural > 3 mg)	Agree	NG	NA	NA	NA	NA	NA	NA	NA	NA	NA	+	NA	NA	NA	NA	NA	NA	NG	NA	NG	NA	NA
1.3 Respiratory monitoring frequency and duration	Provided	NG	NA	NA	NA	NA	NA	NA	NA	NA	NA	+	NA	NA	NA	NA	NA	NA	+/-	NA	NG	NA	NA
-Low risk	Provided	NG	NA	NA	NA	NA	NA	NA	NA	NA	NA	+	NA	NA	NA	NA	NA	NA	+/-	NA	NG	NA	NA
--Routine postoperative vital sign monitoring	Agree	NG	NA	NA	NA	NA	NA	NA	NA	NA	NA	+	NA	NA	NA	NA	NA	NA	+	NA	NG	NA	NA
-- q2h clinical assessment for 12 h	Agree	+/-	NA	NA	NA	NA	NA	NA	NA	NA	NA	+	NA	NA	NA	NA	NA	NA	+	NA	NG	NA	NA
-High risk	Provided	NG	NA	NA	NA	NA	NA	NA	NA		NA	+		NA	NA	NA	NA	NA	+	NA	NG	NA	NA
-- q1h clinical assessment for first 12 h	Agree	NG	NA	NA	NA	NA	NA	NA	NA	NA	NA	+	NA	NA	NA	NA	NA	NA	half hour for 2 hours	NA	NG	NA	NA
-- q2h clinical assessment for first 12-24 h	Agree	+/-	NA	NA	NA	NA	NA	NA	NA	NA	NA	+	NA	NA	NA	NA	NA	NA	NG	NA	NG	NA	NA

For peer review only - http://bmjopen.bmj.com/site/about/guidelines.xhtml

--Consider additional monitoring modalities (e.g., pulse oximetry, capnography)	Agree	NG	NA	NA	NA	NA	NA	NA	NA	NA	NA	+	NA	NA	NA	NA	NA	NA	+/-	NA	NG	NA	NA
**Management of Intra- or Postoperative Pain**																							
**1. Implement strategies to minimize systemic opioid utilization**	Provided	NA	NA	NA	NA	NA	NA	+	+	NA	NA	+	NA	+	+	NA	NA	+	+	+	+/-	NA	NA
**2. Neuraxial adjuvant drugs**	Provided	NA	NA	NA	NA	NA	NA	+	+	NA	NA	+	NA	+	+	NA	NA	+	+	NG	+	NA	NA
-Spinal anesthesia	Provided	NA	NA	NA	NA	NA	NA	NG	+	NA	NA	+	NA	+	+	NA	NA	+	+	NG	+	NA	NA
--Morphine	50-150 µg	NA	NA	NA	NA	NA	NA	NG	+	NA	NA	50-150 µg	NA	+	+	NA	NA	50-150 µg	NG	NG	150-500µg	NA	NA
-Hydromorphone	75-150 µg	NA	NA	NA	NA	NA	NA	NG	NG	NA	NA	NG	NA	NG	NG	NA	NA	NG	NG	NG	+	NA	NA
-Epidural or combined spinal-epidural technique.	Provided	NA	NA	NA	NA	NA	NA	NG	+	NA	NA	+	NA	+	NG	NA	NA	+	+	NG	+	NA	NA
--Morphine	1-3 mg	NA	NA	NA	NA	NA	NA	NG	+	NA	NA	1-3 mg	NA	+	NG	NA	NA	1~3 mg	NG	NG	1-3mg	NA	NA

For peer review only - http://bmjopen.bmj.com/site/about/guidelines.xhtml

--Diamorphine	2-3 mg	NA	NA	NA	NA	NA	NA	NG	NG	NA	NA	NG	NA	+	NG	NA	NA	NG	2.5-5 mg	NG	NG	NA	NA
**3. Others analgesic (after delivery)**	Provided	NA	NA	NA	NA	NA	NA	+	+	NA	NA	+	NA	+	+	NA	NA	+	+	NG	+	NA	NA
-Non-steroidal anti-inflammatory drug (e.g., paracetamol)	Agree	NA	NA	NA	NA	NA	NA	+	+	NA	NA	+	NA	+	+	NA	NA	+	+	NG	+	NA	NA
-A single dose of intravenous dexamethasone	Agree	NA	NA	NA	NA	NA	NA	NG	NG	NA	NA	NG	NA	+	NG	NA	NA	NG	NG	NG	+	NA	NA
**4. Local anesthetic infiltration**	Agree	NA	NA	NA	NA	NA	NA	+	+	NA	NA	NG	NA	+	+	NA	NA	+	NG	NG	+	NA	NA
**5. Continuous wound local anesthetic infusion**	Agree	NA	NA	NA	NA	NA	NA	NG	+	NA	NA	NG	NA	+	+	NA	NA	NG	NG	NG	NG	NA	NA
**6. Fascial plane blocks (e.g., transversus abdominis plane, erector spinae plane)**	Agree	NA	NA	NA	NA	NA	NA	+	+	NA	NA	NG	NA	+	+	NA	NA	+	NG	NG	NG	NA	NA
**7. Patient-controlled intravenous analgesia**	Agree	NA	NA	NA	NA	NA	NA	NG	+	NA	NA	NG	NA	+	+	NA	NA	NG	+	NG	NG	NA	NA

+: consistent the reference with content;

+/-: partial1y consistent the reference with content;

For peer review only - http://bmjopen.bmj.com/site/about/guidelines.xhtml

For peer review only

-: inconsistent the reference with content;

NA: not applicable;

NG: not given.

For peer review only - http://bmjopen.bmj.com/site/about/guidelines.xhtml

Supplementary Table 14. Summary of recommendations for the adverse events by included guidance document.

Recommendations	Reference contents	OG-OG-CMA_2014	OG-A-CMA_2016	ASA_2016	AAGBI_2017	ERAS_2018 (Part 1)	ERAS_2018 (Part 2)	ERAS_2018 (Part 3)	ACOG_2019	SOGC_2019	SOGC_2022	SOAP_2019	AA and OAA_2020	PROSPECT_2020	CHBSA_2020	CAA_2020	CAA_2020	SOAP_2020	NICE_2021	OAA_2022	ANNA_2023	RCoA_2023	French_2021
**1. Hypotension prevention strategies**	Provided	NA	NA	+	+	NA	+/-	+	+	+	NA	NA	NA	NA	+	+	+	+	+	NA	+/-	NA	NA
1.1 Blood pressure target of systolic arterial pressure	≥90% of baseline and avoid decrease to < 80% baseline	NA	NA	NG	+	NA	NG	NG	NG	NG	NA	NA	NA	NA	NG	NG	NG	NG	+	NA	NG	NA	NA
1.2 Position (uterine left-leaning)	Yes	NA	NA	+	+	NA	NG	NG	NG	NG	NA	NA	NA	NA	+	+	+	NG	+	NA	+	NA	NA
1.3 Volume expansion (crystals or colloids)	500-1000 ml	NA	NA	+	+	NA	+	+	+	+	NA	NA	NA	NA	+	+	+	NG	+	NA	+	NA	NA
1.4 Vasoactive drugs	Yes	NA	NA	+	+	NA	NG	+	+	NG	NA	NA	NA	NA	+	+	+	+	+	NA	+	NA	NA
-Pump infusion is recommended	Provided	NA	NA	+	+	NA	NG	NG	NG	NG	NA	NA	NA	NA	NG	NG	NG	NG	NG	NA	NG	NA	NA
-Type of drugs	Provided	NA	NA	+	+	NA	NG	+	+	NG	NA	NA	NA	NA	+	+	+	+	NG	NA	+/-	NA	NA
--Phenylephrine	20-50 µg Or	NA	NA	+	20~50 µg or	NA	NG	+	+	NG	NA	NA	NA	NA	+	+	+	+	NG	NA	+	NA	NA

For peer review only - http://bmjopen.bmj.com/site/about/guidelines.xhtml

	0.5 μg . kg^{-1}. min^{-1}				0.25. kg^{-1}. min^{-1}																		
--Metaraminol	1-2 mg or 4 μg.kg^{-1}. min^{-1}	NA	NA	NG	0.5mg.ml^{-1}	NA	NG	NG	NG	NG	NA	NA	NA	NA	NG	NG	NG	NG	NG	NA	NG	NA	NA
--Noradrenalin	4-6 μg or 0.08 μg kg^{-1}. min^{-1}	NA	NA	NG	NG	NA	NG	NG	NG	NG	NA	NA	NA	NA	NG	+	NG	NG	NG	NA	NG	NA	NA
--Ephedrine	2-15 mg for IV	NA	NA	NG	2-4mg	NA	NG	+	+	NG	NA	NA	NA	NA	+	+	+	NG	NG	NA	NG	NA	NA
1.5 Prophylactic vasopressor infusion should not be recommended in pre-eclampsia	Agree	NA	NA	NG	+	NA	NG	NG	NG	NG	NA	NA	NA	NA	NG	+	NG	NG	NG	NA	NG	NA	NA
**2 Inadequate neuraxial anesthesia**	Provided	NA	NA	NA	NA	NA	NA	NA	+	NA	NA	NA	NA	NA	NA	NA	+	NA	NA	+	+	+	+
2.1 Block assessment	Provided	NA	NA	NA	NA	NA	NA	NA	NG	NA	NA	NA	NA	NA	NA	NA	NG	NA	NA	+	+	NG	+
-Testing sensory block after the evidence of motor block	Agree	NA	NA	NA	NA	NA	NA	NA	NG	NA	NA	NA	NA	NA	NA	NA	NG	NA	NA	+	+	NG	NG
-Check the block at relatively fixed time intervals	Agree	NA	NA	NA	NA	NA	NA	NA	NG	NA	NA	NA	NA	NA	NA	NA	NG	NA	NA	+	NG	NG	NG
-Perform the first check early enough to allow positional changes	Agree	NA	NA	NA	NA	NA	NA	NA	NG	NA	NA	NA	NA	NA	NA	NA	NG	NA	NA	+	NG	NG	NG

For peer review only - http://bmjopen.bmj.com/site/about/guidelines.xhtml

2.2 Rescue for inadequate neuraxial block (change anesthesia method)	Provided	NA	NA	NA	NA	NA	NA	NA	+	NA	NA	NA	NA	NA	NA	NA	NG	NA	NA	+	+	NG	+
-A second neuraxial technique (in the case of spinal anesthesia)	Agree	NA	NA	NA	NA	NA	NA	NA	+	NA	NA	NA	NA	NA	NA	NA	+	NA	NA	+	+	NG	+
-Extending the neuraxial technique (in the case of combined spinal–epidural)	Agree	NA	NA	NA	NA	NA	NA	NA	+	NA	NA	NA	NA	NA	NA	NA	NG	NA	NA	+	+	NG	NG
-Above options are not possible, general anesthesia should be recommended	Agree	NA	NA	NA	NA	NA	NA	NA	+	NA	NA	NA	NA	NA	NA	NA	NG	NA	NA	+	+	NG	+
2.3 Rescue for inadequate neuraxial block (change anesthetic)	Provided	NA	NA	NA	NA	NA	NA	NA	+	NA	NA	NA	NA	NA	NA	NA	NG	NA	NA	+	+	NG	+
-Nitrous oxide and oxygen alone	Disagree	NA	NA	NA	NA	NA	NA	NA	NG	NA	NA	NA	NA	NA	NA	NA	NG	NA	NA	+	NG	NG	NG
-Continue with neuraxial block, consider repeated boluses of fast acting opioids	Agree	NA	NA	NA	NA	NA	NA	NA	NG	NA	NA	NA	NA	NA	NA	NA	NG	NA	NA	+	NG	NG	NG
--Fentanyl	25-50 µg	NA	NA	NA	NA	NA	NA	NA	NG	NA	NA	NA	NA	NA	NA	NA	NG	NA	NA	+	NG	NG	NG
--Alfentanil	250-500 µg	NA	NA	NA	NA	NA	NA	NA	NG	NA	NA	NA	NA	NA	NA	NA	NG	NA	NA	+	NG	NG	NG
--Ketamine	10 mg boluses	NA	NA	NA	NA	NA	NA	NA	NG	NA	NA	NA	NA	NA	NA	NA	NG	NA	NA	+	10 mg or	NG	

For peer review only - http://bmjopen.bmj.com/site/about/guidelines.xhtml

																					0.15 mg $.kg^{-1}$		+/-
-Do not treat pain with anxiolytics	Agree	NA	NA	NA	NA	NA	NA	NA	NG	NA	NA	NA	NA	NA	NA	NA	NG	NA	NA	+	NG	NG	NG
-Above options are not possible, general anesthesia should be recommended	Agree	NA	NA	NA	NA	NA	NA	NA	+	NA	NA	NA	NA	NA	NA	NA	NG	NA	NA	+	+	NG	+
**3. Post dural puncture headache (PDPH)**	Provided	NA	NA	NA	NA	NA	NA	NA	+	NA	NA	NA	NA	NA	NA	NA	NA	NA	NA	NA	+	+/-	NA
3.1 Measures be used to prevent PDPH	Provided	NA	NA	NA	NA	NA	NA	NA	NG	NA	NA	NA	NA	NA	NA	NA	NA	NA	NA	NA	+/-	NG	NA
-Noncutting spinal needles are associated with decreased PDPH risk	Agree	NA	NA	NA	NA	NA	NA	NA	NG	NA	NA	NA	NA	NA	NA	NA	NA	NA	NA	NA	+	NG	NA
-An intrathecal catheter may be considered to provide anesthesia or analgesia	Agree	NA	NA	NA	NA	NA	NA	NA	NG	NA	NA	NA	NA	NA	NA	NA	NA	NA	NA	NA	+	NG	NA
-A prophylactic epidural blood patch is not recommended as routine	Agree	NA	NA	NA	NA	NA	NA	NA	NG	NA	NA	NA	NA	NA	NA	NA	NA	NA	NA	NA	NG	NG	NA
-Bed rest is not routinely recommended	Agree	NA	NA	NA	NA	NA	NA	NA	NG	NA	NA	NA	NA	NA	NA	NA	NA	NA	NA	NA	NG	NG	NA

For peer review only - http://bmjopen.bmj.com/site/about/guidelines.xhtml

as prophylaxis against PDPH																							
-Routine injection of substance to prevent PDPH is not recommended	Agree	NA	NA	NA	NA	NA	NA	NA	NG	NA	NA	NA	NA	NA	NA	NA	NA	NA	NA	NA	NG	NG	NA
-Routine systemic drug for PDPH prophylaxis is not recommended	Agree	NA	NA	NA	NA	NA	NA	NA	NG	NA	NA	NA	NA	NA	NA	NA	NA	NA	NA	NA	NG	NG	NA
3.2 Measures be used to treat PDPH	Provided	NA	NA	NA	NA	NA	NA	NA	NG	NA	NA	NA	NA	NA	NA	NA	NA	NA	NA	NA	+/-	NG	NA
-Epidural blood patch	Agree	NA	NA	NA	NA	NA	NA	NA	+	NA	NA	NA	NA	NA	NA	NA	NA	NA	NA	NA	+	NG	NA
-The routine use of bed rest to treat PDPH	Disagree	NA	NA	NA	NA	NA	NA	NA	NG	NA	NA	NA	NA	NA	NA	NA	NA	NA	NA	NA	NG	NG	NA
-Adequate hydration should be maintained with oral fluids or intravenous	Agree	NA	NA	NA	NA	NA	NA	NA	NG	NA	NA	NA	NA	NA	NA	NA	NA	NA	NA	NA	+	NG	NA
-Routine use of abdominal binders or aromatherapy to treat PDPH	Disagree	NA	NA	NA	NA	NA	NA	NA	NG	NA	NA	NA	NA	NA	NA	NA	NA	NA	NA	NA	NG	NG	NA
-Regular multimodal analgesia	Agree	NA	NA	NA	NA	NA	NA	NA	NG	NA	NA	NA	NA	NA	NA	NA	NA	NA	NA	NA	+/-	NG	NA
-Short-term use of opioids if regular	Agree	NA	NA	NA	NA	NA	NA	NA	NG	NA	NA	NA	NA	NA	NA	NA	NA	NA	NA	NA	NG	NG	NA

For peer review only - http://bmjopen.bmj.com/site/about/guidelines.xhtml

multimodal analgesia is ineffective																							
-Long-term opioid use is not recommended in the treatment of PDPH	Agree	NA	NA	NA	NA	NA	NA	NA	NG	NA	NA	NA	NA	NA	NA	NA	NA	NA	NA	NA	NG	NG	NA
-Caffeine offered in the first 24h with a maximum dose of 900 mg per day	Agree	NA	NA	NA	NA	NA	NA	NA	NG	NA	NA	NA	NA	NA	NA	NA	NA	NA	NA	NA	+/-	NG	NA
-Caffeine offered 200-300 mg if breastfeeding	Agree	NA	NA	NA	NA	NA	NA	NA	NG	NA	NA	NA	NA	NA	NA	NA	NA	NA	NA	NA	NG	NG	NA
-Routine use of hydrocortisone, adrenocorticotropic hormone or cosyntropin	Disagree	NA	NA	NA	NA	NA	NA	NA	NG	NA	NA	NA	NA	NA	NA	NA	NA	NA	NA	NA	+/-	NG	NA
-Routine use of theophylline, neostigmine or atropine, piritramide, and gabapentin	Disagree	NA	NA	NA	NA	NA	NA	NA	NG	NA	NA	NA	NA	NA	NA	NA	NA	NA	NA	NA	NG	NG	NA
-Routine use of acupuncture to treat PDPH	Disagree	NA	NA	NA	NA	NA	NA	NA	NG	NA	NA	NA	NA	NA	NA	NA	NA	NA	NA	NA	NG	NG	NA
-Routine use of sphenopalatine ganglion blocks to treat PDPH	Disagree	NA	NA	NA	NA	NA	NA	NA	NG	NA	NA	NA	NA	NA	NA	NA	NA	NA	NA	NA	+	NG	NA

For peer review only - http://bmjopen.bmj.com/site/about/guidelines.xhtml

-Greater occipital nerve blocks be offered to patients with PDPH	Agree	NA	NA	NA	NA	NA	NA	NA	NG	NA	NA	NA	NA	NA	NA	NA	NA	NA	NA	NA	NG	NG	NA
-The use of spinal and epidural morphine to treat PDPH	Disagree	NA	NA	NA	NA	NA	NA	NA	NG	NA	NA	NA	NA	NA	NA	NA	NA	NA	NA	NA	NG	NG	NA
-Routine use of epidural dextran, gelatine, or hydroxyethyl starch to treat PDPH	Disagree	NA	NA	NA	NA	NA	NA	NA	NG	NA	NA	NA	NA	NA	NA	NA	NA	NA	NA	NA	NG	NG	NA
-Routine use of fibrin glue to treat PDPH	Disagree	NA	NA	NA	NA	NA	NA	NA	NG	NA	NA	NA	NA	NA	NA	NA	NA	NA	NA	NA	NG	NG	NA
-Fibrin glue should be reserved for management of PDPH refractory to EBP	Agree	NA	NA	NA	NA	NA	NA	NA	NG	NA	NA	NA	NA	NA	NA	NA	NA	NA	NA	NA	NG	NG	NA
-Fibrin glue be used when autologous blood injection is contraindicated	Agree	NA	NA	NA	NA	NA	NA	NA	NG	NA	NA	NA	NA	NA	NA	NA	NA	NA	NA	NA	NG	NG	NA

+: consistent the reference with content;

+/-: partial1y consistent the reference with content;

-: inconsistent the reference with content;

NA: not applicable;

NG: not given.

For peer review only - http://bmjopen.bmj.com/site/about/guidelines.xhtml

Supplementary Table 15. Summary of recommendations for the monitoring and management of obstetric and anesthetic emergencies by included guidance document.

Recommendations	Reference contents	OG-OG-CMA_2014	OG-A-CMA_2016	ASA_2016	AAGBI_2017	ERAS_2018 (Part 1)	ERAS_2018 (Part 2)	ERAS_2018 (Part 3)	ACOG_2019	SOGC_2019	SOGC_2022	SOAP_2019	AA and OAA_2020	PROSPECT_2020	CHBSA_2020	CAA_2020	CAA_2020	SOAP_2020	NICE_2021	OAA_2022	ANNA_2023	RCoA_2023	French_2021
**1. Resources for management of Hemorrhagic emergencies**	Provided	NA	NA	+	NA	NA	NA	NA	NA	NA	NA	NA	NA	NA	NA	+/-	NA	NA	NA	NA	+	+/-	NA
-Large-bore IV catheters	Agree	NA	NA	+	NA	NA	NA	NA	NA	NA	NA	NA	NA	NA	NA	+	NA	NA	NA	NA	+	NG	NA
-Fluid warmer	Agree	NA	NA	+	NA	NA	NA	NA	NA	NA	NA	NA	NA	NA	NA	NG	NA	NA	NA	NA	NG	NG	NA
-Forced-air body warmer	Agree	NA	NA	+	NA	NA	NA	NA	NA	NA	NA	NA	NA	NA	NA	NG	NA	NA	NA	NA	NG	NG	NA
-Availability of blood bank resources	Agree	NA	NA	+	NA	NA	NA	NA	NA	NA	NA	NA	NA	NA	NA	NG	NA	NA	NA	NA	+	NG	NA
-Massive transfusion protocol (type-specific, or O-negative blood, or salvage)	Agree	NA	NA	+	NA	NA	NA	NA	NA	NA	NA	NA	NA	NA	NA	NG	NA	NA	NA	NA	+	NG	NA
-Equipment for infusing IV fluids and blood products rapidly	Agree	NA	NA	+	NA	NA	NA	NA	NA	NA	NA	NA	NA	NA	NA	NG	NA	NA	NA	NA	NG	NG	NA

For peer review only - http://bmjopen.bmj.com/site/about/guidelines.xhtml

For peer review only

**2. Equipment for management of airway emergencies**	Provided	NA	NA	+	NA	NA	NA	NA	+	+/-	NA	NA	NA	NA	NA	+	NA	NA	+	NA	+/-	+/-	NA
2.1 Laryngoscope and assorted blades	Agree	NA	NA	+	NA	NA	NA	NA	+	NG	NA	NA	NA	NA	NA	NG	NA	NA	NG	NA	+	NG	NA
2.2 Endotracheal tubes, with stylets	Agree	NA	NA	+	NA	NA	NA	NA	+	NG	NA	NA	NA	NA	NA	NG	NA	NA	NG	NA	+	NG	NA
2.3 Oxygen source	Agree	NA	NA	+	NA	NA	NA	NA	NG	NG	NA	NA	NA	NA	NA	NG	NA	NA	+	NA	+	NG	NA
2.4 Suction source with tubing and tonsil suction tip	Agree	NA	NA	+	NA	NA	NA	NA	NG	NG	NA	NA	NA	NA	NA	NG	NA	NA	+	NA	+	NG	NA
2.5 Self-inflating bag and mask for positive-pressure ventilation	Agree	NA	NA	+	NA	NA	NA	NA	NG	NG	NA	NA	NA	NA	NA	NG	NA	NA	NG	NA	+	NG	NA
2.6 Medications for blood pressure support, muscle relaxation, and hypnosis	Agree	NA	NA	+	NA	NA	NA	NA	NG	NG	NA	NA	NA	NA	NA	NG	NA	NA	NA	NA	NG	NG	NA
2.7 Tracheal intubation has failed protocol	Agree	NA	NA	+	NA	NA	NA	NA	NG	NG	NA	NA	NA	NA	NA	+	NA	NA	+	NA	+	NG	NA
**3. Cardiopulmonary Resuscitation**	Provided	NA	NA	+	NA	NA	NA	NA	NA	NA	NA	NA	NA	NA	NA	NA	NA	NA	NA	NA	+/-	+/-	NA
3.1 Basic and advanced life-support equipment	Agree	NA	NA	+	NA	NA	NA	NA	NA	NA	NA	NA	NA	NA	NA	NA	NA	NA	NA	NA	+	NG	NA

For peer review only

3.2 Cardiac arrest occurs	Provided	NA	NA	+	NA	NA	NA	NA	NA	NA	NA	NA	NA	NA	NA	NA	NA	NA	NA	NA	+	NG	NA
-Uterine displacement (usually left displacement) should be maintained	Agree	NA	NA	+	NA	NA	NA	NA	NA	NA	NA	NA	NA	NA	NA	NA	NA	NA	NA	NA	+	NG	NA
-If circulation is not restored within 4 min, cesarean should be performed	Agree	NA	NA	+	NA	NA	NA	NA	NA	NA	NA	NA	NA	NA	NA	NA	NA	NA	NA	NA	4-5 min	NG	NA
**4. Emergency cesarean delivery**	Provided	NA	NA	+	NA	NA	NA	NA	+	NA	NA	NA	NA	NA	NA	NA	NA	NA	+	NA	+/-	+/-	+
4.1 Response times limited	30 or 75 minutes	NA	NA	NG	NA	NA	NA	NA	NG	NA	NA	NA	NA	NA	NA	NA	NA	NA	+	NA	NG	NG	15-30 minutes
4.2 Anesthesia methods recommendation	Provided	NA	NA	+/-	NA	NA	NA	NA	+	NA	NA	NA	NA	NA	NA	NA	NA	NA	+/-	NA	NG	NG	+
-Spinal	Agree	NA	NA	+/-	NA	NA	NA	NA	+	NA	NA	NA	NA	NA	NA	NA	NA	NA	NG	NA	NG	NG	+
-Epidural	Agree	NA	NA	+/-	NA	NA	NA	NA	+	NA	NA	NA	NA	NA	NA	NA	NA	NA	NG	NA	NG	NG	+
-Combined spinal–epidural analgesia	Agree	NA	NA	+/-	NA	NA	NA	NA	+	NA	NA	NA	NA	NA	NA	NA	NA	NA	NG	NA	NG	NG	+
-General anesthesia	Agree	NA	NA	+/-	NA	NA	NA	NA	+	NA	NA	NA	NA	NA	NA	NA	NA	NA	NG	NA	NG	NG	+

+/-: partial1y consistent the reference with content;
-: inconsistent the reference with content;
NA: not applicable;
NG: not given.

For peer review only - http://bmjopen.bmj.com/site/about/guidelines.xhtml

■ Consistent with the reference content
■ Inconsistent with the reference content
■ Partially consistent with the reference content
■ Not given
■ Not applicable

Recommendations	Reference contents	OG-OG-CMA_2014	OG-A-CMA_2016	ASA_2016	AAGBI_2017	ERAS_2018 (Part 1)	ERAS_2018 (Part 2)	ERAS_2018 (Part 3)	ACOG_2019	SOGC_2019	SOGC_2022	SOAP_2019	AA and OAA_2020	PROSPECT_2020	CHBSA_2020	CAA_2020	CAA_2020	SOAP_2020	NICE_2021	OAA_2022	ANNA_2023	RCoA_2023	French_2021
**Peri-anesthetic Evaluation and Preparation**																							
**1. History and physical examination before providing anesthetic care**	Yes	Consistent	Consistent	Consistent	Not applicable	Not given	Not applicable	Not applicable	Consistent	Not applicable	Not applicable	Not applicable	Not applicable	Not applicable	Consistent	Consistent	Consistent	Not given	Partially consistent	Not applicable	Consistent	Not given	Consistent
1.1 Maternal health and anesthetic history	Agree	Consistent	Consistent	Consistent	Not applicable	Not given	Not applicable	Not applicable	Consistent	Not applicable	Not applicable	Not applicable	Not applicable	Not applicable	Consistent	Consistent	Consistent	Not given	Not given	Not applicable	Partially consistent	Not given	Consistent
1.2 Relevant obstetric history	Agree	Consistent	Consistent	Consistent	Not applicable	Not given	Not applicable	Not applicable	Not given	Not applicable	Not applicable	Not applicable	Not applicable	Not applicable	Not given	Consistent	Consistent	Not given	Not given	Not applicable	Not given	Not given	Not given
1.3 Baseline blood pressure measurement	Agree	Consistent	Consistent	Consistent	Not applicable	Not given	Not applicable	Not applicable	Not given	Not applicable	Not applicable	Not applicable	Not applicable	Not applicable	Consistent	Inconsistent	Consistent	Not given	Not given	Not applicable	Partially consistent	Not given	Not given
1.4 Airway examination	Agree	Not given	Consistent	Consistent	Not applicable	Not given	Not applicable	Not applicable	Consistent	Not applicable	Not applicable	Not applicable	Not applicable	Not applicable	Not given	Consistent	Consistent	Not given	Not given	Not applicable	Consistent	Not given	Not given
1.5 Heart and lung examination	Agree	Not given	Not given	Consistent	Not applicable	Not given	Not applicable	Not applicable	Consistent	Not applicable	Not applicable	Not applicable	Not applicable	Not applicable	Consistent	Consistent	Consistent	Not given	Not given	Not applicable	Consistent	Not given	Not given
1.6 When a neuraxial anesthetic is planned, examine the patient’s back	Agree	Not given	Consistent	Consistent	Not applicable	Not given	Not applicable	Not applicable	Consistent	Not applicable	Not applicable	Not applicable	Not applicable	Not applicable	Not given	Consistent	Consistent	Not given	Not given	Not applicable	Consistent	Not given	Not given
1.7 Consultation between the obstetrician and the anesthesiologist	Agree	Consistent	Not given	Consistent	Not applicable	Consistent	Not applicable	Not applicable	Consistent	Not applicable	Not applicable	Not applicable	Not applicable	Not applicable	Not given	Consistent	Consistent	Not given	Not given	Not applicable	Consistent	Not given	Consistent
**2. Preoperative communication**	Encouraged	Consistent	Not given	Consistent	Not applicable	Consistent	Not applicable	Not applicable	Not given	Not applicable	Not applicable	Not applicable	Not applicable	Not applicable	Consistent	Consistent	Inconsistent	Partially consistent	Consistent	Not applicable	Consistent	Partially consistent	Consistent
**3. Intrapartum platelet count**	Yes	Consistent	Consistent	Consistent	Not applicable	Not applicable	Not applicable	Not applicable	Consistent	Not applicable	Not applicable	Not applicable	Not applicable	Not applicable	Not given	Consistent	Not given	Not given	Consistent	Not applicable	Not given	Not given	Not applicable
-Routine	Agree	Consistent	Consistent	Consistent	Not applicable	Not applicable	Not applicable	Not applicable	Consistent	Not applicable	Not applicable	Not applicable	Not applicable	Not applicable	Not given	Not given	Not given	Not given	Inconsistent	Not applicable	Not given	Not given	Not applicable
-Individualized	Agree	Inconsistent	Inconsistent	Consistent	Not applicable	Not applicable	Not applicable	Not applicable	Consistent	Not applicable	Not applicable	Not applicable	Not applicable	Not applicable	Not given	Not given	Not given	Not given	Consistent	Not applicable	Not given	Not given	Not applicable
**4. Blood type and screen**	Yes	Consistent	Consistent	Consistent	Not applicable	Not applicable	Not applicable	Not applicable	Not given	Not applicable	Not applicable	Not applicable	Not applicable	Not applicable	Not given	Consistent	Not given	Not given	Consistent	Not applicable	Not given	Not given	Not applicable
-Routine	Agree	Consistent	Consistent	Inconsistent	Not applicable	Not applicable	Not applicable	Not applicable	Not given	Not applicable	Not applicable	Not applicable	Not applicable	Not applicable	Not given	Consistent	Not given	Not given	Inconsistent	Not applicable	Not given	Not given	Not applicable
-Individualized	Agree	Inconsistent	Inconsistent	Consistent	Not applicable	Not applicable	Not applicable	Not applicable	Not given	Not applicable	Not applicable	Not applicable	Not applicable	Not applicable	Not given	Not given	Not given	Not given	Consistent	Not applicable	Not given	Not given	Not applicable
**5. Blood cross-match**	Yes	Consistent	Not given	Consistent	Not applicable	Not applicable	Not applicable	Not applicable	Not given	Not applicable	Not applicable	Not applicable	Not applicable	Not applicable	Not given	Not given	Not given	Not given	Consistent	Not applicable	Not given	Not given	Not applicable
-Routine	Agree	Consistent	Not given	Inconsistent	Not applicable	Not applicable	Not applicable	Not applicable	Not given	Not applicable	Not applicable	Not applicable	Not applicable	Not applicable	Not given	Not given	Not given	Not given	Inconsistent	Not applicable	Not given	Not given	Not applicable
-Individualized	Agree	Not given	Not given	Consistent	Not applicable	Not applicable	Not applicable	Not applicable	Not given	Not applicable	Not applicable	Not applicable	Not applicable	Not applicable	Not given	Not given	Not given	Not given	Consistent	Not applicable	Not given	Not given	Not applicable
6. Peri-anesthetic recording of fetal heart rate	Yes	Consistent	Consistent	Consistent	Not applicable	Not applicable	Not applicable	Not applicable	Consistent	Consistent	Consistent	Not applicable	Not applicable	Not applicable	Not given	Consistent	Not given	Not given	Not given	Not applicable	Consistent	Not given	Not applicable
**7. Aspiration prevention**	Provided	Consistent	Not given	Consistent	Not applicable	Consistent	Not applicable	Not applicable	Not applicable	Consistent	Not applicable	Not applicable	Not applicable	Not applicable	Consistent	Partially consistent	Not given	Partially consistent	Partially consistent	Not applicable	Partially consistent	Partially consistent	Not applicable
7.1 Clear liquids	Up to 2 h	Not given	Not given	Consistent	Not applicable	Consistent	Not applicable	Not applicable	Not applicable	Not given	Not applicable	Not applicable	Not applicable	Not applicable	Consistent	Consistent	Not given	Consistent	Not given	Not applicable	Not given	Partially consistent	Not applicable
7.2 Solids	6-8 h	Consistent	Not given	Consistent	Not applicable	Consistent	Not applicable	Not applicable	Not applicable	Not given	Not applicable	Not applicable	Not applicable	Not applicable	Consistent	Consistent	Not given	Consistent	Not given	Not applicable	Not given	Not given	Not applicable
7.3 Antacids (surgical procedures-caesarean delivery or postpartum tubal ligation)	Agree	Not given	Not given	Consistent	Not applicable	Consistent	Not applicable	Not applicable	Not applicable	Not given	Not applicable	Not applicable	Not applicable	Not applicable	Consistent	Consistent	Not given	Not given	Consistent	Not applicable	Not given	Consistent	Not applicable
7.4 H_2-receptor antagonists (surgical procedures)	Agree	Not given	Not given	Consistent	Not applicable	Consistent	Not applicable	Consistent	Not applicable	Not given	Not applicable	Not applicable	Not applicable	Not applicable	Consistent	Consistent	Not given	Not given	Consistent	Not applicable	Not given	Not given	Not applicable
7.5 Metoclopramide (surgical procedures)	Agree	Not given	Not given	Consistent	Not applicable	Not given	Not applicable	Consistent	Not applicable	Not given	Not applicable	Not applicable	Not applicable	Not applicable	Not given	Consistent	Not given	Not given	Not given	Not applicable	Not given	Not given	Not applicable
**8. Preoperative heparin or low-molecular-weight heparin**	Yes	Not given	Consistent	Not given	Not applicable	Not applicable	Not applicable	Not applicable	Consistent	Not applicable	Not applicable	Not applicable	Not applicable	Not applicable	Not applicable	Not given	Partially consistent	Partially consistent	Not given	Not applicable	Partially consistent	Partially consistent	Not applicable
8.1 Prophylactic subcutaneous unfractionated heparin (<5,000 units twice daily)	Provided	Not given	Not given	Not given	Not applicable	Not applicable	Not applicable	Not applicable	Consistent	Not applicable	Not applicable	Not applicable	Not applicable	Not applicable	Not applicable	Not given	Not given	Not given	Not given	Not applicable	Not given	Not given	Not applicable
-Contraindication to neuraxial techniques	No	Not given	Not given	Not given	Not applicable	Not applicable	Not applicable	Not applicable	Consistent	Not applicable	Not applicable	Not applicable	Not applicable	Not applicable	Not applicable	Not given	Not given	Not given	Not given	Not applicable	Not given	Not given	Not applicable
-Catheter placement or removal since last dose	4-6 hours	Not given	Not given	Not given	Not applicable	Not applicable	Not applicable	Not applicable	Consistent	Not applicable	Not applicable	Not applicable	Not applicable	Not applicable	Not applicable	Not given	Not given	Not given	Not given	Not applicable	Not given	Not given	Not applicable
8.2 Intermediate unfractionated heparin (75,000-100,000 units)	Provided	Not given	Not given	Not given	Not applicable	Not applicable	Not applicable	Not applicable	Consistent	Not applicable	Not applicable	Not applicable	Not applicable	Not applicable	Not applicable	Not given	Not given	Not given	Not given	Not applicable	Not given	Not given	Not applicable
-Catheter placement or removal since last dose	> 12 hours	Not given	Not given	Not given	Not applicable	Not applicable	Not applicable	Not applicable	Consistent	Not applicable	Not applicable	Not applicable	Not applicable	Not applicable	Not applicable	Not given	Not given	Not given	Not given	Not applicable	Not given	Not given	Not applicable
8.3 High-dose unfractionated heparin (total daily dose greater than 20,000 units)	Provided	Not given	Not given	Not given	Not applicable	Not applicable	Not applicable	Not applicable	Consistent	Not applicable	Not applicable	Not applicable	Not applicable	Not applicable	Not applicable	Not given	Not given	Not given	Not given	Not applicable	Not given	Not given	Not applicable
-Catheter placement or removal since last dose	> 24 hours	Not given	Not given	Not given	Not applicable	Not applicable	Not applicable	Not applicable	Consistent	Not applicable	Not applicable	Not applicable	Not applicable	Not applicable	Not applicable	Not given	Not given	Not given	Not given	Not applicable	Not given	Not given	Not applicable
-With the activated partial thromboplastin time being within normal range	Yes	Not given	Not given	Not given	Not applicable	Not applicable	Not applicable	Not applicable	Consistent	Not applicable	Not applicable	Not applicable	Not applicable	Not applicable	Not applicable	Not given	Not given	Not given	Not given	Not applicable	Not given	Not given	Not applicable
**Anesthetic Care for labor and Vaginal Delivery**																							
**1. Equipment, facilities, and support personnel**	Agree	Not applicable	Consistent	Consistent	Not applicable	Not applicable	Not applicable	Not applicable	Consistent	Not applicable	Not applicable	Not applicable	Not applicable	Not applicable	Consistent	Not applicable	Consistent	Not applicable	Not applicable	Not applicable	Consistent	Consistent	Not applicable
**2. Timing of neuraxial analgesia and outcome of labor**	Provided	Not applicable	Consistent	Consistent	Not applicable	Not applicable	Not applicable	Not applicable	Not given	Not applicable	Not applicable	Not applicable	Not applicable	Not applicable	Not given	Not applicable	Consistent	Not applicable	Not applicable	Not applicable	Not given	Not given	Not applicable

2.1 Less 5 cm dilation	Agree
2.2 Offer neuraxial analgesia on an individualized basis	Agree
**3. Neuraxial analgesia and trial of labor after prior caesarean delivery**	Agree
**4. Continuous infusion epidural analgesia**	Provided
4.1 Using local anesthetic	Agree
4.2 Add opioid	Agree
4.3 Platelet concentration corresponding to contraindications to puncture	Less than $70*10^9$/L
4.4 Puncture location	L_{2-3} or L_{3-4}
4.5 Analgesic concentrations	Provided
-Bupivacaine	Provided
--Loading dose	0.04-0.125%
--Maintenance dose	0.05-0.125%
-Ropivacaine	Provided
--Loading dose	0.0625-0.15%
--Maintenance dose	0.0625-0.125%
-Levobupivacaine	Provided
--Loading dose	0.04-0.125%
--Maintenance dose	0.05-0.125%
4.6 Opioids concentrations	Provided
-Fentanyl	Provided
--Loading dose	0.5-2 μg/ml
--Maintenance dose	1-2 μg/ml
-Sufentanyl	Provided
--Loading dose	0.2~0.6 μg/ml
--Maintenance dose	0.3~0.6 μg/ml
**5. Combined spinal–epidural analgesia**	Provided
5.1 Using local anesthetic	Agree
5.2 Add opioid	Agree
5.3 Platelet concentration corresponding to contraindications to puncture	Less than $70*10^9$/L
5.4 Puncture location	L_{2-3} or L_{3-4}
5.5 Epidural anesthetics (consistent with 4.5 and 4.6)	Provided
5.6 Spinal anesthetics	Provided
-Protocol 1	Provided
--Single sufentanyl	2.5-7 μg
--Single ropivacaine	2.3-3 mg
--Combined sufentanyl and ropivacaine	2.5 μg / 2.5 mg
-Protocol 2	Provided
--Single fentanyl	15-25 μg
--Single bupivacaine	2.0-2.5 mg
--Combined fentanyl and bupivacaine	2.5 μg / 2.0 mg
**6. Single-injection spinal**	Provided
6.1 Using local anesthetic	Agree
6.2 Add opioid	Agree
6.3 Platelet concentration corresponding to contraindications to puncture	Less than $50*10^9$/L
6.4 Puncture location	L_{3-4}

6.6 Analgesic concentrations (consistent with 5.6)	Agree
6.7 Spinal needles	Provided
-Cutting-bevel	Disagree
-Pencil-point	Agree
**7. Patient-controlled epidural analgesia (for labor analgesia)**	Agree
**Anesthetic Care for caesarean Delivery**	
**1. Equipment, facilities, and support personnel**	Agree
**2. Continuous infusion epidural analgesia**	Provided
2.1 Using local anesthetic	Agree
2.2 Add opioid	Agree
2.3 Platelet concentration corresponding to contraindications to puncture	Less than $70*10^9/L$
2.4 Puncture location	L_{1-2} or L_{2-3}
2.5 Recommend level of block	T_{6-4}
2.6 Analgesic concentrations	Provided
-Lidocaine	1.5%-2%
-Bupivacaine	0.50%
-Ropivacaine	0.5%-0.75%
-Levobupivacaine	0.5%-0.75%
-Cloprocaine	3%
2.7 Prevention of local anesthetic poisoning	Provided
-Withdrawal before injection	Agree
-Administration of test dose	Agree
-Addition of epinephrine	Agree
**3. Single-injection spinal**	Provided
3.1 Using local anesthetic	Agree
3.2 Add opioid	Agree
3.3 Platelet concentration corresponding to contraindications to puncture	Less than $70*10^9/L$
3.4 Puncture location	L_{3-4}
3.5 Recommend level of block	T_{6-4}
3.6 Analgesic concentrations	Provided
-Bupivacaine	5~15 mg
-Ropivacaine	10~20 mg
3.7 Spinal needles	Provided
-Cutting-bevel	Disagree
-Pencil-point	Agree
**4. Combined spinal–epidural analgesia**	Provided
4.1 Puncture location	Provided
-Single space technique	L_{3-4}
-Double-space technique	T_{12}-L_2/L_{3-5}
4.2 Anesthetics (consistent with spinal or epidural)	Agree
**5. General anesthesia**	Provided
5.1 General anesthesia selected condition	Provided
-Profound fetal bradycardia	Agree
-Ruptured uterus	Agree
-Severe hemorrhage	Agree

-Severe placental abruption	Agree
-Umbilical cord prolapse	Agree
-Preterm footling breech	Agree
-Inability to place neuraxial anesthesia	
5.2 Intravenous anesthetics recommendation	Provided
-Thiopental sodium	4-5 mg/kg
-Propofol	1.5-2.5 mg/kg
-Etomidate	0.2-0.3 mg/kg
-Ketamine	0.5-1 mg/kg
5.3 Opioids analgesic recommendation	Provided
-Fentanyl	2-5 μg/kg
-Sufentanyl	0.2-0.5 μg/kg
-Remifentanyl	0.5-1 μg/kg for IV
-Remifentanyl	4 ng/kg for target
5.4 Muscle relaxants recommendation	Provided
-Succinylcholine	1-1.5 mg/kg
-Rocuronium	0.6-1.2 mg/kg
5.5 Induction-delivery interval	within 10 min
**Neurological Monitoring Associated With Obstetric Neuraxial Block**	
**1. Screening method (During the recovery phase) -Straight-leg raising**	Agree
**2. Alerts incidences-unable to straight-leg raise at 4 h from the last dose**	Agree
**Respiratory Depression Monitoring With Neuraxial Block With Opioids**	
**1. Frequency and modality of respiratory monitoring should be based on:**	Provided
1.1 Low risk	Provided
-Neuraxial morphine dose (intrathecal ≤ 0.15 mg or epidural ≤ 3 mg)	Agree
1.2 High risk	Provided
-Cardiopulmonary/neurological co-morbidity	Agree
-Obesity (body mass index ≥ 40 kg/m^2)	Agree
-Known or suspected obstructive sleep apnea	Agree
-Chronic opioid use or abuse	Agree
-Hypertension	Agree
-Magnesium administration	Agree
-Neuraxial morphine dose (intrathecal > 0.15 mg or epidural > 3 mg)	Agree
1.3 Respiratory monitoring frequency and duration	Provided
-Low risk	Provided
--Routine postoperative vital sign monitoring	Agree
-- q2h clinical assessment for 12 h	Agree
-High risk	Provided
-- q1h clinical assessment for first 12 h	Agree
-- q2h clinical assessment for first 12-24 h	Agree
--Consider additional monitoring modalities (e.g., pulse oximetry, capnography)	Agree
**Management of Obstetric and anesthetic Emergencies or Adverse Events**	
**1. Resources for management of hemorrhagic emergencies**	Provided
-Large-bore IV catheters	Agree
-Fluid warmer	Agree
-Forced-air body warmer	Agree

-Availability of blood bank resources	Agree
-Massive transfusion protocol (type-specific, or O-negative blood, or salvage)	Agree
-Equipment for infusing IV fluids and blood products rapidly	Agree
**2. Hypotension prevention strategies**	Provided
2.1 Blood pressure target of systolic arterial pressure	⩾ 90% of baseline obtained
2.2 Position (uterine left-leaning)	Yes
2.3 Volume expansion (crystals or colloids)	500-1000 ml
2.4 Vasoactive drugs	Yes
-Pump infusion is recommended	Provided
-Type of drugs	Provided
--Phenylephrine	20-50 μg or 0.5 μg. kg^{-1}. min^{-1}
--Methoxymine	1-2 mg or 4 μg. kg-1. min-1
--Noradrenalin	4-6 μg or 0.08 μg. kg-1. min-1
--Efedrina	5-15 mg for IV
2.5 Prophylactic vasopressor infusion should not be recommended in pre-eclampsia	Agree
**3. Equipment for management of airway emergencies**	Provided
3.1 Laryngoscope and assorted blades	Agree
3.2 Endotracheal tubes, with stylets	Agree
3.3 Oxygen source	Agree
3.4 Suction source with tubing and tonsil suction tip	Agree
3.5 Self-inflating bag and mask for positive-pressure ventilation	Agree
3.6 Medications for blood pressure support, muscle relaxation, and hypnosis	Agree
3.7 Tracheal intubation has failed protocol	Agree
**4. Cardiopulmonary Resuscitation**	Provided
4.1 Basic and advanced life-support equipment	Agree
4.2 Cardiac arrest occurs	Provided
-Uterine displacement (usually left displacement) should be maintained	Agree
-If circulation is not restored within 4 min, caesarean should be performed	Agree
**5. Emergency caesarean delivery**	Provided
5.1 Response times limited	30 minutes
5.2 Anesthesia methods recommendation	Provided
-Spinal	Agree
-Epidural	Agree
-Combined spinal–epidural analgesia	Agree
-General anesthesia	Agree
**6. Inadequate neuraxial anesthesia**	Provided
6.1 Block assessment	Provided
-Testing sensory block after the evidence of motor block	Agree
-Check the block at relatively fixed time intervals	Agree
-Perform the first check early enough to allow positional changes	Agree
6.2 Rescue for inadequate neuraxial block (change anesthesia method)	Provided
-A second neuraxial technique (in the case of spinal anesthesia)	Agree
-Extending the neuraxial technique (in the case of combined spinal–epidural)	Agree
-Above options are not possible, general anesthesia should be recommended	Agree

6.3 Rescue for inadequate neuraxial block (change anesthetic)	Provided
-Nitrous oxide and oxygen alone	Disagree
-Continue with neuraxial block, consider repeated boluses of fastacting opioids	Agree
--Fentanyl	25-50 µg
--Alfentanil	250-500 µg
--Ketamine	10 mg boluses
-Do not treat pain with anxiolytics	Agree
-Above options are not possible, general anesthesia should be recommended	Agree
**Management of Intra- or Postoperative Pain**	
**1. Implement strategies to minimize systemic opioid utilization**	Provided
**2. Neuraxial adjuvant drugs**	Provided
-Spinal anesthesia	Provided
--Morphine	50-100 µg
--Hydromorphone	75-150 µg
-Epidural or combined spinal-epidural technique.	Provided
--Morphine	2-3 mg
--Diamorphine	2-3 mg
**3. Others analgesic (after delivery)**	Provided
-Non-steroidal anti-inflammatory drug (e.g., paracetamol)	Agree
-A single dose of intravenous dexamethasone	Agree
**4. Local anesthetic infiltration**	Agree
**5. Continuous wound local anesthetic infusion**	Agree
**6. Fascial plane blocks (e.g., transversus abdominis plane, erector spinae plane)**	Agree
**7. Patient-controlled intravenous analgesia**	Agree

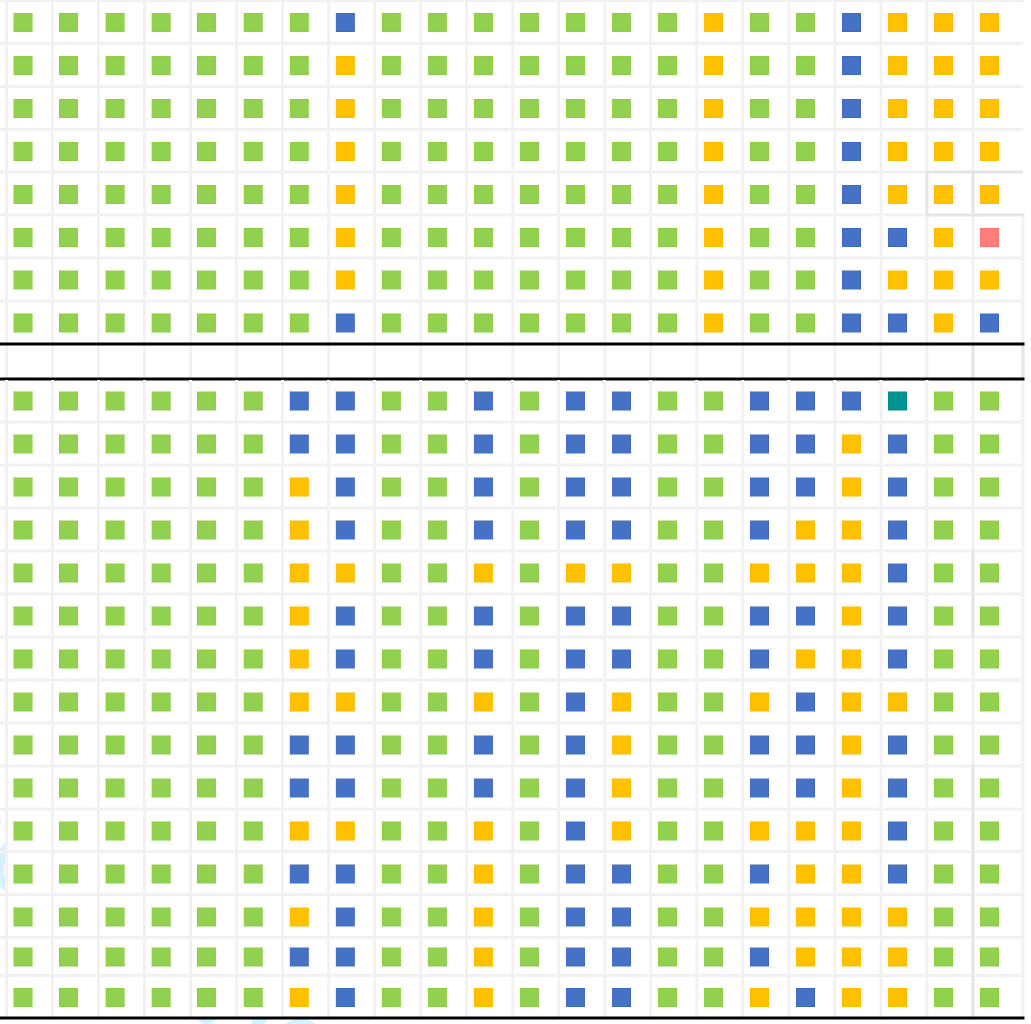

For peer review only

PRISMA 2020 Checklist

Section and Topic	Item #	Checklist item	Location where item is reported
**TITLE**			Page 1
Title	1	Identify the report as a systematic review.	
**ABSTRACT**			Page 2
Abstract	2	See the PRISMA 2020 for Abstracts checklist.	
**INTRODUCTION**			Page 5
Rationale	3	Describe the rationale for the review in the context of existing knowledge.	
Objectives	4	Provide an explicit statement of the objective(s) or question(s) the review addresses.	
**METHODS**			Page 6 to 9
Eligibility criteria	5	Specify the inclusion and exclusion criteria for the review and how studies were grouped for the syntheses.	
Information sources	6	Specify all databases, registers, websites, organisations, reference lists and other sources searched or consulted to identify studies. Specify the date when each source was last searched or consulted.	
Search strategy	7	Present the full search strategies for all databases, registers and websites, including any filters and limits used.	
Selection process	8	Specify the methods used to decide whether a study met the inclusion criteria of the review, including how many reviewers screened each record and each report retrieved, whether they worked independently, and if applicable, details of automation tools used in the process.	
Data collection process	9	Specify the methods used to collect data from reports, including how many reviewers collected data from each report, whether they worked independently, any processes for obtaining or confirming data from study investigators, and if applicable, details of automation tools used in the process.	
Data items	10a	List and define all outcomes for which data were sought. Specify whether all results that were compatible with each outcome domain in each study were sought (e.g. for all measures, time points, analyses), and if not, the methods used to decide which results to collect.	
	10b	List and define all other variables for which data were sought (e.g. participant and intervention characteristics, funding sources). Describe any assumptions made about any missing or unclear information.	
Study risk of bias assessment	11	Specify the methods used to assess risk of bias in the included studies, including details of the tool(s) used, how many reviewers assessed each study and whether they worked independently, and if applicable, details of automation tools used in the process.	
Effect measures	12	Specify for each outcome the effect measure(s) (e.g. risk ratio, mean difference) used in the synthesis or presentation of results.	
Synthesis methods	13a	Describe the processes used to decide which studies were eligible for each synthesis (e.g. tabulating the study intervention characteristics and comparing against the planned groups for each synthesis (item #5)).	
	13b	Describe any methods required to prepare the data for presentation or synthesis, such as handling of missing summary statistics, or data conversions.	
	13c	Describe any methods used to tabulate or visually display results of individual studies and syntheses.	
	13d	Describe any methods used to synthesize results and provide a rationale for the choice(s). If meta-analysis was performed, describe the model(s), method(s) to identify the presence and extent of statistical heterogeneity, and software package(s) used.	
	13e	Describe any methods used to explore possible causes of heterogeneity among study results (e.g. subgroup analysis, meta-regression).	
	13f	Describe any sensitivity analyses conducted to assess robustness of the synthesized results.	
Reporting bias assessment	14	Describe any methods used to assess risk of bias due to missing results in a synthesis (arising from reporting biases).	
Certainty	15	Describe any methods used to assess certainty (or confidence) in the body of evidence for an outcome.	

For peer review only - http://bmjopen.bmj.com/site/about/guidelines.xhtml

PRISMA 2020 Checklist

Section and Topic	Item #	Checklist item	Location where item is reported
assessment			
**RESULTS**			Page 10 to 21
Study selection	16a	Describe the results of the search and selection process, from the number of records identified in the search to the number of studies included in the review, ideally using a flow diagram.	
	16b	Cite studies that might appear to meet the inclusion criteria, but which were excluded, and explain why they were excluded.	
Study characteristics	17	Cite each included study and present its characteristics.	
Risk of bias in studies	18	Present assessments of risk of bias for each included study.	
Results of individual studies	19	For all outcomes, present, for each study: (a) summary statistics for each group (where appropriate) and (b) an effect estimate and its precision (e.g. confidence/credible interval), ideally using structured tables or plots.	
Results of syntheses	20a	For each synthesis, briefly summarise the characteristics and risk of bias among contributing studies.	
	20b	Present results of all statistical syntheses conducted. If meta-analysis was done, present for each the summary estimate and its precision (e.g. confidence/credible interval) and measures of statistical heterogeneity. If comparing groups, describe the direction of the effect.	
	20c	Present results of all investigations of possible causes of heterogeneity among study results.	
	20d	Present results of all sensitivity analyses conducted to assess the robustness of the synthesized results.	
Reporting biases	21	Present assessments of risk of bias due to missing results (arising from reporting biases) for each synthesis assessed.	
Certainty of evidence	22	Present assessments of certainty (or confidence) in the body of evidence for each outcome assessed.	
**DISCUSSION**			Page 21 to 26
Discussion	23a	Provide a general interpretation of the results in the context of other evidence.	
	23b	Discuss any limitations of the evidence included in the review.	
	23c	Discuss any limitations of the review processes used.	
	23d	Discuss implications of the results for practice, policy, and future research.	
**OTHER INFORMATION**			Page 27
Registration and protocol	24a	Provide registration information for the review, including register name and registration number, or state that the review was not registered.	
	24b	Indicate where the review protocol can be accessed, or state that a protocol was not prepared.	
	24c	Describe and explain any amendments to information provided at registration or in the protocol.	
Support	25	Describe sources of financial or non-financial support for the review, and the role of the funders or sponsors in the review.	
Competing interests	26	Declare any competing interests of review authors.	
Availability of data, code and other materials	27	Report which of the following are publicly available and where they can be found: template data collection forms; data extracted from included studies; data used for all analyses; analytic code; any other materials used in the review.	

For peer review only - http://bmjopen.bmj.com/site/about/guidelines.xhtml

PRISMA 2020 Checklist

From: Page MJ, McKenzie JE, Bossuyt PM, Boutron I, Hoffmann TC, Mulrow CD, et al. The PRISMA 2020 statement: an updated guideline for reporting systematic reviews. BMJ 2021;372:n71. doi: 10.1136/bmj.n71

For more information, visit: http://www.prisma-statement.org/

For peer review only

BMJ Open

Appraisal of clinical practice guidelines and consensus statements on obstetric anesthesia: A systematic review using the AGREE II instrument

Journal:	*BMJ Open*
Manuscript ID	bmjopen-2024-084759.R2
Article Type:	Original research
Date Submitted by the Author:	10-May-2024
Complete List of Authors:	Huang, Lu; Sichuan University West China Second University Hospital, Department of anesthesiology; Sichuan University West China Second University Hospital Key Laboratory of Birth Defects and Related Diseases of Women and Children<br>Hu, Na; Sichuan University West China Second University Hospital, Department of Anesthesiology ; Sichuan University West China Second University Hospital Key Laboratory of Birth Defects and Related Diseases of Women and Children<br>Jiang, Ling; Sichuan University West China Second University Hospital, Department of anesthesiology; Sichuan University West China Second University Hospital Key Laboratory of Birth Defects and Related Diseases of Women and Children<br>Xiong, Xinglong; The Affiliated Hospital of Guizhou Medical University, Department of Anesthesiology<br>Shi, Jing; The Affiliated Hospital of Guizhou Medical University, Department of Anesthesiology<br>Chen, Dongxu; Sichuan University West China Second University Hospital, Department of anesthesiology; Sichuan University West China Second University Hospital Key Laboratory of Birth Defects and Related Diseases of Women and Children
**Primary Subject Heading**:	Anaesthesia
Secondary Subject Heading:	Obstetrics and gynaecology
Keywords:	Anaesthesia in obstetrics < ANAESTHETICS, Pain management < ANAESTHETICS, PAIN MANAGEMENT

SCHOLARONE™
Manuscripts

I, the Submitting Author has the right to grant and does grant on behalf of all authors of the Work (as defined in the below author licence), an exclusive licence and/or a non-exclusive licence for contributions from authors who are: i) UK Crown employees; ii) where BMJ has agreed a CC-BY licence shall apply, and/or iii) in accordance with the terms applicable for US Federal Government officers or employees acting as part of their official duties; on a worldwide, perpetual, irrevocable, royalty-free basis to BMJ Publishing Group Ltd ("BMJ") its licensees and where the relevant Journal is co-owned by BMJ to the co-owners of the Journal, to publish the Work in this journal and any other BMJ products and to exploit all rights, as set out in our licence.

The Submitting Author accepts and understands that any supply made under these terms is made by BMJ to the Submitting Author unless you are acting as an employee on behalf of your employer or a postgraduate student of an affiliated institution which is paying any applicable article publishing charge ("APC") for Open Access articles. Where the Submitting Author wishes to make the Work available on an Open Access basis (and intends to pay the relevant APC), the terms of reuse of such Open Access shall be governed by a Creative Commons licence – details of these licences and which Creative Commons licence will apply to this Work are set out in our licence referred to above.

Other than as permitted in any relevant BMJ Author's Self Archiving Policies, I confirm this Work has not been accepted for publication elsewhere, is not being considered for publication elsewhere and does not duplicate material already published. I confirm all authors consent to publication of this Work and authorise the granting of this licence.

For peer review only

Appraisal of clinical practice guidelines and consensus statements on obstetric anesthesia: A systematic review using the AGREE II instrument

Lu Huang[1,2], Na Hu[1,2], Ling Jiang[1,2], Xing Long, Xiong[3], Jing Shi[3], Dong Xu Chen[1,2, *] ,

Affiliation Information for All Authors:

1. Department of anesthesiology, West China Second Hospital of Sichuan University, Sichuan Province, Chengdu, 610041, China
2. Key Laboratory of Birth Defects and Related Diseases of Women and Children (Sichuan University), Ministry of Education
3. Department of Anesthesiology, The Affiliated Hospital of Guizhou Medical University, Guiyang, China

Corresponding to:
Dong Xu Chen, PhD. Department of anesthesiology, West China Second Hospital of Sichuan University, Sichuan Province, Chengdu, 610041, China (e-mail: scucdx@foxmail.com)

Abstract

Objectives: Despite the publication of hundreds of trials on obstetric anesthesia, the management of these conditions remains suboptimal. We aimed to assess the quality and consistency of guidance documents for obstetric anesthesia.

Design: Systematic review and quality assessment using the appraisal of guidelines for research and evaluation (AGREE) II methodology.

Data sources: PubMed and EMBASE (8 June 2023), three Chinese academic databases, six guideline databases (7 June 2023), and Google and Google scholar (1 August 2023).

Eligibility criteria: We included the latest version of international and national/regional clinical practice guidelines and consensus statements for the anesthetic management of pregnant patients during labor, nonoperative delivery, operative delivery, and selected aspects of perioperative monitoring, postpartum care, and analgesia, published in English or Chinese.

Data extraction and synthesis: Two reviewers independently screened the searched items and extracted data. Four reviewers independently scored documents using AGREE II. Recommendations from all documents were tabulated and visualized in a colored grid.

Results: Twenty-two guidance documents (14 clinical practice guidelines and 8 consensus statements) were included. Included documents performed well in the domains of scope and purpose (median 76.4%, interquartile range (IQR) 69.4 to 79.2%) and clarity of presentation (median 72.2%, IQR 61.1% to 80.6%), but were unsatisfactory in applicability (median 21.9%, IQR 13.5% to 27.1%) and editorial

For peer review only

independence (median 47.9%, IQR 6.3% to 73.2%). The majority of obstetric anesthesia guidelines or consensus centered on different topics. Less than thirty percent of them specifically addressed the management of obstetric anesthesia perioperatively. Recommendations were concordant on the perioperative preparation, and on some indications for the choice of anesthesia method. Substantially different recommendations were provided for some items, especially for preoperative blood type and screen, and for the types and doses of neuraxial administration.

Conclusions: The methodological quality in guidance documents for obstetric anesthesia necessitates enhancement. Despite numerous trials in this area, evidence gaps persist for specific clinical queries in this filed. One potential approach to mitigate these challenges involves the endorsement of standardized guidance development methods and the synthesis of robust clinical evidence, aimed at diminishing difference in recommendations.

For peer review only

Strengths and limitations of this study

1. A strength of this review is the comprehensive search strategy to identify clinical practice guidelines for obstetric anesthesia.

2. The review was restricted to documents published in English and Chinese.

3. AGREE II was used to evaluate the quality of guidelines with less attention on detailed recommendations and the potential for unconscious bias arose from the subjective assessment of the documents included.

4. Caution is warranted in interpreting the AGREE II results, as the AGREE framework assigns equal weighting to all six domains, irrespective of their individual significance.

For peer review only

Introduction

Obstetric anesthesia refers to labor analgesia, cesarean delivery anesthesia, pain management during the procedure of labor and delivery, and addressing obstetric emergencies. Epidural analgesia is considered the gold standard of intrapartum analgesia [1], with approximately 10%-83% of women giving birth under neuraxial anesthesia [2]. Despite technological advancements, anesthesiologists still encounter significant challenges in safely administering anesthesia to pregnant patients. Aligning with the 2021 World Patient Safety Day theme 'Safe Maternal and Newborn Care', the primary goal remains safeguarding the health of both mother and fetus during anesthesia administration.

To enhance anesthetic care for obstetric patients, many clinical guidelines and consensus statements have been created by healthcare institutions and professionals. They cover aspects like peri-anesthetic assessment, and management for vaginal and cesarean deliveries. For example, the American Society of Anesthesiologists (ASA) released obstetric anesthesia guidelines in 1998, with updates in 2007 and 2016 [3, 4]. In China, the consensus on obstetric anesthesia was initially published in 2007, with subsequent updated in 2017 and 2020 [5, 6]. Additionally, China published two editions of guidelines for labor analgesia in 2016 [7] and 2020 [8].

Despite the variety of documents, current guidelines and consensuses on obstetric anesthesia and analgesia provide varied recommendations, such as whether require a platelet count, whether to order or require a blood type and screen or cross-match [4]. Furthermore, guideline development may be influenced by the experience and

For peer review only

composition of the guideline development groups. Low-quality guidance documents would pose risk to individual patients and communities, and impede the application of guideline recommendations in clinical practice [9]. Some guidelines were not written clearly, which may lead to clinician misunderstanding or not identifying key areas clearly, contributing to misjudged prioritizing of clinical actions [10]. Hence, it is essential to summarize and compare existing obstetric anesthesia guidelines or consensus statements to ensure the safety of both maternal and neonates during obstetric anesthesia. In this study, we aimed to evaluate the scope, content, and consistency of current guidance documents on obstetric anesthesia and analgesia. The quality of included guidelines and consensus statements were evaluated using the Appraisal of Guidelines for Research and Evaluation (AGREE) II instrument and presented as scores.

Methods

This study was developed based on the Preferred Reporting Items for Systematic Reviews and Meta Analyses for Protocols (PRISMA-P) [11]. The protocol of this study was registered prospectively at the International Platform of Registered Systematic Review and Meta-analysis Protocol.

Literature search and selection criteria

We conducted a systematically search of PubMed and EMBASE from inception to 8 June, 2023, employing a comprehensive search strategy (see online

For peer review only

Supplementary Table 1 and 2) to identify guidelines and consensus recommendations for anesthetic management of pregnant during labor, nonoperative delivery, operative delivery, and selected aspects of perioperative monitoring, postpartum care, and analgesia. We also searched three academic databases for Chinese publications (the Chinese Biomedical Literature Database, Wanfang Data, and Chinese Medical Association Database; see online **Supplementary Table 3**) and six guideline databases from inception to 7 June, 2023, using search strategies tailored to different databases (online **Supplementary Table 4**). To ensure thoroughness, we conducted additional searched on Google and Google Scholar on 1 August, 2023. Additionally, we scanned the reference lists of the most relevant clinical practice guidelines and review articles to identify any additional relevant guidelines. We included the latest versions of all international and national/regional clinical practice guidelines and consensus statements for the obstetric anesthetic management published in English or Chinese. We defined clinical practice guidelines and consensus statements as documents providing recommendations for patient care, which are derived from a systematic review of existing evidence or from collective opinions of an expert panel [12]. The exclusion criteria were: (1) original investigation, study protocols, comments on existing guidelines or consensus, and conference abstracts or posters; (2) draft documents that are under development or not finalized; (3) previous documents replaced by updated versions from the same organization.

Two reviewers (L.H and D.X.C) independently screened all searched documents. Full texts for potentially relevant guidelines or consensus statements were retrieved and

For peer review only

examined for eligibility. Reasons for exclusion were provided for documents excluded during the full-text review (online **Supplementary Table 5**). Disagreements were resolved through discussion with a third reviewer (J.S).

Data extraction

The following data was extracted from each included document, including publication year, funding body, and evidence base. Additionally, the data encompassed recommendations pertaining to monitoring and management, postpartum care, and analgesia. Data were extracted by one investigator and cross-checked by another investigator. In the event of any discrepancies, consensus was reached through discussion and resolution.

Appraisal of guidance documents

The included documents were evaluated by four independent reviewers (L.H, N.H, L.J., and D.X.C) using the Appraisal of Guidelines for Research and Evaluation (AGREE) II tool [13]. AGREE II is a validated instrument widely used to assess the quality of clinical practice guidelines and consensus statements [14]. The tool consists of 23 items categorized into six domains: scope and purpose, stakeholder involvement, rigor of development, clarity of presentation, applicability, and editorial independence. Each item is rated on a seven-point scale, ranging from 1 (Strongly Disagree) to 7 (Strongly Agree), indicating the level of agreement. The method for each domain appraised is present in Supplementary methods. In the current study, all appraisers completed an

For peer review only

online training tutorial (Available: http://ww center-agree-ii-training-tools/ [Accessed September 1, 2023]) prior to commencing the review process to ensure standardization. Detailed instruction from the AGREE II User's Manual [13] and objective scoring criteria (online **Supplementary Table 6**) for each item were used to promote scoring consistency.

Synthesis of recommendations

We meticulously extracted recommendations pertaining to critical clinical queries from a comprehensive set to guidance and consensus documents. Recommendations were extracted by two investigators (L.H and N. H) and checked by another (X.L.X and D.X.C). To visually represent these recommendations, we utilized a color-coded grid to illustrate any difference (online **Summary of Key Recommendations**).

Statistical analysis

All data was analyzed using the SPSS V.25.0 software. Median and interquartile range (IQR) were computed for the domain scores. The inter-rater agreement was computed using the intraclass correlation coefficient (ICC) with a two-way random effects model for the total score was computed. The level of agreement (ICC) was categorized according to widely recognized thresholds: poor (<0.40), fair (0.40–0.59), good (0.60–0.74) or excellent (0.75–1.00) [15]. Following the approach described in a previously published article, a domain is considered to have superior quality if its score exceeds 50%.

For peer review only

Patient and public involvement

No patient involved.

Results

Search results

We retrieved 6921 citations, with 6797 being excluded after screening titles and abstracts for not meeting eligibility criteria (**Figure 1**). We thoroughly assessed the remaining 124 citations and excluded 102 as they did not align with our defined criteria. Ultimately, we included fourteen guidelines [4, 16-28] and eight consensus statements [6-8, 29-33].

Characteristics of included guidelines and consensus statements

A summary of the characteristics of the eligible guidelines and consensus statements is presented in **Table 1**. Fourteen of guidelines [4, 16-28] and eight consensus statements [6-8, 29-33] with the latest versions were identified, published from 2014 to 2023, involving 15 national or multi-international organizations predominantly comprising anesthesiologists or obstetricians and gynecologists from the United States, United Kingdom, Europe, and China. Eight documents [17, 18, 22, 24, 26-28, 30] clearly stated being externally reviewed. Eight of these guidelines [4, 6, 16, 18, 19, 21, 24, 25] included procedures for updating in response to emerging evidence, while only six [4, 16, 19, 22, 29, 32] disclosed their funding body. The focus of these guidelines was primarily on the obstetric patients undergoing vaginal delivery

For peer review only

or cesarean delivery (**Figure 2**), with eight documents [4, 6, 8, 18, 22-25] intended for use by anesthesiologists or other health care providers. Additionally, ten documents [4, 17-19, 21-23, 26, 27, 33] conducted systematic literature reviews as part of their development process, which eleven documents [4, 16-18, 21, 22, 24, 26, 27, 32, 33] reported the level of evidence supporting recommendations and grading the strength of the guidance recommendations themselves.

Appraisal of guidelines and consensus statements

The standardized AGREE II domain scores for each guidance document are presented in **Figure 3** and provided in value as online **Supplementary Table 6 and 7**. Mean scores for each AGREE II item can be found in **Supplementary Table 8**. The ICC were above 0.90 (online **Supplementary Table 9**). The overall quality of guidance documents varied both between documents across domains and within documents across domains. The ASA guideline published in 2016 [4] had the highest domain scores, with five domains scoring in the upper quartile. This was followed by the guidelines published by the Enhanced Recovery After Surgery Society Recommendations (ERAS) (part2) [27], American College of Obstetricians and Gynecologists (ACOG) [16], and Royal College of Anesthetists (RcoA) [24], all with four domains scoring above the upper quartile. In total, 81.8% (18 of 22) of the included documents were excellent quality. Guidance documents received the highest scores for the scope and purpose domain (median 76.4%, IQR 69.4%–79.2%) and the clarity of presentation domain (median 72.2%, IQR 61.1 %–80.6%). However, they received the

For peer review only

lowest scores for the applicability domain (median 21.9%, IQR 13.5%–27.1%) and the editorial independence domain (median 47.9%, IQR 6.3%–73.2%). No tendency of improvement in the quality score over time was observed.

Synthesis of recommendations

Approaches to the peri-anesthetic evaluation and preparation

Sixteen documents [4, 6-8, 16-18, 21, 22, 24-26, 28, 29, 31, 32]were included in the peri-anesthetic evaluation and preparation. Key recommendations are presented in **Supplementary Table 10**. Ten documents [4, 6-8, 16, 24, 25, 28, 29, 31] provided recommendations for history and physical examination in pregnant individuals, with an emphasis on maternal health, anesthetic history, and relevant obstetric history. Of these, six documents [4, 6-8, 16, 25] specially recommended examining the patient's back when a neuraxial anesthetic was planned. Eight documents [4, 6, 8, 16, 25, 26, 28, 29] highlighted the important of communication between obstetric providers, and anesthesiologists to recognize significant anesthetic or obstetric risk factors.

Six guidelines [4, 6, 7, 16, 21, 29] referenced in the literature emphasize the important of platelet count in predicting anesthesia-related complications. Three of these guidelines [4, 16, 21] recommended individualizing the platelet count based on a patient's medical history. Furthermore, five documents [4, 6, 7, 21, 29] discussed the necessity of blood type and screen or cross-match, with two of them[4, 21] suggesting that routine test is not essential for healthy and uncomplicated parturient (**Table 2**). Additionally, nine documents [4, 6-8, 16, 18, 22, 25, 29] indicated that fetal heart rate

For peer review only

patterns should be monitored during the anesthetic process.

Eleven documents [4, 6, 17, 18, 21, 24-26, 29, 31, 32] addressed aspiration prevention during delivery. Regarding fasting, five documents [4, 6, 26, 31, 32] suggested that clear liquids up to 2 hours before anesthesia induction, and six documents [4, 6, 26, 29, 31, 32] recommended a fasting period of 6-8 hours for solids. The use of antacids, H_2-receptor antagonists, and metoclopramide were also recommended for aspiration prophylaxis during cesarean delivery [4, 6, 17, 21, 24, 26, 31].

Five documents [7, 8, 16, 24, 25] discussed the use of heparin or low-molecular weight heparin during the peri- and post- anesthesia, and the ACOG [16] explicitly stated that prophylactic subcutaneous unfractionated heparin at a dosage of 5,000 units twice daily did not contraindicate neuraxial techniques, as long as catheter placement or removal more than 4-6 hours since last dose. In the case of intermediate-dose unfractionated heparin (7,500–10,000 units) more than 12 hours after the last dose or high-dose unfractionated heparin (total daily dose greater than 20,000 units) more than 24 hours after the last dose with activated partial thromboplastin time, it was deemed low risk to proceed with neuraxial anesthesia.

Approaches to the anesthetic care for labor and vaginal delivery

Eight documents [4, 7, 8, 16, 22, 24, 25, 31] addressed anesthetic care for labor and vaginal delivery (summarizing in **Supplementary Table 11**). Three documents [4, 7, 8] explicitly indicated that neuraxial analgesia could be administrated based on the

level of discomfort the parturient, without limitation of cervical dilation. The ASA suggested providing labor neuraxial analgesia for parturient in early labor (i.e. cervical dilations of less than 5 cm dilation) [4]. These documents [4, 7, 8] also recommended to offer neuraxial analgesia on an individualized basis. The ASA also provided recommendations on the administration of neuraxial techniques for patients attempting vaginal birth after previous cesarean delivery, as well as early placement of a neuraxial catheter [4].

There were three methods for neuraxial anesthetic: continuous infusion epidural analgesia (CIEA), combined spinal–epidural analgesia (CSEA), and single-injection spinal. Five documents [4, 7, 8, 16, 25] covered both CIEA and CSEA and recommended using local anesthetic, as well as adding opioids. Before a neuraxial procedure, the platelet count was required more than $70*10^9/L$ to reduce the risk of hematoma [8, 16, 25]. The puncture location was recommended on L2-3 or L3-4 in China published consensus [7, 8]. Local anesthetic including bupivacaine, ropivacaine, and levobupivacaine were used with loading dose or maintenance doses. In China, published consensus [7, 8] recommended using 0.04% to 0.125% bupivacaine or 0.0625% to 0.15% ropivacaine to initiate analgesia and maintain with 0.05% to 0.125% bupivacaine or 0.0625% to 0.15% ropivacaine (**Table 2**). The Chinese Associations of Anesthesiology (CAA) consensus also provided recommendations for 0.04% to 0.125% levobupivacaine for loading dose and 0.05% to 0.125% levobupivacaine for maintenance [8]. These two documents suggested that opioids, including fentanyl or sufentanyl, could be injected with local anesthetic, with recommended loading dose of

For peer review only

fentanyl was 0.5-2 μg.ml^{-1} and 1-2 μg.ml^{-1} for maintenance, and the loading dose of sufentanyl was 0.2-0.6 μg.ml^{-1} with maintenance dose 0.3-0.6 μg.ml^{-1} [7, 8]. The American Association of Nurse Anesthesiology (ANNA) guideline suggested using 0.0625% to 0.125% bupivacaine or 0.08% to 0.2% ropivacaine with or without opioids in CIEA [25]. In CSEA, opioids and local anesthetics could be delivered in spinal, with two recommended protocols [7, 8, 16, 25]: sufentanyl or ropivacaine used singly or in combination. The single dose of sufentanyl was 2.5-7 μg, single dose of ropivacaine was 2.3-3 mg, and dose of combined sufentanyl and ropivacaine was 2.5 μg/2.5 mg, respectively [7, 8]. Another protocol was that fentanyl and bupivacaine was used singly or with combination. The single dose of fentanyl was 15-25 μg, single dose of bupivacaine was 2.0-2.5 mg, and dose of combined fentanyl and bupivacaine was 2.5 μg/2.0 mg, respectively [7, 8]. For CSEA and single-injection spinal, both documents recommended using a small-gauge, non-cutting, pencil point needle to access the subarachnoid space [4, 25]. Additionally, five documents [4, 7, 8, 25, 31] provided recommendations for using patient-controlled epidural analgesia for labor analgesia.

For peer review only

Approaches to the anesthetic care for cesarean delivery

Ten documents [4, 6, 16, 18, 24, 25, 27-29, 31] covered the anesthetic care including neuraxial anesthesia and general anesthesia for cesarean delivery (summarizing in **Supplementary Table 12**). The recommendations for neuraxial anesthesia including CIEA, single-injection spinal, and CSEA were consistent with those for labor and vaginal delivery. Eight of the documents [4, 6, 16, 18, 24, 25, 27,

28, 31] covered CIEA, CSEA, and single spinal injection, with four documents [6, 16, 25, 28] recommended the use of local anesthetic with or without opioids. A platelet count more than $70*10^9$/L was recommended to reduce the risk of hematoma [6, 16, 25]. Puncture locations were recommended as follows: L1-2 or L2-3 for CIEA, L3-4 for single-injection in spinal, and L3-4 in single space for CSEA, while in double space, the puncture location was recommended in T12-L2 or L3-5 [6, 25]. To test the effect of neuraxial anesthesia, the recommended level of block was T6-4 [6]. Various local anesthetic, including lidocaine, bupivacaine, ropivacaine, and levobupivacaine were recommended for use in CIEA. In CAA 2020 [6], lidocaine was recommended with 1.5%-2% concentration, bupivacaine with 0.5%, ropivacaine with 0.5%-0.75% (**Table 2**). And 3% chloroprocaine was also recommended. In contrast, ANNA [25] recommended lower concentrations, with 0.0625-0.125% for bupivacaine and 0.08-0.2% for ropivacaine. For single injection spinal procedures, the recommended doses were 5-15 mg bupivacaine or 10-20 mg ropivacaine [6, 28]. To prevent local anesthetic poisoning, it was recommended that anesthesiologist withdraw before injection and administer a test dose [6, 16]. It was also suggested to add epinephrine to local anesthetic solutions to delay absorption and increase duration of blockade through vasoconstriction of the blood vessels in the area [6, 16]. Furthermore, the use of a small-gauge, non-cutting, pencil point needle was recommended [4, 6, 25].

Additionally, eight documents [4, 6, 16, 21, 24, 25, 29, 31] provided recommendations on general anesthesia for cesarean delivery. According to the ASA [4], general anesthesia may be the most appropriate choice in cases of profound fetal

For peer review only

bradycardia, ruptured uterus, severe hemorrhage, severe placental abruption, umbilical cord prolapse, and preterm footling breech. Other documents [16, 25] recommended the use of general anesthesia if neuraxial anesthesia placement was unsuccessful. Induction-delivery was recommended within 10 minutes, with specific intravenous anesthetics. CAA [6] detailly recommended intravenous anesthetics including thiopental sodium (4-5 mg.kg^{-1}), propofol (1.5-2.5 mg.kg^{-1}), etomidate (0.2-0.3 mg.kg^{-1}), and ketamine (0.5-1 mg.kg^{-1}) as induction agents. Fentanyl (2-5 µg.kg^{-1}), sufentanyl (0.2-0.5 µg.kg^{-1}), and remifentanil (4 ng.kg^{-1} for target) were commonly applied in the general anesthesia. Succinylcholine (1-1.5 mg.kg^{-1}) or rocuronium (0.6-1.2 mg.kg^{-1}) was used as a muscle relaxant.

Approaches to the monitoring of obstetric and anesthetic or intra- or postoperative pain management

To mitigate post-cesarean delivery pain, nine documents [16, 17, 19, 21, 23, 25, 31-33] provided recommendations for the management of intra- or postoperative pain (summarizing in **Supplementary Table 13**). Six of these documents [16, 19, 21, 25, 31-33] recommended the administration of intrathecal morphine 50-150 µg or epidural 1-3 mg for analgesia. Two documents [19, 21] also suggested the use of epidural diamorphine (2-3mg or 2.5-5 mg) for analgesia. ANNA [25] recommended hydromorphone 75-150 µg in intrathecal administration as well. When morphine was utilized in neuraxial block, four documents [21, 25, 29, 33] suggested monitoring respiratory frequency and modality. The Society of Obstetric Anesthesia and

For peer review only

Perinatology (SOAP) [33] explicitly stated that using morphine less than 0.15 mg or epidural morphine less than 3 mg in healthy parturient would pose low risk, and recommended monitoring respiratory rate and sedation measurement every 2 hours for 12 hours postoperatively. However, in high-risk circumstances such as cardiopulmonary or neurological comorbidity, obesity (body mass index ≥ 40 kg/m^2), obstructive sleep apnea, chronic opioid use or abuse, and hypertension, or who was administrated with magnesium, as well as high doses of morphine (morphine greater than 0.15 mg or epidural morphine greater than 3 mg), more intensive monitoring (respiratory rate and sedation assessments were monitored for every 1 h for first 12 h, every 2 h for 12–24 h) and additional modalities such as pulse oximetry and capnography were suggested (**Table 2**). To minimize systemic opioids utilization, eight documents [16, 17, 19, 21, 25, 31-33] recommended the administration of non-steroidal anti-inflammatory drug for postoperative pain. Two guidelines [19, 25] suggested a single dose of intravenous dexamethasone for analgesia. Local anesthetic infiltration for the wound after cesarean delivery was recommended by six documents [16, 17, 19, 25, 31, 32], and continuous local anesthetic infusion with the wound was recommended by three documents [16, 19, 31]. Nerve blocks, including transversus abdominis plane block and erector spinae plane block, for improving post-cesarean delivery pain was recommended by five documents [16, 17, 19, 31, 32]. Patient-controlled intravenous analgesia used for pain management after cesarean was recommended by four documents [16, 19, 21, 31]. Regarding obstetric neuraxial block, The Association of Anesthetists and the Obstetric Anesthetists' Association(AA and OAA) [20] stated that

For peer review only

straight-leg raising was used for neurological monitoring, and if a maternal was unable to straight-leg raise at 4 h from the last dose of epidural/spinal local anesthetic, neurological injury required be investigated.

Approaches to the adverse events during obstetric anesthesia

Twelve documents [4, 6, 8, 16-18, 21, 25, 27, 30-32] provided recommendations for preventing the hypotension during obstetric anesthesia (summarizing in **Supplementary Table 14**). Eleven documents [4, 6, 8, 16-18, 21, 25, 27, 30, 31] advised on using 500-1000 ml volume expansion with crystals or colloids, as well as seven documents [4, 6, 8, 21, 25, 30, 31] recommended utilizing left uterine displacement once the maternal was positioned supine for surgery to reduce inferior vena cava compression. Vasopressor management during neuraxial anesthesia was recommended by ten documents [4, 6, 8, 16, 17, 21, 25, 30-32], with vasoactive drugs such as phenylephrine, metaraminol, noradrenalin and ephedrine. Phenylephrine with 20-50 $\mu g.min^{-1}$ or 0.25-0.5 $\mu g.\ kg^{-1}.\ min^{-1}$ was recommended by nine documents [4, 6, 8, 16, 17, 25, 30-32]. Ephedrine 2-15 mg intravenous was recommended by six documents [6, 8, 16, 17, 30, 31] (**Table 2**). The Association of Anesthetists of Great Britain and Ireland (AAGBI) [30] recommended that metaraminol with 0.5 $mg.ml^{-1}$ could be used to mediate blood pressure. CAA [6] also recommended that noradrenalin 4-6 μg or 0.08 $\mu g.\ kg^{-1}.\ min^{-1}$ was useful for preventing hypotension. The target of blood pressure was to maintain systolic arterial pressure greater than 90% and avoid a decrease to < 80% baseline [21, 30]. For maternal with pre-eclampsia, two documents

For peer review only

[6, 30] advised that vasopressor infusion should not be prophylactically used.

Six documents [8, 16, 23-25, 28] provided recommendations for inadequate neuraxial anesthesia, three guidelines [23, 25, 28] suggested assessing other causes of pain, evaluating the catheter site, and reassessing the sensory block level. For rescuing inadequate anesthesia, four documents [16, 23, 25, 28] provided recommendations including administrating a second neuraxial technique or extending the neuraxial technique, repeating boluses of fast-acting opioids (fentanyl 25-50 μg, alfentanil 250-500 μg) or ketamine (10 mg boluses), and if necessary, recommending general anesthesia.

Unintentional dural puncture would lead to post-dural puncture headache (PDPH). AANA [25] provided recommendations on using small, non-cutting spinal needle or placing intrathecal catheter for preventing PDPH. Treatment options for PDPH included epidural blood patch, adequate hydration, multimodal analgesia, caffeine, sumatriptan, gabapentin, and sphenopalatine ganglion block.

Approaches to the management of obstetric and anesthetic emergencies

Four documents [4, 6, 24, 25] provided recommendations for managing hemorrhagic emergencies, with three of them [4, 6, 25] consistently recommending the use of large bore intravenous catheters (summarizing in **Supplementary Table 15**). The ASA [4] and ANNA [25] suggested the implementation of a massive transfusion protocol. Additionally, the ASA [4] recommended the availability of blood bank resource, fluid warmer, forced -air body warmer, and equipment for rapid infusion of

For peer review only

intravenous fluids and blood products.

Seven documents [4, 6, 16, 18, 21, 24, 25] addressed airway emergency in obstetric anesthesia, with three of them [4, 16, 25] recommending the availability of personnel and equipment, such as laryngoscope with assorted blades and endotracheal tubes with stylets. In cases where tracheal intubation fails, the use of emergency procedures such as cricothyrotomy was advised. Access to oxygen source and suction source with tubing were also recommended [4, 21, 25]. The ASA provided specific recommendations for medications to support blood pressure, muscle relaxation, and hypnosis [4].

Three documents [4, 24, 25] covered cardiopulmonary resuscitation, with both the ASA [4] and ANNA [25] recommending the availability of basic and advanced life-support equipment during cardiac arrest. It was also recommended to maintain uterine left displacement during cardiac arrest. If circulation was not restored within 4-5 min, two guidelines suggested performing a cesarean section [4, 24, 25].

Six documents [4, 16, 21, 24, 25, 28] addressed emergency cesarean delivery, with the National Institute for Health and Care Excellence (NICE) [21] providing specific recommendations for the time of unplanned and emergency cesarean births when there is a threat to the life of the woman or fetus, within 30 minutes. The use of neuraxial anesthesia and general anesthesia was recommended for emergency cesarean delivery [16, 21, 28].

Discussion

For peer review only

In this study, we systematically reviewed fourteen guidelines [4, 16-28] and eight consensus statements [6-8, 29-33]. The comparison of the content of the twenty-two documents showed that several documents were very comprehensive, covering many aspects of obstetric anesthesia [4, 6-8, 16, 18, 21, 24, 25, 27, 28, 31, 32],while other documents may focus on the specific contents [17, 19, 20, 22, 23, 26, 29, 30, 33]. We found generally average methodological quality and some varied recommendations from included documents covering the preoperative screening and management of obstetric anesthesia and analgesia. We summarized all key recommendations, and compared and visualized the difference among them, providing a concise but informative overview for clinicians and researchers.

Comparison with existing research

Our study systematically incorporated both guidelines and consensus statements in the field of obstetric anesthesia and analgesia, and we observed varied performance across different AGREE II domains for both types of documents. The assessment of the guidelines using the AGREE II tool revealed that the documents assessed in our study excelled in the domains of scope and purpose (domain 1) and clarity of presentation (domain 4); but performed poorly in the domain of applicability (domain 5) and editorial independence (domain 6). This distribution of AGREE II domain scores has been observed in previous guideline appraisal studies, with documents scoring higher in the scope and purpose domain and the clarity of presentation domain, and

For peer review only

lower in the applicability domain and the editorial independence domain. This pattern was not only observed in guidance documents for endocrinology diseases [34, 35], but also in documents for other clinical specialties [36, 37]. Despite generally low and varied scores in the applicability domain, guidance documents for obstetric anesthesia and analgesia performed notably worse compared to documents for other conditions [34, 36], indicating that improving the applicability of guidance in this field may be more challenging. One major barrier to achieving good applicability of guidance documents is the time and cost required to perform economic evaluations and pilot studies. A stable and long-term task force for guideline development is needed to conduct these evaluations and studies. While forming such a task force may be difficult in some regions and countries, guidance documents should at least inform the audience about the need to consider these issues. Low scores in the editorial independence domain often resulted from a lack of detailed information on the influence of the funding body and conflicts of interest, with 45.5% of included documents in the current study failing to provide this information. It is essential to disclose the potential influence of the pharmaceutical industry on the synthesis of clinical guidance and promote transparency in financial declarations.

Gaps, clinical implications and future research

Current guidance and consensus documents uniformly endorse thorough perioperative evaluations and tailored anesthesia methodologies for delivery, including cesarean sections. Notwithstanding these overarching agreements, variances

For peer review only

particularly in areas characterized by high uncertainty persist, likely stemming from the variable quality of the documents, gaps in current evidence, or reliance on antiquated studies. This phenomenon is commonplace across all medical disciplines, including obstetric anesthesia. It underscores the complexity of clinical decision-making and the evolving nature of medical knowledge. Thus, continuous learning and future research endeavors are essential in narrowing the gap and ensuring optimal patient care in obstetric anesthesia and beyond.

It is important to acknowledge that discrepancies in recommendations could stem from differing practices or organizational approaches internationally. For instance, some countries initiate blood transfusions following a type and screen with different procedures [38]. It is also imperative to recognize that numerous clinical considerations remain underrepresented in these guidance and consensus documents. Among these, the post-neuraxial anesthesia evaluation of nerve blocks warrants particular attention. This includes the necessity for continuous postoperative sensory monitoring, vigilant respiratory depression surveillance, and strategies for addressing inadequate analgesia.

In obstetric practice, neuraxial anesthesia is a cornerstone of pain management. However, it is not without risk. Atypical presentations of motor or sensory block, such as unexpectedly dense or prolonged effects, may herald complications including unintended intrathecal placement or, in rare instances, neurological pathologies. Our review reveals that among the documents evaluated, only the AA and OAA [20] provided recommendations for neurological monitoring, specifically endorsing the straight-leg raise test. There is a conspicuous need for enhanced vigilance in post-

For peer review only

procedure monitoring, particularly in the immediate post-partum period. Current clinical practice often overlooks the importance of neuraxial monitoring; a survey conducted in the UK highlighted that only 56% of units had a formal protocol for postoperative neurological assessment, with a few units deferring evaluation until after the first post-delivery day [39]. This discrepancy indicates that despite adequate guideline recommendations, clinical care often fails to meet expected standards. Therefore, when well-supported guidelines are introduced, it is crucial to intensify efforts to enhance the clinical adoption process. Shortening the timeframe for unified practice and enhancing the implementation efficacy of these guidelines can be achieved through the development of clinical practice pathways and the organization of training sessions to disseminate guideline updates. Additionally, greater emphasis should be placed on promoting the implementation of these guidelines. As the adage goes, there is much more to be gained in improving global health by applying existing knowledge than by investing in the development of new drugs.

Our review critically examined respiratory depression monitoring in the context of neuraxial anesthesia, particularly when neuraxial opioids such as morphine, fentanyl, sufentanyl, and remifentanil are administered. The ASA guideline [4] recommended continuous monitored of ventilation, oxygenation, and consciousness in patients receiving neuraxial opioids. They further suggested intensified monitoring for those with a heightened risk of respiratory depression. Although the widespread endorsement of opioids in neuraxial anesthesia, it seemed that the monitoring of respiratory depression wasn't sufficiently highlight by these guidance documents.

For peer review only

Neuraxial anesthesia, while widely employed, does not always successful. The definition of ‘inadequate’ varies within the literature, contributing to this broad prevalence range. The prevalence of inadequate neuraxial anesthesia ranged from 10.2% - 30.3%[40]. Although there were several guidelines [8, 16, 23-25, 28]provided some recommendations on the management of inadequate neuraxial anesthesia. Patel *et al.* [41] revealed that only 23% of clinicians have received formal training in management of inadequate neuraxial anesthesia. Notably, the literature indices a significant gap in both the identification and management of inadequate neuraxial analgesia. Future initiatives must prioritize the development and dissemination of standardized education and training protocols to effectively recognized and manage inadequate neuraxial anesthesia.

Emergency cesarean section, often performed in cases with life-threatening conditions for the mother and fetus. Decision-to-delivery intervals was recommended within 30 minutes in category 1 cesarean birth when there was immediate threat to the life of the woman or fetus, and within 75 minutes in category 2 cesarean birth when there was maternal or fetal compromise which is not immediately life-threatening [21]. Nevertheless, studies found that decision-to-delivery intervals were frequently unmet [42, 43]. There is significant heterogeneity in how decision-to-delivery intervals are defined and measured [9]. A pressing concern for anesthesiologists is the selection of anesthesia type- neuraxial anesthesia versus general anesthesia-in these emergent scenarios, for which the guidelines lack specific, universally applicable recommendations.

For peer review only

Limitations

Firstly, our inclusion of only English and Chinese documents may have overlooked key guidance from non-English/Chinese speaking regions, though we sought English versions where possible. Secondly, we focused on the anesthetic management for pregnant, some specialty guidelines which was also involved in pregnant (i.e. guidelines for cardiac arrest for maternal [44]) were not included. Thirdly, unconscious bias might have affected our subjective AGREE II ratings, warranting caution in their clinical application. The subjective nature of AGREE II scoring also challenges the reproducibility and comparison of different reviews. Lastly, certain topics in obstetric anesthesia, like epidural-related maternal fever, postpartum depression, and intra-partum hypothermia, were not covered.

Conclusions

The current obstetric anesthesia and analgesia guidelines require significant improvements in methodological quality. They showed inconsistencies in recommendations, highlighting the need for standardized guideline development and high-quality evidence synthesis. Crucially, areas like neuraxial analgesia management and monitoring of postoperative neurological functions and intrapartum fever are underrepresented, indicating a gap in evidence-based guidelines and necessitating comprehensive research.

For peer review only

Data availability statement

All data relevant to the study are included in the article or uploaded as supplementary information.

Ethics statements

Patient consent for publication

Not applicable.

Ethics approval

This study does not involve human participants. All analyses were based on previously published guidelines or consensus, thus no ethical approval is required.

Funding information

This study was funded, in part, by a grant from the Science and Technology Department of Sichuan Province (No. 2023NSFSC1570 and 24NSFSC6388).

Conflict of interest statement

The authors report no conflict of interest.

Author contributions

Dong Xu Chen designed the study. Lu Huang and Dong Xu Chen performed the literature search and screening and extracted the data. Lu Huang, Na Hu, Ling Jian, Xing Long Xiong, Jing Shi and Dong Xu Chen conducted the data analyses. Lu Huang and Dong Xu Chen created the figures and tables and drafted the manuscript. All authors participated in the interpretation of results, and critically revised the manuscript. All authors approved the final version of the manuscript for publication.

For peer review only

Reference

[1] Anim-Somuah M, Smyth RM, Cyna AM, Cuthbert A. Epidural versus non-epidural or no analgesia for pain management in labour. *Cochrane Database of Syst Rev* 2018;5:Cd000331. http://doi.org/10.1002/14651858.CD000331.pub4.

[2] Seijmonsbergen-Schermers AE, van den Akker T, Rydahl E, *et al*. Variations in use of childbirth interventions in 13 high-income countries: A multinational cross-sectional study. *PLOS Med* 2020;17:e1003103. http://doi.org/10.1371/journal.pmed.1003103.

[3] Practice guidelines for obstetric anesthesia: an updated report by the American Society of Anesthesiologists Task Force on Obstetric Anesthesia. *Anesthesiology* 2007;106:843-63. http://doi.org/10.1097/01.anes.0000264744.63275.10.

[4] Practice Guidelines for Obstetric Anesthesia: An Updated Report by the American Society of Anesthesiologists Task Force on Obstetric Anesthesia and the Society for Obstetric Anesthesia and Perinatology. *Anesthesiology* 2016;124:270-300. http://doi.org/10.1097/ALN.0000000000000935.

[5] Chinese Society of Anesthesiology. Chapter 8, expert consensus on the obstetric anesthesia. Expert consensus and guidelines for Anesthetic Management in China (original document in Chinese). Beijing: People′s Medical Publishing House; 2017.

[6] Chinese Society of Anesthesiology. Chapter 8, expert consensus on the obstetric anesthesia. Expert consensus and guidelines for Anesthetic Management in China (original document in Chinese). Beijing: People′s Medical Publishing House; 2020.

[7] Group of Obstetric Anesthesia of Chinese Society of Anesthesiology. Expert consensus on labour analgesia (original document in Chinese). *J Clin Anesthesiol* 2016; 32: 816-8.

[8] Chinese Society of Anesthesiology. Chapter 9, expert consensus on the labour analgesia. Expert consensus and guidelines for Anesthetic Management in China (original document in Chinese). Beijing: People′s Medical Publishing House; 2020.

[9] Winther LP, Mitchell AU, MØLler AM. Inconsistencies in clinical guidelines for obstetric anaesthesia for cesarean section. *Acta Anaesthesiol Scand* 2012;57:141-49.

http://doi.org/10.1111/aas.12004.

[10] Woolf SH, Grol R, Hutchinson A, Eccles M, Grimshaw J. Clinical guidelines: potential benefits, limitations, and harms of clinical guidelines. *BMJ* 1999;318:527-30. https://doi.org/10.1136/bmj.318.7182.527.

[11] Shamseer L, Moher D, Clarke M, *et al*. Preferred reporting items for systematic review and meta-analysis protocols (PRISMA-P) 2015: elaboration and explanation. *BMJ* 2015;350:g7647. https://doi.org/10.1136/bmj.g7647.

[12] Robin Graham, Michelle Mancher, Dianne Miller Wolman, Sheldon Greenfield, Earl Steinberg, editors. Clinical Practice Guidelines We Can Trust. Washington (DC): National Academies Press (US) 2011.

[13] Brouwers MC, Kho ME, Browman GP, *et al*. AGREE II: advancing guideline development, reporting, and evaluation in health care. *Prev Med* 2010;51:421-4. http://doi.org/10.1016/j.ypmed.2010.08.005.

[14] Deng Y, Luo L, Hu Y, Fang K, Liu J. Clinical practice guidelines for the management of neuropathic pain: a systematic review. *BMC anesthesiol* 2016;16:12. http://doi.org/10.1186/s12871-015-0150-5.

[15] Brouwers MC, Kho ME, Browman GP, *et al*. Development of the AGREE II, part 1: performance, usefulness and areas for improvement. *CMAJ* 2010;182:1045-52. http://doi.org/10.1503/cmaj.091714.

[16] American College of Obstetricians and Gynecologists' Committee on Practice Bulletins—Obstetrics. ACOG Practice Bulletin No. 209: Obstetric Analgesia and Anesthesia. *Obstet Gynecol* 2019;133:e208-e25. http://doi.org/10.1097/aog.0000000000003132.

[17] Macones GA, Caughey AB, Wood SL, *et al*. Guidelines for postoperative care in cesarean delivery: Enhanced Recovery After Surgery (ERAS) Society recommendations (part 3). *Am J Obstet Gynecol* 2019;221:247.e1-e9. http://doi.org/10.1016/j.ajog.2019.04.012.

[18] Maxwell C, Gaudet L, Cassir G, *et al*. Guideline No. 392-Pregnancy and Maternal Obesity Part 2: Team Planning for Delivery and Postpartum Care. *J Obstet Gynaecol*

For peer review only

Can 2019;41:1660-75. http://doi.org/10.1016/j.jogc.2019.03.027.

[19] Roofthooft E, Joshi GP, Rawal N, *et al*. PROSPECT guideline for elective cesarean section: updated systematic review and procedure-specific postoperative pain management recommendations. *Anaesthesia* 2020;76:665-80. http://doi.org/10.1111/anae.15339.

[20] Yentis SM, Lucas DN, Brigante L, *et al*. Safety guideline: neurological monitoring associated with obstetric neuraxial block 2020. *Anaesthesia* 2020;75:913-19. http://doi.org/10.1111/anae.14993.

[21] National Institute for Clinical Excellence. Cesarean Section. Published: 31 March 2021, Available at: https://wwwniceorguk/guidance/NG192 2021.

[22] Mei-Dan E, Jain V, Melamed N, *et al*. Guideline No. 428: Management of Dichorionic Twin Pregnancies. *J Obstet Gynaecol Can* 2022;44:819-34.e1. http://doi.org/10.1016/j.jogc.2022.05.002.

[23] Plaat F, Stanford SER, Lucas DN, *et al*. Prevention and management of intra-operative pain during cesarean section under neuraxial anaesthesia: a technical and interpersonal approach. *Anaesthesia* 2022;77:588-97. http://doi.org/10.1111/anae.15717.

[24] Royal College of Anaesthetists. Guidelines for the Provision of Anaesthetic Services: Chapter 9 Guidelines for the Provision of Anaesthesia Services for an Obstetric Population 2023. Available at: https://www.rcoa.ac.uk/gpas/chapter-9.

[25] American Association of Nurse Anesthesiology. Analgesia and Anesthesia for the Obstetric Patient. Available at: https://issuu.com/aanapublishing/docs/analgesia_and_anesthesia_for_the_obstetric_patient?fr=sN2ZlNTU2NDAxMjU.

[26] Wilson RD, Caughey AB, Wood SL, *et al*. Guidelines for Antenatal and Preoperative care in Cesarean Delivery: Enhanced Recovery After Surgery Society Recommendations (Part 1). *Am J Obstet Gynecol* 2018;219:523.e1-e15. http://doi.org/10.1016/j.ajog.2018.09.015.

[27] Caughey AB, Wood SL, Macones GA, *et al*. Guidelines for intraoperative care in

For peer review only

cesarean delivery: Enhanced Recovery After Surgery Society Recommendations (Part 2). *Am J Obstet Gynecol* 2018;219:533-44. http://doi.org/10.1016/j.ajog.2018.08.006.

[28] Keita H, Deruelle P, Bouvet L, *et al*. Raising awareness to prevent, recognise and manage acute pain during caesarean delivery: The French Practice Bulletin. *Anaesth Crit Care Pain Med* 2021;40:100934. https://doi.org/10.1016/j.accpm.2021.100934.

[29] Obstetrics Subgroup, Society of Obstetrics and Gynecology, Chinese Medical Association. Expert consensus on cesarean delivery. *Zhonghua fu chan ke za zhi* 2014;49:721-24.

[30] Kinsella SM, Carvalho B, Dyer RA, *et al*. International consensus statement on the management of hypotension with vasopressors during cesarean section under spinal anaesthesia. *Anaesthesia* 2017;73:71-92. http://doi.org/10.1111/anae.14080.

[31] China Healthy Birth Science Association. Expert Consensus on Enhanced Recovery after Delivery. *Progress in Obstetrics and Gynecology* 2020;29:561-67.

[32] Bollag L, Lim G, Sultan P, *et al*. Society for Obstetric Anesthesia and Perinatology: Consensus Statement and Recommendations for Enhanced Recovery After Cesarean. *Anesth Analg* 2021;132:1362-77. http://doi.org/10.1213/ane.0000000000005257.

[33] Bauchat JR, Weiniger CF, Sultan P, *et al*. Society for Obstetric Anesthesia and Perinatology Consensus Statement: Monitoring Recommendations for Prevention and Detection of Respiratory Depression Associated With Administration of Neuraxial Morphine for Cesarean Delivery Analgesia. *Anesth Analg* 2019;129:458-74. http://doi.org/10.1213/ane.0000000000004195.

[34] Wu CM, Wu AM, Young BK, *et al*. An Appraisal of Clinical Practice Guidelines for Diabetic Retinopathy. *Am J Med* 2016;31:370-5. http://doi.org/10.1177/1062860615574863.

[35] Fang Y, Yao L, Sun J, *et al*. Appraisal of clinical practice guidelines on the management of hypothyroidism in pregnancy using the Appraisal of Guidelines for Research and Evaluation II instrument. *Endocrine* 2018;60:4-14. http://doi.org/10.1007/s12020-018-1535-2.

For peer review only

[36] Gavriilidis P, Roberts KJ, Askari A, *et al*. Evaluation of the current guidelines for resection of hepatocellular carcinoma using the Appraisal of Guidelines for Research and Evaluation II instrument. *J Hepatol* 2017;67:991-98. http://doi.org/10.1016/j.jhep.2017.06.028.

[37] Li Q, Li X, Wang J, *et al*. Diagnosis and treatment for hyperuricemia and gout: a systematic review of clinical practice guidelines and consensus statements. *BMJ open* 2019;9:e026677. http://doi.org/10.1136/bmjopen-2018-026677.

[38] Pei Z, Szallasi A. Prevention of surgical delays by pre-admission type and screen in patients with scheduled surgical procedures: improved efficiency. *Blood transfus* 2015;13:310-2. http://doi.org/10.2450/2014.0172-14.

[39] Roderick E, Hoyle J, Yentis SM. A national survey of neurological monitoring practice after obstetric regional anaesthesia in the UK. *Anaesthesia* 2017;72:755-59. http://doi.org/10.1111/anae.13800.

[40] Patel R, Kua J, Sharawi N, *et al*. Inadequate neuraxial anaesthesia in patients undergoing elective cesarean section: a systematic review. *Anaesthesia* 2022;77:598-604. http://doi.org/10.1111/anae.15657.

[41] Patel R, Russell R, Plaat F, *et al*. Inadequate neuraxial anaesthesia during cesarean delivery: a survey of practitioners. *Int J Obstet Anesth* 2023;56:103905. http://doi.org/10.1016/j.ijoa.2023.103905.

[42] Ayeni OM, Aboyeji AP, Ijaiya MA, *et al*. Determinants of the decision-to-delivery interval and the effect on perinatal outcome after emergency cesarean delivery: a cross-sectional study. *Malawi Med J* 2021;33:28-36. http://doi.org/ 10.4314/mmj.v33i1.5.

[43] Kitaw TM, Limenh SK, Chekole FA, *et al*. Decision to delivery interval and associated factors for emergency cesarean section: a cross-sectional study. *BMC Pregnancy and Childbirth* 2021;21:224. http://doi.org/10.1186/s12884-021-03706-8.

[44] Lott C, Truhlář A, Alfonzo A, *et al*. European Resuscitation Council Guidelines 2021: Cardiac arrest in special circumstances. *Resuscitation* 2021;161:152-219. https://doi.org/10.1016/j.resuscitation.2021.02.011.

For peer review only

Table 1. Characteristics of included guidelines and consensus statements.

Document	Issuing organization	Country	Funding body	Target population	Target audience	Guideline development group	Guideline review	Guideline update	Evidence base	LOE	SOR
**Guidelines**											
**ASA_2016** [4]	ASA	US	Institutional	Intrapartum and postpartum patients with uncomplicated pregnancies or with common obstetric problems.	Anesthesiologists	Multi	NG	1999-2006-2016	CS and SLR	+	+
**ERAS_2018 (Part 1, 2, 3)** [17, 26, 27]	ERAS	Multination	NG	Cesarean delivery patients	NG	ERAS	External review	NG	SLR	+	+
**ACOG_2019** [16]	ACOG	US	Institutional	NG	NG	ACOG	NG	2002-2007-2013-2017-2019	NG	+	+
**SOGC_2019** [18]	SOGC	Canada	NG	Women with obesity who are pregnant or planning pregnancies	Health care providers	SOGC	External review	5 years	SLR	+	+
**SOGC_2022** [22]	SOGC	Canada	Institutional	Pregnant women with a dichorionic twin pregnancy	Health care providers	SOGC	External review	NG	SLR	+	+
**AA and OAA_2020** [20]	AA and OAA	UK	None	Obstetric neuraxial block patients	NG	AA and OAA	NG	NG	NG	-	-
**PROSPECT_2020** [19]	PROSPECT	Europe	OAA	Cesarean delivery patients under neuraxial anaesthesia	NG	Multi	NG	2014-2020	SLR	-	-
**NICE_2021** [21]	NICE	UK	NG	Cesarean delivery patients	NG	NICE	NG	2011-2014-2021	SLR	+	+
**OAA_2022** [23]	OAA	UK	None	Cesarean delivery patients	Anesthetists	OAA	NG	NG	CS and	-	-

For peer review only

									SLR		
**ANNA_2023** [25]	ANNA	US	NG	Obstetric patients during labor and delivery and related procedures	CRNAs	AANA	NG	2008-2023	NG	-	-
**RCoA_2023** [24]	RCoA	UK	NG	All pregnant women	Health care providers	RCoA	External review	Planned in 2024	NG	+	+
**French_2021**[28]	CARO	France	NG	Cesarean delivery patients	Stakeholders involved in the care of women undergoing caesarean section	Multi	External review	NG	NG	-	-
**Consensus statements**											
**OG-OG-CMA_2014** [29]	OG-OG-CMA	China	The Research Special Fund for Public Welfare Industry of Health of the Ministry of Health of China	Cesarean delivery patients	NG	OG-OG-CMA	NG	NG	NG	-	-
**OG-A-CMA_2016** [7]	OG-A-CMA	China	NG	Labor analgesia	NG	OG-A-CMA	NG	NG	NG	-	-
**AAGBI_2017** [30]	AAGBI	Europe (Ireland)	None	Cesarean delivery patients	NG	AAGBI	External review	NG	NG	-	-
**SOAP_2019** [33]	SOAP	US	None	Cesarean delivery patients	NG	SOAP	NG	NG	SLR	+	+
**CHBSA_2020** [31]	CHBSA	China	NG	All pregnant women	NG	CHBSA	NG	NG	NG	-	-
**CAA_2020** [6]	CAA	China	NG	All pregnant women	Anesthesiologists	CAA	NG	2008-2017-2020	NG	-	-
**CAA_2020** [8]	CAA	China	NG	Labor and vaginal delivery	Anesthesiologists	CAA	NG	NG	NG	-	-
**SOAP_2020** [32]	SOAP	US	NIH	Cesarean delivery patients	NG	SOAP	NG	NG	NG	+	+

For peer review only

ASA, American Society of Anesthesiologists; ERAS, Enhanced Recovery After Surgery; ACOG, American College of Obstetricians and Gynecologists; SOGC, Society of Obstetricians and Gynecologists of Canada; AA and OAA, Association of Anesthetists and the Obstetric Anesthetists' Association; PROSPECT, Procedure-specific postoperative pain management working group; NICE, National Institute for Health and Care Excellence; OAA, Obstetric Anesthetists' Association; ANNA, American Association of Nurse Anesthesiology; RcoA, Royal College of Anesthetists; CARO, Club anesthe´sie-re´animation en obste´trique; OG-OG-CMA, Obstetrics Group, Obstetrics and Gynecology Branch of Chinese Medical Association; OG-A-CMA, Obstetrics Group, Anesthesiology Branch of Chinese Medical Association; AAGBI, Association of Anesthetists of Great Britain and Ireland; SOAP, Society for Obstetric Anesthesia and Perinatology; CHBSA, China Healthy Birth Science Association; CAA, Chinese Association of Anesthesiology. LOE, level of evidence; SOR, strength of recommendation; SLR, systematic literature review. NG, not given.

For peer review only

Table2. Some different recommendations summarized.

		The most frequently recommended content	A partially consistent recommendation	Contrary recommendation
**Approaches to the peri-anesthetic evaluation and preparation**				
**Routine intrapartum platelet count**		Agree	-	Individualized
**Routine blood type and screen**		Agree	-	Individualized
**Routine blood cross-match**		Agree	-	Individualized
**Approaches to the anesthetic care for labor and vaginal delivery**				
**Bupivacaine**	Continuous infusion epidural analgesia	0.04% to 0.125%	0.0625% to 0.125%	-
	Single-injection spinal	No guideline	2.0-2.5mg	-
**Ropivacaine**	Continuous infusion epidural analgesia	0.0625% to 0.15%	0.08% to 0.2%	-
	Single-injection spinal	No guideline	2.3-3 mg	-
**Fentanyl**	Continuous infusion epidural analgesia	0.5 to 2 $\mu g.ml^{-1}$	1 to 2 $\mu g.ml^{-1}$	-
**Sufentanyl**	Continuous infusion epidural analgesia	0.2 to 0.6 $\mu g.ml^{-1}$	0.4 to 0.6 $\mu g.ml^{-1}$	-
**Approaches to the anesthetic care for cesarean delivery**				
**Bupivacaine**	Continuous infusion epidural analgesia	0.50%	0.0625% to 0.125%	
	Single-injection spinal	No guideline	5-15 mg	-
**Ropivacaine**	Continuous infusion epidural analgesia	0.5% to 0.75%	0.08% to 0.2%	
	Single-injection spinal	No guideline	10-20 mg	-
**Approaches to the monitoring of obstetric and anesthetic or intra- or postoperative pain management**				
**High risk maternal respiratory monitoring**		No guideline	q1h clinical assessment for first 12 h or half hour for 2 hours	-
**Neuraxial adjuvant drugs in spinal anesthesia**	Morphine	50 to 150 μg	150 to 500 μg	-
**Neuraxial adjuvant drugs in epidural or**	Diamorphine	2 to 3 mg	2.5 to 5 mg	-

For peer review only - http://bmjopen.bmj.com/site/about/guidelines.xhtml

**combined spinal-epidural technique.**				
**Approaches to the adverse events during obstetric anesthesia**				
**Vasoactive drugs used for preventing hypotension**	Phenylephrine	20-50 μg or 0.5 μg. kg $^{-1}$. min $^{-1}$	20~50 μg or 0.25. kg $^{-1}$. Min $^{-1}$	-
	Metaraminol	No guideline	1-2 mg or 4 μg. kg $^{-1}$. min^{-1} or 0.5 mg.ml^{-1}	-
	Noradrenalin	4-6 μg or 0.08 μg. kg $^{-1}$. min^{-1}	-	-
	Ephedrine	2 to 15 mg iv	2 to 4 mg	

Note: It is paramount to emphasize that the above contradictory recommendations serve as a concise overview, without claiming comprehensiveness.

For peer review only

Figures Legend

Figure 1. Flow diagram for literature search.

CBM, Chinese Biomedical Literature Database; CMAD, Chinese Medical Association Database; NGC, National Guideline Clearinghouse; GIN, Guidelines International Network; NICE, National Institute for Health and Care Excellence; SIGN, Scottish Intercollegiate Guidelines Network; TRIP, Turning Research into Practice Database. AGREE, appraisal of guidelines for research and evaluation.

Figure 2. A summary of the main focus of the included document.

ASA, American Society of Anesthesiologists; ERAS, Enhanced Recovery After Surgery; ACOG, American College of Obstetricians and Gynecologists; SOGC, Society of Obstetricians and Gynecologists of Canada; AA and OAA, Association of Anesthetists and the Obstetric Anesthetists' Association; PROSPECT, Procedure-specific postoperative pain management working group; NICE, National Institute for Health and Care Excellence; OAA, Obstetric Anesthetists' Association; ANNA, American Association of Nurse Anesthesiology; RcoA, Royal College of Anesthetists; OG-OG-CMA, Obstetrics Group, Obstetrics and Gynecology Branch of Chinese Medical Association; OG-A-CMA, Obstetrics Group, Anesthesiology Branch of Chinese Medical Association; AAGBI, Association of Anesthetists of Great Britain and Ireland; SOAP, Society for Obstetric Anesthesia and Perinatology; CHBSA, China Healthy Birth Science Association; CAA, Chinese Association of Anesthesiology.

Figure 3. Standardized domain scores for each document.

ASA, American Society of Anesthesiologists; ERAS, Enhanced Recovery After Surgery; ACOG, American College of Obstetricians and

For peer review only

Gynecologists; SOGC, Society of Obstetricians and Gynecologists of Canada; AA and OAA, Association of Anesthetists and the Obstetric Anesthetists' Association; PROSPECT, Procedure-specific postoperative pain management working group; NICE, National Institute for Health and Care Excellence; OAA, Obstetric Anesthetists' Association; ANNA, American Association of Nurse Anesthesiology; RcoA, Royal College of Anesthetists; OG-OG-CMA, Obstetrics Group, Obstetrics and Gynecology Branch of Chinese Medical Association; OG-A-CMA, Obstetrics Group, Anesthesiology Branch of Chinese Medical Association; AAGBI, Association of Anesthetists of Great Britain and Ireland; SOAP, Society for Obstetric Anesthesia and Perinatology; CHBSA, China Healthy Birth Science Association; CAA, Chinese Association of Anesthesiology.

For peer review only

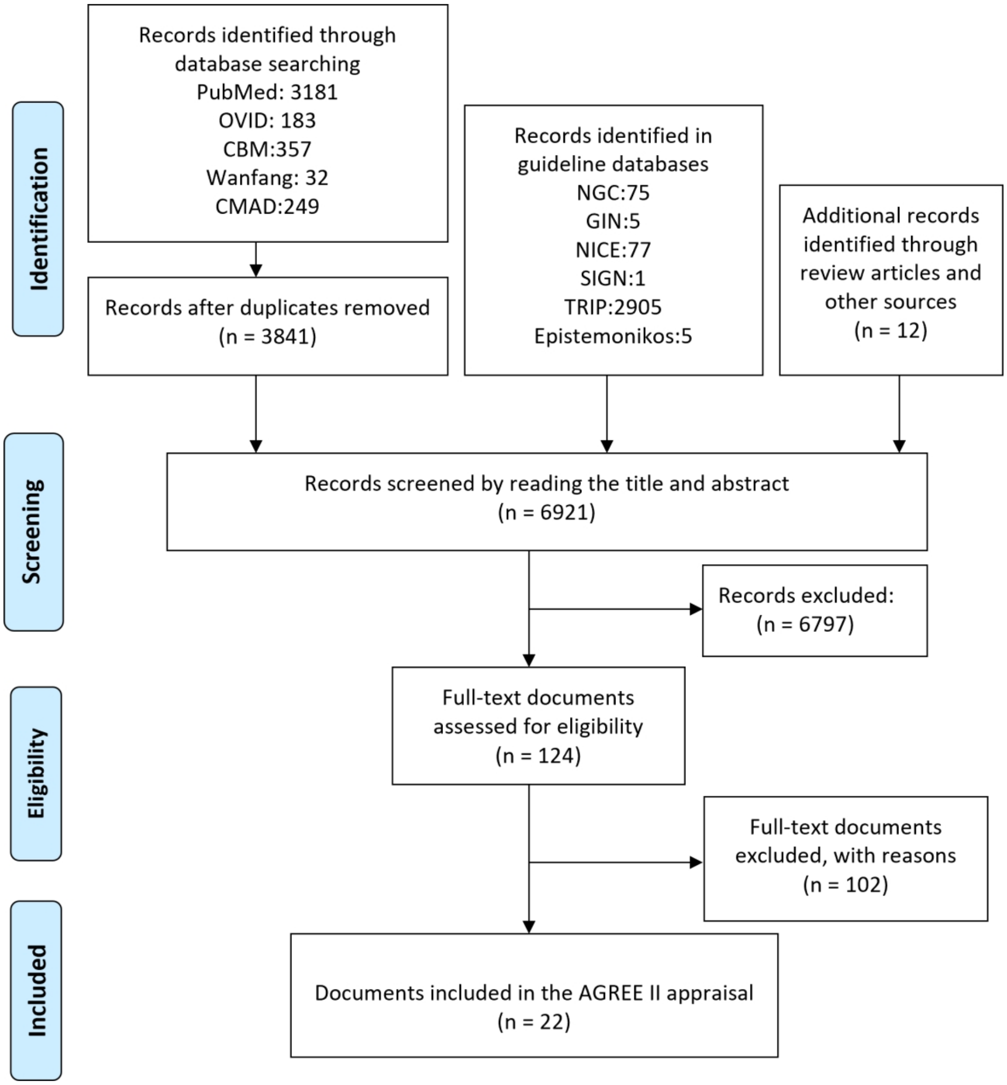

Flow diagram for literature search.

162x175mm (330 x 330 DPI)

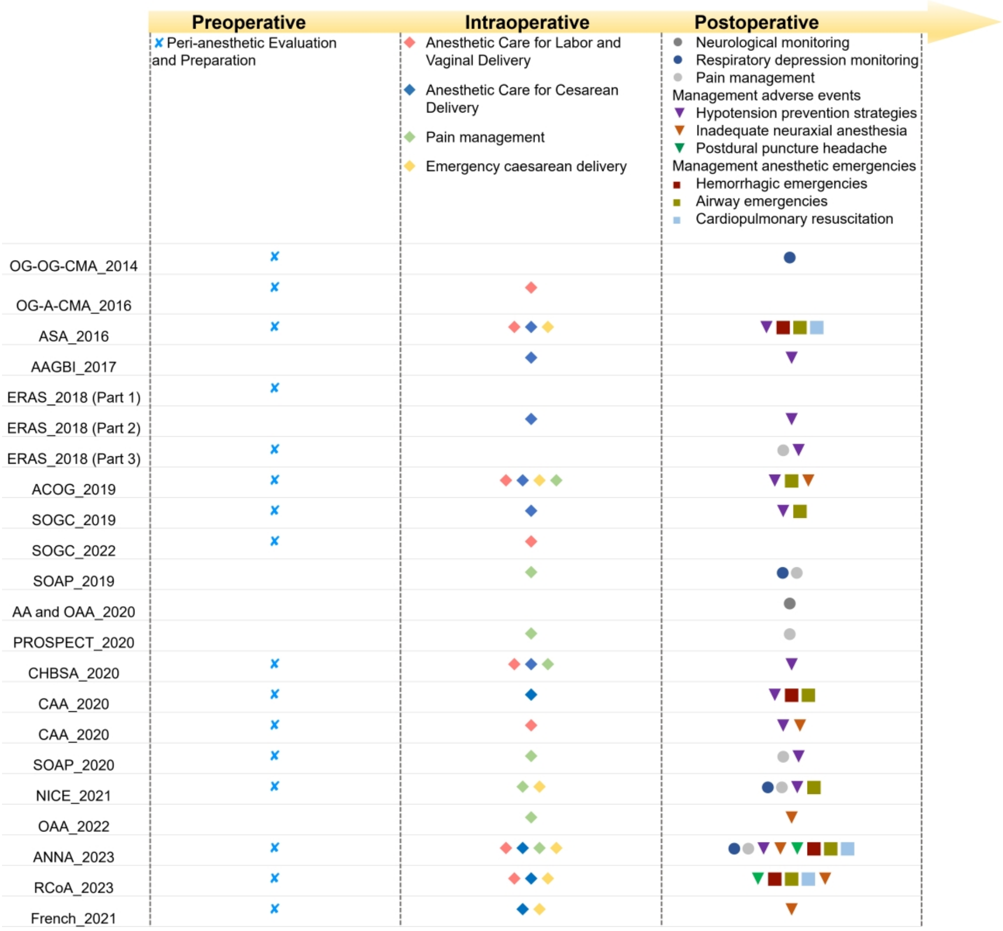

A summary of the main focus of the included document.

336x311mm (330 x 330 DPI)

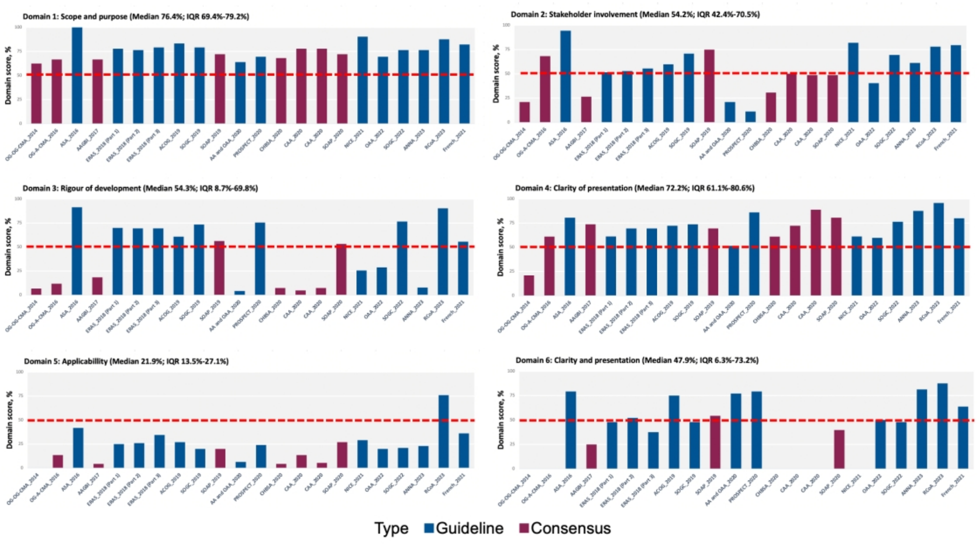

Standardized domain scores for each document

440x250mm (166 x 166 DPI)

Supplementary Materials

Contents

Supplementary Table 1. Search strategy in PubMed.
Supplementary Table 2. Search strategy in EMBASE using the OVID interface.
Supplementary Table 3. Searches in Chinese databases.
Supplementary Table 4. Searches in guideline databases.
Supplementary Table 5. Excluded studies and reasons for exclusion.
Supplementary methods. How to calculate the scores.
Supplementary Table 6. Scores for each individual AGREE II domain items by each reviewer.
Supplementary Table 7. Domain score for each included guidance document.
Supplementary Table 8. Mean scores across reviewers for the individual AGREE II domain items.
Supplementary Table 9. The intraclass correlation coefficient between reviewers for each individual AGREE II domain item.
Supplementary Table 10. Summary of recommendations for the peri-anesthetic evaluation and preparation by included guidance document.
Supplementary Table 11. Summary of recommendations for the anesthetic care for labor and vaginal delivery by included guidance document.
Supplementary Table 12. Summary of recommendations for the anesthetic care for cesarean delivery by included guidance document.
Supplementary Table 13. Summary of recommendations for the monitoring and management of obstetric and anesthetic or intra- or postoperative pain management by included guidance document.
Supplementary Table 14. Summary of recommendations for the management of adverse events by included guidance document.
Supplementary Table 15. Summary of recommendations for the management of obstetric and anesthetic emergencies by included guidance document.

For peer review only - http://bmjopen.bmj.com/site/about/guidelines.xhtml

Supplementary Table 1. Search strategy in PubMed.

1	((((((cesarean[Title/Abstract]) OR (cesarean delivery[Title/Abstract])) OR (cesarean section[Title/Abstract])) OR (obstetric[Title/Abstract])) OR (obstetrical delivery[Title/Abstract])) OR (obstetric anesthesia[Title/Abstract])) OR (delivery[Title/Abstract])	633,790
2	((expert consensus[Title/Abstract]) OR (guideline[Title/Abstract])) OR (recommendation statement[Title/Abstract])	88,342
3	("2000/01/01"[Date - Publication]: "3000"[Date - Publication])	22,218,196
4	1 AND 2 AND 3	3,181

Date of literature search: June 6, 2023

For peer review only

Supplementary Table 2. Search strategy in EMBASE using the OVID interface.

1	cesarean.m_titl.	26,523
2	cesarean delivery.m_titl.	8,147
3	cesarean section.m_titl.	12,708
4	obstetric.m_titl.	23,566
5	obstetrical delivery.m_titl.	16
6	obstetric anesthesia.m_titl.	961
7	delivery.m_titl.	185,141
8	1 or 2 or 3 or 4 or 5 or 6 or 7	224,835
9	guideline.m_titl.	29,968
10	expert consensus.m_titl.	3,906
11	recommendation statement.m_titl.	538
26	9 or 10 or 11	34,373
21	8 and 12	183

Date of literature search: Embase <1974 to 2023 June 06>

For peer review only

For peer review only - http://bmjopen.bmj.com/site/about/guidelines.xhtml

Supplementary Table 3. Searches in Chinese databases.

**Chinese Biomedical Literature Database**			
[Original search term in Chinese] (cesarean OR obstetric OR obstetric anesthesia) AND (guideline OR consensus OR statement OR recommendation)	Results found	Full text screened	Included documents
	357	2	1
Date of literature search: June 8, 2023			
**Wanfang Data**			
[Original search term in Chinese] (cesarean OR obstetric OR obstetric anesthesia) AND (guideline OR consensus OR statement OR recommendation)	Results found	Full text screened	Included documents
	32	2	2
Date of literature search: June 8, 2023			
**Chinese Medical Association Database**			
[Original search term in Chinese] (cesarean OR obstetric OR obstetric anesthesia) AND (guideline OR consensus OR statement OR recommendation)	Results found	Full text screened	Included documents
	249	4	2
Date of literature search: June 8, 2023			

For peer review only - http://bmjopen.bmj.com/site/about/guidelines.xhtml

Supplementary Table 4. Searches in guideline databases.

Databases	Date of search	Search strategy	Results found	Full text screened	Included documents	URL
National Guideline Clearinghouse	2023/6/7	cesarean OR cesarean section OR cesarean delivery OR obstetric OR obstetrical delivery OR obstetric anesthesia OR delivery	75	1	0	www.guideline.gov
Guidelines International Network	2023/6/7	cesarean OR cesarean section OR cesarean delivery OR obstetric OR obstetrical delivery OR obstetric anesthesia OR delivery<br>Search mode: Guidelines	5	1	1	www.g-i-n.net
National Institute for Health and Care Excellence	2023/6/7	cesarean OR obstetric<br>filter type: guidance	77	3	1	www.nice.org.uk
Scottish Intercollegiate Guidelines Network	2023/6/7	Not applicable	1	0	0	www.sign.ac.uk/our-guidelines.html
Turning Research Into Practice Database	2023/6/7	cesarean OR cesarean section OR obstetric OR obstetric anesthesia<br>filter: Guidelines	2,905	11	6	www.tripdatabase.com
Epistemonikos database	2023/6/7	cesarean OR obstetric OR obstetric anesthesia<br>filter: Broad syntheses OR Structured summaries	5	1	1	www.epistemonikos.org

For peer review only - http://bmjopen.bmj.com/site/about/guidelines.xhtml

Supplementary Table 5. Excluded studies and reasons for exclusion.

Study	Reason for exclusion
Caughey AB, Sundaram V, Kaimal AJ, et al. Maternal and neonatal outcomes of elective induction of labor. Evidence Report/Technology Assessment No. 176. (Prepared by the Stanford University-UCSF Evidenced-based Practice Centre under contract No. 290-02-0017.) AHRQ Publication No. 09-E005. Rockville, MD.: Agency for Healthcare Research and Quality. March 2009.	Not providing specific recommendations for obstetric anesthesia
Association of Ontario Midwives. Vaginal birth after previous low-segment cesarean section. 2021; (Clinical Practice Guideline No. 14).	Not providing specific recommendations for obstetric anesthesia
Obstetric Care Consensus: Safe prevention of the primary cesarean delivery. March 2014	Not providing specific recommendations for obstetric anesthesia
Guidelines for neuraxial analgesia or anesthesia in obstetrics. American Society of Anesthesiologists, October 13, 2021.	Conference abstract
Optimal goals for anesthesia care in obstetrics. American Society of Anesthesiologists, October 13, 2021.	Conference abstract
NICE guideline: Preterm labor and birth. 10 June 2022	Not providing specific recommendations for obstetric anesthesia
Y Gu, YB Xu, CH, Wang, et al. The reading of the United States Society for Obstetric Anesthesia and Perinatology: consensus statement on the management of cardiac arrest in pregnancy. Journal of Chinese Physician 2017; 19(9): 1286-1293.	Secondary interpretation of foreign guidelines, not original guidelines
Interpretation of the American College of Obstetricians and Gynecologists: 2017 Obstetric Analgesia and Anesthesia Practice Guide. Journal of practical obstetrics and gynecology 2017; 33 (8):586-589.	Secondary interpretation of foreign guidelines, not original guidelines
Expert Consensus on Obstetric Anesthesia in China (2008) and (2017)	Replaced by updated versions from the same organization
Hawkins JL. et al. Practice guidelines for obstetric anesthesia: an update report by the American Society of Anesthesiologists Task Force on Obstetric Anesthesia. Anesthesiology 2007; 106: 843–63.	Replaced by updated versions from the same organization

For peer review only - http://bmjopen.bmj.com/site/about/guidelines.xhtml

Opderbecke HW. et al. Vereinbarung über die Zusammenarbeit in der operativen Gynäkologie und in der Geburtshilfe. Anästh Intensivmed 1996; 37: 414–8.	Not English
Opderbecke HW. et al. Airway management. Anästh Intensivmed 2004; 45: 302–6.	Not English
Gogarten W. et al. Regionalanästhesieverfahren in der Geburtshilfe. Anästh Intensivmed 2004; 45: 151–3.	Not English
T. Piepho1. et al. S1 guidelines on airway management. Guideline of the German Society of Anesthesiology and Intensive Care Medicine. Anesthetist. 2015 Dec;64 Supple 1:27-40.	Not providing specific recommendations for obstetric anesthesia
The National Institute of Clinical Excellence (NICE) guidelines for cesarean sections: implications for the Anesthetist (2004), (2011) and (2014)	Replaced by updated versions from the same organization
Intrapartum Care: Care of Healthy Women and Their Babies During Childbirth (2014)	Replaced by updated versions from the same organization
Osborne, L. et al. Evidence-based anesthesia: fever of unknown origin in parturient and neuraxial anesthesia.	Replaced by updated versions from the same organization
Kearsley, A. et al. The development of a local guideline for epidural insertion in obstetric patients with signs of maternal sepsis. Anesthesia 2015. 70: 56.	Abstract
Korneliussen, J. et al. Implementation of new guideline recommendations, using ultra-low dose intrathecal morphine, for post-cesarean analgesia and its impact on pain, nausea and pruritus. Acta anesthesiologic Scandinavica 2023. 67: 537-538.	Abstract
Nasser, L. et al. IN SITU SIMULATION TO FACILITATE INTRODUCTION OF A NEW OBSTETRIC RAPID SEQUENCE OF ANESTHESIA GUIDELINE. BMJ Simulation and Technology Enhanced Learning 2017. 3: A3.	Abstract
Cohen, J. et al. Implementation of the Association of Anesthetists safety guideline: neurological monitoring associated with obstetric neuraxial block (2020): improving patient safety in a district general hospital labor ward requires a multidisciplinary and multimodal approach. Anesthesia 2022. 77: 12.	Conference abstract
McGuire, R. et al. Changing of the weather? Introduction of an evidence-based guideline for obstetric general anesthesia after regional surveys and the use of simulation to facilitate guideline implementation at University Hospital South Manchester. Anesthesia 2017. 72: 87.	Conference abstract

For peer review only - http://bmjopen.bmj.com/site/about/guidelines.xhtml

Han, W. Y. et al. Anesthesia and perioperative management for cesarean section in patient with factor XI deficiency: a case report. Beijing da xue xue bao. Yi xue ban. Health sciences 2014. 46: 329-332.	Case report
McDonnell, N. J. et al. Acute magnesium toxicity in an obstetric patient undergoing general anesthesia for cesarean delivery. International journal of obstetric anesthesia 2010. 19: 226-231.	Case report
Oji-Zurmeyer, J. et al. National survey of obstetric anesthesia clinical practices in the republic of Austria. International journal of obstetric anesthesia 2019. 39: 95-98.	Primary study
Terblanche, N. C. et al. A survey of Anesthetists on uterotonic usage practices for elective cesarean section in Australia and New Zealand. Anesthesia and intensive care 2021. 49: 440-447.	Primary study
Terfasa, E. A. et al. Obstetric analgesia utilization in labor pain management and associated factors among obstetric care providers in the West Shewa Zone, Central Ethiopia. SAGE open medicine 2022. 10: 20503121221088705.	Primary study
Thuillier, C. et al. Impact of recommended changes in labor management for prevention of the primary cesarean delivery. American journal of obstetrics and gynecology 2018. 218: 341.e341- e349.	Primary study
Cobb, B. et al. Do obstetric Anesthesiologists provide more guideline-concordant care for patients undergoing cesarean delivery? Anesthesia and analgesia 2018. 126: 404-408.	Primary study
Fiedler, A. et al. Adverse effect of delayed pushing on postpartum blood loss in nulliparous women with epidural analgesia. International journal of gynecology and obstetrics: the official organ of the International Federation of Gynecology and Obstetrics 2020. 150: 92-97.	Primary study
Gunka, V. et al. Determination of ChloraPrep® drying time before neuraxial anesthesia in elective cesarean delivery. A prospective observational study. International journal of obstetric anesthesia 2019. 38: 19-24.	Primary study
Kinsella, S. M. et al. Category-1 cesarean section: a survey of anesthetic and peri-operative management in the UK. Anesthesia 2010. 65: 362-368.	Primary study
Lee, E. K. et al. Investigating a Needle-Based Epidural Procedure in Obstetric Anesthesia. AMIA ... Annual Symposium proceedings. AMIA Symposium 2018. 2018: 720-729.	Primary study
Nasrallah, F. K. et al. The 30-minute decision-to-incision interval for emergency cesarean delivery: fact or fiction? American journal of perinatology 2004. 21: 63-68.	Primary study
Ward, C. et al. Does the presence of epidural analgesia reduce the risk of neonatal sepsis in the setting of an intrapartum fever? The journal of maternal-fetal & neonatal medicine: the official journal of the European	Primary study

For peer review only - http://bmjopen.bmj.com/site/about/guidelines.xhtml

Association of Perinatal Medicine, the Federation of Asia and Oceania Perinatal Societies, the International Society of Perinatal Obstet 2022. 35: 2110-2115.	
Jejaw, M. et al. Comprehensive emergency management of obstetric and newborn care program implementation at University of Gondar Comprehensive Specialized Hospital, Northwest Ethiopia, 2021: an evaluation study. Reproductive health 2023. 20: 76.	Primary study
Ji, H. et al. Factors contributing to the rapid rise of cesarean section: a prospective study of primiparous Chinese women in Shanghai. BMJ open 2015. 5: e008994.	Primary study
Johri, M. et al. A cluster-randomized trial to reduce cesarean delivery rates in Quebec: cost-effectiveness analysis. BMC medicine 2017. 15: 96.	Primary study
McDougall, A. R. A. et al. Expert consensus on novel medicines to prevent preterm birth and manage preterm labor: Target product profiles. BJOG: an international journal of obstetrics and gynecology 2022.	Not providing specific recommendations for obstetric anesthesia
Sultan, P. et al. Expert Consensus Regarding Core Outcomes for Enhanced Recovery after Cesarean Delivery Studies: A Delphi Study. Anesthesiology 2022. 137: 201-211.	Not providing specific recommendations for obstetric anesthesia
Bremerich, D. et al. S1 Guideline: Obstetric analgesia and anesthesia. Anesthesiology und Intensivmedizin 2020. 61: S300-S340.	Not English
Lavery, J. P. et al. Is the obstetric guideline of 30 minutes from decision to incision for Cesarean delivery clinically significant? Journal of healthcare risk management: the journal of the American Society for Healthcare Risk Management 1999. 19: 11-20.	Pre 2000
Santerre, R. E. The effect of the ACOG guideline on vaginal births after cesarean. Medical care research and review : MCRR 1996. 53: 315-329.	Pre 2000
Morris, J. M. et al. Protocol for the immediate delivery versus expectant care of women with preterm prelabor rupture of the membranes close to term (PPROMT) Trial [ISRCTN44485060]. BMC pregnancy and childbirth 2006. 6: 9.	Protocol
Gochnour, G. et al. The UTAH VBAC Study. Maternal and child health journal 2005. 9: 181-188.	Review
King, V. J. et al. Clinical practice guideline executive summary: Labor after cesarean/planned vaginal birth after cesarean. Annals of family medicine 2015. 13: 80-81.	Review

For peer review only - http://bmjopen.bmj.com/site/about/guidelines.xhtml

Keag, O. E. et al. Long-term risks and benefits associated with cesarean delivery for mother, baby, and subsequent pregnancies: Systematic review and meta-analysis. PLoS medicine 2018. 15: e1002494.	Review
Gulbransen, K. et al. Scoping Review of Best Practice Guidelines for Care in the Labor and Birth Setting of Pregnant Women Who Use Methamphetamines. Journal of obstetric, gynecologic, and neonatal nursing: JOGNN 2022. 51: 141-152.	Review
Hocaloski, S. et al. Perinatal Care for Women with Spinal Cord Injuries: A Collaborative Workshop for Consensus on Care in Canada. Topics in spinal cord injury rehabilitation 2017. 23: 386-396.	Review
Hong, J. et al. Comparison of Maternal Labor-Related Complications and Neonatal Outcomes Following Elective Induction of Labor at 39 Weeks of Gestation vs Expectant Management: A Systematic Review and Meta-analysis. JAMA network open 2023. 6: e2313162.	Review
Indermuhle, P. et al. Use of Scheduled Nonopioid Analgesia to Decrease Inpatient Opioid Consumption After Scheduled Cesarean Birth. Nursing for women's health 2022. 26: 344-352.	Review
James, B. et al. Obstetric general anesthetic safety checklist: Guideline development through team simulation. International journal of obstetric anesthesia 2015. 24: S24.	Review
Kuehn, B. M. Obstetrics group relaxes guideline for trial of labor after cesarean delivery. Jama 2010. 304: 951-952.	Review
Lagro-Janssen, T. Pain management during labor--an informed choice. Nederlands tijdschrift voor geneeskunde 2009. 153: A742.	Review
Mash, R. et al. Alternative mechanisms for delivery of medication in South Africa: A scoping review. South African family practice : official journal of the South African Academy of Family Practice/Primary Care 2021. 63: e1-e8.	Review
Melman, S. et al. Identification of barriers and facilitators for optimal cesarean section care: perspective of professionals. BMC pregnancy and childbirth 2017. 17: 230.	Review
Morris, S. et al. Breech presentation management: A critical review of leading clinical practice guidelines. Women and birth : journal of the Australian College of Midwives 2022. 35: e233-e242.	Review
Mutlu, S. et al. Evaluation of pregnancy, delivery, and postpartum effectiveness of maternity school trainings organized based on the guideline of ministry of health in Turkey: A comparative study. International journal of preventive medicine 2021. 12: 173.	Review

For peer review only

Heslehurst, N. et al. GestationaL Obesity Weight management: Implementation of National Guidelines (GLOWING): a pilot cluster randomized controlled trial of a guideline implementation intervention for the management of maternal obesity by midwives. Pilot and feasibility studies 2018. 4: 47.	Review
Lyerly, A. D. et al. Toward an ethically responsible approach to vaginal birth after cesarean. Seminars in perinatology 2010. 34: 337-344.	Review
Nasser, L. et al. Farewell thiopentone? Northwest deanery surveys of obstetric general anesthesia highlight increasing popularity of induction with propofol. Would a region-wide guideline improve training and safety? Anesthesia 2017. 72: 94.	Review
Neuhaus, S. et al. "Why mothers die". Learning from the analysis of anesthesia-related maternal deaths (1985-2013). Der Anesthetist 2016. 65: 281-294.	Review
Panda, S. et al. Review of cesarean section on maternal request in a tertiary care institute; scenario in developing country. Kathmandu University medical journal (KUMJ) 2013. 11: 349-354.	Review
Reale, S. C. et al. The Most Influential Publications in Obstetric Anesthesiology, 1998-2017: Utilizing the Delphi Method for Expert Consensus. Anesthesia and analgesia 2020. 131: 239-244.	Review
Roderick, E. et al. A national survey of neurological monitoring practice after obstetric regional anesthesia in the UK. Anesthesia 2017. 72: 755-759.	Review
Rungreungvanich, M. et al. An analysis of intraoperative recall of awareness in Thai Anesthesia Incidents Study (THAI Study). Journal of the Medical Association of Thailand 2005. 88 Supple 7: S95-101.	Review
Sakoda, A. et al. Review of 197 cases of urgent cesarean section performed in 2010 using NICE classification. Masui. The Japanese journal of Anesthesiology 2014. 63: 1339-1343.	Review
Sommerness, S. A. et al. The Perinatal Birth Environment: Communication Strategies and Processes for Adherence to a Standardized Guideline in Women Undergoing Second-Stage Labor With Epidural Anesthesia. The Journal of perinatal & neonatal nursing 2017. 31: 41-50.	Review
Terui, K. Obstetric anesthesia: from basics to recent advances in neonatal resuscitation: from Apgar score to NCPR program. Masui. The Japanese journal of Anesthesiology 2010. 59: 293-302.	Review
Van Gorp, S. F. J. et al. Do we need a national guideline for obstetric analgesia in the Netherlands? Evaluation of a questionnaire among dutch Anesthetists. Nederlands Tijdschrift voor Anesthesiology 2005. 18: 100-103.	Review
Williamson, R. M. et al. Availability of lipid emulsion in obstetric anesthesia in the UK: a national questionnaire	Review

For peer review only - http://bmjopen.bmj.com/site/about/guidelines.xhtml

survey. Anesthesia 2008. 63: 385-388.	
Winther, L. P. et al. Failed intubation and subsequent airway management during Cesarean section in general anesthesia. Ugeskrift for laeger 2010. 172: 1914-1916.	Review
Friedman, C. L. et al. Resident Attitudes Towards Cesarean Delivery in Canadian Obstetrics and Gynecology Residency Programs. Journal of obstetrics and gynecology. JOGC 2020. 42: 16-24.	Review
Bagou, G. et al. Guidelines for the management of urgent obstetric situations in emergency medicine, 2022. Anesthesia, critical care & pain medicine 2022. 41: 101127.	Not providing specific recommendations for obstetric anesthesia
Crawford, C. New guideline covers labor, vaginal birth after cesarean. Annals of family medicine 2015. 13: 186-187.	Not providing specific recommendations for obstetric anesthesia
Elammary, M. N. et al. Middle eastern college of obstetricians and gynecologists (MCOG) practice guidelines: Role of prediction models in management of trial of labor after cesarean section. Practice guideline no. 05-O-22. Journal of gynecology obstetrics and human reproduction 2023. 52: 102598.	Not providing specific recommendations for obstetric anesthesia
Fischer, T. et al. From the OEGGG - S3 Guideline "Vaginal delivery at term": The position of the Austrian Society of Gynecology and Obstetrics (OEGGG). Geburtshilfe und Frauenheilkunde 2021. 81: 857-861.	Not providing specific recommendations for obstetric anesthesia
Gagnon, R. et al. SOGC CLINICAL PRACTICE GUIDELINE: guidelines for the management of vasa previa. International journal of gynecology and obstetrics: the official organ of the International Federation of Gynecology and Obstetrics 2010. 108: 85-89.	Not providing specific recommendations for obstetric anesthesia
Hey, J. Clinical practice guideline on vaginal delivery of breech presentation. Journal of obstetrics and gynecology Canada. 2009. 31: 1123.	Not providing specific recommendations for obstetric anesthesia
Jain, V. et al. Guideline No. 402: Diagnosis and Management of Placenta Previa. Journal of obstetrics and gynecology Canada. 2020. 42: 906-917.e901.	Not providing specific recommendations for obstetric anesthesia
Jain, V. et al. Guidelines for the Management of a Pregnant Trauma Patient. Journal of obstetrics and gynecology Canada. 2015. 37: 553-574.	Not providing specific recommendations for obstetric

For peer review only - http://bmjopen.bmj.com/site/about/guidelines.xhtml

	anesthesia
Jain, V. et al. Guideline No. 439: Diagnosis and Management of Vasa Previa. Journal of obstetrics and gynecology Canada. 2023.	Not providing specific recommendations for obstetric anesthesia
Ladhani, N. N. N. et al. No. 347-Obstetric Management at Borderline Viability. Journal of obstetrics and gynecology Canada. 2017. 39: 781-791.	Not providing specific recommendations for obstetric anesthesia
Lalonde, A. B. Vaginal breech delivery guideline: the time has come. Journal of obstetrics and gynecology Canada. 2009. 31: 483-486.	Not providing specific recommendations for obstetric anesthesia
Marte, K. et al. Reduction in the Cesarean Delivery Rate after Obstetric Care Consensus Guideline Implementation. Obstetrics and gynecology 2016. 128: 1445.	Not providing specific recommendations for obstetric anesthesia
Maxwell, C. et al. SOGC Clinical Practice Guideline. Management guidelines for obstetric patients and neonates born to mothers with suspected or probable severe acute respiratory syndrome (SARS). No. 225, April 2009. International journal of gynecology and obstetrics: the official organ of the International Federation of Gynecology and Obstetrics 2009. 107: 82-86.	Not providing specific recommendations for obstetric anesthesia
Nelson, G. et al. Guideline for perioperative obstetrical care highlights evidence gap related to timing of urinary catheter removal after elective cesarean delivery. American journal of obstetrics and gynecology 2020. 222: 635.	Not providing specific recommendations for obstetric anesthesia
Oliver, E. et al. Comparison of international guidelines on the management of twin pregnancy. European journal of obstetrics, gynecology, and reproductive biology 2023. 285: 97-104.	Not providing specific recommendations for obstetric anesthesia
Podrasky, A. E. et al. ACR appropriateness Criteria® second and third trimester bleeding. Ultrasound quarterly 2013. 29: 293-301.	Not providing specific recommendations for obstetric anesthesia
Robinson, D. et al. Guideline No. 431: Postpartum Hemorrhage and Hemorrhagic Shock. Journal of obstetrics and gynecology Canada. 2022. 44: 1293-1310.e1291.	Not providing specific recommendations for obstetric

For peer review only - http://bmjopen.bmj.com/site/about/guidelines.xhtml

	anesthesia
Scott-Warren, V. et al. Development of a fibrinogen concentrate guideline for use in major obstetric Hemorrhage. Transfusion Medicine 2016. 26: 30.	Not providing specific recommendations for obstetric anesthesia
Stafford, I. A. et al. Association between Maternal Obesity Class, Adherence to Labor Guidelines, and Perinatal Outcomes. AJP reports 2021. 11: e105-e112.	Not providing specific recommendations for obstetric anesthesia
Triebwasser, J. et al. Effect of Single-Unit Transfusion Guideline on Red Blood Cell Transfusion for Obstetric Patients. Obstetrics and gynecology 2023. 141: 59S.	Not providing specific recommendations for obstetric anesthesia
Zhu, L. et al. The Chinese guideline for prevention of pelvic and abdominal adhesions after obstetric and gynecologic surgery (2023 edition). Zhonghua fu chan ke za zhi 2023. 58: 161-169.	Not providing specific recommendations for obstetric anesthesia
Bonnet, M. P. et al. Reprint of: Severe pre-eclampsia: guidelines for clinical practice from the French Society of anesthesiology and intensive care (SFAR) and the French College of gynaecologists and obstetricians (CNGOF). Gynecol Obstet Fertil Senol. 2022 Jan;50(1):2-25.	Not English

Reason for exclusion: Review; Primary study; Editorial; Conference abstract; Not providing specific recommendations for obstetric anesthesia; Multimedia section; Replaced by updated versions from the same organization; Conference abstract; Comment letter; Technology appraisal; Book; Not released by a professional association.

For peer review only

Supplementary methods. How to calculate the scores.

The reviewers individually assessed each item using a Likert scale, and the scores were combined to calculate the domain scores. The total score for each domain was calculated by summing the scores of all the items within that domain for each reviewer. This total score was then standardized as a percentage of the maximum possible score for that specific domain. The calculation method is as follows:

$$\frac{Obtained\ score - Minimum\ possible\ score}{Maximum\ possible\ score - Minimum\ possible\ score} * 100\%$$

The minimum possible score is calculated as: (number of questions) × (number of reviewers) × 1. The maximum possible score is calculated as: (number of questions) × (number of reviewers) × 7.

Five guidance documents were selected for the pilot scoring phase, and discussions were held to clarify scoring criteria. After completing scoring for all included documents, a meeting was held to discuss items with scores differing by more than one point. Reviewers were then given the opportunity to revise their scores or maintain their original scores, with all original and revised scores, along with reasons for modifying scores, meticulously recorded for quality control purposes.

For peer review only - http://bmjopen.bmj.com/site/about/guidelines.xhtml

Supplementary Table 6. Scores for each individual AGREE II domain items by each reviewer.

**Document**	**Domain 1**			**Domain 2**			**Domain 3**								**Domain 4**			**Domain 5**				**Domain 6**	
**Item**	**1**	**2**	**3**	**4**	**5**	**6**	**7**	**8**	**9**	**10**	**11**	**12**	**13**	**14**	**15**	**16**	**17**	**18**	**19**	**20**	**21**	**22**	**23**
OG-OG-CMA_2014																							
Rev-1	4	4	7	2	1	3	1	1	1	1	3	1	1	1	5	1	1	1	1	1	1	1	1
Rev-2	4	3	7	2	3	2	1	1	1	1	3	1	1	1	5	1	1	1	1	1	1	1	1
Rev-3	4	3	7	3	3	2	1	1	1	1	4	1	1	1	5	1	1	1	1	1	1	1	1
Rev-4	3	4	7	2	1	3	1	1	1	1	3	1	1	1	4	1	1	1	1	1	1	1	1
OG-A-CMA_2016																							
Rev-1	5	6	5	6	6	3	1	1	1	1	6	1	1	1	5	6	3	1	5	1	1	1	1
Rev-2	6	4	4	6	6	4	1	1	1	1	6	1	1	1	6	6	3	1	4	1	1	1	1
Rev-3	5	6	5	6	6	3	1	1	1	1	5	1	1	1	5	6	3	1	4	1	1	1	1
Rev-4	5	4	5	6	6	3	1	1	1	1	6	1	1	1	5	5	3	1	4	1	1	1	1
ASA_2016																							
Rev-1	7	7	7	7	6	7	7	7	6	7	7	6	6	6	6	6	6	6	6	1	1	6	6
Rev-2	7	7	7	7	7	7	7	6	6	7	7	6	7	6	6	6	6	6	6	1	1	6	6
Rev-3	7	7	7	6	6	7	7	7	6	7	6	6	6	6	6	6	5	6	6	1	1	5	6
Rev-4	7	7	7	7	7	6	7	6	6	7	7	6	7	6	6	5	6	6	6	1	1	5	6
AAGBI_2017																							
Rev-1	5	5	5	6	1	1	2	2	2	4	2	1	1	1	6	5	5	2	1	1	1	2	3
Rev-2	5	5	5	6	1	1	2	2	2	4	2	1	1	1	6	5	5	2	1	1	1	2	3
Rev-3	4	5	5	5	1	1	3	2	2	5	2	1	1	1	5	6	5	2	1	1	1	3	2
Rev-4	5	5	6	6	1	1	2	3	2	4	3	1	1	1	6	5	6	2	1	1	1	2	3
ERAS_2018 (Part 1)																							
Rev-1	6	6	6	5	4	4	6	6	6	6	6	6	4	3	5	5	5	2	5	1	2	2	6
Rev-2	5	6	5	5	4	3	6	6	5	6	6	5	3	3	5	5	4	2	5	1	2	3	5
Rev-3	5	6	6	5	4	3	6	5	6	5	6	6	4	3	5	3	5	2	5	1	2	2	6
Rev-4	6	5	6	5	3	4	6	6	6	6	5	6	3	3	6	3	5	2	5	1	2	2	5

For peer review only - http://bmjopen.bmj.com/site/about/guidelines.xhtml

ERAS_2018 (Part 2)																							
Rev-1	6	6	6	5	4	4	6	6	6	6	6	6	4	3	5	5	5	2	5	1	2	2	6
Rev-2	6	6	4	5	3	4	5	6	4	6	5	6	4	3	5	6	5	2	4	1	2	3	6
Rev-3	5	5	6	5	4	3	5	6	5	6	6	5	4	3	5	5	6	2	5	1	2	2	5
Rev-4	6	6	5	5	4	4	6	6	5	6	6	6	4	3	4	6	5	2	6	1	3	3	6
ERAS_2018 (Part 3)																							
Rev-1	6	6	6	5	4	4	6	6	6	6	6	6	4	3	5	5	5	3	5	1	2	1	6
Rev-2	6	6	5	3	4	5	6	6	5	6	5	6	3	3	5	4	5	4	5	1	3	1	5
Rev-3	5	6	6	5	4	4	5	6	6	5	6	5	4	3	6	5	6	3	6	1	2	1	6
Rev-4	6	5	6	6	4	4	6	5	6	6	6	6	3	3	5	6	5	4	5	1	3	1	5
ACOG_2019																							
Rev-1	7	6	5	5	4	5	6	6	5	5	5	4	3	5	6	6	5	4	4	1	1	6	6
Rev-2	7	5	5	4	4	4	6	5	5	4	4	3	3	4	6	6	4	5	5	1	1	5	6
Rev-3	7	6	6	5	5	5	5	6	4	5	5	4	4	5	5	6	5	4	4	1	1	6	5
Rev-4	7	6	5	5	4	5	6	5	5	5	4	3	3	5	6	5	4	4	4	1	1	5	5
SOGC_2019 (Delivery and Postpartum Care)																							
Rev-1	6	6	7	6	3	7	7	6	6	6	6	6	3	6	6	6	6	4	3	1	1	2	6
Rev-2	5	5	7	5	3	7	7	5	5	5	6	5	4	5	4	5	5	3	3	1	1	2	6
Rev-3	6	5	6	5	4	7	7	5	4	5	5	6	3	5	5	6	5	4	4	1	1	2	5
Rev-4	5	5	6	6	3	7	7	5	6	6	5	5	4	6	6	5	6	3	3	1	1	2	6
SOGC_2022 (Twin Pregnancies)																							
Rev-1	6	6	6	6	3	7	7	6	6	6	6	6	5	6	6	6	6	4	3	1	1	2	6
Rev-2	5	5	6	6	3	7	7	5	5	6	6	5	4	5	6	5	5	3	3	1	1	2	6
Rev-3	5	5	5	5	4	6	7	5	4	6	5	6	4	5	5	6	5	4	4	1	1	2	5
Rev-4	6	6	6	5	3	7	7	5	6	6	5	5	5	6	6	5	6	4	3	1	1	2	6
SOAP_2019																							
Rev-1	6	6	5	6	6	5	4	4	4	4	5	5	6	3	5	5	5	4	3	1	1	2	7

For peer review only - http://bmjopen.bmj.com/site/about/guidelines.xhtml

Rev-2	6	5	5	6	6	5	3	4	4	4	5	5	6	3	5	6	5	3	3	1	1	2	6
Rev-3	5	5	5	5	5	5	4	4	3	4	5	6	5	3	5	5	5	4	3	1	1	2	7
Rev-4	6	5	5	6	6	5	4	4	4	4	5	5	6	3	5	6	5	4	3	1	1	2	6
AA and OAA_2020																							
Rev-1	5	5	5	4	1	1	2	1	1	1	1	1	1	1	4	4	5	2	1	1	1	6	6
Rev-2	5	5	4	6	1	1	2	1	1	1	1	1	1	1	4	3	4	2	2	1	1	5	6
Rev-3	6	5	4	4	1	1	2	1	1	1	1	1	1	1	4	4	5	2	1	1	1	5	5
Rev-4	5	5	4	5	1	1	2	1	1	1	1	1	1	1	4	3	5	2	2	1	1	6	6
PROSPECT_2020																							
Rev-1	5	6	5	2	1	2	7	7	6	7	7	7	1	1	7	6	7	6	1	1	1	6	6
Rev-2	5	5	5	2	1	2	7	7	7	7	7	7	3	1	6	6	6	6	3	1	1	6	6
Rev-3	5	6	5	2	1	2	7	7	6	6	7	7	1	1	6	6	6	5	1	1	1	5	6
Rev-4	5	5	5	2	1	2	7	7	7	7	6	7	3	1	6	6	6	6	3	1	1	6	5
CHBSA_2020																							
Rev-1	5	5	5	4	3	2	1	1	1	1	4	1	1	1	5	5	5	2	1	1	1	1	1
Rev-2	5	5	5	4	2	2	1	1	1	1	4	1	1	1	5	5	4	2	1	1	1	1	1
Rev-3	5	6	5	4	3	2	1	1	1	1	3	1	1	1	4	5	5	2	1	1	1	1	1
Rev-4	5	5	5	4	2	2	1	1	1	1	3	1	1	1	5	4	4	2	1	1	1	1	1
CAA_2020 (spinal)																							
Rev-1	5	6	6	5	1	7	1	1	1	1	3	1	1	1	6	6	6	1	5	1	1	1	1
Rev-2	6	6	6	5	1	6	1	1	1	1	2	1	1	1	5	5	5	1	4	1	1	1	1
Rev-3	5	6	5	5	1	6	1	1	1	1	2	1	1	1	5	6	5	1	4	1	1	1	1
Rev-4	5	6	6	4	1	6	1	1	1	1	2	1	1	1	5	5	5	1	4	1	1	1	1
CAA_2020 (delivery analgesia)																							
Rev-1	6	6	5	5	2	4	1	1	1	1	3	1	1	1	7	6	6	1	3	1	1	1	1
Rev-2	6	6	6	6	2	5	1	1	1	1	4	1	1	1	7	6	6	1	2	1	1	1	1
Rev-3	5	6	5	5	2	4	1	1	1	1	3	1	1	1	7	6	6	1	2	1	1	1	1

For peer review only - http://bmjopen.bmj.com/site/about/guidelines.xhtml

Rev-4	6	6	5	6	2	4	1	1	1	1	4	1	1	1	7	6	6	1	2	1	1	1	1
SOAP_2020																							
Rev-1	5	5	5	6	3	2	6	5	7	6	2	3	1	1	6	6	7	5	3	1	1	1	6
Rev-2	6	6	5	7	4	2	6	6	7	6	4	4	1	1	6	6	6	6	3	1	1	1	6
Rev-3	5	5	5	6	3	2	6	5	6	5	3	4	1	1	5	6	5	6	2	1	1	1	5
Rev-4	5	6	6	7	3	2	6	6	7	6	4	4	1	1	6	5	6	6	3	1	1	1	6
NICE_2021																							
Rev-1	5	7	7	6	6	5	1	1	1	1	6	1	1	7	3	5	3	3	4	1	1	1	1
Rev-2	6	6	7	7	6	6	1	1	1	1	7	1	1	7	5	6	6	5	6	1	1	1	1
Rev-3	6	7	7	6	5	5	1	1	1	1	7	1	1	6	3	4	4	3	4	1	1	1	1
Rev-4	6	6	7	7	6	6	1	1	1	1	7	1	1	7	5	6	6	5	6	1	1	1	1
OAA_2022																							
Rev-1	5	5	4	6	1	4	5	4	1	4	4	1	1	1	3	3	5	5	3	1	1	2	4
Rev-2	5	5	6	6	1	3	6	3	2	3	4	1	1	1	5	5	5	4	2	1	1	4	5
Rev-3	5	5	5	5	1	4	6	4	1	5	3	1	1	1	4	5	5	4	3	1	1	4	4
Rev-4	6	5	6	6	1	3	6	2	2	3	4	1	1	1	5	5	5	4	2	1	1	4	5
ANNA_2023																							
Rev-1	6	6	5	6	3	5	1	1	1	2	1	1	1	3	6	6	5	5	2	1	1	6	6
Rev-2	6	6	6	6	2	6	1	1	1	2	1	1	1	2	7	7	6	5	3	1	1	6	6
Rev-3	5	5	5	5	3	6	1	1	1	2	1	1	1	4	6	6	6	5	2	1	1	6	6
Rev-4	6	5	6	6	2	6	1	1	1	2	1	1	1	2	7	7	6	5	3	1	1	5	6
RCoA_2023																							
Rev-1	7	6	6	7	3	7	6	7	7	7	7	5	5	7	6	6	7	6	6	6	5	7	7
Rev-2	6	6	7	7	4	6	5	7	7	7	7	6	6	6	7	7	7	6	6	6	4	6	6
Rev-3	7	5	6	7	4	6	6	7	7	7	7	6	5	7	7	6	7	6	6	5	5	6	6
Rev-4	6	6	7	7	4	6	5	7	7	7	7	6	6	6	7	7	7	6	5	6	5	6	6
French_2021																							

Rev-1	6	4	6	6	3	4	1	1	1	4	1	1	4	1	5	3	5	1	3	1	1	1	4
Rev-2	6	5	7	7	2	5	1	1	1	5	2	1	5	1	6	4	6	2	2	1	1	1	5
Rev-3	5	5	6	7	3	5	1	1	1	6	1	1	5	1	6	3	6	3	3	1	1	1	5
Rev-4	7	4	7	7	4	6	1	1	1	5	1	1	4	1	7	2	7	1	2	1	1	1	4

For peer review only

For peer review only - http://bmjopen.bmj.com/site/about/guidelines.xhtml

Supplementary Table 7. Domain score for each included guidance document.

Document	Domain 1, %	Domain 2, %	Domain 3, %	Domain 4, %	Domain 5, %	Domain 6, %
OG-OG-CMA_2014	62.5	20.8	6.6	20.8	0.0	0.0
OG-A-CMA_2016	66.7	68.1	11.7	61.1	13.5	0.0
ASA_2016	100.0	94.4	91.3	80.6	41.7	79.2
AAGBI_2017	66.7	26.4	18.4	73.6	4.2	25.0
ERAS_2018 (Part 1)	77.8	51.4	69.9	61.1	25.0	47.9
ERAS_2018 (Part 2)	76.4	52.8	69.4	69.4	26.0	52.1
ERAS_2018 (Part 3)	79.2	55.6	69.4	69.4	34.4	37.5
ACOG_2019	83.3	59.7	60.7	72.2	27.1	75.0
SOGC_2019 (Delivery and Postpartum Care)	79.2	70.8	73.5	73.6	19.8	47.9
SOGC_2022 (Twin Pregnancies)	76.4	69.4	76.5	76.4	20.8	47.9
SOAP_2019	72.2	75.0	56.1	69.4	19.8	54.2
AA and OAA_2020	63.9	20.8	4.1	51.4	6.3	77.1
PROSPECT_2020	69.4	11.1	75.5	86.1	24.0	79.2
CHBSA_2020	68.1	30.6	7.1	61.1	4.2	0.0
CAA_2020 (spinal)	77.8	50.0	4.6	72.2	13.5	0.0
CAA_2020 (delivery analgesia)	77.8	48.6	7.1	88.9	5.2	0.0
SOAP_2020	72.2	48.6	53.1	80.6	27.1	39.6
NICE_2021	90.3	81.9	25.5	61.1	29.2	0.0
OAA_2022	69.4	40.3	28.6	59.7	19.8	50.0
ANNA_2023	76.4	61.1	7.7	87.5	22.9	81.3
RCoA_2023	87.5	77.8	90.3	95.8	76.0	87.5
French_2021	82.35	79.66	55.56	80.0	36.0	63.64

For peer review only

Supplementary Table 8. Mean scores across reviewers for the individual AGREE II domain items.

Document	The intraclass correlation coefficient, 95% confidence interval																						
	Domain 1			Domain 2			Domain 3								Domain 4			Domain 5				Domain 6	
	1	2	3	4	5	6	7	8	9	10	11	12	13	14	15	16	17	18	19	20	21	22	23
OG-OG-CMA_2014	3.8	4.7	7.0	2.3	2.0	2.5	1.0	1.0	1.0	1.0	3.3	1.0	1.0	1.0	4.8	1.0	1.0	1.0	1.0	1.0	1.0	1.0	1.0
OG-A-CMA_2016	5.3	5.0	6.0	6.0	6.0	3.3	1.0	1.0	1.0	1.0	5.8	1.0	1.0	1.0	5.3	5.8	3.0	1.0	4.3	1.0	1.0	1.0	1.0
ASA_2016	7.0	7.0	7.0	6.8	6.5	6.8	7.0	6.5	6.0	7.0	6.8	6.0	6.5	6.0	6.0	5.8	5.8	6.0	6.0	1.0	1.0	5.5	6.0
AAGBI_2017	4.8	5.0	5.3	5.8	1.0	1.0	2.3	2.3	2.0	4.3	2.3	41.0	1.0	1.0	5.8	5.3	5.3	1.0	1.0	1.0	2.3	2.3	2.8
ERAS_2018 (Part 1)	5.5	5.8	5.8	5.0	3.8	3.5	6.0	5.8	5.8	5.8	5.8	5.8	3.5	3.0	5.3	4.0	4.8	2.0	5.0	1.0	2.0	2.3	5.5
ERAS_2018 (Part 2)	5.8	5.8	5.3	5.0	3.8	3.8	5.5	6.0	5.0	6.0	5.8	5.8	4.0	3.0	4.8	5.5	5.3	2.0	5.0	1.0	2.3	2.5	5.8
ERAS_2018 (Part 3)	5.8	5.8	5.8	4.8	4.0	4.3	5.8	5.8	5.8	5.8	5.8	5.8	3.5	3.0	5.3	5.0	5.3	3.5	5.3	1.0	2.5	1.0	5.5
ACOG_2019	7.0	5.8	5.3	4.8	4.3	4.8	5.8	5.5	4.8	4.8	4.5	3.5	3.3	4.8	5.8	5.8	4.5	4.3	4.3	1.0	1.0	5.5	5.5
SOGC_2019 (Delivery and Postpartum Care)	5.5	5.3	6.5	5.5	3.3	7.0	7.0	5.3	5.3	5.5	5.5	5.5	3.5	5.5	5.3	5.5	5.5	3.5	3.3	1.0	1.0	2.0	5.8
SOGC_2022 (Twin Pregnancies)	5.5	5.5	5.8	5.5	3.3	6.8	7.0	5.3	5.3	6.0	5.5	5.5	4.5	5.5	5.8	5.5	5.5	3.8	3.3	1.0	1.0	2.0	5.8
SOAP_2019	5.8	5.3	5.0	5.8	5.8	5.0	3.8	4.0	3.8	4.0	5.0	5.3	5.8	3.0	5.0	5.5	5.0	3.8	3.0	1.0	1.0	2.0	6.5
AA and OAA_2020	5.3	5.0	4.3	4.8	1.0	1.0	2.0	1.0	1.0	1.0	1.0	1.0	1.0	1.0	4.0	3.5	4.8	2.0	1.5	1.0	1.0	5.5	5.8
PROSPECT_2020	5.0	5.5	5.0	2.0	1.0	2.0	7.0	7.0	6.5	6.8	6.8	7.0	2.0	1.0	6.3	6.0	6.3	5.8	2.0	1.0	1.0	5.8	5.8
GHBSA_2020	5.0	5.3	5.0	4.0	2.5	2.0	1.0	1.0	1.0	1.0	3.5	1.0	1.0	1.0	4.8	4.8	4.5	2.0	1.0	1.0	1.0	1.0	1.0
CAA_2020 (spinal)	5.3	6.0	5.8	4.8	1.0	6.3	1.0	1.0	1.0	1.0	2.3	1.0	1.0	1.0	5.3	5.5	5.3	1.0	4.3	1.0	1.0	1.0	1.0
CAA_2020 (delivery analgesia)	5.8	6.0	5.3	5.5	2.0	4.3	1.0	1.0	1.0	1.0	3.5	1.0	1.0	1.0	7.0	6.0	6.0	1.0	2.3	1.0	1.0	1.0	1.0
SOAP_2020	5.3	5.5	5.3	6.5	3.3	2.0	6.0	5.5	6.8	5.8	3.3	3.8	1.0	1.0	5.8	5.8	6.0	5.8	2.8	1.0	1.0	1.0	5.8
NICE_2021	5.8	6.5	7.0	6.5	5.8	5.5	1.0	1.0	1.0	1.0	6.8	1.0	1.0	6.8	4.0	5.3	4.8	4.0	5.0	1.0	1.0	1.0	1.0
OAA_2022	5.3	5.0	5.3	5.8	1.0	3.5	5.8	3.3	1.5	3.8	3.8	1.0	1.0	1.0	4.3	4.5	5.0	4.3	2.5	1.0	1.0	3.5	4.5
ANNA_2023	5.8	5.5	5.5	5.8	2.5	5.8	1.0	1.0	1.0	2.0	1.0	1.0	1.0	2.8	6.5	6.5	5.8	5.0	2.5	1.0	1.0	5.8	6.0
RCoA_2023	6.5	5.8	6.5	7.0	3.8	6.3	5.5	7.0	7.0	7.0	7.0	5.8	5.5	6.5	6.8	6.5	7.0	6.0	5.8	5.8	4.8	6.3	6.3

For peer review only - http://bmjopen.bmj.com/site/about/guidelines.xhtml

French_2021	6.0	4.5	6.5	6.75	3.0	5.0	1.0	1.0	1.0	5.0	1.25	1.0	4.5	1.0	6.0	3.0	6.0	1.75	2.5	1.0	1.0	1.0	4.5

For peer review only - http://bmjopen.bmj.com/site/about/guidelines.xhtml

Supplementary Table 9. The intraclass correlation coefficient between reviewers for each individual AGREE II domain item.

Document	The intraclass correlation coefficient (95% confidence interval)	*P* valve
OG-OG-CMA_2014	0.987 (0.975, 0.994)	<0.001
OG-A-CMA_2016	0.993 (0.986, 0.997)	<0.001
ASA_2016	0.989 (0.980, 0.995)	<0.001
AAGBI_2017	0.990 (0.982, 0.995)	<0.001
ERAS_2018 (Part 1)	0.975 (0.952, 0.988)	<0.001
ERAS_2018 (Part 2)	0.971 (0.945, 0.986)	<0.001
ERAS_2018 (Part 3)	0.967 (0.938, 0.985)	<0.001
ACOG_2019	0.970 (0.942, 0.986)	<0.001
SOGC_2019 (Delivery and Postpartum Care)	0.974 (0.950, 0.988)	<0.001
SOGC_2022 (Twin Pregnancies)	0.976 (0.953, 0.989)	<0.001
SOAP_2019	0.986 (0.974, 0.994)	<0.001
AA and OAA_2020	0.991 (0.983, 0.996)	<0.001
PROSPECT_2020	0.990 (0.981, 0.995)	<0.001
CHBSA_2020	0.994 (0.988, 0.997)	<0.001
CAA_2020 (spinal)	0.995 (0.989, 0.998)	<0.001
CAA_2020 (delivery analgesia)	0.996 (0.993, 0.998)	<0.001
SOAP_2020	0.987 (0.972, 0.994)	<0.001
NICE_2021	0.984 (0.966, 0.993)	<0.001
OAA_2022	0.970 (0.944, 0.986)	<0.001
ANNA_2023	0.991 (0.984, 0.996)	<0.001
RCoA_2023	0.925 (0.859, 0.965)	<0.001
French_2021	0.941 (0.905, 0.986)	<0.001

For peer review only - http://bmjopen.bmj.com/site/about/guidelines.xhtml

Supplementary Table 10. Summary of recommendations for the peri-anesthetic evaluation and preparation by included guidance document.

Recommendations	Reference contents	OG-OG-CMA_2014	OG-A-CMA_2016	ASA_2016	AAGBI_2017	ERAS_2018 (Part 1)	ERAS_2018 (Part 2)	ERAS_2018 (Part 3)	ACOG_2019	SOGC_2019	SOGC_2022	SOAP_2019	AA and OAA_2020	PROSPECT_2020	CHBSA_2020	CAA_2020	CAA_2020	SOAP_2020	NICE_2021	OAA_2022	ANNA_2023	RCoA_2023	French_2021
**Peri-anesthetic Evaluation and Preparation**																							
**1. History and physical examination before providing anesthetic care**	Yes	+	+	+	NA	NG	NA	NA	+	NA	NA	NA	NA	NA	+	+	+	NG	+/-	NA	+	NG	+
1.1 Maternal health and anesthetic history	Agree	+	+	+	NA	NG	NA	NA	+	NA	NA	NA	NA	NA	+	+	+	NG	NG	NA	+/-	NG	+
1.2 Relevant obstetric history	Agree	+	+	+	NA	NG	NA	NA	NG	NA	NA	NA	NA	NA	NG	+	+	NG	NG	NA	NG	NG	NG
1.3 Baseline blood pressure measurement	Agree	+	+	+	NA	NG	NA	NA	NG	NA	NA	NA	NA	NA	+	-	+	NG	NG	NA	+/-	NG	NG
1.4 Airway examination	Agree	NG	+	+	NA	NG	NA	NA	+	NA	NA	NA	NA	NA	NG	+	+	NG	NG	NA	+	NG	NG
1.5 Heart and lung examination	Agree	NG	NG	+	NA	NG	NA	NA	+	NA	NA	NA	NA	NA	+	+	+	NG	NG	NA	+	NG	NG

For peer review only - http://bmjopen.bmj.com/site/about/guidelines.xhtml

1.6 When a neuraxial anesthetic is planned, examine the patient's back	Agree	NG	+	+	NA	NG	NA	NA	+	NA	NA	NA	NA	NA	NG	+	+	NG	NG	NA	+	NG	NG
1.7 Consultation between the obstetrician and the anesthesiologist	Agree	+	NG	+	NA	+	NA	NA	+	NA	NA	NA	NA	NA	NG	+	+	NG	NG	NA	+	NG	+
**2. Preoperative communication**	Encouraged	+	NG	+	NA	+	NA	NA	NG	NA	NA	NA	NA	NA	+	+	-	+/-	+	NA	+	+/-	+
**3. Intrapartum platelet count**	Yes	+	+	+	NA	NA	NA	NA	+	NA	NA	NA	NA	NA	NG	+	NG	NG	+	NA	NG	NG	NA
-Routine	Agree	+	+	-	NA	NA	NA	NA	+	NA	NA	NA	NA	NA	NG	NG	NG	NG	-	NA	NG	NG	NA
-Individualized	Agree	-	-	+	NA	NA	NA	NA	+	NA	NA	NA	NA	NA	NG	NG	NG	NG	+	NA	NG	NG	NA
**4. Blood type and screen**	Yes	+	+	+	NA	NA	NA	NA	NG	NA	NA	NA	NA	NA	NG	+	NG	NG	+	NA	NG	NG	NA
-Routine	Agree	+	+	-	NA	NA	NA	NA	NG	NA	NA	NA	NA	NA	NG	+	NG	NG	+	NA	NG	NG	NA
-Individualized	Agree	-	-	+	NA	NA	NA	NA	NG	NA	NA	NA	NA	NA	NG	NG	NG	NG	-	NA	NG	NG	NA
**5. Blood cross-match**	Yes	+	NG	+	NA	NA	NA	NA	NG	NA	NA	NA	NA	NA	NG	NG	NG	NG	+	NA	NG	NG	NA
-Routine	Agree	+	NG	-	NA	NA	NA	NA	NG	NA	NA	NA	NA	NA	NG	NG	NG	NG	-	NA	NG	NG	NA
-Individualized	Agree	-	NG	+	NA	NA	NA	NA	NG	NA	NA	NA	NA	NA	NG	NG	NG	NG	+	NA	NG	NG	NA
**6. Peri-anesthetic recording of fetal heart rate**	Yes	+	+	+	NA	NA	NA	NA	+	+	+	NA	NA	NA	NG	+	+	NG	NG	NA	+	NG	+
**7. Aspiration prevention**	Provided	+	NG	+	NA	+	NA	NG	NA	+	NA	NA	NA	NA	+	+/-	NG	+/-	+/-	NA	+/-	+/-	NA
7.1 Clear liquids	Up to 2 h	NG	NG	Up to 2 h	NA	Up to 2 h	NA	NG	NA	NG	NA	NA	NA	NA	Up to 2 h	+	NG	+	NG	NA	NG	+/-	NA
7.2 Solids	6-8 h	+	NG	6-8 h	NA	Light meal	NA	NG	NA	NG	NA	NA	NA	NA	6 h	+	NG	+	NG	NA	NG	NG	NA

For peer review only - http://bmjopen.bmj.com/site/about/guidelines.xhtml

						to 6 h																	
7.3 Antacids (surgical procedures-cesarean delivery or postpartum tubal ligation)	Agree	NG	NG	+	NA	+	NA	+	NA	NG	NA	NA	NA	NA	+	+	NG	NG	+	NA	NG	+	NA
7.4 H_2-receptor antagonists (surgical procedures)	Agree	NG	NG	+	NA	+	NA	+	NA	NG	NA	NA	NA	NA	+	+	NG	NG	+	NA	NG	NG	NA
7.5 Metoclopramide (surgical procedures)	Agree	NG	NG	+	NA	NG	NA	20 mg	NA	NG	NA	NA	NA	NA	NG	+	NG	NG	NG	NA	NG	NG	NA
**8. Preoperative heparin or low-molecular-weight heparin**	Yes	NA	+	NG	NA	NA	NA	NA	+	NA	NA	NA	NA	NA	NA	NG	+/-	NG	NG	NA	+/-	+/-	NA
8.1 Prophylactic subcutaneous unfractionated heparin (<5,000 units twice daily)	Provided	NA	NG	NG	NA	NA	NA	NA	+	NA	NA	NA	NA	NA	NA	NG	NG	NG	NG	NA	NG	NG	NA
-Contraindication to neuraxial techniques	No	NA	NG	NG	NA	NA	NA	NA	+	NA	NA	NA	NA	NA	NA	NG	NG	NG	NG	NA	NG	NG	NA
-Catheter placement or removal since last dose	4-6 hours	NA	NG	NG	NA	NA	NA	NA	4-6 h	NA	NA	NA	NA	NA	NA	NG	NG	NG	NG	NA	NG	NG	NA
8.2 Intermediate unfractionated heparin (75,000-100,000 units)	Provided	NA	NG	NG	NA	NA	NA	NA	+	NA	NA	NA	NA	NA	NA	NG	NG	NG	NG	NA	NG	NG	NA
-Catheter placement or removal since last dose	> 12 hours	NA	NG	NG	NA	NA	NA	NA	> 12 hours	NA	NA	NA	NA	NA	NA	NG	NG	NG	NG	NA	NG	NG	NA

For peer review only

8.3 High-dose unfractionated heparin (total daily dose greater than 20,000 units)	Provided	NA	NG	NG	NA	NA	NA	NA	+	NA	NA	NA	NA	NA	NA	NG	NG	NG	NG	NA	NG	NG	NA
-Catheter placement or removal since last dose	> 24 hours	NA	NG	NG	NA	NA	NA	NA	> 24 hours	NA	NA	NA	NA	NA	NA	NG	NG	NG	NG	NA	NG	NG	NA
-With the activated partial thromboplastin time being within normal range	Yes	NA	NG	NG	NA	NA	NA	NA	+	NA	NA	NA	NA	NA	NA	NG	NG	NG	NG	NA	NG	NG	NA

+: consistent the reference with content;

+/-: partial1y consistent the reference with content;

-: inconsistent the reference with content;

NA: not applicable;

NG: not given.

For peer review only

Supplementary Table 11. Summary of recommendations for the anesthetic care for labor and vaginal delivery by included guidance document.

Recommendations	Reference contents	OG-OG-CMA_2014	OG-A-CMA_2016	ASA_2016	AAGBI_2017	ERAS_2018 (Part 1)	ERAS_2018 (Part 2)	ERAS_2018 (Part 3)	ACOG_2019	SOGC_2019	SOGC_2022	SOAP_2019	AA and OAA_2020	PROSPECT_2020	CHBSA_2020	CAA_2020	CAA_2020	SOAP_2020	NICE_2021	OAA_2022	ANNA_2023	RCoA_2023	French_2021
**Anesthetic Care for Labor and Vaginal Delivery**																							
**1. Equipment, facilities, and support personnel**	Agree	NA	+	+	NA	NA	NA	NA	+	NA	NA	NA	NA	NA	+	NA	+	NA	NA	NA	+	+	NA
**2. Timing of neuraxial analgesia and outcome of labor**	Provided	NA	+	+	NA	NA	NA	NA	NG	NA	NA	NA	NA	NA	NG	NA	+	NA	NA	NA	NG	NG	NA
2.1 Less 5 cm dilation	Agree	NA	+/-	+	NA	NA	NA	NA	NG	NA	NA	NA	NA	NA	NG	NA	+/-	NA	NA	NA	NG	NG	NA
2.2 Offer neuraxial analgesia on an individualized basis	Agree	NA	+	+	NA	NA	NA	NA	NG	NA	NA	NA	NA	NA	NG	NA	+	NA	NA	NA	NG	NG	NA
**3. Neuraxial analgesia and trial of labor after prior cesarean delivery**	Agree	NA	NG	+	NA	NA	NA	NA	NG	NA	NA	NA	NA	NA	NG	NA	NG	NA	NA	NA	NG	NG	NA
**4. Continuous infusion epidural analgesia**	Provided	NA	+	+	NA	NA	NA	NA	+	NA	+/-	NA	NA	NA	+	NA	+	NA	NA	NA	+	NG	NA
4.1 Using local anesthetic	Agree	NA	+	+	NA	NA	NA	NA	+	NA	NG	NA	NA	NA	+	NA	+	NA	NA	NA	+	NG	NA
4.2 Add opioid	Agree	NA	+	+	NA	NA	NA	NA	+	NA	NG	NA	NA	NA	NG	NA	+	NA	NA	NA	+/-	NG	NA
4.3 Platelet concentration corresponding to	Less than 70*10^9/L	NA	NG	NG	NA	NA	NA	NA	+	NA	NG	NA	NA	NA	NG	NA	NG	NA	NA	NA	+	NG	NA

For peer review only - http://bmjopen.bmj.com/site/about/guidelines.xhtml

contraindications to puncture																							
4.4 Puncture location	L_{2-3} or L_{3-4}	NA	+	NG	NA	NA	NA	NA	NG	NA	NG	NA	NA	NA	NG	NA	+	NA	NA	NA	NG	NG	NA
4.5 Analgesic concentrations	Provided	NA	+	NG	NA	NA	NA	NA	NG	NA	NG	NA	NA	NA	NG	NA	+	NA	NA	NA	+	NG	NA
-Bupivacaine	Provided	NA	+	NG	NA	NA	NA	NA	NG	NA	NG	NA	NA	NA	NG	NA	+	NA	NA	NA	+	NG	NA
--Loading dose	0.04-0.125%	NA	+	NG	NA	NA	NA	NA	NG	NA	NG	NA	NA	NA	NG	NA	+	NA	NA	NA	0.0625-0.125%	NG	NA
--Maintenance dose	0.05-0.125%	NA	+	NG	NA	NA	NA	NA	NG	NA	NG	NA	NA	NA	NG	NA	+	NA	NA	NA	0.0625-0.125%	NG	NA
-Ropivacaine	Provided	NA	+	NG	NA	NA	NA	NA	NG	NA	NG	NA	NA	NA	NG	NA	+	NA	NA	NA	+	NG	NA
--Loading dose	0.0625-0.15%	NA	+	NG	NA	NA	NA	NA	NG	NA	NG	NA	NA	NA	NG	NA	+	NA	NA	NA	0.08-0.2%	NG	NA
--Maintenance dose	0.0625-0.125%	NA	+	NG	NA	NA	NA	NA	NG	NA	NG	NA	NA	NA	NG	NA	+	NA	NA	NA	0.08-0.2%	NG	NA
-Levobupivacaine	Provided	NA	NG	NG	NA	NA	NA	NA	NG	NA	NG	NA	NA	NA	NG	NA	+	NA	NA	NA	NG	NG	NA
--Loading dose	0.04-0.125%	NA	NG	NG	NA	NA	NA	NA	NG	NA	NG	NA	NA	NA	NG	NA	+	NA	NA	NA	NG	NG	NA
--Maintenance dose	0.05-0.125%	NA	NG	NG	NA	NA	NA	NA	NG	NA	NG	NA	NA	NA	NG	NA	+	NA	NA	NA	NG	NG	NA
4.6 Opioids concentrations	Provided	NA	+	NG	NA	NA	NA	NA	NG	NA	NG	NA	NA	NA	NG	NA	+	NA	NA	NA	+/-	NG	NA
-Fentanyl	Provided	NA	+	NG	NA	NA	NA	NA	NG	NA	NG	NA	NA	NA	NG	NA	+	NA	NA	NA	NG	NG	NA
--Loading dose	0.5-2 $\mu g.ml^{-1}$	NA	1-2	NG	NA	NA	NA	NA	NG	NA	NG	NA	NA	NA	NG	NA	+	NA	NA	NA	NG	NG	NA
--Maintenance dose	1-2 $\mu g.ml^{-1}$	NA	+	NG	NA	NA	NA	NA	NG	NA	NG	NA	NA	NA	NG	NA	+	NA	NA	NA	NG	NG	NA
-Sufentanyl	Provided	NA	+	NG	NA	NA	NA	NA	NG	NA	NG	NA	NA	NA	NG	NA	+	NA	NA	NA	NG	NG	NA

For peer review only - http://bmjopen.bmj.com/site/about/guidelines.xhtml

--Loading dose	0.2-0.6 µg.ml^{-1}	NA	0.4-0.6	NG	NA	NA	NA	NA	NG	NA	NG	NA	NA	NA	NG	NA	+	NA	NA	NA	NG	NG	NA
--Maintenance dose	0.3-0.6 µg.ml^{-1}	NA	0.4-0.6	NG	NA	NA	NA	NA	NG	NA	NG	NA	NA	NA	NG	NA	+	NA	NA	NA	NG	NG	NA
**5. Combined spinal–epidural analgesia**	Provided	NA	+	+	NA	NA	NA	NA	+	NA	NA	NA	NA	NA	NG	NA	+	NA	NA	NA	+/-	NG	NA
5.1 Using local anesthetic	Agree	NA	+	NG	NA	NA	NA	NA	+	NA	NA	NA	NA	NA	NG	NA	+	NA	NA	NA	+	NG	NA
5.2 Add opioid	Agree	NA	+	NG	NA	NA	NA	NA	+	NA	NA	NA	NA	NA	NG	NA	+	NA	NA	NA	+	NG	NA
5.3 Platelet concentration corresponding to contraindications to puncture	Less than 70*10^{9}/L	NA	NG	NG	NA	NA	NA	NA	+	NA	NA	NA	NA	NA	NG	NA	+	NA	NA	NA	+	NG	NA
5.4 Puncture location	L_{2-3} or L_{3-4}	NA	+	NG	NA	NA	NA	NA	NG	NA	NA	NA	NA	NA	NG	NA	+/-	NA	NA	NA	NG	NG	NA
5.5 Epidural anesthetics (consistent with 4.5 and 4.6)	Provided	NA	+	NG	NA	NA	NA	NA	NG	NA	NA	NA	NA	NA	NG	NA	+	NA	NA	NA	+/-	NG	NA
5.6 Spinal anesthetics	Provided	NA	+	NG	NA	NA	NA	NA	+	NA	NA	NA	NA	NA	NG	NA	+	NA	NA	NA	NG	NG	NA
-Protocol 1	Provided	NA	+	NG	NA	NA	NA	NA	+	NA	NA	NA	NA	NA	NG	NA	+	NA	NA	NA	NG	NG	NA
--Single sufentanyl	2.5-7 µg	NA	+	NG	NA	NA	NA	NA	+/-	NA	NA	NA	NA	NA	NG	NA	+	NA	NA	NA	NG	NG	NA
--Single ropivacaine	2.3-3 mg	NA	2.5-3	NG	NA	NA	NA	NA	+/-	NA	NA	NA	NA	NA	NG	NA	+	NA	NA	NA	NG	NG	NA
--Combined sufentanyl and ropivacaine	2.5 µg / 2.5 mg	NA	+	NG	NA	NA	NA	NA	+/-	NA	NA	NA	NA	NA	NG	NA	+	NA	NA	NA	Sufentanyl 5-10 µg	NG	NA
-Protocol 2	Provided	NA	+	NG	NA	NA	NA	NA	+/-	NA	NA	NA	NA	NA	NG	NA	+	NA	NA	NA	NG	NG	NA
--Single fentanyl	15-25 µg	NA	+	NG	NA	NA	NA	NA	+/-	NA	NA	NA	NA	NA	NG	NA	+	NA	NA	NA	NG	NG	NA
--Single bupivacaine	2.0-2.5 mg	NA	+	NG	NA	NA	NA	NA	+/-	NA	NA	NA	NA	NA	NG	NA	+	NA	NA	NA	NG	NG	NA
--Combined fentanyl and bupivacaine	2.5 µg / 2.0 mg	NA	+	NG	NA	NA	NA	NA	+/-	NA	NA	NA	NA	NA	NG	NA	+	NA	NA	NA	Fentanyl 50-100 µg	NG	NA

For peer review only - http://bmjopen.bmj.com/site/about/guidelines.xhtml

**6. Single-injection spinal**	Provided	NA	NG	+	NA	NA	NA	NA	+	NA	NA	NA	NA	NA	NG	NA	+	NA	NA	NA	+	NG	NA
6.1 Using local anesthetic	Agree	NA	NG	+	NA	NA	NA	NA	+	NA	NA	NA	NA	NA	NG	NA	+	NA	NA	NA	NG	NG	NA
6.2 Add opioid	Agree	NA	NG	+	NA	NA	NA	NA	+	NA	NA	NA	NA	NA	NG	NA	+	NA	NA	NA	NG	NG	NA
6.3 Platelet concentration corresponding to contraindications to puncture	Less than 70*10^9/L	NA	NG	NG	NA	NA	NA	NA	+	NA	NA	NA	NA	NA	NG	NA	NG	NA	NA	NA	+	NG	NA
6.4 Puncture location	L_{3-4}	NA	NG	NG	NA	NA	NA	NA	NG	NA	NA	NA	NA	NA	NG	NA	NG	NA	NA	NA	NG	NG	NA
6.6 Analgesic concentrations (consistent with 5.6)	Agree	NA	NG	NG	NA	NA	NA	NA	NG	NA	NA	NA	NA	NA	NG	NA	+	NA	NA	NA	NG	NG	NA
6.7 Spinal needles	Provided	NA	NG	+	NA	NA	NA	NA	NG	NA	NA	NA	NA	NA	NG	NA	NG	NA	NA	NA	+	NG	NA
-Cutting-bevel	Disagree	NA	NG	+	NA	NA	NA	NA	NG	NA	NA	NA	NA	NA	NG	NA	NG	NA	NA	NA	+	NG	NA
-Pencil-point	Agree	NA	NG	+	NA	NA	NA	NA	NG	NA	NA	NA	NA	NA	NG	NA	NG	NA	NA	NA	+	NG	NA
**7. Patient-controlled epidural analgesia (for labor analgesia)**	Agree	NA	+	+	NA	NA	NA	NA	NG	NA	NA	NA	NA	NA	+	NA	+	NA	NA	NA	+	NG	NA

+: consistent the reference with content;

+/-: partial1y consistent the reference with content;

-: inconsistent the reference with content;

NA: not applicable;

NG: not given.

Supplementary Table 12. Summary of recommendations for the anesthetic care for cesarean delivery by included guidance document.

For peer review only - http://bmjopen.bmj.com/site/about/guidelines.xhtml

Recommendations	Reference contents	OG-OG-CMA_2014	OG-A-CMA_2016	ASA_2016	AAGBI_2017	ERAS_2018 (Part 1)	ERAS_2018 (Part 2)	ERAS_2018 (Part 3)	ACOG_2019	SOGC_2019	SOGC_2022	SOAP_2019	AA and OAA_2020	PROSPECT_2020	CHBSA_2020	CAA_2020	CAA_2020	SOAP_2020	NICE_2021	OAA_2022	ANNA_2023	RCoA_2023	French_2021
**Anesthetic Care for Cesarean Delivery**																							
**1. Equipment, facilities, and support personnel**	Agree	+	NA	+	NA	NA	NG	NA	+	+	NA	NA	NA	NA	+	NG	NA	NA	NA	NA	+	+	NA
**2. Continuous infusion epidural analgesia**	Provided	+	NA	+	NA	NA	+/-	NA	+	+/-	NA	NA	NA	NA	+	+	NA	NA	NA	NA	+	NG	NA
2.1 Using local anesthetic	Agree	NG	NA	NG	NA	NA	NG	NA	+	NG	NA	NA	NA	NA	+	+	NA	NA	NA	NA	+	NG	NA
2.2 Add opioid	Agree	NG	NA	NG	NA	NA	NG	NA	+	NG	NA	NA	NA	NA	NG	+	NA	NA	NA	NA	+	NG	NA
2.3 Platelet concentration corresponding to contraindications to puncture	Less than 70*10^9/L	NG	NA	NG	NA	NA	NG	NA	+	NG	NA	NA	NA	NA	NG	+	NA	NA	NA	NA	+	NG	NA
2.4 Puncture location	L_{1-2} or L_{2-3}	NG	NA	NG	NA	NA	NG	NA	NG	NG	NA	NA	NA	NA	NG	+	NA	NA	NA	NA	NG	NG	NA
2.5 Recommend level of block	T_{6-4}	NG	NA	NG	NA	NA	NG	NA	T4	NG	NA	NA	NA	NA	NG	+	NA	NA	NA	NA	NG	NG	NA
2.6 Analgesic concentrations	Provided	NG	NA	NG	NA	NA	NG	NA	NG	NG	NA	NA	NA	NA	NG	+	NA	NA	NA	NA	+/-	NG	NA
-Lidocaine	1.5%-2%	NG	NA	NG	NA	NA	NG	NA	NG	NG	NA	NA	NA	NA	NG	+	NA	NA	NA	NA	NG	NG	NA
-Bupivacaine	0.50%	NG	NA	NG	NA	NA	NG	NA	NG	NG	NA	NA	NA	NA	NG	+	NA	NA	NA	NA	0.0625-0.125%	NG	NA

For peer review only

-Ropivacaine	0.5%-0.75%	NG	NA	NG	NA	NA	NG	NA	NG	NG	NA	NA	NA	NA	NG	+	NA	NA	NA	NA	0.08-0.2%	NG	NA
-Levobupivacaine	0.5%-0.75%	NG	NA	NG	NA	NA	NG	NA	NG	NG	NA	NA	NA	NA	NG	+	NA	NA	NA	NA	NG	NG	NA
-Chloroprocaine	3%	NG	NA	NG	NA	NA	NG	NA	NG	NG	NA	NA	NA	NA	NG	+	NA	NA	NA	NA	NG	NG	NA
2.7 Prevention of local anesthetic poisoning	Provided	NG	NA	NG	NA	NA	NG	NA	+	NG	NA	NA	NA	NA	NG	+	NA	NA	NA	NA	NA	NG	NA
-Withdrawal before injection	Agree	NG	NA	NG	NA	NA	NG	NA	NG	NG	NA	NA	NA	NA	NG	+	NA	NA	NA	NA	NA	NG	NA
-Administration of test dose	Agree	NG	NA	NG	NA	NA	NG	NA	NG	NG	NA	NA	NA	NA	NG	+	NA	NA	NA	NA	NA	NG	NA
-Addition of epinephrine	Agree	NG	NA	NG	NA	NA	NG	NA	+	NG	NA	NA	NA	NA	NG	+	NA	NA	NA	NA	NA	NG	NA
**3. Single-injection spinal**	Provided	NA	NA	+	+/-	NA	+/-	NA	+	+/-	NA	NA	NA	NA	+	+	NA	NA	NA	NA	+	NG	+
3.1 Using local anesthetic	Agree	NA	NA	NG	NG	NA	NG	NA	+	NG	NA	NA	NA	NA	+	+	NA	NA	NA	NA	+	NG	+
3.2 Add opioid	Agree	NA	NA	NG	NG	NA	NG	NA	+	NG	NA	NA	NA	NA	NG	+	NA	NA	NA	NA	+	NG	+
3.3 Platelet concentration corresponding to contraindications to puncture	Less than $70*10^9$/L	NA	NA	NG	NG	NA	NG	NA	+	NG	NA	NA	NA	NA	NG	+	NA	NA	NA	NA	+	NG	NG
3.4 Puncture location	L_{3-4}	NA	NA	NG	NG	NA	NG	NA	NG	NG	NA	NA	NA	NA	NG	+	NA	NA	NA	NA	NG	NG	NG
3.5 Recommend level of block	T_{6-4}	NA	NA	NG	NG	NA	NG	NA	NG	NG	NA	NA	NA	NA	NG	+	NA	NA	NA	NA	NG	NG	NG
3.6 Analgesic dose	Provided	NA	NA	NG	NG	NA	NG	NA	NG	NG	NA	NA	NA	NA	NG	+	NA	NA	NA	NA	NG	NG	+
-Bupivacaine	5-15 mg	NA	NA	NG	NG	NA	NG	NA	NG	NG	NA	NA	NA	NA	NG	+	NA	NA	NA	NA	NG	NG	+
-Ropivacaine	10-20 mg	NA	NA	NG	NG	NA	NG	NA	NG	NG	NA	NA	NA	NA	NG	+	NA	NA	NA	NA	NG	NG	NG
3.7 Spinal needles	Provided	NA	NA	+	NG	NA	NG	NA	NG	NG	NA	NA	NA	NA	NG	+	NA	NA	NA	NA	+	NG	NG
-Cutting-bevel	Disagree	NA	NA	+	NG	NA	NG	NA	NG	NG	NA	NA	NA	NA	NG	+	NA	NA	NA	NA	+	NG	NG
-Pencil-point	Agree	NA	NA	+	NG	NA	NG	NA	NG	NG	NA	NA	NA	NA	NG	+	NA	NA	NA	NA	+	NG	NG

For peer review only - http://bmjopen.bmj.com/site/about/guidelines.xhtml

**4. Combined spinal–epidural analgesia**	Provided	+	NA	+	+	NA	+/-	NA	+	+	NA	NA	NA	NA	+	+	NA	NA	NA	NA	+/-	NG	+
4.1 Puncture location	Provided	NG	NA	NG	NG	NA	NG	NA	NG	NG	NA	NA	NA	NA	NG	+	NA	NA	NA	NA	+/-	NG	NG
-Single space technique	L_{3-4}	NG	NA	NG	NG	NA	NG	NA	NG	NG	NA	NA	NA	NA	NG	+	NA	NA	NA	NA	+/-	NG	NG
-Double space technique	T_{12}-L_2/L_{3-5}	NG	NA	NG	NG	NA	NG	NA	NG	NG	NA	NA	NA	NA	NG	+	NA	NA	NA	NA	+/-	NG	NG
4.2 Anesthetics (consistent with spinal or epidural)	Agree	NG	NA	NG	NG	NA	NG	NA	+	NG	NA	NA	NA	NA	NG	+	NA	NA	NA	NA	+/-	NG	+/-
**5. General anesthesia**	Provided	+	NA	+	NA	NA	NA	NA	+	NA	NA	NA	NA	NA	+	+	NA	NA	+	NA	+	+/-	NA
5.1 General anesthesia selected condition	Provided	NG	NA	+	NA	NA	NA	NA	+	NA	NA	NA	NA	NA	NG	NG	NA	NA	NG	NA	+	NG	NA
-Profound fetal bradycardia	Agree	NG	NA	+	NA	NA	NA	NA	NG	NA	NA	NA	NA	NA	NG	NG	NA	NA	NG	NA	NG	NG	NA
-Ruptured uterus	Agree	NG	NA	+	NA	NA	NA	NA	NG	NA	NA	NA	NA	NA	NG	NG	NA	NA	NG	NA	NG	NG	NA
-Severe Hemorrhage	Agree	NG	NA	+	NA	NA	NA	NA	NG	NA	NA	NA	NA	NA	NG	NG	NA	NA	NG	NA	NG	NG	NA
-Severe placental abruption	Agree	NG	NA	+	NA	NA	NA	NA	NG	NA	NA	NA	NA	NA	NG	NG	NA	NA	NG	NA	NG	NG	NA
-Umbilical cord prolapse	Agree	NG	NA	+	NA	NA	NA	NA	NG	NA	NA	NA	NA	NA	NG	NG	NA	NA	NG	NA	NG	NG	NA
-Preterm footling breech	Agree	NG	NA	+	NA	NA	NA	NA	NG	NA	NA	NA	NA	NA	NG	NG	NA	NA	NG	NA	NG	NG	NA
-Inability to place neuraxial anesthesia	Agree	NG	NA	NG	NA	NA	NA	NA	+	NA	NA	NA	NA	NA	NG	NG	NA	NA	NG	NA	+	NG	NA
5.2 Intravenous anesthetics recommendation	Provided	NG	NA	NG	NA	NA	NA	NA	+	NA	NA	NA	NA	NA	NG	+	NA	NA	NG	NA	NG	NG	NA

For peer review only - http://bmjopen.bmj.com/site/about/guidelines.xhtml

-Thiopental sodium	4-5 mg.kg^{-1}	NG	NA	NG	NA	NA	NA	NA	NG	NA	NA	NA	NA	NA	NG	+	NA	NA	NG	NA	NG	NG	NA
-Propofol	1.5-2.5 mg.kg^{-1}	NG	NA	NG	NA	NA	NA	NA	+	NA	NA	NA	NA	NA	NG	+	NA	NA	NG	NA	NG	NG	NA
-Etomidate	0.2-0.3 mg.kg^{-1}	NG	NA	NG	NA	NA	NA	NA	NG	NA	NA	NA	NA	NA	NG	+	NA	NA	NG	NA	NG	NG	NA
-Ketamine	0.5~1 mg.kg^{-1}	NG	NA	NG	NA	NA	NA	NA	+	NA	NA	NA	NA	NA	NG	+	NA	NA	NG	NA	NG	NG	NA
5.3 Opioids analgesic recommendation	Provided	NG	NA	NG	NA	NA	NA	NA	NG	NA	NA	NA	NA	NA	NG	+	NA	NA	NG	NA	NG	NG	NA
-Fentanyl	2-5 µg.kg^{-1}	NG	NA	NG	NA	NA	NA	NA	NG	NA	NA	NA	NA	NA	NG	+	NA	NA	NG	NA	NG	NG	NA
-Sufentanyl	0.2~0.5 µg.kg^{-1}	NG	NA	NG	NA	NA	NA	NA	NG	NA	NA	NA	NA	NA	NG	+	NA	NA	NG	NA	NG	NG	NA
-Remifentanyl	0.5-1 µg.kg^{-1} for IV	NG	NA	NG	NA	NA	NA	NA	NG	NA	NA	NA	NA	NA	NG	+	NA	NG	NG	NA	NG	NG	NA
-Remifentanyl	4 ng.kg^{-1} for target	NG	NA	NG	NA	NA	NA	NA	NG	NA	NA	NA	NA	NA	NG	+	NA	NG	NG	NA	NG	NG	NA
5.4 Muscle relaxants recommendation	Provided	NG	NA	NG	NA	NA	NA	NA	+	NA	NA	NA	NA	NA	NG	+	NA	NG	NG	NA	NG	NG	NA
-Succinylcholine	1-1.5 mg.kg^{-1}	NG	NA	NG	NA	NA	NA	NA	NG	NA	NA	NA	NA	NA	NG	+	NA	NG	NG	NA	NG	NG	NA
-Rocuronium	0.6-1.2 mg.kg^{-1}	NG	NA	NG	NA	NA	NA	NA	NG	NA	NA	NA	NA	NA	NG	+	NA	NG	NG	NA	NG	NG	NA
5.5 Induction-delivery interval	within 10 min	NG	NA	NG	NA	NA	NA	NA	NG	NA	NA	NA	NA	NA	NG	+	NA	NG	NG	NA	NG	NG	NA

+: consistent the reference with content;

+/-: partial1y consistent the reference with content;

-: inconsistent the reference with content;

NA: not applicable;

For peer review only

NG: not given.

Supplementary Table 13. Summary of recommendations for the monitoring and management of obstetric and anesthetic or intra- or postoperative pain management by included guidance document.

		OG-OG-CMA_2014	OG-A-CMA_2016	ASA_2016	AAGBI_2017	ERAS_2018 (Part 1)	ERAS_2018 (Part 2)	ERAS_2018 (Part 3)	ACOG_2019	SOGC_2019	SOGC_2022	SOAP_2019	AA and OAA_2020	PROSPECT_2020	CHBSA_2020	CAA_2020	CAA_2020	SOAP_2020	NICE_2021	OAA_2022	ANNA_2023	RCoA_2023	French_2021
**Recommendations**	**Reference contents**																						
**Neurological Monitoring Associated with Obstetric Neuraxial Block**																							
**1. Screening method (During the recovery phase) - Straight-leg raising**	Agree	NA	NA	NA	NA	NA	NA	NA	NA	NA	NA	NA	+	NA	NA	NA	NA	NA	NA	NA	NA	NA	NA
**2. Alerts incidences-unable to straight-leg raise at 4 h from the last dose**	Agree	NA	NA	NA	NA	NA	NA	NA	NA	NA	NA	NA	+	NA	NA	NA	NA	NA	NA	NA	NA	NA	NA

For peer review only

For peer review only - http://bmjopen.bmj.com/site/about/guidelines.xhtml

**Respiratory Depression Monitoring with Neuraxial Block With Opioids**																							
**1. Frequency and modality of respiratory monitoring should be based on:**	Provided	+	NA	NA	NA	NA	NA	NA	NA	NA	NA	+	NA	NA	NA	NA	NA	NA	+/-	NA	+/-	NA	NA
1.1 Low risk	Provided	NG	NA	NA	NA	NA	NA	NA	NA	NA	NA	+	NA	NA	NA	NA	NA	NA	+/-	NA	NG	NA	NA
-Neuraxial morphine dose (intrathecal ≤ 0.15 mg or epidural ≤ 3 mg)	Agree	NG	NA	NA	NA	NA	NA	NA	NA	NA	NA	+	NA	NA	NA	NA	NA	NA	NG	NA	NG	NA	NA
1.2 High risk	Provided	NG	NA	NA	NA	NA	NA	NA	NA	NA	NA	+	NA	NA	NA	NA	NA	NA	+/-	NA	NG	NA	NA
Cardiopulmonary/ neurological comorbidity	Agree	NG	NA	NA	NA	NA	NA	NA	NA	NA	NA	+	NA	NA	NA	NA	NA	NA	NG	NA	NG	NA	NA
-Obesity (body mass index ≥ 40 kg/m²)	Agree	NG	NA	NA	NA	NA	NA	NA	NA	NA	NA	+	NA	NA	NA	NA	NA	NA	NG	NA	NG	NA	NA
-Known or suspected obstructive sleep apnea	Agree	NG	NA	NA	NA	NA	NA	NA	NA	NA	NA	+	NA	NA	NA	NA	NA	NA	NG	NA	NG	NA	NA

For peer review only

-Chronic opioid use or abuse	Agree	NG	NA	NA	NA	NA	NA	NA	NA	NA	NA	+	NA	NA	NA	NA	NA	NA	NG	NA	NG	NA	NA
-Hypertension	Agree	NG	NA	NA	NA	NA	NA	NA	NA	NA	NA	+	NA	NA	NA	NA	NA	NA	NG	NA	NG	NA	NA
-Magnesium administration	Agree	NG	NA	NA	NA	NA	NA	NA	NA	NA	NA	+	NA	NA	NA	NA	NA	NA	NG	NA	NG	NA	NA
-Neuraxial morphine dose (intrathecal > 0.15 mg or epidural > 3 mg)	Agree	NG	NA	NA	NA	NA	NA	NA	NA	NA	NA	+	NA	NA	NA	NA	NA	NA	NG	NA	NG	NA	NA
1.3 Respiratory monitoring frequency and duration	Provided	NG	NA	NA	NA	NA	NA	NA	NA	NA	NA	+	NA	NA	NA	NA	NA	NA	+/-	NA	NG	NA	NA
-Low risk	Provided	NG	NA	NA	NA	NA	NA	NA	NA	NA	NA	+	NA	NA	NA	NA	NA	NA	+/-	NA	NG	NA	NA
--Routine postoperative vital sign monitoring	Agree	NG	NA	NA	NA	NA	NA	NA	NA	NA	NA	+	NA	NA	NA	NA	NA	NA	+	NA	NG	NA	NA
-- q2h clinical assessment for 12 h	Agree	+/-	NA	NA	NA	NA	NA	NA	NA	NA	NA	+	NA	NA	NA	NA	NA	NA	+	NA	NG	NA	NA
-High risk	Provided	NG	NA	NA	NA	NA	NA	NA	NA		NA	+		NA	NA	NA	NA	NA	+	NA	NG	NA	NA
-- q1h clinical assessment for first 12 h	Agree	NG	NA	NA	NA	NA	NA	NA	NA	NA	NA	+	NA	NA	NA	NA	NA	NA	half hour for 2 hours	NA	NG	NA	NA
-- q2h clinical assessment for first 12-24 h	Agree	+/-	NA	NA	NA	NA	NA	NA	NA	NA	NA	+	NA	NA	NA	NA	NA	NA	NG	NA	NG	NA	NA

For peer review only - http://bmjopen.bmj.com/site/about/guidelines.xhtml

--Consider additional monitoring modalities (e.g., pulse oximetry, capnography)	Agree	NG	NA	NA	NA	NA	NA	NA	NA	NA	NA	+	NA	NA	NA	NA	NA	NA	+/-	NA	NG	NA	NA
**Management of Intra- or Postoperative Pain**																							
**1. Implement strategies to minimize systemic opioid utilization**	Provided	NA	NA	NA	NA	NA	NA	+	+	NA	NA	+	NA	+	+	NA	NA	+	+	+	+/-	NA	NA
**2. Neuraxial adjuvant drugs**	Provided	NA	NA	NA	NA	NA	NA	+	+	NA	NA	+	NA	+	+	NA	NA	+	+	NG	+	NA	NA
-Spinal anesthesia	Provided	NA	NA	NA	NA	NA	NA	NG	+	NA	NA	+	NA	+	+	NA	NA	+	+	NG	+	NA	NA
--Morphine	50-150 µg	NA	NA	NA	NA	NA	NA	NG	+	NA	NA	50-150 µg	NA	+	+	NA	NA	50-150 µg	NG	NG	150-500µg	NA	NA
-Hydromorphone	75-150 µg	NA	NA	NA	NA	NA	NA	NG	NG	NA	NA	NG	NA	NG	NG	NA	NA	NG	NG	NG	+	NA	NA
-Epidural or combined spinal-epidural technique.	Provided	NA	NA	NA	NA	NA	NA	NG	+	NA	NA	+	NA	+	NG	NA	NA	+	+	NG	+	NA	NA
--Morphine	1-3 mg	NA	NA	NA	NA	NA	NA	NG	+	NA	NA	1-3 mg	NA	+	NG	NA	NA	1~3 mg	NG	NG	1-3mg	NA	NA

For peer review only

For peer review only - http://bmjopen.bmj.com/site/about/guidelines.xhtml

--Diamorphine	2-3 mg	NA	NA	NA	NA	NA	NA	NG	NG	NA	NA	NG	NA	+	NG	NA	NA	NG	2.5-5 mg	NG	NG	NA	NA
**3. Others analgesic (after delivery)**	Provided	NA	NA	NA	NA	NA	NA	+	+	NA	NA	+	NA	+	+	NA	NA	+	+	NG	+	NA	NA
-Non-steroidal anti-inflammatory drug (e.g., paracetamol)	Agree	NA	NA	NA	NA	NA	NA	+	+	NA	NA	+	NA	+	+	NA	NA	+	+	NG	+	NA	NA
-A single dose of intravenous dexamethasone	Agree	NA	NA	NA	NA	NA	NA	NG	NG	NA	NA	NG	NA	+	NG	NA	NA	NG	NG	NG	+	NA	NA
**4. Local anesthetic infiltration**	Agree	NA	NA	NA	NA	NA	NA	+	+	NA	NA	NG	NA	+	+	NA	NA	+	NG	NG	+	NA	NA
**5. Continuous wound local anesthetic infusion**	Agree	NA	NA	NA	NA	NA	NA	NG	+	NA	NA	NG	NA	+	+	NA	NA	NG	NG	NG	NG	NA	NA
**6. Fascial plane blocks (e.g., transversus abdominis plane, erector spinae plane)**	Agree	NA	NA	NA	NA	NA	NA	+	+	NA	NA	NG	NA	+	+	NA	NA	+	NG	NG	NG	NA	NA
**7. Patient-controlled intravenous analgesia**	Agree	NA	NA	NA	NA	NA	NA	NG	+	NA	NA	NG	NA	+	+	NA	NA	NG	+	NG	NG	NA	NA

+: consistent the reference with content;

+/-: partial1y consistent the reference with content;

For peer review only

For peer review only - http://bmjopen.bmj.com/site/about/guidelines.xhtml

-: inconsistent the reference with content;
NA: not applicable;
NG: not given.

For peer review only

For peer review only - http://bmjopen.bmj.com/site/about/guidelines.xhtml

Supplementary Table 14. Summary of recommendations for the adverse events by included guidance document.

Recommendations	Reference contents	OG-OG-CMA_2014	OG-A-CMA_2016	ASA_2016	AAGBI_2017	ERAS_2018 (Part 1)	ERAS_2018 (Part 2)	ERAS_2018 (Part 3)	ACOG_2019	SOGC_2019	SOGC_2022	SOAP_2019	AA and OAA_2020	PROSPECT_2020	CHBSA_2020	CAA_2020	CAA_2020	SOAP_2020	NICE_2021	OAA_2022	ANNA_2023	RCoA_2023	French_2021
**1. Hypotension prevention strategies**	Provided	NA	NA	+	+	NA	+/-	+	+	+	NA	NA	NA	NA	+	+	+	+	+	NA	+/-	NA	NA
1.1 Blood pressure target of systolic arterial pressure	≥90% of baseline and avoid decrease to < 80% baseline	NA	NA	NG	+	NA	NG	NG	NG	NG	NA	NA	NA	NA	NG	NG	NG	NG	+	NA	NG	NA	NA
1.2 Position (uterine left-leaning)	Yes	NA	NA	+	+	NA	NG	NG	NG	NG	NA	NA	NA	NA	+	+	+	NG	+	NA	+	NA	NA
1.3 Volume expansion (crystals or colloids)	500-1000 ml	NA	NA	+	+	NA	+	+	+	+	NA	NA	NA	NA	+	+	+	NG	+	NA	+	NA	NA
1.4 Vasoactive drugs	Yes	NA	NA	+	+	NA	NG	+	+	NG	NA	NA	NA	NA	+	+	+	+	+	NA	+	NA	NA
-Pump infusion is recommended	Provided	NA	NA	+	+	NA	NG	NG	NG	NG	NA	NA	NA	NA	NG	NG	NG	NG	NG	NA	NG	NA	NA
-Type of drugs	Provided	NA	NA	+	+	NA	NG	+	+	NG	NA	NA	NA	NA	+	+	+	+	NG	NA	+/-	NA	NA
--Phenylephrine	20-50 μg Or	NA	NA	+	20~50 μg or	NA	NG	+	+	NG	NA	NA	NA	NA	+	+	+	+	NG	NA	+	NA	NA

For peer review only - http://bmjopen.bmj.com/site/about/guidelines.xhtml

	0.5 µg . kg^{-1}. min^{-1}				0.25. kg^{-1}. min^{-1}																		
--Metaraminol	1-2 mg or 4 µg.kg^{-1}. min^{-1}	NA	NA	NG	0.5mg.ml^{-1}	NA	NG	NG	NG	NG	NA	NA	NA	NA	NG	NG	NG	NG	NG	NA	NG	NA	NA
--Noradrenalin	4-6 µg or 0.08 µg kg^{-1}. min^{-1}	NA	NA	NG	NG	NA	NG	NG	NG	NG	NA	NA	NA	NA	NG	+	NG	NG	NG	NA	NG	NA	NA
--Ephedrine	2-15 mg for IV	NA	NA	NG	2-4mg	NA	NG	+	+	NG	NA	NA	NA	NA	+	+	+	NG	NG	NA	NG	NA	NA
1.5 Prophylactic vasopressor infusion should not be recommended in pre-eclampsia	Agree	NA	NA	NG	+	NA	NG	NG	NG	NG	NA	NA	NA	NA	NG	+	NG	NG	NG	NA	NG	NA	NA
**2 Inadequate neuraxial anesthesia**	Provided	NA	NA	NA	NA	NA	NA	NA	+	NA	NA	NA	NA	NA	NA	NA	+	NA	NA	+	+	+	+
2.1 Block assessment	Provided	NA	NA	NA	NA	NA	NA	NA	NG	NA	NA	NA	NA	NA	NA	NA	NG	NA	NA	+	+	NG	+
-Testing sensory block after the evidence of motor block	Agree	NA	NA	NA	NA	NA	NA	NA	NG	NA	NA	NA	NA	NA	NA	NA	NG	NA	NA	+	+	NG	NG
-Check the block at relatively fixed time intervals	Agree	NA	NA	NA	NA	NA	NA	NA	NG	NA	NA	NA	NA	NA	NA	NA	NG	NA	NA	+	NG	NG	NG
-Perform the first check early enough to allow positional changes	Agree	NA	NA	NA	NA	NA	NA	NA	NG	NA	NA	NA	NA	NA	NA	NA	NG	NA	NA	+	NG	NG	NG

For peer review only

2.2 Rescue for inadequate neuraxial block (change anesthesia method)	Provided	NA	NA	NA	NA	NA	NA	NA	+	NA	NA	NA	NA	NA	NA	NA	NG	NA	NA	+	+	NG	+
-A second neuraxial technique (in the case of spinal anesthesia)	Agree	NA	NA	NA	NA	NA	NA	NA	+	NA	NA	NA	NA	NA	NA	NA	+	NA	NA	+	+	NG	+
-Extending the neuraxial technique (in the case of combined spinal–epidural)	Agree	NA	NA	NA	NA	NA	NA	NA	+	NA	NA	NA	NA	NA	NA	NA	NG	NA	NA	+	+	NG	NG
-Above options are not possible, general anesthesia should be recommended	Agree	NA	NA	NA	NA	NA	NA	NA	+	NA	NA	NA	NA	NA	NA	NA	NG	NA	NA	+	+	NG	+
2.3 Rescue for inadequate neuraxial block (change anesthetic)	Provided	NA	NA	NA	NA	NA	NA	NA	+	NA	NA	NA	NA	NA	NA	NA	NG	NA	NA	+	+	NG	+
-Nitrous oxide and oxygen alone	Disagree	NA	NA	NA	NA	NA	NA	NA	NG	NA	NA	NA	NA	NA	NA	NA	NG	NA	NA	+	NG	NG	NG
-Continue with neuraxial block, consider repeated boluses of fast acting opioids	Agree	NA	NA	NA	NA	NA	NA	NA	NG	NA	NA	NA	NA	NA	NA	NA	NG	NA	NA	+	NG	NG	NG
--Fentanyl	25-50 µg	NA	NA	NA	NA	NA	NA	NA	NG	NA	NA	NA	NA	NA	NA	NA	NG	NA	NA	+	NG	NG	NG
--Alfentanil	250-500 µg	NA	NA	NA	NA	NA	NA	NA	NG	NA	NA	NA	NA	NA	NA	NA	NG	NA	NA	+	NG	NG	NG
--Ketamine	10 mg boluses	NA	NA	NA	NA	NA	NA	NA	NG	NA	NA	NA	NA	NA	NA	NA	NG	NA	NA	+	10 mg or	NG	

For peer review only

																					0.15 mg .kg^{-1}		+/-
-Do not treat pain with anxiolytics	Agree	NA	NA	NA	NA	NA	NA	NA	NG	NA	NA	NA	NA	NA	NA	NA	NG	NA	NA	+	NG	NG	NG
-Above options are not possible, general anesthesia should be recommended	Agree	NA	NA	NA	NA	NA	NA	NA	+	NA	NA	NA	NA	NA	NA	NA	NG	NA	NA	+	+	NG	+
**3. Post dural puncture headache (PDPH)**	Provided	NA	NA	NA	NA	NA	NA	NA	+	NA	NA	NA	NA	NA	NA	NA	NA	NA	NA	NA	+	+/-	NA
3.1 Measures be used to prevent PDPH	Provided	NA	NA	NA	NA	NA	NA	NA	NG	NA	NA	NA	NA	NA	NA	NA	NA	NA	NA	NA	+/-	NG	NA
-Noncutting spinal needles are associated with decreased PDPH risk	Agree	NA	NA	NA	NA	NA	NA	NA	NG	NA	NA	NA	NA	NA	NA	NA	NA	NA	NA	NA	+	NG	NA
-An intrathecal catheter may be considered to provide anesthesia or analgesia	Agree	NA	NA	NA	NA	NA	NA	NA	NG	NA	NA	NA	NA	NA	NA	NA	NA	NA	NA	NA	+	NG	NA
-A prophylactic epidural blood patch is not recommended as routine	Agree	NA	NA	NA	NA	NA	NA	NA	NG	NA	NA	NA	NA	NA	NA	NA	NA	NA	NA	NA	NG	NG	NA
-Bed rest is not routinely recommended	Agree	NA	NA	NA	NA	NA	NA	NA	NG	NA	NA	NA	NA	NA	NA	NA	NA	NA	NA	NA	NG	NG	NA

For peer review only - http://bmjopen.bmj.com/site/about/guidelines.xhtml

as prophylaxis against PDPH																							
-Routine injection of substance to prevent PDPH is not recommended	Agree	NA	NA	NA	NA	NA	NA	NA	NG	NA	NA	NA	NA	NA	NA	NA	NA	NA	NA	NA	NG	NG	NA
-Routine systemic drug for PDPH prophylaxis is not recommended	Agree	NA	NA	NA	NA	NA	NA	NA	NG	NA	NA	NA	NA	NA	NA	NA	NA	NA	NA	NA	NG	NG	NA
3.2 Measures be used to treat PDPH	Provided	NA	NA	NA	NA	NA	NA	NA	NG	NA	NA	NA	NA	NA	NA	NA	NA	NA	NA	NA	+/-	NG	NA
-Epidural blood patch	Agree	NA	NA	NA	NA	NA	NA	NA	+	NA	NA	NA	NA	NA	NA	NA	NA	NA	NA	NA	+	NG	NA
-The routine use of bed rest to treat PDPH	Disagree	NA	NA	NA	NA	NA	NA	NA	NG	NA	NA	NA	NA	NA	NA	NA	NA	NA	NA	NA	NG	NG	NA
-Adequate hydration should be maintained with oral fluids or intravenous	Agree	NA	NA	NA	NA	NA	NA	NA	NG	NA	NA	NA	NA	NA	NA	NA	NA	NA	NA	NA	+	NG	NA
-Routine use of abdominal binders or aromatherapy to treat PDPH	Disagree	NA	NA	NA	NA	NA	NA	NA	NG	NA	NA	NA	NA	NA	NA	NA	NA	NA	NA	NA	NG	NG	NA
-Regular multimodal analgesia	Agree	NA	NA	NA	NA	NA	NA	NA	NG	NA	NA	NA	NA	NA	NA	NA	NA	NA	NA	NA	+/-	NG	NA
-Short-term use of opioids if regular	Agree	NA	NA	NA	NA	NA	NA	NA	NG	NA	NA	NA	NA	NA	NA	NA	NA	NA	NA	NA	NG	NG	NA

For peer review only

multimodal analgesia is ineffective																							
-Long-term opioid use is not recommended in the treatment of PDPH	Agree	NA	NA	NA	NA	NA	NA	NA	NG	NA	NA	NA	NA	NA	NA	NA	NA	NA	NA	NA	NG	NG	NA
-Caffeine offered in the first 24h with a maximum dose of 900 mg per day	Agree	NA	NA	NA	NA	NA	NA	NA	NG	NA	NA	NA	NA	NA	NA	NA	NA	NA	NA	NA	+/-	NG	NA
-Caffeine offered 200-300 mg if breastfeeding	Agree	NA	NA	NA	NA	NA	NA	NA	NG	NA	NA	NA	NA	NA	NA	NA	NA	NA	NA	NA	NG	NG	NA
-Routine use of hydrocortisone, adrenocorticotropic hormone or cosyntropin	Disagree	NA	NA	NA	NA	NA	NA	NA	NG	NA	NA	NA	NA	NA	NA	NA	NA	NA	NA	NA	+/-	NG	NA
-Routine use of theophylline, neostigmine or atropine, piritramide, and gabapentin	Disagree	NA	NA	NA	NA	NA	NA	NA	NG	NA	NA	NA	NA	NA	NA	NA	NA	NA	NA	NA	NG	NG	NA
-Routine use of acupuncture to treat PDPH	Disagree	NA	NA	NA	NA	NA	NA	NA	NG	NA	NA	NA	NA	NA	NA	NA	NA	NA	NA	NA	NG	NG	NA
-Routine use of sphenopalatine ganglion blocks to treat PDPH	Disagree	NA	NA	NA	NA	NA	NA	NA	NG	NA	NA	NA	NA	NA	NA	NA	NA	NA	NA	NA	+	NG	NA

For peer review only - http://bmjopen.bmj.com/site/about/guidelines.xhtml

-Greater occipital nerve blocks be offered to patients with PDPH	Agree	NA	NA	NA	NA	NA	NA	NA	NG	NA	NA	NA	NA	NA	NA	NA	NA	NA	NA	NA	NG	NG	NA
-The use of spinal and epidural morphine to treat PDPH	Disagree	NA	NA	NA	NA	NA	NA	NA	NG	NA	NA	NA	NA	NA	NA	NA	NA	NA	NA	NA	NG	NG	NA
-Routine use of epidural dextran, gelatine, or hydroxyethyl starch to treat PDPH	Disagree	NA	NA	NA	NA	NA	NA	NA	NG	NA	NA	NA	NA	NA	NA	NA	NA	NA	NA	NA	NG	NG	NA
-Routine use of fibrin glue to treat PDPH	Disagree	NA	NA	NA	NA	NA	NA	NA	NG	NA	NA	NA	NA	NA	NA	NA	NA	NA	NA	NA	NG	NG	NA
-Fibrin glue should be reserved for management of PDPH refractory to EBP	Agree	NA	NA	NA	NA	NA	NA	NA	NG	NA	NA	NA	NA	NA	NA	NA	NA	NA	NA	NA	NG	NG	NA
-Fibrin glue be used when autologous blood injection is contraindicated	Agree	NA	NA	NA	NA	NA	NA	NA	NG	NA	NA	NA	NA	NA	NA	NA	NA	NA	NA	NA	NG	NG	NA

+: consistent the reference with content;

+/-: partial1y consistent the reference with content;

-: inconsistent the reference with content;

NA: not applicable;

NG: not given.

For peer review only

Supplementary Table 15. Summary of recommendations for the monitoring and management of obstetric and anesthetic emergencies by included guidance document.

Recommendations	Reference contents	OG-OG-CMA_2014	OG-A-CMA_2016	ASA_2016	AAGBI_2017	ERAS_2018 (Part 1)	ERAS_2018 (Part 2)	ERAS_2018 (Part 3)	ACOG_2019	SOGC_2019	SOGC_2022	SOAP_2019	AA and OAA_2020	PROSPECT_2020	CHBSA_2020	CAA_2020	CAA_2020	SOAP_2020	NICE_2021	OAA_2022	ANNA_2023	RCoA_2023	French_2021
**1. Resources for management of Hemorrhagic emergencies**	Provided	NA	NA	+	NA	NA	NA	NA	NA	NA	NA	NA	NA	NA	NA	+/-	NA	NA	NA	NA	+	+/-	NA
-Large-bore IV catheters	Agree	NA	NA	+	NA	NA	NA	NA	NA	NA	NA	NA	NA	NA	NA	+	NA	NA	NA	NA	+	NG	NA
-Fluid warmer	Agree	NA	NA	+	NA	NA	NA	NA	NA	NA	NA	NA	NA	NA	NA	NG	NA	NA	NA	NA	NG	NG	NA
-Forced-air body warmer	Agree	NA	NA	+	NA	NA	NA	NA	NA	NA	NA	NA	NA	NA	NA	NG	NA	NA	NA	NA	NG	NG	NA
-Availability of blood bank resources	Agree	NA	NA	+	NA	NA	NA	NA	NA	NA	NA	NA	NA	NA	NA	NG	NA	NA	NA	NA	+	NG	NA
-Massive transfusion protocol (type-specific, or O-negative blood, or salvage)	Agree	NA	NA	+	NA	NA	NA	NA	NA	NA	NA	NA	NA	NA	NA	NG	NA	NA	NA	NA	+	NG	NA
-Equipment for infusing IV fluids and blood products rapidly	Agree	NA	NA	+	NA	NA	NA	NA	NA	NA	NA	NA	NA	NA	NA	NG	NA	NA	NA	NA	NG	NG	NA

**2. Equipment for management of airway emergencies**	Provided	NA	NA	+	NA	NA	NA	NA	+	+/-	NA	NA	NA	NA	NA	+	NA	NA	+	NA	+/-	+/-	NA
2.1 Laryngoscope and assorted blades	Agree	NA	NA	+	NA	NA	NA	NA	+	NG	NA	NA	NA	NA	NA	NG	NA	NA	NG	NA	+	NG	NA
2.2 Endotracheal tubes, with stylets	Agree	NA	NA	+	NA	NA	NA	NA	+	NG	NA	NA	NA	NA	NA	NG	NA	NA	NG	NA	+	NG	NA
2.3 Oxygen source	Agree	NA	NA	+	NA	NA	NA	NA	NG	NG	NA	NA	NA	NA	NA	NG	NA	NA	+	NA	+	NG	NA
2.4 Suction source with tubing and tonsil suction tip	Agree	NA	NA	+	NA	NA	NA	NA	NG	NG	NA	NA	NA	NA	NA	NG	NA	NA	+	NA	+	NG	NA
2.5 Self-inflating bag and mask for positive-pressure ventilation	Agree	NA	NA	+	NA	NA	NA	NA	NG	NG	NA	NA	NA	NA	NA	NG	NA	NA	NG	NA	+	NG	NA
2.6 Medications for blood pressure support, muscle relaxation, and hypnosis	Agree	NA	NA	+	NA	NA	NA	NA	NG	NG	NA	NA	NA	NA	NA	NG	NA	NA	NA	NA	NG	NG	NA
2.7 Tracheal intubation has failed protocol	Agree	NA	NA	+	NA	NA	NA	NA	NG	NG	NA	NA	NA	NA	NA	+	NA	NA	+	NA	+	NG	NA
**3. Cardiopulmonary Resuscitation**	Provided	NA	NA	+	NA	NA	NA	NA	NA	NA	NA	NA	NA	NA	NA	NA	NA	NA	NA	NA	+/-	+/-	NA
3.1 Basic and advanced life-support equipment	Agree	NA	NA	+	NA	NA	NA	NA	NA	NA	NA	NA	NA	NA	NA	NA	NA	NA	NA	NA	+	NG	NA

For peer review only

3.2 Cardiac arrest occurs	Provided	NA	NA	+	NA	NA	NA	NA	NA	NA	NA	NA	NA	NA	NA	NA	NA	NA	NA	NA	+	NG	NA
-Uterine displacement (usually left displacement) should be maintained	Agree	NA	NA	+	NA	NA	NA	NA	NA	NA	NA	NA	NA	NA	NA	NA	NA	NA	NA	NA	+	NG	NA
-If circulation is not restored within 4 min, cesarean should be performed	Agree	NA	NA	+	NA	NA	NA	NA	NA	NA	NA	NA	NA	NA	NA	NA	NA	NA	NA	NA	4-5 min	NG	NA
**4. Emergency cesarean delivery**	Provided	NA	NA	+	NA	NA	NA	NA	+	NA	NA	NA	NA	NA	NA	NA	NA	NA	+	NA	+/-	+/-	+
4.1 Response times limited	30 or 75 minutes	NA	NA	NG	NA	NA	NA	NA	NG	NA	NA	NA	NA	NA	NA	NA	NA	NA	+	NA	NG	NG	15-30 minutes
4.2 Anesthesia methods recommendation	Provided	NA	NA	+/-	NA	NA	NA	NA	+	NA	NA	NA	NA	NA	NA	NA	NA	NA	+/-	NA	NG	NG	+
-Spinal	Agree	NA	NA	+/-	NA	NA	NA	NA	+	NA	NA	NA	NA	NA	NA	NA	NA	NA	NG	NA	NG	NG	+
-Epidural	Agree	NA	NA	+/-	NA	NA	NA	NA	+	NA	NA	NA	NA	NA	NA	NA	NA	NA	NG	NA	NG	NG	+
-Combined spinal–epidural analgesia	Agree	NA	NA	+/-	NA	NA	NA	NA	+	NA	NA	NA	NA	NA	NA	NA	NA	NA	NG	NA	NG	NG	+
-General anesthesia	Agree	NA	NA	+/-	NA	NA	NA	NA	+	NA	NA	NA	NA	NA	NA	NA	NA	NA	NG	NA	NG	NG	+

+/-: partial1y consistent the reference with content;

-: inconsistent the reference with content;

NA: not applicable;

NG: not given.

For peer review only - http://bmjopen.bmj.com/site/about/guidelines.xhtml

For peer review only

- Consistent with the reference content
- Inconsistent with the reference content
- Partially consistent with the reference content
- Not given
- Not applicable

Recommendations	Reference contents	OG-OG-CMA_2014	OG-A-CMA_2016	ASA_2016	AAGBI_2017	ERAS_2018 (Part 1)	ERAS_2018 (Part 2)	ERAS_2018 (Part 3)	ACOG_2019	SOGC_2019	SOGC_2022	SOAP_2019	AA and OAA_2020	PROSPECT_2020	CHBSA_2020	CAA_2020	CAA_2020	SOAP_2020	NICE_2021	OAA_2022	ANNA_2023	RCoA_2023	French_2021
**Peri-anesthetic Evaluation and Preparation**																							
**1. History and physical examination before providing anesthetic care**	Yes	C	C	C	NA	NG	NA	NA	C	NA	NA	NA	NA	NA	C	C	C	NG	P	NA	C	NG	C
1.1 Maternal health and anesthetic history	Agree	C	C	C	NA	NG	NA	NA	C	NA	NA	NA	NA	NA	C	C	C	NG	NG	NA	P	NG	C
1.2 Relevant obstetric history	Agree	C	C	C	NA	NG	NA	NA	NG	NA	NA	NA	NA	NA	NG	C	C	NG	NG	NA	NG	NG	NG
1.3 Baseline blood pressure measurement	Agree	C	C	C	NA	NG	NA	NA	NG	NA	NA	NA	NA	NA	C	I	C	NG	NG	NA	P	NG	NG
1.4 Airway examination	Agree	NG	C	C	NA	NG	NA	NA	C	NA	NA	NA	NA	NA	NG	C	C	NG	NG	NA	C	NG	NG
1.5 Heart and lung examination	Agree	NG	NG	C	NA	NG	NA	NA	C	NA	NA	NA	NA	NA	C	C	C	NG	NG	NA	C	NG	NG
1.6 When a neuraxial anesthetic is planned, examine the patient’s back	Agree	NG	C	C	NA	NG	NA	NA	C	NA	NA	NA	NA	NA	NG	C	C	NG	NG	NA	C	NG	NG
1.7 Consultation between the obstetrician and the anesthesiologist	Agree	C	NG	C	NA	C	NA	NA	C	NA	NA	NA	NA	NA	NG	C	C	NG	NG	NA	C	NG	C
**2. Preoperative communication**	Encouraged	C	NG	C	NA	C	NA	NA	NG	NA	NA	NA	NA	NA	C	C	I	P	C	NA	C	P	C
**3. Intrapartum platelet count**	Yes	C	C	C	NA	NA	NA	NA	C	NA	NA	NA	NA	NA	NG	C	NG	NG	C	NA	NG	NG	NA
-Routine	Agree	C	C	C	NA	NA	NA	NA	C	NA	NA	NA	NA	NA	NG	NG	NG	NG	I	NA	NG	NG	NA
-Individualized	Agree	I	I	C	NA	NA	NA	NA	C	NA	NA	NA	NA	NA	NG	NG	NG	NG	C	NA	NG	NG	NA
**4. Blood type and screen**	Yes	C	C	C	NA	NA	NA	NA	NG	NA	NA	NA	NA	NA	NG	C	NG	NG	C	NA	NG	NG	NA
-Routine	Agree	C	C	I	NA	NA	NA	NA	NG	NA	NA	NA	NA	NA	NG	C	NG	NG	I	NA	NG	NG	NA
-Individualized	Agree	I	I	C	NA	NA	NA	NA	NG	NA	NA	NA	NA	NA	NG	NG	NG	NG	C	NA	NG	NG	NA
**5. Blood cross-match**	Yes	C	NG	C	NA	NA	NA	NA	NG	NA	NA	NA	NA	NA	NG	NG	NG	NG	C	NA	NG	NG	NA
-Routine	Agree	C	NG	I	NA	NA	NA	NA	NG	NA	NA	NA	NA	NA	NG	NG	NG	NG	I	NA	NG	NG	NA
-Individualized	Agree	NG	NG	C	NA	NA	NA	NA	NG	NA	NA	NA	NA	NA	NG	NG	NG	NG	C	NA	NG	NG	NA
6. Peri-anesthetic recording of fetal heart rate	Yes	C	C	C	NA	NA	NA	NA	C	C	C	NA	NA	NA	NG	C	NG	NG	NG	NA	C	NG	NA
**7. Aspiration prevention**	Provided	C	NG	C	NA	C	NA	NA	NA	C	NA	NA	NA	NA	C	P	NG	P	P	NA	P	P	NA
7.1 Clear liquids	Up to 2 h	NG	NG	C	NA	C	NA	NA	NA	NG	NA	NA	NA	NA	C	C	NG	C	NG	NA	NG	P	NA
7.2 Solids	6-8 h	C	NG	C	NA	C	NA	NA	NA	NG	NA	NA	NA	NA	C	C	NG	C	NG	NA	NG	NG	NA
7.3 Antacids (surgical procedures-caesarean delivery or postpartum tubal ligation)	Agree	NG	NG	C	NA	C	NA	NA	NA	NG	NA	NA	NA	NA	C	C	NG	NG	C	NA	NG	C	NA
7.4 H_2-receptor antagonists (surgical procedures)	Agree	NG	NG	C	NA	C	NA	C	NA	NG	NA	NA	NA	NA	C	C	NG	NG	C	NA	NG	NG	NA
7.5 Metoclopramide (surgical procedures)	Agree	NG	NG	C	NA	NG	NA	C	NA	NG	NA	NA	NA	NA	NG	C	NG	NG	NG	NA	NG	NG	NA
**8. Preoperative heparin or low-molecular-weight heparin**	Yes	NG	C	NG	NA	NA	NA	NA	C	NA	NA	NA	NA	NA	NA	NG	P	P	NG	NA	P	P	NA
8.1 Prophylactic subcutaneous unfractionated heparin (<5,000 units twice daily)	Provided	NG	NG	NG	NA	NA	NA	NA	C	NA	NA	NA	NA	NA	NA	NG	NG	NG	NG	NA	NG	NG	NA
-Contraindication to neuraxial techniques	No	NG	NG	NG	NA	NA	NA	NA	C	NA	NA	NA	NA	NA	NA	NG	NG	NG	NG	NA	NG	NG	NA
-Catheter placement or removal since last dose	4-6 hours	NG	NG	NG	NA	NA	NA	NA	C	NA	NA	NA	NA	NA	NA	NG	NG	NG	NG	NA	NG	NG	NA
8.2 Intermediate unfractionated heparin (75,000-100,000 units)	Provided	NG	NG	NG	NA	NA	NA	NA	C	NA	NA	NA	NA	NA	NA	NG	NG	NG	NG	NA	NG	NG	NA
-Catheter placement or removal since last dose	> 12 hours	NG	NG	NG	NA	NA	NA	NA	C	NA	NA	NA	NA	NA	NA	NG	NG	NG	NG	NA	NG	NG	NA
8.3 High-dose unfractionated heparin (total daily dose greater than 20,000 units)	Provided	NG	NG	NG	NA	NA	NA	NA	C	NA	NA	NA	NA	NA	NA	NG	NG	NG	NG	NA	NG	NG	NA
-Catheter placement or removal since last dose	> 24 hours	NG	NG	NG	NA	NA	NA	NA	C	NA	NA	NA	NA	NA	NA	NG	NG	NG	NG	NA	NG	NG	NA
-With the activated partial thromboplastin time being within normal range	Yes	NG	NG	NG	NA	NA	NA	NA	C	NA	NA	NA	NA	NA	NA	NG	NG	NG	NG	NA	NG	NG	NA
**Anesthetic Care for labor and Vaginal Delivery**																							
**1. Equipment, facilities, and support personnel**	Agree	NA	C	C	NA	NA	NA	NA	C	NA	NA	NA	NA	NA	C	NA	C	NA	NA	NA	C	C	NA
**2. Timing of neuraxial analgesia and outcome of labor**	Provided	NA	C	C	NA	NA	NA	NA	NG	NA	NA	NA	NA	NA	NG	NA	C	NA	NA	NA	NG	NG	NA

2.1 Less 5 cm dilation	Agree
2.2 Offer neuraxial analgesia on an individualized basis	Agree
**3. Neuraxial analgesia and trial of labor after prior caesarean delivery**	Agree
**4. Continuous infusion epidural analgesia**	Provided
4.1 Using local anesthetic	Agree
4.2 Add opioid	Agree
4.3 Platelet concentration corresponding to contraindications to puncture	Less than $70*10^9/L$
4.4 Puncture location	L_{2-3} or L_{3-4}
4.5 Analgesic concentrations	Provided
-Bupivacaine	Provided
--Loading dose	0.04-0.125%
--Maintenance dose	0.05-0.125%
-Ropivacaine	Provided
--Loading dose	0.0625-0.15%
--Maintenance dose	0.0625-0.125%
-Levobupivacaine	Provided
--Loading dose	0.04-0.125%
--Maintenance dose	0.05-0.125%
4.6 Opioids concentrations	Provided
-Fentanyl	Provided
--Loading dose	0.5-2 μg/ml
--Maintenance dose	1-2 μg/ml
-Sufentanyl	Provided
--Loading dose	0.2~0.6 μg/ml
--Maintenance dose	0.3~0.6 μg/ml
**5. Combined spinal–epidural analgesia**	Provided
5.1 Using local anesthetic	Agree
5.2 Add opioid	Agree
5.3 Platelet concentration corresponding to contraindications to puncture	Less than $70*10^9/L$
5.4 Puncture location	L_{2-3} or L_{3-4}
5.5 Epidural anesthetics (consistent with 4.5 and 4.6)	Provided
5.6 Spinal anesthetics	Provided
-Protocol 1	Provided
--Single sufentanyl	2.5-7 μg
--Single ropivacaine	2.3-3 mg
--Combined sufentanyl and ropivacaine	2.5 μg / 2.5 mg
-Protocol 2	Provided
--Single fentanyl	15-25 μg
--Single bupivacaine	2.0-2.5 mg
--Combined fentanyl and bupivacaine	2.5 μg / 2.0 mg
**6. Single-injection spinal**	Provided
6.1 Using local anesthetic	Agree
6.2 Add opioid	Agree
6.3 Platelet concentration corresponding to contraindications to puncture	Less than $50*10^9/L$
6.4 Puncture location	L_{3-4}

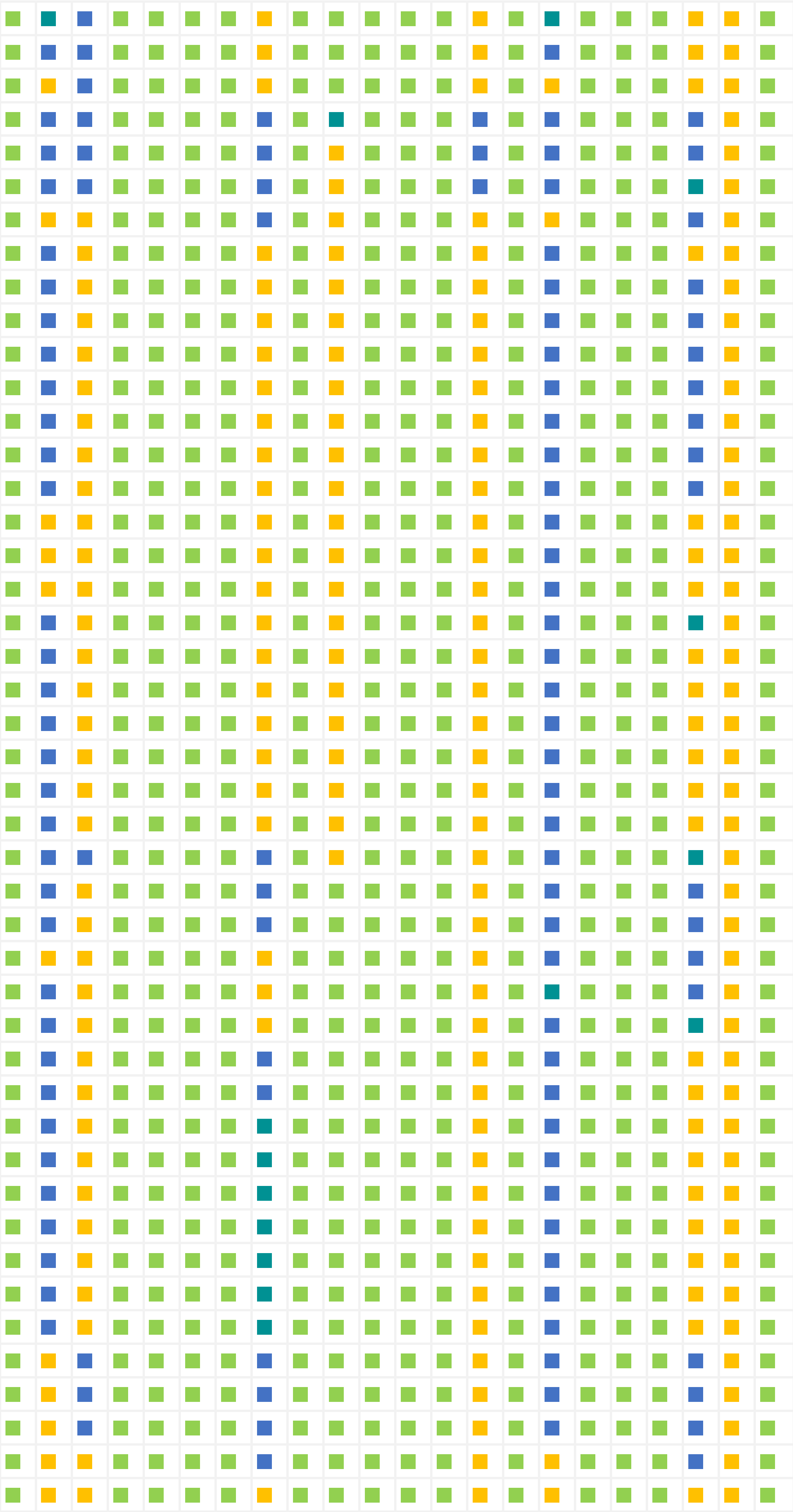

6.6 Analgesic concentrations (consistent with 5.6)	Agree
6.7 Spinal needles	Provided
-Cutting-bevel	Disagree
-Pencil-point	Agree
**7. Patient-controlled epidural analgesia (for labor analgesia)**	Agree
**Anesthetic Care for caesarean Delivery**	
**1. Equipment, facilities, and support personnel**	Agree
**2. Continuous infusion epidural analgesia**	Provided
2.1 Using local anesthetic	Agree
2.2 Add opioid	Agree
2.3 Platelet concentration corresponding to contraindications to puncture	Less than $70*10^9/L$
2.4 Puncture location	L_{1-2} or L_{2-3}
2.5 Recommend level of block	T_{6-4}
2.6 Analgesic concentrations	Provided
-Lidocaine	1.5%-2%
-Bupivacaine	0.50%
-Ropivacaine	0.5%-0.75%
-Levobupivacaine	0.5%-0.75%
-Cloprocaine	3%
2.7 Prevention of local anesthetic poisoning	Provided
-Withdrawal before injection	Agree
-Administration of test dose	Agree
-Addition of epinephrine	Agree
**3. Single-injection spinal**	Provided
3.1 Using local anesthetic	Agree
3.2 Add opioid	Agree
3.3 Platelet concentration corresponding to contraindications to puncture	Less than $70*10^9/L$
3.4 Puncture location	L_{3-4}
3.5 Recommend level of block	T_{6-4}
3.6 Analgesic concentrations	Provided
-Bupivacaine	5~15 mg
-Ropivacaine	10~20 mg
3.7 Spinal needles	Provided
-Cutting-bevel	Disagree
-Pencil-point	Agree
**4. Combined spinal–epidural analgesia**	Provided
4.1 Puncture location	Provided
-Single space technique	L_{3-4}
-Double-space technique	$T_{12}-L_2/L_{3-5}$
4.2 Anesthetics (consistent with spinal or epidural)	Agree
**5. General anesthesia**	Provided
5.1 General anesthesia selected condition	Provided
-Profound fetal bradycardia	Agree
-Ruptured uterus	Agree
-Severe hemorrhage	Agree

-Severe placental abruption	Agree
-Umbilical cord prolapse	Agree
-Preterm footling breech	Agree
-Inability to place neuraxial anesthesia	
5.2 Intravenous anesthetics recommendation	Provided
-Thiopental sodium	4-5 mg/kg
-Propofol	1.5-2.5 mg/kg
-Etomidate	0.2-0.3 mg/kg
-Ketamine	0.5-1 mg/kg
5.3 Opioids analgesic recommendation	Provided
-Fentanyl	2-5 μg/kg
-Sufentanyl	0.2-0.5 μg/kg
-Remifentanyl	0.5-1 μg/kg for IV
-Remifentanyl	4 ng/kg for target
5.4 Muscle relaxants recommendation	Provided
-Succinylcholine	1-1.5 mg/kg
-Rocuronium	0.6-1.2 mg/kg
5.5 Induction-delivery interval	within 10 min
**Neurological Monitoring Associated With Obstetric Neuraxial Block**	
**1. Screening method (During the recovery phase) -Straight-leg raising**	Agree
**2. Alerts incidences-unable to straight-leg raise at 4 h from the last dose**	Agree
**Respiratory Depression Monitoring With Neuraxial Block With Opioids**	
**1. Frequency and modality of respiratory monitoring should be based on:**	Provided
1.1 Low risk	Provided
-Neuraxial morphine dose (intrathecal ≤ 0.15 mg or epidural ≤ 3 mg)	Agree
1.2 High risk	Provided
-Cardiopulmonary/neurological co-morbidity	Agree
-Obesity (body mass index ≥ 40 kg/m^2)	Agree
-Known or suspected obstructive sleep apnea	Agree
-Chronic opioid use or abuse	Agree
-Hypertension	Agree
-Magnesium administration	Agree
-Neuraxial morphine dose (intrathecal > 0.15 mg or epidural > 3 mg)	Agree
1.3 Respiratory monitoring frequency and duration	Provided
-Low risk	Provided
--Routine postoperative vital sign monitoring	Agree
-- q2h clinical assessment for 12 h	Agree
-High risk	Provided
-- q1h clinical assessment for first 12 h	Agree
-- q2h clinical assessment for first 12-24 h	Agree
--Consider additional monitoring modalities (e.g., pulse oximetry, capnography)	Agree
**Management of Obstetric and anesthetic Emergencies or Adverse Events**	
**1. Resources for management of hemorrhagic emergencies**	Provided
-Large-bore IV catheters	Agree
-Fluid warmer	Agree
-Forced-air body warmer	Agree

-Availability of blood bank resources	Agree
-Massive transfusion protocol (type-specific, or O-negative blood, or salvage)	Agree
-Equipment for infusing IV fluids and blood products rapidly	Agree
**2. Hypotension prevention strategies**	Provided
2.1 Blood pressure target of systolic arterial pressure	⩾ 90% of baseline obtained
2.2 Position (uterine left-leaning)	Yes
2.3 Volume expansion (crystals or colloids)	500-1000 ml
2.4 Vasoactive drugs	Yes
-Pump infusion is recommended	Provided
-Type of drugs	Provided
--Phenylephrine	20-50 μg or 0.5 μg. kg^{-1}. min^{-1}
--Methoxymine	1-2 mg or 4 μg. kg-1. min-1
--Noradrenalin	4-6 μg or 0.08 μg. kg-1. min-1
--Efedrina	5-15 mg for IV
2.5 Prophylactic vasopressor infusion should not be recommended in pre-eclampsia	Agree
**3. Equipment for management of airway emergencies**	Provided
3.1 Laryngoscope and assorted blades	Agree
3.2 Endotracheal tubes, with stylets	Agree
3.3 Oxygen source	Agree
3.4 Suction source with tubing and tonsil suction tip	Agree
3.5 Self-inflating bag and mask for positive-pressure ventilation	Agree
3.6 Medications for blood pressure support, muscle relaxation, and hypnosis	Agree
3.7 Tracheal intubation has failed protocol	Agree
**4. Cardiopulmonary Resuscitation**	Provided
4.1 Basic and advanced life-support equipment	Agree
4.2 Cardiac arrest occurs	Provided
-Uterine displacement (usually left displacement) should be maintained	Agree
-If circulation is not restored within 4 min, caesarean should be performed	Agree
**5. Emergency caesarean delivery**	Provided
5.1 Response times limited	30 minutes
5.2 Anesthesia methods recommendation	Provided
-Spinal	Agree
-Epidural	Agree
-Combined spinal–epidural analgesia	Agree
-General anesthesia	Agree
**6. Inadequate neuraxial anesthesia**	Provided
6.1 Block assessment	Provided
-Testing sensory block after the evidence of motor block	Agree
-Check the block at relatively fixed time intervals	Agree
-Perform the first check early enough to allow positional changes	Agree
6.2 Rescue for inadequate neuraxial block (change anesthesia method)	Provided
-A second neuraxial technique (in the case of spinal anesthesia)	Agree
-Extending the neuraxial technique (in the case of combined spinal–epidural)	Agree
-Above options are not possible, general anesthesia should be recommended	Agree

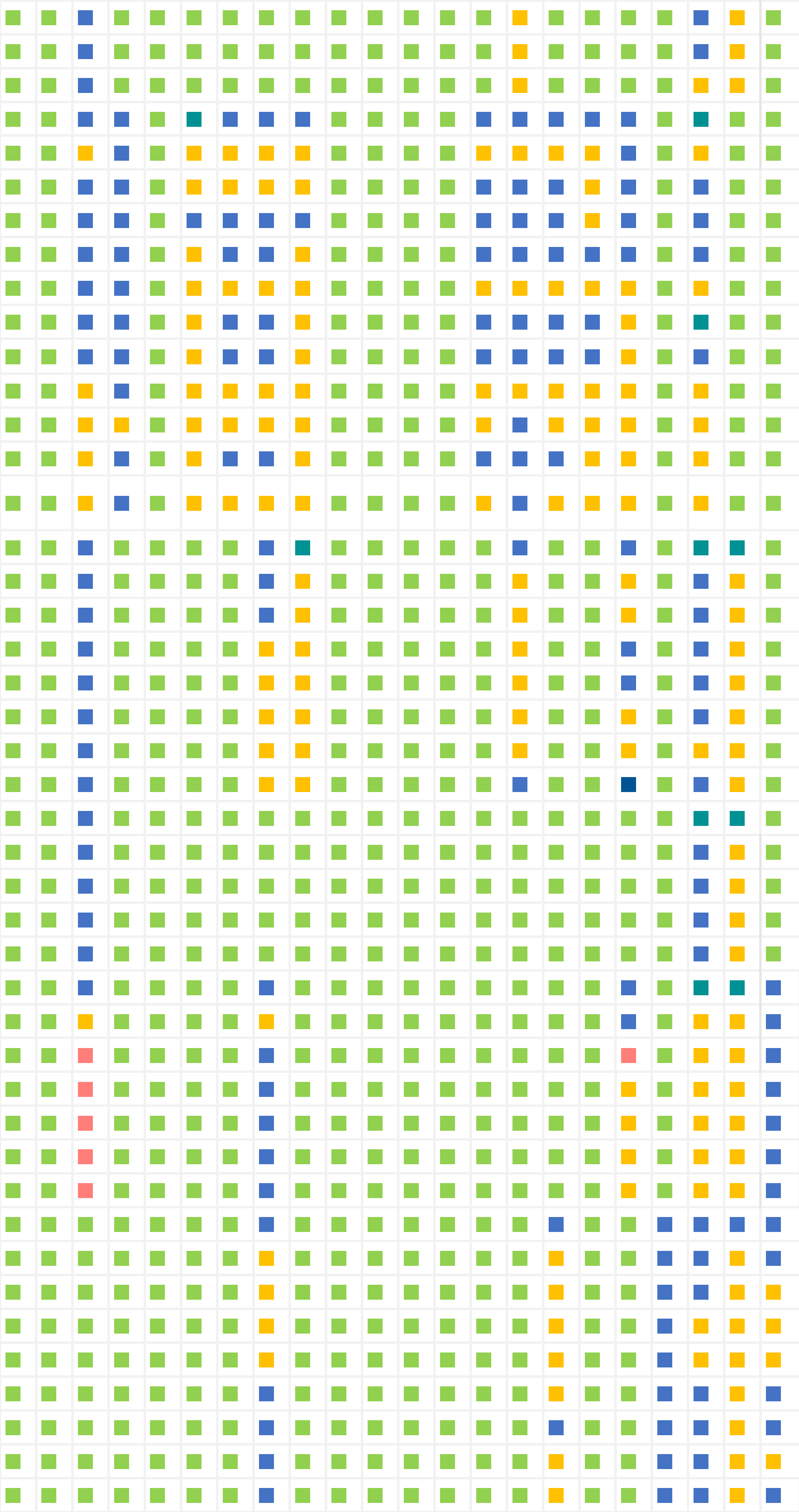

6.3 Rescue for inadequate neuraxial block (change anesthetic)	Provided
-Nitrous oxide and oxygen alone	Disagree
-Continue with neuraxial block, consider repeated boluses of fastacting opioids	Agree
--Fentanyl	25-50 µg
--Alfentanil	250-500 µg
--Ketamine	10 mg boluses
-Do not treat pain with anxiolytics	Agree
-Above options are not possible, general anesthesia should be recommended	Agree
**Management of Intra- or Postoperative Pain**	
**1. Implement strategies to minimize systemic opioid utilization**	Provided
**2. Neuraxial adjuvant drugs**	Provided
-Spinal anesthesia	Provided
--Morphine	50-100 µg
--Hydromorphone	75-150 µg
-Epidural or combined spinal-epidural technique.	Provided
--Morphine	2-3 mg
--Diamorphine	2-3 mg
**3. Others analgesic (after delivery)**	Provided
-Non-steroidal anti-inflammatory drug (e.g., paracetamol)	Agree
-A single dose of intravenous dexamethasone	Agree
**4. Local anesthetic infiltration**	Agree
**5. Continuous wound local anesthetic infusion**	Agree
**6. Fascial plane blocks (e.g., transversus abdominis plane, erector spinae plane)**	Agree
**7. Patient-controlled intravenous analgesia**	Agree

For peer review only

PRISMA 2020 Checklist

Section and Topic	Item #	Checklist item	Location where item is reported
**TITLE**			Page 1
Title	1	Identify the report as a systematic review.	
**ABSTRACT**			Page 2
Abstract	2	See the PRISMA 2020 for Abstracts checklist.	
**INTRODUCTION**			Page 5
Rationale	3	Describe the rationale for the review in the context of existing knowledge.	
Objectives	4	Provide an explicit statement of the objective(s) or question(s) the review addresses.	
**METHODS**			Page 6 to 9
Eligibility criteria	5	Specify the inclusion and exclusion criteria for the review and how studies were grouped for the syntheses.	
Information sources	6	Specify all databases, registers, websites, organisations, reference lists and other sources searched or consulted to identify studies. Specify the date when each source was last searched or consulted.	
Search strategy	7	Present the full search strategies for all databases, registers and websites, including any filters and limits used.	
Selection process	8	Specify the methods used to decide whether a study met the inclusion criteria of the review, including how many reviewers screened each record and each report retrieved, whether they worked independently, and if applicable, details of automation tools used in the process.	
Data collection process	9	Specify the methods used to collect data from reports, including how many reviewers collected data from each report, whether they worked independently, any processes for obtaining or confirming data from study investigators, and if applicable, details of automation tools used in the process.	
Data items	10a	List and define all outcomes for which data were sought. Specify whether all results that were compatible with each outcome domain in each study were sought (e.g. for all measures, time points, analyses), and if not, the methods used to decide which results to collect.	
	10b	List and define all other variables for which data were sought (e.g. participant and intervention characteristics, funding sources). Describe any assumptions made about any missing or unclear information.	
Study risk of bias assessment	11	Specify the methods used to assess risk of bias in the included studies, including details of the tool(s) used, how many reviewers assessed each study and whether they worked independently, and if applicable, details of automation tools used in the process.	
Effect measures	12	Specify for each outcome the effect measure(s) (e.g. risk ratio, mean difference) used in the synthesis or presentation of results.	
Synthesis methods	13a	Describe the processes used to decide which studies were eligible for each synthesis (e.g. tabulating the study intervention characteristics and comparing against the planned groups for each synthesis (item #5)).	
	13b	Describe any methods required to prepare the data for presentation or synthesis, such as handling of missing summary statistics, or data conversions.	
	13c	Describe any methods used to tabulate or visually display results of individual studies and syntheses.	
	13d	Describe any methods used to synthesize results and provide a rationale for the choice(s). If meta-analysis was performed, describe the model(s), method(s) to identify the presence and extent of statistical heterogeneity, and software package(s) used.	
	13e	Describe any methods used to explore possible causes of heterogeneity among study results (e.g. subgroup analysis, meta-regression).	
	13f	Describe any sensitivity analyses conducted to assess robustness of the synthesized results.	
Reporting bias assessment	14	Describe any methods used to assess risk of bias due to missing results in a synthesis (arising from reporting biases).	
Certainty	15	Describe any methods used to assess certainty (or confidence) in the body of evidence for an outcome.	

For peer review only

PRISMA 2020 Checklist

Section and Topic	Item #	Checklist item	Location where item is reported
assessment			
**RESULTS**			Page 10 to 21
Study selection	16a	Describe the results of the search and selection process, from the number of records identified in the search to the number of studies included in the review, ideally using a flow diagram.	
	16b	Cite studies that might appear to meet the inclusion criteria, but which were excluded, and explain why they were excluded.	
Study characteristics	17	Cite each included study and present its characteristics.	
Risk of bias in studies	18	Present assessments of risk of bias for each included study.	
Results of individual studies	19	For all outcomes, present, for each study: (a) summary statistics for each group (where appropriate) and (b) an effect estimate and its precision (e.g. confidence/credible interval), ideally using structured tables or plots.	
Results of syntheses	20a	For each synthesis, briefly summarise the characteristics and risk of bias among contributing studies.	
	20b	Present results of all statistical syntheses conducted. If meta-analysis was done, present for each the summary estimate and its precision (e.g. confidence/credible interval) and measures of statistical heterogeneity. If comparing groups, describe the direction of the effect.	
	20c	Present results of all investigations of possible causes of heterogeneity among study results.	
	20d	Present results of all sensitivity analyses conducted to assess the robustness of the synthesized results.	
Reporting biases	21	Present assessments of risk of bias due to missing results (arising from reporting biases) for each synthesis assessed.	
Certainty of evidence	22	Present assessments of certainty (or confidence) in the body of evidence for each outcome assessed.	
**DISCUSSION**			Page 21 to 26
Discussion	23a	Provide a general interpretation of the results in the context of other evidence.	
	23b	Discuss any limitations of the evidence included in the review.	
	23c	Discuss any limitations of the review processes used.	
	23d	Discuss implications of the results for practice, policy, and future research.	
**OTHER INFORMATION**			Page 27
Registration and protocol	24a	Provide registration information for the review, including register name and registration number, or state that the review was not registered.	
	24b	Indicate where the review protocol can be accessed, or state that a protocol was not prepared.	
	24c	Describe and explain any amendments to information provided at registration or in the protocol.	
Support	25	Describe sources of financial or non-financial support for the review, and the role of the funders or sponsors in the review.	
Competing interests	26	Declare any competing interests of review authors.	
Availability of data, code and other materials	27	Report which of the following are publicly available and where they can be found: template data collection forms; data extracted from included studies; data used for all analyses; analytic code; any other materials used in the review.	

For peer review only

For peer review only - http://bmjopen.bmj.com/site/about/guidelines.xhtml

PRISMA 2020 Checklist

From: Page MJ, McKenzie JE, Bossuyt PM, Boutron I, Hoffmann TC, Mulrow CD, et al. The PRISMA 2020 statement: an updated guideline for reporting systematic reviews. BMJ 2021;372:n71. doi: 10.1136/bmj.n71

For more information, visit: http://www.prisma-statement.org/

For peer review only
